# Supplementary material for: Synthesis, Biological Evaluation and Docking Studies of Ring-Opened Analogues of Ipomoeassin F
Source: Molecules. 2022 Jul 10;27(14):4419. doi: 10.3390/molecules27144419 (PMC9320607; doi:10.3390/molecules27144419)

Supplementary Material S1:

Synthesis, Biological Evaluation and Docking Studies of Ring-Opened Analogues of Ipomoeassin F

Sarah O'Keefe, Pratiti Bhadra, Kwabena B. Duah, Guanghui Zong, Levise Tenay, Lauren Andrews, Hayden Schneider, Ashley Anderson, Zhijian Hu, Hazim S. Aljewari, Belinda S. Hall, *Rachel E. Simmonds,* Volkhard Helms, Stephen High and Wei Q. Shi

National Cancer Institute Developmental Therapeutics Program Dose Response Curves	NSC: D - 791001 / 1	SSPL: 1A0E	EXP. ID: 1606NS35
	Report Date: October 22, 2016	Test Date: June 27, 2016	

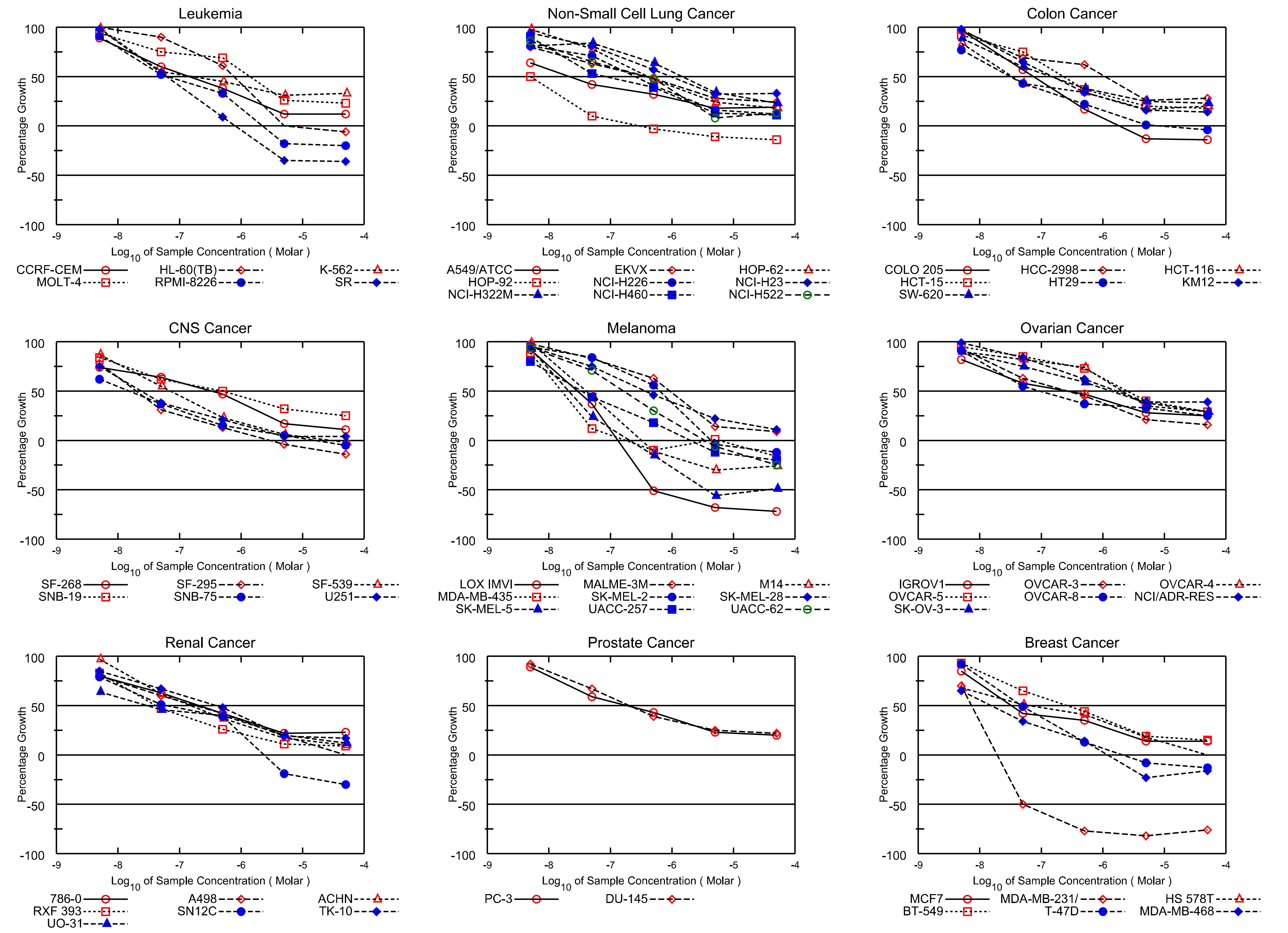

National Cancer Institute Developmental Therapeutics Program
In-Vitro Testing Results

NSC : D - 791001 / 1	Experiment ID : 1606NS35	Test Type : 08	Units : Molar
Report Date : October 22, 2016	Test Date : June 27, 2016	QNS :	MC :
COMI : ZIF-3	Stain Reagent : SRB Dual-Pass Related	SSPL : 1A0E	

	Log10 Concentration														
	Time		Mean Optical Densities					Percent Growth							
Panel/Cell Line	Zero	Ctrl	-8.3	-7.3	-6.3	-5.3	-4.3	-8.3	-7.3	-6.3	-5.3	-4.3	GI50	TGI	LC50
Leukemia															
CCRF-CEM	0.461	2.206	2.022	1.513	1.123	0.679	0.667	89	60	38	12	12	1.44E-7	> 5.00E-5	> 5.00E-5
HL-60(TB)	1.083	3.227	3.236	3.021	2.398	1.079	1.021	100	90	61	.	-6	7.63E-7	4.92E-6	> 5.00E-5
K-562	0.291	2.158	2.134	1.313	1.135	0.869	0.900	99	55	45	31	33	1.57E-7	> 5.00E-5	> 5.00E-5
MOLT-4	0.724	2.775	2.662	2.261	2.146	1.253	1.206	94	75	69	26	23	1.39E-6	> 5.00E-5	> 5.00E-5
RPMI-8226	0.958	2.741	2.573	1.886	1.546	0.784	0.763	91	52	33	-18	-20	6.39E-8	2.20E-6	> 5.00E-5
SR	0.525	1.822	1.793	1.222	0.637	0.340	0.334	98	54	9	-35	-36	6.06E-8	7.86E-7	> 5.00E-5
Non-Small Cell Lung Cancer															
A549/ATCC	0.394	1.610	1.172	0.899	0.778	0.607	0.629	64	42	32	18	19	2.09E-8	> 5.00E-5	> 5.00E-5
EKVX	0.601	1.848	1.593	1.383	1.205	0.954	0.901	80	63	48	28	24	3.90E-7	> 5.00E-5	> 5.00E-5
HOP-62	0.716	1.525	1.509	1.348	1.096	0.907	0.861	98	78	47	24	18	3.99E-7	> 5.00E-5	> 5.00E-5
HOP-92	1.270	1.783	1.525	1.319	1.227	1.125	1.098	50	10	-3	-11	-14	< 5.00E-9	2.72E-7	> 5.00E-5
NCI-H226	1.017	1.773	1.630	1.550	1.330	1.139	1.109	81	71	41	16	12	2.53E-7	> 5.00E-5	> 5.00E-5
NCI-H23	0.524	1.771	1.697	1.531	1.241	0.928	0.930	94	81	57	32	33	9.93E-7	> 5.00E-5	> 5.00E-5
NCI-H322M	0.885	2.177	1.927	1.969	1.713	1.319	1.177	81	84	64	34	23	1.45E-6	> 5.00E-5	> 5.00E-5
NCI-H460	0.306	2.670	2.433	1.550	1.230	0.620	0.565	90	53	39	13	11	7.81E-8	> 5.00E-5	> 5.00E-5
NCI-H522	0.925	1.968	1.822	1.604	1.424	1.008	1.057	86	65	48	8	13	3.74E-7	> 5.00E-5	> 5.00E-5
Colon Cancer															
COLO 205	0.469	1.640	1.602	1.133	0.665	0.408	0.405	97	57	17	-13	-14	7.35E-8	1.82E-6	> 5.00E-5
HCC-2998	0.837	2.837	2.771	2.213	2.077	1.352	1.392	97	69	62	26	28	1.07E-6	> 5.00E-5	> 5.00E-5
HCT-116	0.561	2.999	2.612	1.612	1.400	0.970	1.047	84	43	34	17	20	3.39E-8	> 5.00E-5	> 5.00E-5
HCT-15	0.384	2.346	2.191	1.848	1.119	0.783	0.731	92	75	37	20	18	2.30E-7	> 5.00E-5	> 5.00E-5
HT29	0.313	1.685	1.372	0.904	0.610	0.326	0.302	77	43	22	1	-4	3.13E-8	7.90E-6	> 5.00E-5
KM12	0.500	2.842	2.792	2.020	1.300	0.869	0.828	98	65	34	16	14	1.53E-7	> 5.00E-5	> 5.00E-5
SW-620	0.291	1.770	1.603	1.179	0.846	0.656	0.631	89	60	38	25	23	1.40E-7	> 5.00E-5	> 5.00E-5
CNS Cancer															
SF-268	0.578	1.988	1.618	1.474	1.242	0.814	0.739	74	64	47	17	11	3.31E-7	> 5.00E-5	> 5.00E-5
SF-295	0.996	2.800	2.406	1.550	1.232	0.956	0.860	78	31	13	-4	-14	1.96E-8	2.89E-6	> 5.00E-5
SF-539	1.027	2.912	2.670	2.063	1.468	1.146	1.010	87	55	23	6	-2	7.16E-8	3.09E-5	> 5.00E-5
SNB-19	0.423	1.651	1.455	1.183	1.035	0.810	0.727	84	62	50	32	25	4.87E-7	> 5.00E-5	> 5.00E-5
SNB-75	0.868	1.733	1.401	1.187	0.999	0.907	0.829	62	37	15	5	-5	1.47E-8	1.57E-5	> 5.00E-5
U251	0.434	1.737	1.408	0.923	0.708	0.488	0.490	75	38	21	4	4	2.31E-8	> 5.00E-5	> 5.00E-5
Melanoma															
LOX IMVI	0.380	2.415	2.243	1.129	0.186	0.124	0.106	92	37	-51	-68	-72	2.87E-8	1.31E-7	4.85E-7
MALME-3M	0.623	1.130	1.119	1.043	0.941	0.694	0.669	98	83	63	14	9	9.10E-7	> 5.00E-5	> 5.00E-5
M14	0.574	2.045	2.035	1.239	0.509	0.399	0.423	99	45	-11	-30	-26	4.08E-8	3.14E-7	> 5.00E-5
MDA-MB-435	0.465	2.368	2.165	0.697	0.420	0.486	0.393	89	12	-10	1	-16	1.62E-8	.	> 5.00E-5
SK-MEL-2	0.978	1.909	1.866	1.758	1.502	0.939	0.861	95	84	56	-4	-12	6.35E-7	4.29E-6	> 5.00E-5
SK-MEL-28	0.742	2.207	2.135	1.839	1.423	1.057	0.909	95	75	46	22	11	3.75E-7	> 5.00E-5	> 5.00E-5
SK-MEL-5	0.770	2.691	2.554	1.230	0.652	0.339	0.396	93	24	-15	-56	-49	2.09E-8	2.03E-7	.
UACC-257	1.062	2.002	1.815	1.473	1.230	0.934	0.851	80	44	18	-12	-20	3.36E-8	1.98E-6	> 5.00E-5
UACC-62	0.761	2.871	2.739	2.254	1.391	0.718	0.567	94	71	30	-6	-25	1.61E-7	3.45E-6	> 5.00E-5
Ovarian Cancer															
IGROV1	0.585	2.011	1.758	1.408	1.254	0.989	0.940	82	58	47	28	25	2.58E-7	> 5.00E-5	> 5.00E-5
OVCAR-3	0.533	1.990	1.892	1.456	1.186	0.843	0.769	93	63	45	21	16	2.62E-7	> 5.00E-5	> 5.00E-5
OVCAR-4	0.776	1.673	1.581	1.514	1.437	1.094	1.034	90	82	74	35	29	2.08E-6	> 5.00E-5	> 5.00E-5
OVCAR-5	0.742	1.903	1.845	1.729	1.587	1.211	1.077	95	85	73	40	29	2.53E-6	> 5.00E-5	> 5.00E-5
OVCAR-8	0.509	1.908	1.775	1.276	1.030	0.965	0.863	91	55	37	33	25	9.42E-8	> 5.00E-5	> 5.00E-5
NCI/ADR-RES	0.502	1.732	1.723	1.538	1.268	0.982	0.977	99	84	62	39	39	1.68E-6	> 5.00E-5	> 5.00E-5
SK-OV-3	0.912	2.092	1.970	1.791	1.604	1.355	1.252	90	75	59	38	29	1.28E-6	> 5.00E-5	> 5.00E-5
Renal Cancer															
786-0	0.656	2.211	1.904	1.629	1.310	0.996	1.008	80	63	42	22	23	2.05E-7	> 5.00E-5	> 5.00E-5
A498	1.368	2.033	1.897	1.768	1.645	1.498	1.368	80	60	42	19	.	1.76E-7	> 5.00E-5	> 5.00E-5
ACHN	0.446	1.962	1.922	1.380	1.013	0.708	0.593	97	62	37	17	10	1.51E-7	> 5.00E-5	> 5.00E-5
RXF 393	0.773	1.646	1.495	1.184	1.003	0.870	0.851	83	47	26	11	9	4.13E-8	> 5.00E-5	> 5.00E-5
SN12C	0.516	2.184	1.840	1.365	1.159	0.421	0.363	79	51	39	-19	-30	5.92E-8	2.37E-6	> 5.00E-5
TK-10	1.079	2.163	1.998	1.804	1.594	1.283	1.262	85	67	48	19	17	3.73E-7	> 5.00E-5	> 5.00E-5
UO-31	0.629	1.749	1.350	1.147	1.080	0.857	0.768	64	46	40	20	12	3.11E-8	> 5.00E-5	> 5.00E-5
Prostate Cancer															
PC-3	0.617	1.662	1.547	1.234	1.067	0.862	0.830	89	59	43	23	20	1.83E-7	> 5.00E-5	> 5.00E-5
DU-145	0.388	1.689	1.581	1.254	0.894	0.715	0.674	92	67	39	25	22	1.98E-7	> 5.00E-5	> 5.00E-5
Breast Cancer															
MCF7	0.444	2.051	1.817	1.117	1.014	0.673	0.661	85	42	35	14	14	3.25E-8	> 5.00E-5	> 5.00E-5
MDA-MB-231/ATCC	0.547	1.366	1.121	0.273	0.127	0.096	0.130	70	-50	-77	-82	-76	7.34E-9	1.91E-8	4.99E-8
HS 578T	1.100	2.018	1.725	1.565	1.472	1.265	1.104	68	51	41	18	.	5.82E-8	> 5.00E-5	> 5.00E-5
BT-549	1.182	2.274	2.195	1.888	1.663	1.388	1.348	93	65	44	19	15	2.56E-7	> 5.00E-5	> 5.00E-5
T-47D	0.873	1.607	1.547	1.231	0.965	0.803	0.757	92	49	13	-8	-13	4.67E-8	2.04E-6	> 5.00E-5
MDA-MB-468	0.706	1.423	1.172	0.949	0.807	0.546	0.593	65	34	14	-23	-16	1.51E-8	1.21E-6	> 5.00E-5

National Cancer Institute Developmental Therapeutics Program Mean Graphs	NSC : D - 791001/1	Units :Molar	SSPL :1A0E	EXP. ID :1606NS35
	Report Date :October 22, 2016		Test Date :June 27, 2016	

Panel/Cell Line	Log_{10}GI50	GI50	Log_{10}TGI	TGI	Log_{10}LC50	LC50
**Leukemia**						
CCRF-CEM	-6.84		> -4.30		> -4.30	
HL-60(TB)	-6.12		-5.31		> -4.30	
K-562	-6.80		> -4.30		> -4.30	
MOLT-4	-5.86		> -4.30		> -4.30	
RPMI-8226	-7.19		-5.66		> -4.30	
SR	-7.22		-6.10		> -4.30	
**Non-Small Cell Lung Cancer**						
A549/ATCC	-7.68		> -4.30		> -4.30	
EKVX	-6.41		> -4.30		> -4.30	
HOP-62	-6.40		> -4.30		> -4.30	
HOP-92	< -8.30		-6.56		> -4.30	
NCI-H226	-6.60		> -4.30		> -4.30	
NCI-H23	-6.00		> -4.30		> -4.30	
NCI-H322M	-5.84		> -4.30		> -4.30	
NCI-H460	-7.11		> -4.30		> -4.30	
NCI-H522	-6.43		> -4.30		> -4.30	
**Colon Cancer**						
COLO 205	-7.13		-5.74		> -4.30	
HCC-2998	-5.97		> -4.30		> -4.30	
HCT-116	-7.47		> -4.30		> -4.30	
HCT-15	-6.64		> -4.30		> -4.30	
HT29	-7.50		-5.10		> -4.30	
KM12	-6.82		> -4.30		> -4.30	
SW-620	-6.86		> -4.30		> -4.30	
**CNS Cancer**						
SF-268	-6.48		> -4.30		> -4.30	
SF-295	-7.71		-5.54		> -4.30	
SF-539	-7.14		-4.51		> -4.30	
SNB-19	-6.31		> -4.30		> -4.30	
SNB-75	-7.83		-4.80		> -4.30	
U251	-7.64		> -4.30		> -4.30	
**Melanoma**						
LOX IMVI	-7.54		-6.88		-6.31	
MALME-3M	-6.04		> -4.30		> -4.30	
M14	-7.39		-6.50		> -4.30	
MDA-MB-435	-7.79				> -4.30	
SK-MEL-2	-6.20		-5.37		> -4.30	
SK-MEL-28	-6.43		> -4.30		> -4.30	
SK-MEL-5	-7.68		-6.69			
UACC-257	-7.47		-5.70		> -4.30	
UACC-62	-6.79		-5.46		> -4.30	
**Ovarian Cancer**						
IGROV1	-6.59		> -4.30		> -4.30	
OVCAR-3	-6.58		> -4.30		> -4.30	
OVCAR-4	-5.68		> -4.30		> -4.30	
OVCAR-5	-5.60		> -4.30		> -4.30	
OVCAR-8	-7.03		> -4.30		> -4.30	
NCI/ADR-RES	-5.77		> -4.30		> -4.30	
SK-OV-3	-5.89		> -4.30		> -4.30	
**Renal Cancer**						
786-0	-6.69		> -4.30		> -4.30	
A498	-6.75		> -4.30		> -4.30	
ACHN	-6.82		> -4.30		> -4.30	
RXF 393	-7.38		> -4.30		> -4.30	
SN12C	-7.23		-5.63		> -4.30	
TK-10	-6.43		> -4.30		> -4.30	
UO-31	-7.51		> -4.30		> -4.30	
**Prostate Cancer**						
PC-3	-6.74		> -4.30		> -4.30	
DU-145	-6.70		> -4.30		> -4.30	
**Breast Cancer**						
MCF7	-7.49		> -4.30		> -4.30	
MDA-MB-231/ATCC	-8.13		-7.72		-7.30	
HS 578T	-7.24		> -4.30		> -4.30	
BT-549	-6.59		> -4.30		> -4.30	
T-47D	-7.33		-5.69		> -4.30	
MDA-MB-468	-7.82		-5.92		> -4.30	
_MID	-6.87		-4.8		-4.39	
Delta	1.43		2.92		2.91	
Range	2.7		3.42		3.0	

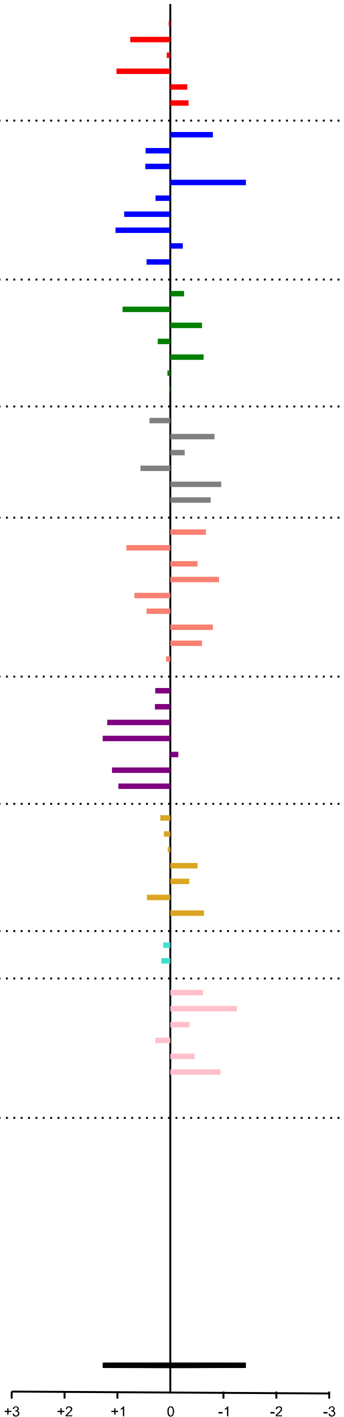

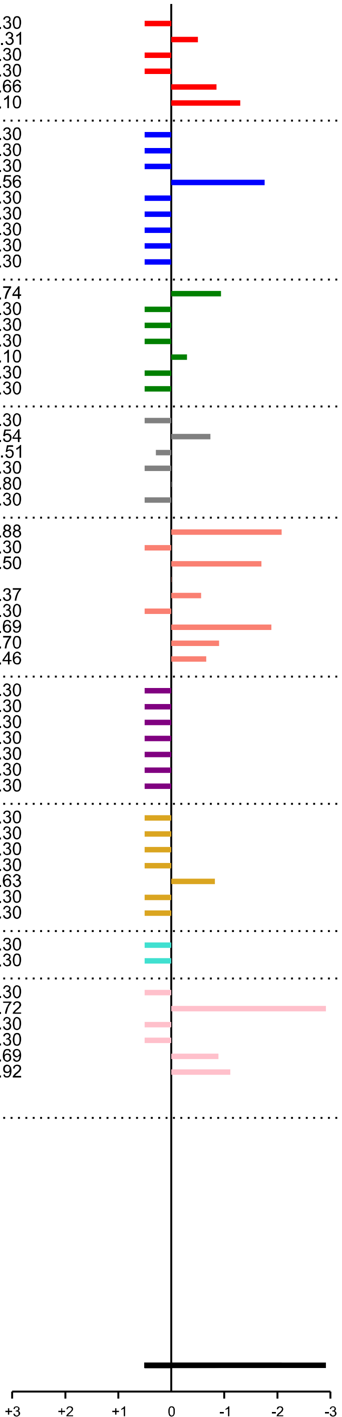

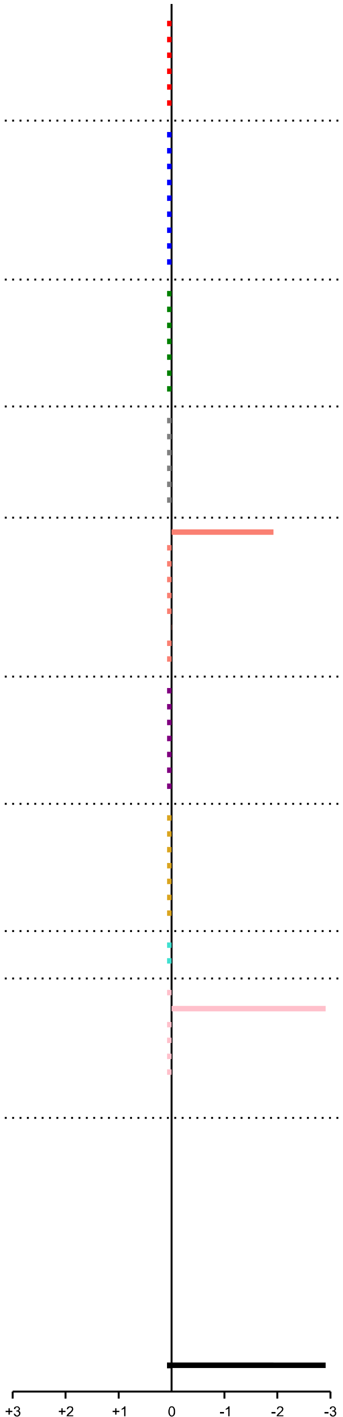

National Cancer Institute Developmental Therapeutics Program Dose Response Curves	NSC : D - 791001/1	SSPL:1A0E	EXP. ID:1606NS35
	Report Date:October 22, 2016	Test Date:June 27, 2016	

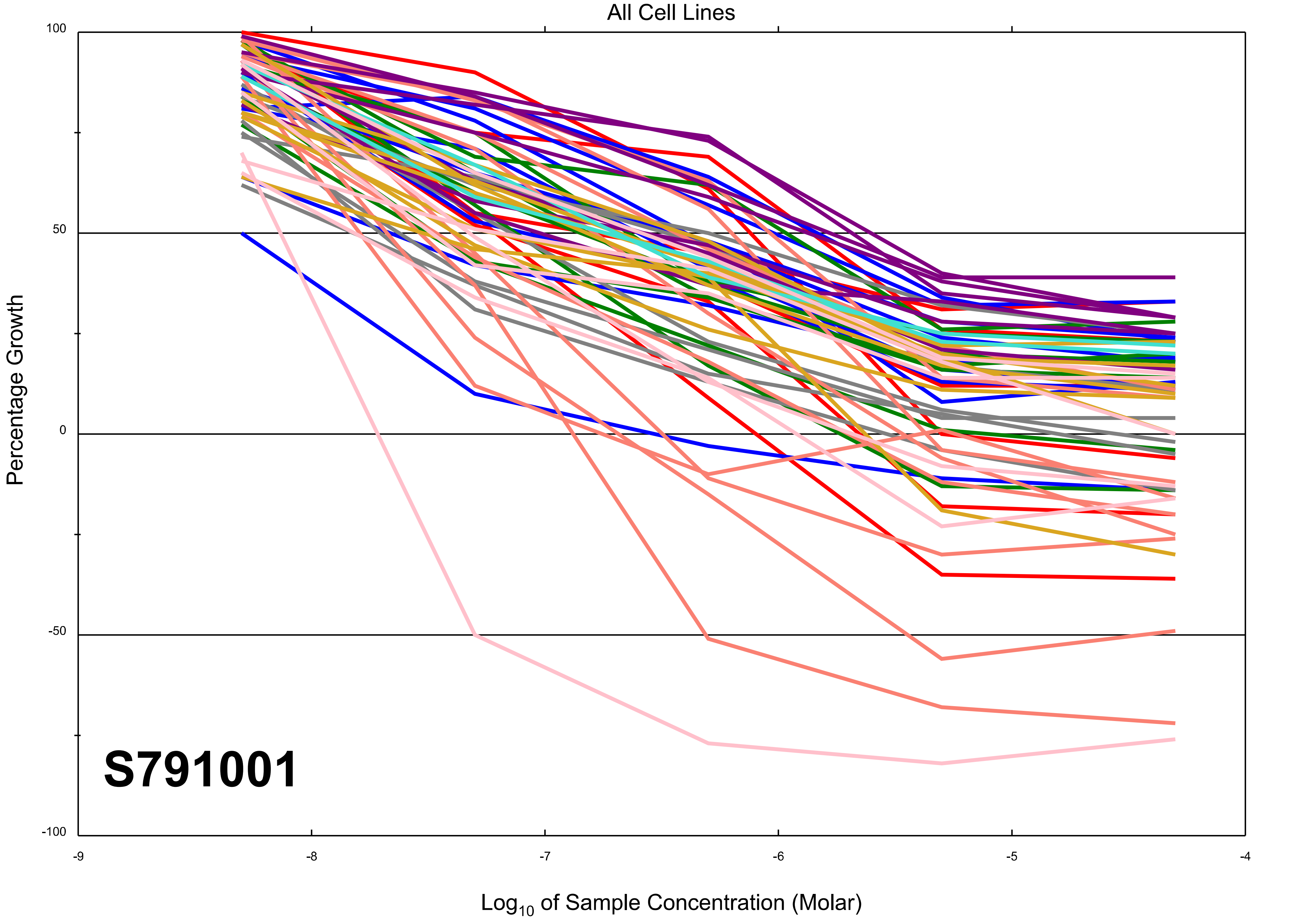

Supplement: Supplementary file 1 [file molecules-27-04419-s001.zip › Supplementary material S1.pdf]
