# Supplementary material for: Synthesis, Biological Evaluation and Docking Studies of Ring-Opened Analogues of Ipomoeassin F
Source: Molecules. 2022 Jul 10;27(14):4419. doi: 10.3390/molecules27144419 (PMC9320607; doi:10.3390/molecules27144419)
Supplement: Supplementary file 1 [file molecules-27-04419-s001.zip › Supplementary material S2.pdf]

Supplementary Material S2:

Synthesis, Biological Evaluation and Docking Studies of Ring-Opened Analogues of Ipomoeassin F

Sarah O'Keefe, Pratiti Bhadra, Kwabena B. Duah, Guanghui Zong, Levise Tenay, Lauren Andrews, Hayden Schneider, Ashley Anderson, Zhijian Hu, Hazim S. Aljewari, Belinda S. Hall, *Rachel E. Simmonds,* Volkhard Helms, Stephen High and Wei Q. Shi

Table of Contents

Chemistry Part I: Synthesis of open-chain analogues **4–12**	S3-S14
Chemistry Part II: Synthesis of the fucose acceptor **24**	S15-S17
Figures S1-S6 and Table S1	S18-S24
References	S25

Chemistry

Part I: Synthesis of open-chain analogues 4–12

General methods: Reactions were carried out in oven-dried glassware. All reagents were purchased from commercial sources and were used without further purification unless noted. Unless stated otherwise, all reactions were carried out under a nitrogen atmosphere and monitored by thin layer chromatography (TLC) using Silica Gel GF_{254} plates (Agela) with detection by charring with 5% (v/v) H_2SO_4 in EtOH or by visualizing in UV light (254 nm). Column chromatography was performed on silica gel (230–450 mesh, Sorbent). The ratio between silica gel and crude product ranged from 100 to 50:1 (w/w). NMR data were collected on a Bruker 400 MHz NMR spectrometer and a Bruker 400 MHz system. ^{1}H NMR spectra were obtained in deuteriochloroform ($CDCl_3$) with chloroform ($CHCl_3$, δ = 7.27 for ^{1}H) as an internal reference. ^{13}C NMR spectra were proton decoupled and were in $CDCl_3$ with $CHCl_3$ (δ = 77.0 for ^{13}C) as an internal reference. Chemical shifts are reported in ppm (δ). Data are presented in the form: chemical shift (multiplicity, coupling constants, and integration). ^{1}H data are reported as though they were first order. The errors between the coupling constants for two coupled protons were less than 0.5 Hz, and the average number was reported. Proton assignments, when made, were done so with the aid of COSY NMR spectra. For some compounds, HSQC and HMBC NMR were also applied to assign the proton signals. Optical rotations were measured on a Autopol III Automatic Polarimeter at 25 ± 1 ^{o}C for solutions in a 1.0 dm cell.

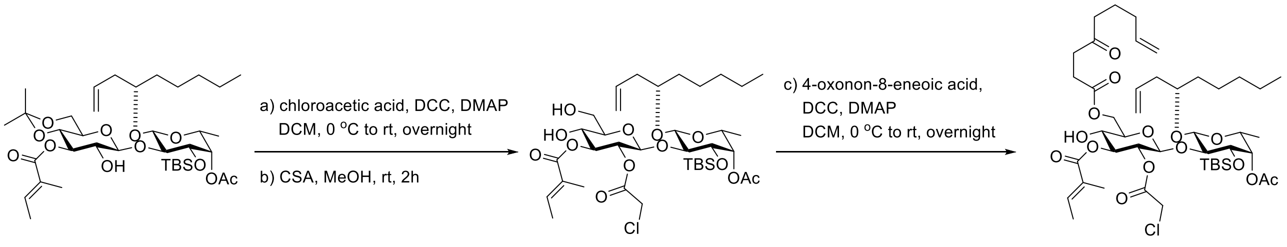

Compound S2. DCC (1.36 g, 6.6 mmol) was added in one portion to a 0°C CH_2Cl_2 (30 mL) solution of **S1** [1] (3.21 g, 4.4 mmol), chloroacetic acid (746 mg, 6.6 mmol) and 4-dimethylaminopyridine (54 mg, 0.44 mmol). The reaction was allowed to warm to ambient temperature and stirred overnight. At this point, TLC (silica, 1:9 EtOAc–hexanes) showed the reaction was complete. The reaction mixture was diluted with ether (40 mL) and hexanes (20 mL), stirred for 20 minutes then filtered thru a pad of celite using ether (20 mL) as the eluent and the filtrate concentrated *in vacuo*. The

residue was purified by column chromatography (silica, EtOAc–hexanes, 1:15 → 1:9) gave chloroacetyl protected intermediate (3.45 g, 97%) as a white foam. CSA (200 mg, 0.86 mmol) was added in one portion to a solution of the obtained product in MeOH (40 mL) at room temperature. The reaction mixture was stirred for 3 h at which point TLC (silica, 1:3 EtOAc–hexanes) showed it was complete. The reaction was quenched with Et_3N (250 µL) and concentrated. The residue was purified by column chromatography (silica, EtOAc–hexanes, 1: 3→ 1:2) gave compound **S2** (2.78 g, 85%) as a white foam. $[\alpha]_D^{25}$ 11.3° (*c* 1 $CHCl_3$). ^{1}H NMR (400 MHz, $CDCl_3$) δ 6.95 – 6.84 (m, 1H, Me-C*H*-C(Me)-C=O), 6.05 – 5.88 (m, 1H, CH_2=C*H*-CH_2-), 5.16 – 4.90 (m, 6H, CH_2=CH-CH_2-, H-1-Glc*p*, H-2-Glc*p*, H-3-Glc*p*, H-4-Fuc*p*), 4.29 (d, *J* = 7.6 Hz, 1H, H-1-Fuc*p*), 4.09 – 3.92 (m, 3H, Cl-CH_2-C=O, H-6-Glc*p*), 3.91 – 3.85 (m, 1H, H-2-Fuc*p*), 3.84 – 3.73 (m, 3H, H-3-Fuc*p*, H-4-Glc*p*, H-6-Glc*p*), 3.67 – 3.56 (m, 2H, H-5-Fuc*p*, -CH_2-C*H*-CH_2-), 3.45 – 3.40 (m, 1H, H-5-Glc*p*), 3.23 (d, *J* = 4.0 Hz, 1H, O*H*), 2.41 – 2.28 (m, 3H), 2.14 (s, 3H, CH_3-C=O), 1.83 – 1.76 (m, 6H, CH_3-CH-C(CH_3)-C=O), 1.63 – 1.43 (m, 2H), 1.40 – 1.21 (m, 6H), 1.13 (d, *J* = 6.4 Hz, 3H, H-6-Fuc*p*), 0.93 – 0.80 (m, 12H), 0.11, 0.10 (2s, 6H, CH_3-Si). ^{13}C NMR (100 MHz, $CDCl_3$) δ 170.8, 168.9, 166.0, 140.3, 135.0, 127.4, 116.9, 101.2, 98.4, 80.5, 76.8, 75.4, 75.1, 73.7, 73.5, 73.2, 69.9, 68.9, 61.9, 40.7, 38.3, 34.3, 31.75, 25.8, 24.7, 22.6, 20.9, 17.7, 16.4, 14.7, 14.0, 11.94, -4.4, -4.6.

Compound S3. DCC (906 mg, 4.4 mmol) was added in one portion to a 0°C CH_2Cl_2 (150 mL) solution of **S2** (2.24 g, 2.93 mmol), 4-oxonon-8-eneoic acid (573 mg, 3.4 mmol) and 4-dimethylaminopyridine (36 mg, 0.29 mmol). The reaction was allowed to warm to ambient temperature and stirred overnight. At this point, TLC (silica, 1:3 EtOAc–hexanes) showed the reaction was complete. The reaction mixture was concentrated to a volume of around 20 mL and then diluted with ether (20 mL) and hexanes (10 mL), stirred for 20 minutes then filtered thru a pad of celite using ether (20 mL) as the eluent and the filtrate concentrated *in vacuo*. The residue was purified by column chromatography (silica, EtOAc–hexanes, 1:8 → 1:4) gave diene **S3** (1.75 g, 65%) as a white foam. $[\alpha]_D^{25}$ –5.9° (*c* 1 $CHCl_3$). ^{1}H NMR (400 MHz, $CDCl_3$) δ 6.98 – 6.81 (m, 1H, Me-C*H*-C(Me)-C=O), 6.10 – 5.93 (m, 1H, CH_2=C*H*-CH_2-), 5.82 – 5.66 (m, 1H, CH_2=C*H*-CH_2-), 5.15 – 4.94 (m, 8H, 2 × CH_2=CH-CH_2-, H-1-Glc*p*, H-2-Glc*p*, H-3-Glc*p*, H-4-Fuc*p*), 4.56 (dd, *J* = 12.4, 4.0 Hz, 1H, H-6-Glc*p*), 4.35 – 4.25 (m, 2H, H-6-Glc*p*, H-1-Fuc*p*), 4.13 – 4.01 (m, 2H, Cl-CH_2-C=O), 3.94 – 3.87 (m, 1H, H-2-

Fucp), 3.80 – 3.68 (m, 2H, H-3-Fucp, H-4-Glcp), 3.67 – 3.56 (m, 2H, H-5-Fucp, -CH_2-C*H*-CH_2-), 3.55 – 3.46 (m, 1H, H-5-Glcp), 3.36 (d, *J* = 4.4 Hz, 1H, O*H*), 2.86 – 2.52 (m, 4H), 2.46 (t, *J* = 7.6 Hz, 2H), 2.38 – 2.27 (m, 2H), 2.13 (s, 3H, CH_3-C=O), 2.09 – 2.00 (m, 2H), 1.82 – 1.77 (m, 6H, CH_3-CH-C(CH_3)-C=O), 1.71 – 1.62 (m, 2H), 1.60 – 1.48 (m, 2H), 1.40 – 1.20 (m, 6H), 1.13 (d, *J* = 6.4 Hz, 3H, H-6-Fucp), 0.93 – 0.80 (m, 12H), 0.11 (s, 6H, CH_3-Si). ^{13}C NMR (100 MHz, $CDCl_3$) δ 208.8, 173.3, 170.7, 168.6, 165.9, 139.8, 137.8, 135.4, 127.5, 116.6, 115.3, 101.4, 98.7, 80.5, 75.9, 75.3, 74.3, 74.1, 73.4, 69.0, 68.9, 62.9, 41.7, 40.7, 38.4, 37.1, 34.3, 33.0, 31.8, 27.8, 25.8(×3), 24.6, 22.7, 22.6, 20.9, 17.8, 16.5, 14.6, 14.0, 12.0, -4.4, -4.5.

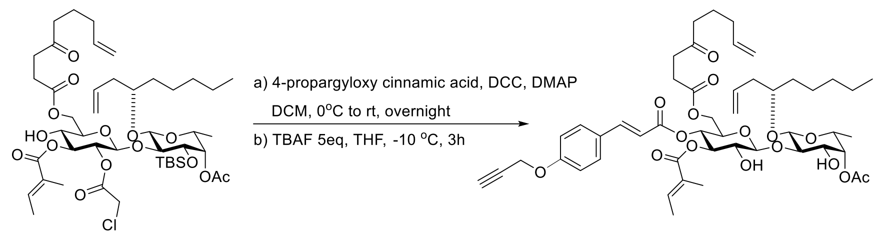

Analogue 4: 4-Propargyloxy cinnamic acid (17.6 mg, 0.087 mmol) was added in one portion to a solution of **S3** (40 mg, 0.044 mmol), DCC (18.0 mg, 0.087 mmol) and 4-dimethylaminopyridine (0.5 mg, 0.0044 mmol) in CH_2Cl_2 (3 mL). The reaction was stirred overnight at room temperature. At this point, TLC (silica, 1:4 EtOAc–hexanes) showed the reaction was complete. The reaction mixture was diluted with ether (3 ml) and hexanes (1.5 ml), stirred for 20 minutes then filtered thru a pad of celite using ether (3 ml) as the eluent and the filtrate concentrated *in vacuo*. To a solution of the obtained crude product in THF (3 mL) was added TBAF (1M solution in THF, 0.22 mL, 0.22 mmol, 5 equiv) at –10 °C. The reaction mixture was stirred at the same temperature for 3 h at which point TLC (silica, 1:1 EtOAc–hexanes) showed it was complete. The reaction mixture was diluted with Et_2O (30 mL), washed with 1M HCl (15 mL), saturated $NaHCO_3$ (15 mL), brine (15 mL). The aqueous layer was extracted with Et_2O (20 mL). The combined organic layer was dried over Na_2SO_4 and concentrated under reduced pressure. The residue was purified by column chromatography (silica, EtOAc–hexanes, 2:1→1:1) gave compound **4** (27.8 mg, 70%) as a colorless syrup. ^{1}H NMR (400 MHz, $CDCl_3$) δ 7.60 (d, *J* = 16.0 Hz, 1H, Ph-C*H*=CH-), 7.51 – 7.43 (m, 2H, 2 × Ar*H*), 7.04 – 6.94 (m, 2H, 2 × Ar*H*), 6.92 – 6.83 (m, 1H, Me-C*H*-C(Me)-C=O), 6.22 (d, *J* = 16.0 Hz, 1H, Ph-CH=C*H*-), 5.92 – 5.69 (m, 2H, CH_2=C*H*-CH_2-), 5.30 – 5.18 (m, 3H, H-3-Glcp, H-4-Glcp, H-4-Fucp), 5.14 – 4.92

(m, 4H, 2 × C*H*$_2$=CH-CH$_2$-), 4.76 – 4.68 (m, 3H, HC≡C-C*H*$_2$, H-1-Glc*p*), 4.46 (d, *J* = 7.2 Hz, 1H, H-1-Fuc*p*), 4.29 – 4.12 (m, 3H, H-6-Glc*p*, O*H*), 3.88 – 3.64 (m, 7H, H-2-Glc*p*, H-5-Glc*p*, H-2-Fuc*p*, H-3-Fuc*p*, H-5-Fuc*p*, -CH$_2$-C*H*-CH$_2$-, O*H*), 2.75 – 2.67 (m, 2H), 2.65 – 2.58 (m, 2H), 2.55 (t, *J* = 2.4 Hz, 1H), 2.44 (t, *J* = 7.4 Hz, 2H), 2.37 – 2.27 (m, 2H), 2.20 (s, 3H, C*H*$_3$-C=O), 2.08 – 2.01 (m, 2H), 1.79 – 1.73 (m, 6H), 1.71 – 1.63 (m, 3H), 1.58 – 1.50 (m, 2H), 1.43 – 1.22 (m, 6H), 1.19 (d, *J* = 6.4 Hz, 3H, H-6-Fuc*p*), 0.88 (t, *J* = 6.8 Hz, 3H).^{13}C NMR (100 MHz, CDCl$_3$) δ 208.6, 172.5, 171.3, 168.1, 165.7, 159.5, 145.9, 139.1, 138.0, 134.3, 129.9, 127.7, 127.5, 117.5, 115.2, 115.2, 114.4, 102.9, 99.6, 78.6, 78.2, 77.9, 76.0, 74.5, 72.7, 72.1, 71.8, 71.3, 69.2, 68.0, 62.4, 55.8, 41.8, 38.0, 37.0, 34.2, 33.0, 31.7, 27.7, 24.6, 22.7, 22.5, 20.9, 16.2, 14.5, 14.1, 12.0.

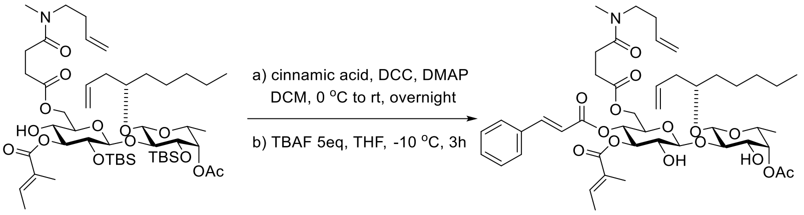

S4 **5**

Analogue 5: DCC (6.2 mg, 0.030 mmol) was added in one portion to a solution of **S4** [2] (19.4 mg, 0.020 mmol), cinnamic acid (4.4 mg, 0.030 mmol) and 4-dimethylaminopyridine (1.2 mg, 0.010 mmol) in CH_2Cl_2 (2 mL). The reaction was stirred at room temperature for 12 h. At this point, TLC (silica, 1:3 EtOAc–hexanes) showed the reaction was complete. The reaction mixture was diluted with ether (2 mL) and hexanes (1 mL), stirred for 20 minutes then filtered thru a pad of Celite using ether (5 mL) as the eluent and the filtrate was concentrated *in vacuo*. To a solution of the obtained crude product in THF (2 mL) was added TBAF (1M solution in THF, 100 μL, 100 μmol, 5 equiv) at –10 °C. The reaction mixture was stirred at the same temperature for 3 h at which point TLC (silica, 1:2 EtOAc–hexanes) showed it was complete. The reaction mixture was diluted with Et_2O (10 mL), washed with 1M HCl (5 mL), saturated $NaHCO_3$ (5 mL), brine (5 mL). The aqueous layer was extracted with Et_2O (10 mL). The combined organic layer was dried over Na_2SO_4 and concentrated under reduced pressure. The residue was purified by column chromatography (silica, EtOAc–hexanes, 1:1) gave analouge **5** (8.7 mg, 50% over two steps) as a colorless syrup. ^{1}H NMR (400 MHz, CDCl$_3$) δ 7.64 (d, *J* = 16.0 Hz, 1H, Ph-C*H*=C-), 7.55 – 7.46 (m, 2H, 2 × Ar*H*), 7.43 – 7.35 (m, 3H, 3 × Ar*H*), 6.93 –

6.82 (m, 1H, Me-C*H*-C(Me)-C=O), 6.34 (d, *J* = 16.0 Hz, 1H, Ph-CH=C*H*-), 5.94 – 5.68 (m, 2H, 2 × CH_2=C*H*-CH_2-), 5.32 – 5.17 (m, 3H, H-3-Glc*p*, H-4-Glc*p*, H-4-Fuc*p*), 5.15 – 4.98 (m, 4H, 2 × CH_2=CH-CH_2-), 4.73 (d, *J* = 8.0 Hz, 1H, H-1-Glc*p*), 4.46 (d, *J* = 7.2 Hz, 1H, H-1-Fuc*p*), 4.29 (dd, *J* = 12.4, 2.4 Hz, 1H, H-6-Glc*p*), 4.20 (dd, *J* = 12.4, 5.6 Hz, 1H, H-6-Glc*p*), 3.93 – 3.65 (m, 6H, H-2-Glc*p*, H-5-Glc*p*, H-2-Fuc*p*, H-3-Fuc*p*, H-5-Fuc*p*, -CH_2-C*H*-CH_2-), 3.46 – 3.32 (m, 2H), 2.99, 2.91 (2s, 3H), 2.75 – 2.50 (m, 4H), 2.39 – 2.23 (m, 4H), 2.21 (s, 3H, CH_3-C=O), 1.81 – 1.71 (m, 6H), 1.61 – 1.49 (m, 2H), 1.44 – 1.22 (m, 6H), 1.19 (d, *J* = 6.4 Hz, 3H, H-6-Fuc*p*), 0.88 (t, *J* = 6.8 Hz, 3H). ^{13}C NMR (100 MHz, $CDCl_3$) δ 172.9, 171.3, 170.8, 168.0, 165.5, 146.4, 139.1, 135.4, 134.3, 134.2, 134.0, 130.7, 128.9(×2), 128.3(×2), 127.7, 117.6, 117.6, 116.6, 116.5, 102.7, 102.7, 99.6, 99.5, 78.5, 77.9, 77.8, 74.4, 72.7, 72.2, 71.8, 71.2, 71.1, 69.2, 68.3, 62.5, 49.2, 47.4, 38.0, 35.4, 34.2, 33.5, 32.6, 31.9, 31.7, 29.3, 29.2, 28.3, 27.8, 24.7, 22.5, 21.0, 16.3, 14.5, 14.1, 12.0.

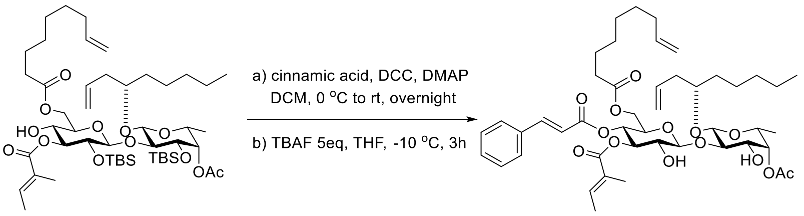

S5 **6**

Analogue 6: DCC (6.9 mg, 0.033 mmol) was added in one portion to a solution of **S5** [3] (21.0 mg, 0.022 mmol), cinnamic acid (5.0 mg, 0.033 mmol) and 4-dimethylaminopyridine (1.4 mg, 0.011 mmol) in CH_2Cl_2 (2 mL). The reaction was stirred at room temperature for 12 h. At this point, TLC (silica, 1:3 EtOAc–hexanes) showed the reaction was complete. The reaction mixture was diluted with ether (2 mL) and hexanes (1 mL), stirred for 20 minutes then filtered thru a pad of Celite using ether (5 mL) as the eluent and the filtrate was concentrated *in vacuo*. To a solution of the obtained crude product in THF (2 mL) was added TBAF (1M solution in THF, 100 µL, 100 µmol, 5 equiv) at –10 °C. The reaction mixture was stirred at the same temperature for 3 h at which point TLC (silica, 1:2 EtOAc–hexanes) showed it was complete. The reaction mixture was diluted with Et_2O (10 mL), washed with 1M HCl (5 mL), saturated $NaHCO_3$ (5 mL), brine (5 mL). The aqueous layer was extracted with Et_2O (10 mL). The combined organic layer was dried over Na_2SO_4 and concentrated under reduced pressure. The residue was purified by column chromatography (silica, EtOAc–hexanes, 1:1) gave analogue **6** (12.0 mg, 64% over

two steps) as a colorless syrup. ^{1}H NMR (400 MHz, $CDCl_3$) δ 7.65 (d, *J* = 16.0 Hz, 1H, Ph-C*H*=C-), 7.54 – 7.46 (m, 2H, 2 × Ar*H*), 7.43 – 7.37 (m, 3H, 3 × Ar*H*), 6.93 – 6.84 (m, 1H, Me-C*H*-C(Me)-C=O), 6.35 (d, *J* = 16.0 Hz, 1H, Ph-CH=C*H*-), 5.94 – 5.73 (m, 1H, CH_2=C*H*-CH_2-), 5.33 – 5.22 (m, 2H, H-3-Glc*p*, H-4-Glc*p*), 5.21 – 5.17 (m, 1H, H-4-Fuc*p*), 5.14 – 4.90 (m, 4H, 2 × CH_2=CH-CH_2-), 4.72 (d, *J* = 8.4 Hz, 1H, H-1-Glc*p*), 4.46 (d, *J* = 7.6 Hz, 1H, H-1-Fuc*p*), 4.31 (d, *J* = 2.0 Hz, 1H, O*H*), 4.25 – 4.16 (m, 2H, H-6-Glc*p*), 3.90 – 3.66 (m, 7H, H-2-Glc*p*, H-5-Glc*p*, H-2-Fuc*p*, H-3-Fuc*p*, H-5-Fuc*p*, -CH_2-C*H*-CH_2-, O*H*), 2.38 – 2.27 (m, 4H), 2.21 (s, 3H, CH_3-C=O), 2.07 – 1.98 (m, 2H), 1.81 – 1.72 (m, 6H), 1.61 – 1.52 (m, 4H), 1.43 – 1.22 (m, 12H), 1.20 (d, *J* = 6.4 Hz, 3H, H-6-Fuc*p*), 0.89 (t, *J* = 6.8 Hz, 3H). ^{13}C NMR (100 MHz, $CDCl_3$) δ 173.4, 171.3, 168.2, 165.4, 146.4, 139.3, 139.0, 134.3, 133.9, 130.7, 128.9, 128.3, 127.7, 117.5, 116.5, 114.2, 103.3, 99.7, 78.8, 78.7, 74.7, 72.7, 72.2, 71.9, 71.5, 69.2, 68.1, 62.2, 38.1, 34.2, 33.9, 33.7, 31.7, 28.9, 28.7, 28.7, 24.7, 24.6, 22.6, 20.9, 16.3, 14.5, 14.1, 12.0.

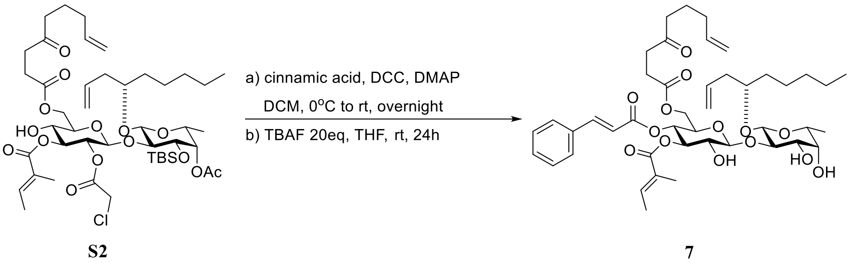

Analogue 7: DCC (11.7 mg, 0.057 mmol) was added in one portion to a 0°C CH_2Cl_2 (1 mL) solution of **S3** (26 mg, 0.028 mmol), cinnamic acid (8.4 mg, 0.057 mmol) and 4-dimethylaminopyridine (1.7 mg, 0.014 mmol). The reaction was allowed to warm to ambient temperature and stirred overnight. At this point, TLC (silica, 1:4 EtOAc–hexanes) showed the reaction was complete. The reaction mixture was diluted with ether (2 mL) and hexanes (1 mL), stirred for 20 minutes then filtered thru a pad of Celite using ether (2 mL) as the eluent and the filtrate was concentrated *in vacuo*. To a solution of the obtained product in THF (3 mL) was added TBAF (1M solution in THF, 0.50 mL, 0.50 mmol, 20 equiv) at rt. The reaction mixture was stirred for 24 h at which point TLC (silica, 1:1 EtOAc–hexanes) showed it was complete. The reaction mixture was diluted with Et_2O (10 mL), washed with 1M HCl (10 mL), saturated $NaHCO_3$ (10 mL), brine (10 mL). The aqueous layer was extracted with Et_2O (10 mL). The combined organic layer was dried over Na_2SO_4 and concentrated under reduced pressure. The residue was purified by column chromatography (silica,

EtOAc–hexanes, 1:1→2:1) gave compound **7** (12.2 mg, 53% over two steps) as a colorless syrup. $[\alpha]_D^{25}$ –33° (*c* 0.1 $CHCl_3$). ^1H NMR (400 MHz, $CDCl_3$) δ 7.65 (d, *J* = 16.0 Hz, 1H, Ph-C*H*=CH-), 7.58 – 7.49 (m, 2H, 2 × Ar*H*), 7.46 – 7.36 (m, 3H, 3 × Ar*H*), 6.92 – 6.81 (m, 1H, Me-C*H*-C(Me)-C=O), 6.34 (d, *J* = 16.0 Hz, 1H, Ph-CH=C*H*-), 5.94 – 5.68 (m, 2H, CH_2=C*H*-CH_2-), 5.31 (t, *J* = 9.6 Hz, 1H, H-4-Glc*p*), 5.22 (t, *J* = 9.6 Hz, 1H, H-3-Glc*p*), 5.16 – 4.94 (m, 4H, 2 × CH_2=CH-CH_2-), 4.74 (d, *J* = 8.4 Hz, 1H, H-1-Glc*p*), 4.45 (d, *J* = 8.0 Hz, 1H, H-1-Fuc*p*), 4.38 (dd, *J* = 12.4, 2.4 Hz, 1H, H-6-Glc*p*), 4.20 (d, *J* = 2.4 Hz, 1H, O*H*), 4.13 (dd, *J* = 12.4, 5.2 Hz, 1H, H-6-Glc*p*), 3.98 – 3.58 (m, 8H, H-2-Glc*p*, H-5-Glc*p*, H-2-Fuc*p*, H-3-Fuc*p*, H-4-Fuc*p*, H-5-Fuc*p*, -CH_2-C*H*-CH_2-, O*H*), 2.90 (d, *J* = 2.0 Hz, 1H, O*H*), 2.82 – 2.55 (m, 4H), 2.47 (t, *J* = 7.4 Hz, 2H), 2.37 – 2.24 (m, 2H), 2.14 – 2.00 (m, 2H), 1.82 – 1.65 (m, 8H), 1.58 – 1.49 (m, 2H), 1.43 – 1.20 (m, 9H), 0.87 (t, *J* = 6.8 Hz, 3H). ^{13}C NMR (100 MHz, $CDCl_3$) δ 208.8, 172.4, 167.7, 165.4, 146.5, 138.8, 137.9, 134.0, 133.9, 130.7, 128.9(×2), 128.3(×2), 127.8, 117.9, 116.5, 115.2, 101.8, 98.8, 77.7, 75.8, 73.8, 72.9, 71.0, 70.4, 69.6, 68.1, 62.1, 41.8, 37.7, 37.0, 34.2, 33.0, 31.7, 27.8, 24.7, 22.7, 22.5, 16.3, 14.5, 14.1, 12.0.

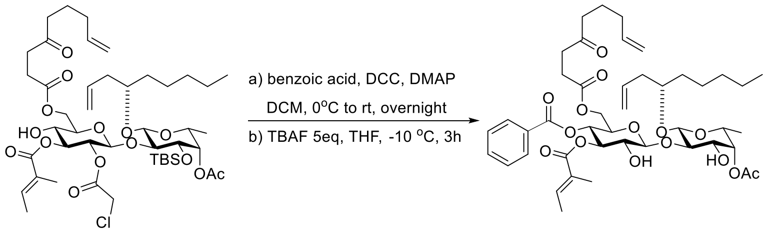

S3 **8**

Analogue 8: Benzoic acid (11.2 mg, 0,092 mmol) was added in one portion to a solution of **S3** (42 mg, 0.046 mmol), DCC (18.9 mg, 0.092 mmol) and 4-dimethylaminopyridine (2.8 mg, 0.023 mmol) in CH_2Cl_2 (2 mL). The reaction was stirred at room temperature for 12 h. At this point, TLC (silica, 1:3 EtOAc–hexanes) showed the reaction was complete. The reaction mixture was diluted with ether (2 ml) and hexanes (1 ml), stirred for 20 minutes then filtered thru a pad of celite using ether (3 ml) as the eluent and the filtrate concentrated *in vacuo*. To a solution of the obtained crude product in THF (3 mL) was added TBAF (1M solution in THF, 0.22 mL, 0.22 mmol, 5 equiv) at –10 °C. The reaction mixture was stirred at the same temperature for 3 h at which point TLC (silica, 1:1 EtOAc–hexanes) showed it was complete. The reaction mixture was diluted with Et_2O (20 mL), washed with 1M HCl (10 mL), saturated $NaHCO_3$ (10 mL), brine (10 mL). The aqueous layer was

extracted with Et_2O (20 mL). The combined organic layer was dried over Na_2SO_4 and concentrated under reduced pressure. The residue was purified by column chromatography (silica, EtOAc–hexanes, 1:1) gave compound **8** (21.1 mg, 56% over two steps) as a colorless syrup. $[\alpha]_D^{25}$ –29.1° (*c* 0.5 $CHCl_3$). ^{1}H NMR (400 MHz, $CDCl_3$) δ 7.94 (d, *J* = 7.2 Hz, 1H, Ar-*H*), 7.59 – 7.53 (m, 1H, Ar-*H*), 7.46 – 7.38 (m, 2H, Ar-*H*), 6.89 – 6.77 (m, 1H, Me-C*H*-C(Me)-C=O), 5.93 – 5.69 (m, 2H, CH_2=C*H*-CH_2-), 5.40 – 5.31 (m, 2H, H-3-Glc*p*, H-4-Glc*p*), 5.20 (d, *J* = 3.6 Hz, 1H, H-4-Fuc*p*), 5.14 – 4.93 (m, 4H, 2 × CH_2=CH-CH_2-), 4.75 (d, *J* = 8.4 Hz, 1H, H-1-Glc*p*), 4.47 (d, *J* = 6.4 Hz, 1H, H-1-Fuc*p*), 4.27 – 4.16 (m, 3H, H-6-Glc*p*, O*H*), 3.95 – 3.69 (m, 6H, H-2-Glc*p*, H-5-Glc*p*, H-2-Fuc*p*, H-3-Fuc*p*, H-5-Fuc*p*, -CH_2-C*H*-CH_2-), 3.66 (d, *J* = 1.6 Hz, 1H, O*H*), 2.71 – 2.63 (m, 2H), 2.58 – 2.52 (m, 2H), 2.44 (t, *J* = 7.4 Hz, 2H), 2.38 – 2.29 (m, 2H), 2.21 (s, 3H, CH_3-C=O), 2.08 – 2.00 (m, 2H), 1.75 – 1.63 (m, 8H), 1.57 – 1.50 (m, 2H), 1.43 – 1.23 (m, 6H), 1.20 (d, *J* = 6.4 Hz, 3H, H-6-Fuc*p*), 0.88 (t, *J* = 6.8 Hz, 3H). ^{13}C NMR (100 MHz, $CDCl_3$) δ 208.5, 172.4, 171.3, 168.0, 165.3, 139.1, 138.0, 134.2, 133.5, 129.8, 128.9, 128.5, 127.6, 117.6, 115.2, 103.0, 99.6, 78.6, 78.2, 74.3, 72.6, 72.2, 71.8, 71.2, 69.2, 68.8, 62.5, 41.7, 38.0, 37.0, 34.2, 33.0, 31.7, 27.7, 24.7, 22.7, 22.5, 20.9, 16.2, 14.4, 14.1, 11.9.

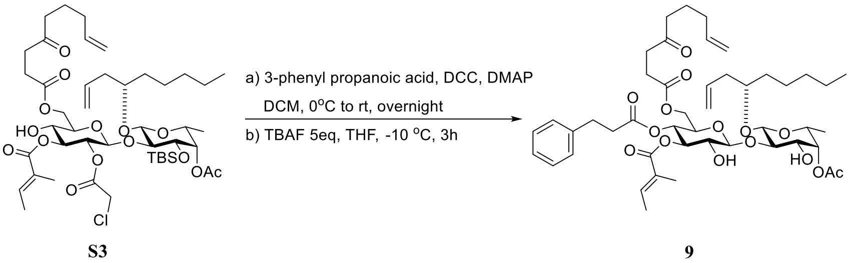

Analogue 9: DCC (12.3 mg, 0.060 mmol) was added in one portion to a 0ºC CH_2Cl_2 (1 mL) solution of **S3** (35.2 mg, 0.040 mmol), 3-phenyl propanoic acid (9.0 mg, 0.060 mmol) and 4-dimethylaminopyridine (0.5 mg, 0.004 mmol). The reaction was allowed to warm to ambient temperature and stirred overnight. At this point, TLC (silica, 1:4 EtOAc–hexanes) showed the reaction was complete. The reaction mixture was diluted with ether (2 mL) and hexanes (1 mL), stirred for 20 minutes then filtered thru a pad of Celite using ether (2 mL) as the eluent and the filtrate was concentrated *in vacuo*. To a solution of the obtained crude product in THF (2 mL) was added TBAF (1M solution in THF, 0.16 mL, 0.16 mmol, 5 equiv) at –10 ºC. The reaction mixture was stirred at the same temperature for 3 h at which point TLC (silica, 1:1 EtOAc–hexanes) showed it was complete. The reaction mixture was diluted with Et_2O (10

mL), washed with 1M HCl (10 mL), saturated $NaHCO_3$ (10 mL), brine (10 mL). The aqueous layer was extracted with Et_2O (10 mL). The combined organic layer was dried over Na_2SO_4 and concentrated under reduced pressure. The residue was purified by column chromatography (silica, EtOAc–hexanes, 1:2→1:1) gave compound **9** (23 mg, 70% over two steps) as a colorless syrup. $[\alpha]_D^{25}$ –16.1° (*c* 1 $CHCl_3$). ^{1}H NMR (400 MHz, $CDCl_3$) δ 7.31 – 7.24 (m, 2H, 2 × Ar*H*), 7.22 – 7.09 (m, 3H, 3 × Ar*H*), 6.92 – 6.84 (m, 1H, Me-C*H*-C(Me)-C=O), 5.91 – 5.70 (m, 2H, CH_2=C*H*-CH_2-), 5.21 – 4.95 (m, 7H, H-3-Glc*p*, H-4-Glc*p*, H-4-Fuc*p*, 2 × CH_2=CH-CH_2-), 4.66 (d, *J* = 8.0 Hz, 1H, H-1-Glc*p*), 4.44 (d, *J* = 7.2 Hz, 1H, H-1-Fuc*p*), 4.25 (d, *J* = 2.4 Hz, 1H, O*H*), 4.12 – 4.07 (m, 2H, H-6-Glc*p*), 3.82 – 3.65 (m, 6H, H-2-Glc*p*, H-5-Glc*p*, H-2-Fuc*p*, H-3-Fuc*p*, H-5-Fuc*p*, -CH_2-C*H*-CH_2-), 3.63 (d, *J* = 2.0 Hz, 1H, O*H*), 2.87 – 2.82 (m, 2H), 2.71 – 2.68 (m, 4H), 2.62 – 2.56 (m, 2H), 2.46 (t, *J* = 7.4 Hz, 2H), 2.34 – 2.28 (m, 2H), 2.19 (s, 3H, CH_3-C=O), 2.10 – 2.02 (m, 2H), 1.79 – 1.74 (m, 6H), 1.71 – 1.63 (m, 2H), 1.58 – 1.48 (m, 2H), 1.41 – 1.22 (m, 6H), 1.19 (d, *J* = 6.4 Hz, 3H, H-6-Fuc*p*), 0.88 (t, *J* = 6.8 Hz, 3H). ^{13}C NMR (100 MHz, $CDCl_3$) δ 208.5, 172.4, 171.5, 171.3, 167.9, 139.9, 139.2, 137.9, 134.2, 128.5(×2), 128.1(×2), 127.7, 126.3, 117.5, 115.2, 102.9, 99.6, 78.6, 78.3, 74.4, 72.5, 72.2, 71.8, 71.2, 69.2, 67.9, 62.0, 41.8, 38.0, 37.0, 35.4, 34.2, 33.0, 31.7, 30.6, 27.7, 24.6, 22.7, 22.5, 20.9, 16.2, 14.5, 14.1, 12.0.

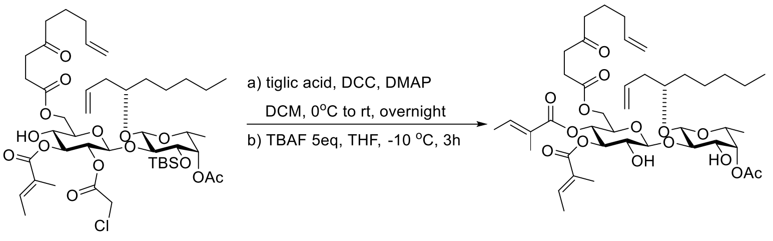

S3 **10**

Analogue 10: DCC (11.6 mg, 0.056 mmol) was added in one portion to a 0°C CH_2Cl_2 (1 mL) solution of **S3** (34.5 mg, 0.038 mmol), tiglic acid (5.6 mg, 0.056 mmol) and 4-dimethylaminopyridine (0.5 mg, 0.004 mmol). The reaction was allowed to warm to ambient temperature and stirred overnight. At this point, TLC (silica, 1:4 EtOAc–hexanes) showed the reaction was complete. The reaction mixture was diluted with ether (2 mL) and hexanes (1 mL), stirred for 20 minutes then filtered thru a pad of Celite using ether (2 mL) as the eluent and the filtrate was concentrated *in vacuo*. To a solution of the obtained crude product in THF (2 mL) was added TBAF (1M solution in THF, 0.16 mL, 0.16 mmol, 5 equiv) at –10 °C. The reaction mixture

was stirred at the same temperature for 3 h at which point TLC (silica, 1:1 EtOAc–hexanes) showed it was complete. The reaction mixture was diluted with Et_2O (10 mL), washed with 1M HCl (10 mL), saturated $NaHCO_3$ (10 mL), brine (10 mL). The aqueous layer was extracted with Et_2O (10 mL). The combined organic layer was dried over Na_2SO_4 and concentrated under reduced pressure. The residue was purified by column chromatography (silica, EtOAc–hexanes, 1:2→1:1) gave analogue **10** (19 mg, 63% over 2 steps) as a colorless syrup. $[\alpha]_D^{25}$ –22.5° (*c* 1 $CHCl_3$). ^1H NMR (400 MHz, $CDCl_3$) δ 6.92 – 6.83 (m, 2H, 2 × Me-C*H*-C(Me)-C=O), 5.94 – 5.70 (m, 2H, 2 × CH_2=C*H*-CH_2-), 5.29 – 4.94 (m, 7H, H-3-Glc*p*, H-4-Glc*p*, H-4-Fuc*p*, 2 × CH_2=CH-CH_2-), 4.69 (d, *J* = 8.0 Hz, 1H, H-1-Glc*p*), 4.45 (d, *J* = 7.2 Hz, 1H, H-1-Fuc*p*), 4.26 – 4.09 (m, 3H, H-6-Glc*p*, O*H*), 3.85 – 3.66 (m, 7H, H-2-Glc*p*, H-5-Glc*p*, H-2-Fuc*p*, H-3-Fuc*p*, H-5-Fuc*p*, -CH_2-C*H*-CH_2-, O*H*), 2.77 – 2.67 (m, 2H), 2.62 – 2.55 (m, 2H), 2.46 (t, *J* = 7.4 Hz, 2H), 2.35 – 2.26 (m, 2H), 2.20 (s, 3H, CH_3-C=O), 2.10 – 2.01 (m, 2H), 1.80 – 1.61 (m, 14H), 1.59 – 1.48 (m, 2H), 1.41 – 1.21 (m, 6H), 1.19 (d, *J* = 6.4 Hz, 3H, H-6-Fuc*p*), 0.88 (t, *J* = 7.0 Hz, 3H). ^{13}C NMR (100 MHz, $CDCl_3$) δ 208.6, 172.5, 171.3, 168.0, 166.5, 139.1, 138.9, 138.0, 134.2, 127.7, 127.5, 117.6, 115.2, 102.8, 99.5, 78.5, 78.0, 74.4, 72.7, 72.1, 71.8, 71.1, 69.2, 68.0, 62.4, 41.8, 38.0, 37.0, 34.2, 33.0, 31.7, 27.7, 24.7, 22.7, 22.5, 20.9, 16.2, 14.5, 14.5, 14.1, 12.0, 11.9.

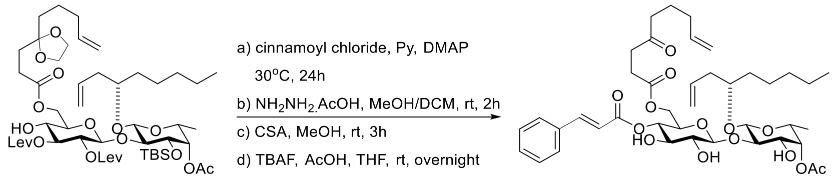

S6 11

Analogue 11: To a cold (0 ºC) solution of compound **S6** [4] (55.0 mg, 0.055 mmol) and DMAP (13.4 mg, 0.11 mmol) in pyridine (4 mL) was added cinnamoyl chloride (36.7 mg, 0.22 mmol). The reaction mixture was heated to 30 ºC and stirred for a further 24 h, at the end of which time TLC (silica, 1:3 EtOAc–hexanes) indicated that the reaction was complete. The reaction was quenched with MeOH (20 µL) and diluted with CH_2Cl_2 (30 mL), washed with 1 M HCl (20 mL), sat. aq. $NaHCO_3$ (20 mL), dried (Na_2SO_4) and concentrated under reduced pressure. Hydrazine acetate (40 mg, 0.43 mmol) was added to a solution of the obtained crude product in 2:1 DCM/MeOH (3 mL) at room temperature. The reaction mixture was stirred for 2 hours, at which point TLC (silica, 1:2 EtOAc–hexanes) showed the reaction was

complete. Then it was quenched with aqueous $NaHCO_3$ (20 mL) and extracted with DCM (20 mL × 2). The combined organic extracts were dried over Na_2SO_4. Evaporation and purification by column chromatography (silica, EtOAc–hexanes, 1:3 → 1:2) to afford the Lev removed intermediate. To a solution of the obtained intermediate in MeOH (2 mL) was added CSA (2.0 mg) at room temperature. The reaction mixture was stirred for 3 hours then water (10 μL) was added. The resulting mixture was stirred for another 2 hours. The reaction was quenched with Et_3N (10 μL) and concentrated. To a solution of the obtained crude product in THF (2 mL) was added AcOH (170 μL) and TBAF (1M solution in THF, 1.5 mL) at 0 °C. The reaction was allowed to warm to ambient temperature and stirred overnight. At this point, TLC (silica, EtOAc) showed the reaction was complete. The reaction mixture was concentrated under reduced pressure. The residue was purified by column chromatography (silica, EtOAc–hexanes, 2:1 → 3:1) gave compound **11** (16.8 mg, 32% over four steps) as a colorless syrup. $[\alpha]_D^{25}$ 7.9° (*c* 1 $CHCl_3$). ^{1}H NMR (400 MHz, $CDCl_3$) δ 7.75 (d, *J* = 16.0 Hz, 1H, Ph-C*H*=CH-), 7.58 – 7.51 (m, 2H, 2 × Ar*H*), 7.44 – 7.35 (m, 3H, 3 × Ar*H*), 6.45 (d, *J* = 16.0 Hz, 1H, Ph-CH=C*H*-), 5.93 – 5.69 (m, 2H, CH_2=C*H*-CH_2-), 5.24 – 5.18 (m, 1H, H-4-Fuc*p*), 5.17 – 4.93 (m, 5H, H-4-Glc*p*, 2 × CH_2=CH-CH_2-), 4.62 (d, *J* = 8.0 Hz, 1H, H-1-Glc*p*), 4.46 (d, *J* = 7.2 Hz, 1H, H-1-Fuc*p*), 4.42 (d, *J* = 2.0 Hz, 1H, O*H*), 4.28 (dd, *J* = 12.4, 2.8 Hz, 1H, H-6-Glc*p*), 4.15 (dd, *J* = 12.4, 6.0 Hz, 1H, H-6-Glc*p*), 3.88 – 3.63 (m, 8H, H-2-Glc*p*, H-3-Glc*p*, H-5-Glc*p*, H-2-Fuc*p*, H-3-Fuc*p*, H-5-Fuc*p*, -CH_2-C*H*-CH_2-, O*H*), 2.93 (d, *J* = 2.8 Hz, 1H, O*H*), 2.75 – 2.68 (m, 2H), 2.61 – 2.53 (m, 2H), 2.43 (t, *J* = 7.6 Hz, 2H), 2.37 – 2.30 (m, 2H), 2.20 (s, 3H, CH_3-C=O), 2.08 – 2.00 (m, 2H), 1.71 – 1.64 (m, 2H), 1.58 – 1.50 (m, 2H), 1.43 – 1.23 (m, 6H), 1.19 (d, *J* = 6.4 Hz, 3H, H-6-Fuc*p*), 0.87 (t, *J* = 6.8 Hz, 3H). ^{13}C NMR (100 MHz, $CDCl_3$) δ 208.7, 172.5, 171.3, 166.3, 146.7, 138.0, 134.0, 134.0, 130.7, 128.9(×2), 128.3(×2), 117.8, 116.7, 115.2, 101.7, 99.4, 78.4, 74.2, 72.9, 72.0, 71.8, 71.6, 70.3, 69.3, 62.6, 41.8, 37.9, 37.0, 34.2, 33.0, 31.6, 27.7, 24.7, 22.7, 22.6, 20.9, 16.2, 14.0.

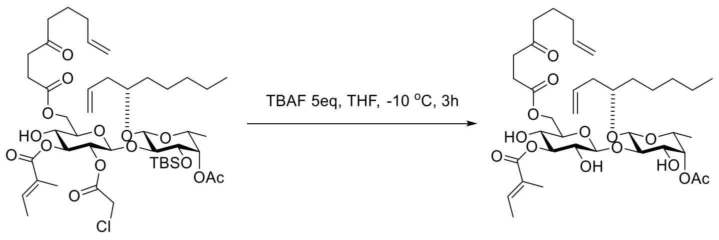

S3 12

Analogue 12: To a solution of **S3** (15.2 mg, 0.030 mmol) in THF (2 mL) was added TBAF (1M solution in THF, 83 µL, 83 µmol, 5 equiv) at –10 °C. The reaction mixture was stirred at the same temperature for 3 h at which point TLC (silica, 1:1 EtOAc–hexanes) showed it was complete. The reaction mixture was diluted with Et_2O (20 mL), washed with 1M HCl (10 mL), saturated $NaHCO_3$ (10 mL), brine (10 mL). The aqueous layer was extracted with Et_2O (20 mL). The combined organic layer was dried over Na_2SO_4 and concentrated under reduced pressure. The residue was purified by column chromatography (silica, EtOAc–hexanes, 1:1) gave compound **12** (7.5 mg, 62%) as a white film. $[\alpha]_D^{25}$ –2.0° (*c* 0.2 $CHCl_3$). ^{1}H NMR (400 MHz, $CDCl_3$) δ 7.09 – 6.97 (m, 1H, Me-C*H*-C(Me)-C=O), 5.93 – 5.70 (m, 2H, CH_2=C*H*-CH_2-), 5.18 (d, *J* = 3.2 Hz, 1H, H-4-Fuc*p*), 5.14 – 4.97 (m, 4H, 2 × CH_2=CH-CH_2-), 4.93 (t, *J* = 9.0 Hz, 1H, H-3-Glc*p*), 4.64 (d, *J* = 8.4 Hz, 1H, H-1-Glc*p*), 4.53 – 4.42 (m, 2H, H-6-Glc*p*, H-1-Fuc*p*), 4.37 – 4.30 (m, 1H, H-6-Glc*p*), 4.21 (d, *J* = 2.0 Hz, 1H, O*H*), 3.85 – 3.57 (m, 8H, H-2-Glc*p*, H-3-Glc*p*, H-4-Glc*p*, H-5-Glc*p*, H-2-Fuc*p*, H-3-Fuc*p*, H-5-Fuc*p*, -CH_2-C*H*-CH_2-), 3.35 (d, *J* = 3.6 Hz, 1H, O*H*), 2.81 – 2.53 (m, 4H), 2.47 (t, *J* = 7.4 Hz, 2H), 2.34 (t, *J* = 7.2 Hz, 2H), 2.17 (s, 3H, CH_3-C=O), 2.12 – 2.02 (m, 2H), 1.90 – 1.81 (m, 6H), 1.75 – 1.64 (m, 2H), 1.59 – 1.51 (m, 2H), 1.43 – 1.22 (m, 6H), 1.19 (d, *J* = 6.4 Hz, 3H, H-6-Fuc*p*), 0.89 (t, *J* = 6.8 Hz, 3H). ^{13}C NMR (100 MHz, $CDCl_3$) δ 208.9, 173.2, 171.2, 169.7, 139.5, 137.9, 134.1, 127.9, 117.6, 115.3, 102.8, 99.6, 78.5, 77.9, 77.8, 75.0, 72.1, 71.8, 70.7, 69.2, 68.9, 62.8, 41.8, 37.8, 37.1, 34.1, 33.0, 31.7, 27.7, 24.6, 22.7, 22.5, 20.9, 16.2, 14.6, 14.1, 12.0.

Part II: Synthesis of the rationally designed open-chain analogue 3

General methods: All reaction glassware were thoroughly washed, and oven dried before any reactions were undertaken. Unless otherwise stated, all reagents obtained commercially were used without any other extra purifications. Also, all reactions were conducted under argon atmosphere unless stated otherwise. Monitoring the progress of the reactions was done by TLC using silica gel MF254 glass back plates with detection by either visualizing under UV lamb (254 nm) or charring with 5 % (v/v) H_2SO_4 (sulfuric acid) in EtOH (ethanol). Column chromatographic purifications were done on silica gel (70 – 230 mesh) with a ratio that spanned from 100 to 50: 1 (w/w) between the silica gel and crude products. All ^{1}H NMR spectra were obtained in deuterated chloroform ($CDCl_3$) with chloroform ($CHCl_3$, δ = 7.27) as internal reference for ^{1}H, in deuterated methanol (CD_3OD) with methanol (CH_3OH, δ = 3.31) as internal reference for ^{1}H, and in deuterated dimethyl sulfoxide ($(CD_3)_2SO$) with dimethyl sulfoxide ($(CH_3)_2SO$, δ = 2.50) as internal reference for ^{1}H. All ^{13}C NMR spectra were proton decoupled and were performed in $CDCl_3$ with $CHCl_3$ (δ = 77.0 for ^{13}C), in CD_3OD with CH_3OH (δ = 49.9 for ^{13}C), and in $(CD_3)_2SO$ with $(CH_3)_2SO$ (δ = 40.4 for ^{13}C) as internal reference. NMR data are reported in the form: chemical shifts (δ) in ppm, multiplicity, coupling constants (J) in Hz, and integrations. ^{1}H data are reported as though they were first order. An error less than 0.5 Hz are reported for coupling constants between two coupled protons. Other 1D and 2D NMR spectra like ^{135}DEPT, COSY, HMQC, and HMBC were collected in addition to ^{1}H and ^{13}C in the characterization of new compounds.

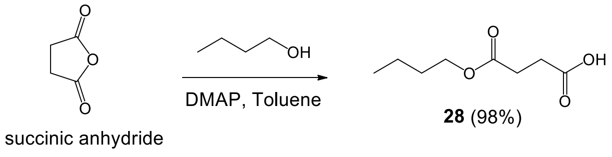

4-Butoxy-4-oxobutanoic acid (mono-butyl succinate) 28: Succinic anhydride (496.6 mg, 4.96 mmol), butanol (0.50 mL, 5.46 mmol, 1.1 eq.) and DMAP (61 mg, 0.49 mmol, 0.1 eq.) were dissolved in Toluene (7 mL). The solution was heated at reflux overnight. After evaporation, the residue was purified by column chromatography (8:1→4:1, hexanes–EtOAc containing 0.5% AcOH) to afford **28** (846.3 mg, 98%) as a colorless thin oil: R_f 0.41 (4:1, hexanes–EtOAc, 0.5% AcOH); ^{1}H NMR (400 MHz, $CDCl_3$, δ_H) 4.11 (t, J = 6.7 Hz, 2H, OCH_2), 2.59–2.71 (m, 4H, $COCH_2CH_2CO$), 1.56–1.66 (m, 2H, CH_2), 1.32–1.43 (m, 2H, CH_2), 0.93 (t, J = 7.4

Hz, 3H, CH_3); ^{13}C NMR (100 MHz, $CDCl_3$, δ_C) 178.2 (C=O), 172.4 (C=O), 64.9 (OCH_2), 30.6 (CH_2), 29.1 (CH_2), 29.0 (CH_2), 19.2 (CH_2), 13.8 (CH_3).

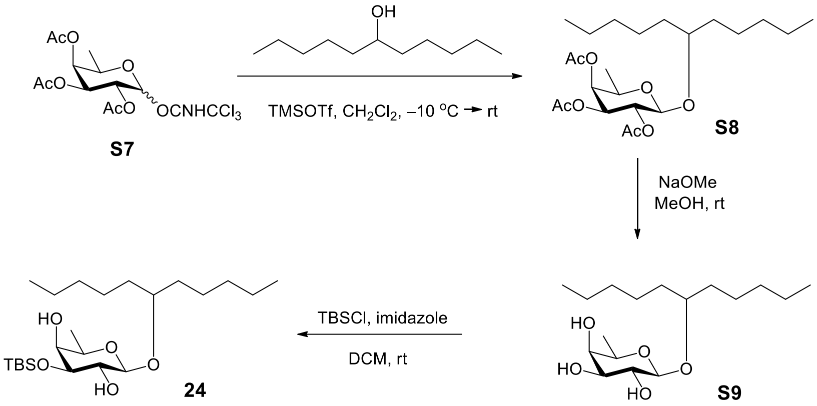

Compound S8: The known fucoside trichloroacetimidate donor **S7** [1] (609.5 mg, 1.40 mmol), crushed activated 4Å molecular sieves (700 mg) and 6-undecanol (253.3 mg, 1.47 mmol, 1.05 eq.) were suspended in anhydrous CH_2Cl_2 (5 mL). The mixture was stirred under an argon atmosphere for ~30 min at room temperature and then cooled to -10 °C. TMSOTf (25.4 μL, 0.14 mmol, 0.1 eq.) was added dropwise via syringe. After adding TMSOTf, the reaction mixture was gradually warmed to 0 °C. Once the starting material **S7** was fully consumed (usually within 1.5–2 h), the reaction mixture was quenched by the addition of Et_3N and filtered through a pad of celite. The filtrate was then concentrated and the resulting residue was purified by column chromatography (15:1→6:1 hexanes–EtOAc) to acquire pure **S8** as a colorless oil (501.0 mg, 80%): R_f 0.49 (4:1 hexanes–EtOAc); ^{1}H NMR (400 MHz, $CDCl_3$, δ_H) 5.21 (br d, $J_{3,4}$ = 3.4 Hz, 1H, H-4), 5.16 (dd, $J_{2,3}$ = 10.4 Hz, $J_{1,2}$ = 7.9 Hz, 1H, H-2), 5.01 (dd, $J_{2,3}$ = 10.4 Hz, $J_{3,4}$ = 3.4 Hz, 1H, H-3), 4.45 (d, $J_{1,2}$ = 7.9 Hz, 1H, H-1), 3.77 (br q, $J_{5,6}$ = 6.4 Hz, 1H, H-5), 3.54 (quint, J = 5.5 Hz, 1H, OCH), 2.17 (s, 3H, $COCH_3$), 2.03 (s, 3H, $COCH_3$), 1.98 (s, 3H, $COCH_3$), 1.16–1.65 (m, 19H), 0.84–0.93 (m, 6H); ^{13}C NMR (100 MHz, $CDCl_3$, δ_C) 170.9 (C=O), 170.4 (C=O), 169.4 (C=O), 100.9 (C-1), 81.3 (OCH), 71.6 (OCH-Fuc*p*), 70.4 (OCH-Fuc*p*), 69.4 (OCH-Fuc*p*), 68.9 (OCH-Fuc*p*), 34.8 (CH_2), 34.0 (CH_2), 32.1 (CH_2), 31.9 (CH_2), 24.8(0) (CH_2), 24.7(5) (CH_2), 22.7 (CH_2), 22.6 (CH_2), 20.9 ($COCH_3$), 20.8 ($COCH_3$), 20.7 ($COCH_3$), 16.2 (CH_3, C-6-Fuc*p*), 14.1 (2, CH_3).

Compound S9: Compound **S8** (501 mg, 0.14 mmol) was dissolved in CH_3OH (10 mL). A catalytic amount of sodium methoxide was added, and then the solution was stirred overnight. Next, the reaction was quenched by acetic acid. The solvent was

evaporated and the residue was purified by column chromatography (40:1→10:1, DCM–MeOH) to acquire pure **S9** (340.3 mg, 95%) as a colorless syrup: R_f 0.27 (10:1 DCM–MeOH); ^{1}H NMR (400 MHz, $CDCl_3$, δ_H) 4.74 (br s, 1H, OH), 4.16 (d, $J_{1,2}$ = 7.6 Hz, 1H, H-1), 4.08 (br s, 1H, OH), 3.89 (br s, 1H, OH), 3.45–3.70 (m, 5H), 1.10–1.60 (m, 19H), 0.76–0.92 (m, 6H); ^{13}C NMR (100 MHz, $CDCl_3$, δ_C) 102.7 (C-1), 80.2 (OCH), 74.3 (OCH), 71.8 (OCH), 71.1 (OCH), 70.5 (OCH), 34.8 (CH_2), 34.1 (CH_2), 32.1 (CH_2), 32.0 (CH_2), 25.0 (2, CH_2), 22.7 (2, CH_2), 16.5 (CH_3, C-6-Fuc*p*), 14.2 (2, CH_3).

Compound 24: To an ice-cold solution of **S9** (340.3 mg, 1.07 mmol) and 1H-imidazole (218.3 mg, 3.21 mmol, 3.0 eq.) in CH_2Cl_2 (5 mL) was added TBSCl (*t*-butyldimethylsilyl chloride) (289.9 mg, 1.92 mmol, 1.8 eq.). The reaction was stirred at 0 °C for at least 30 min and then was gradually warmed to ambient temperature overnight. At this point, TLC (silica, 4:1 hexanes-EtOAc) confirmed complete conversion of **S9**. The reaction mixture was then washed with brine (15 mL). The aqueous layer was back extracted with CH_2Cl_2 (10 mL). The combined organic layer was dried over Na_2SO_4, filtered and concentrated. The residue was purified by column chromatography (30:1→20:1 hexanes–EtOAc) to give the diol acceptor **24** (353.0 mg, 76%) as a colorless oil: R_f 0.50 (10:1 hexanes–EtOAc); ^{1}H NMR (400 MHz, $CDCl_3$, δ_H) 4.15 (d, $J_{1,2}$ = 7.8 Hz, 1H, H-1), 3.44–3.63 (m, 5H), 2.58 (br s, 1H, OH), 2.11 (d, J = 2.1 Hz, 1H, OH), 1.14–1.60 (m, 19H), 0.80–0.92 (m, 15H), 0.12 (s, 3H, $SiCH_3$), 0.10 (s, 3H, $SiCH_3$); ^{13}C NMR (100 MHz, $CDCl_3$, δ_C) 102.2 (C-1), 79.6 (OCH), 75.0 (OCH), 72.3 (OCH), 72.1 (OCH), 70.0 (OCH), 34.8 (CH_2), 34.0 (CH_2), 32.1 (CH_2), 32.0 (CH_2), 25.8 ($C(\underline{C}H_3)_3$), 25.0 (CH_2), 24.8 (CH_2), 22.7 (2, CH_2), 18.2 ($\underline{C}(CH_3)_3$), 16.5 (CH_3, C-6-Fuc*p*), 14.2 (CH_3), 14.1 (CH_3), -4.4 ($SiCH_3$), -4.9 ($SiCH_3$).

Supplementary Data 2 Figs

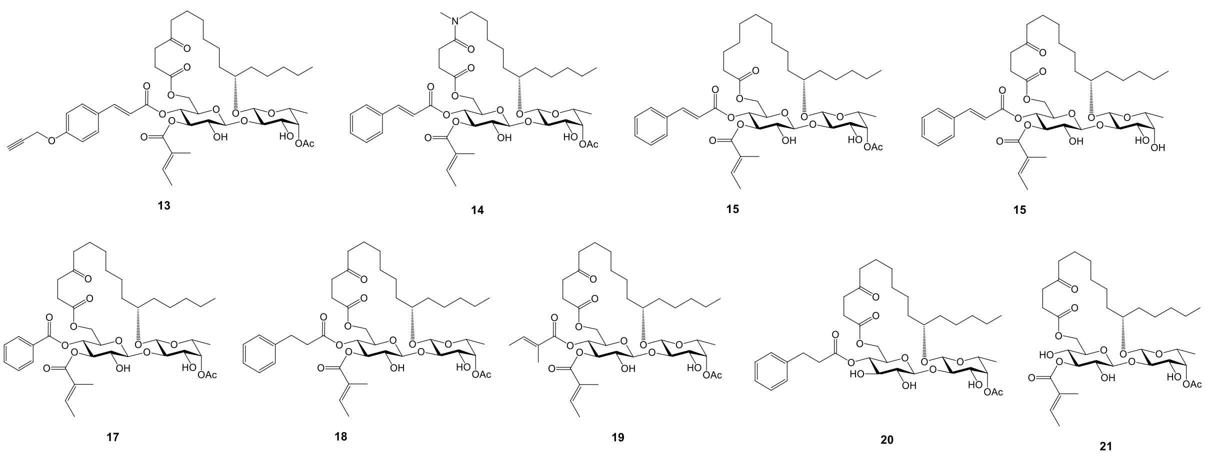

Fig. S1 (related to Fig. 2). Structures of ring-closed analogues **13–21**.

Table S1 (related to Fig. 2). Cytotoxicity of open-chain analogues **4–12** and their corresponding closed-chain analogues **13**–**21** against MDA-MB-231 cells.*

Ring-open	IC_{50} ± SD (nM)	Ring-closed	IC_{50} ± SD (nM)
**1**	59 ± 8.1	Ipom-F	6.5 ± 0.4
**4**	168 ± 19	**13**	6.3 ± 0.9
**5**	43 ± 11	**14**	12 ± 2.9
**6**	205 ± 11	**15**	16 ± 4.2
**7**	3,885 ± 184	**16**	128 ± 11
**8**	4,614 ± 169	**17**	979 ± 170
**9**	2,173 ± 348	**18**	980 ± 40
**10**	2,741 ± 120	**19**	4,500 ± 329
**11**	6,315 ± 341	**20**	6,848 ± 308
**12**	5,604 ± 466	**21**	7,129 ± 292

* IC_{50} for cytotoxicity was derived from at least three independent experiments.

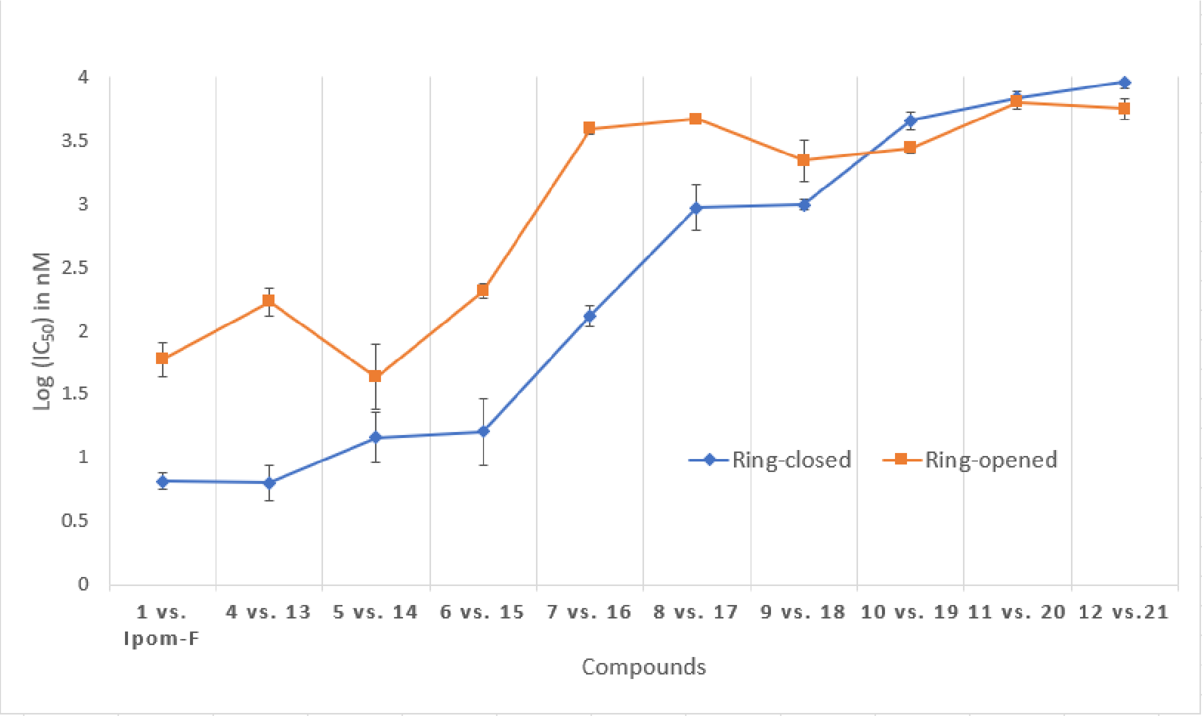

Fig. S2 (related to Fig. 2). Correlation curves between ring-opened analogues **4–12** and ring-closed analogues **13–21**.

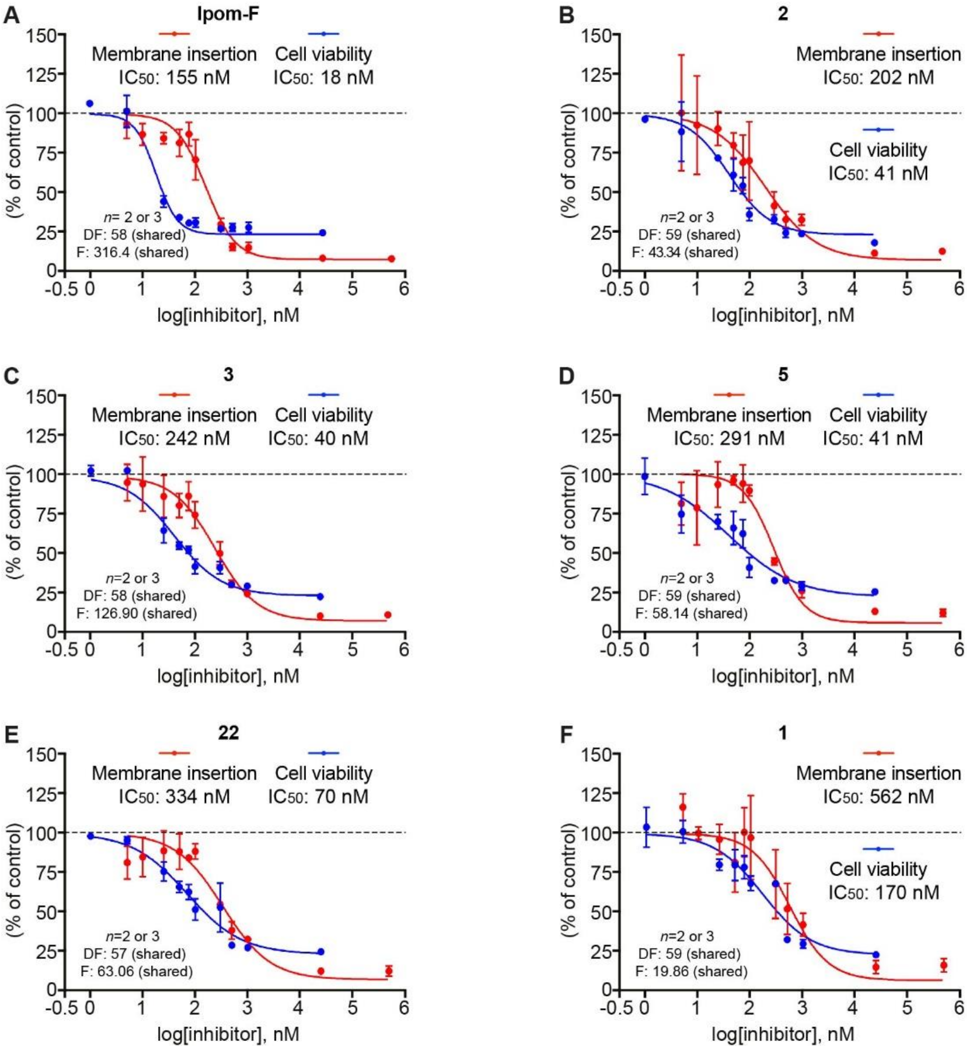

Fig. S3 (related to Figs 3 and 6). Comparison of compound IC_{50} curves derived from *in vitro* and *in cellula* analyses. IC_{50} curves derived from the efficiency of Ii membrane integration in the presence of 500 µM-5 nM concentrations of (A) Ipom-F and analogues (B) **2**, (C) **3**, (D) **5**, (E) **22** and (F) **1** (red curves; presented in Figs 2E and 5C) shown on the same graph as those derived from resazurin-based viability assays using HCT116 Sec61α-WT cells treated with 25 µM-1 nM of the same compound (blue curves; presented in Figs 2E and 5E). In both cases, quantifications normalised to the DMSO control are given as means ± SEM for independent experiments performed in triplicate (Ipom-F, analogues **22** and **3** for 25 µM-25 nM concentrations; analogues **1**, **2** and **5** for 25 µM-5 nM concentrations) or duplicate (Ipom-F, analogues **22** and **3** for 5-1 nM concentrations; analogues **1**, **2** and **5** for 1 nM concentration) (*n* = 2 or 3, biologically independent experiments).

A At the **cytosol** (binding site of Mycolactone) **Plug** **TM2** **CL4** **TM7** **TM3** **CL8** **Others**

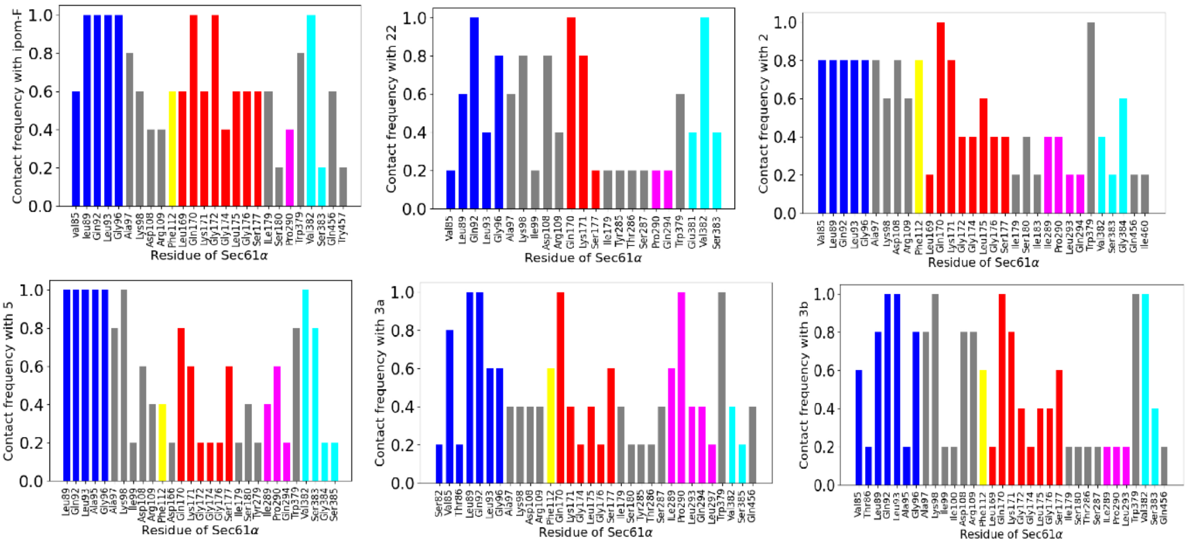

B At the **lateral gate**

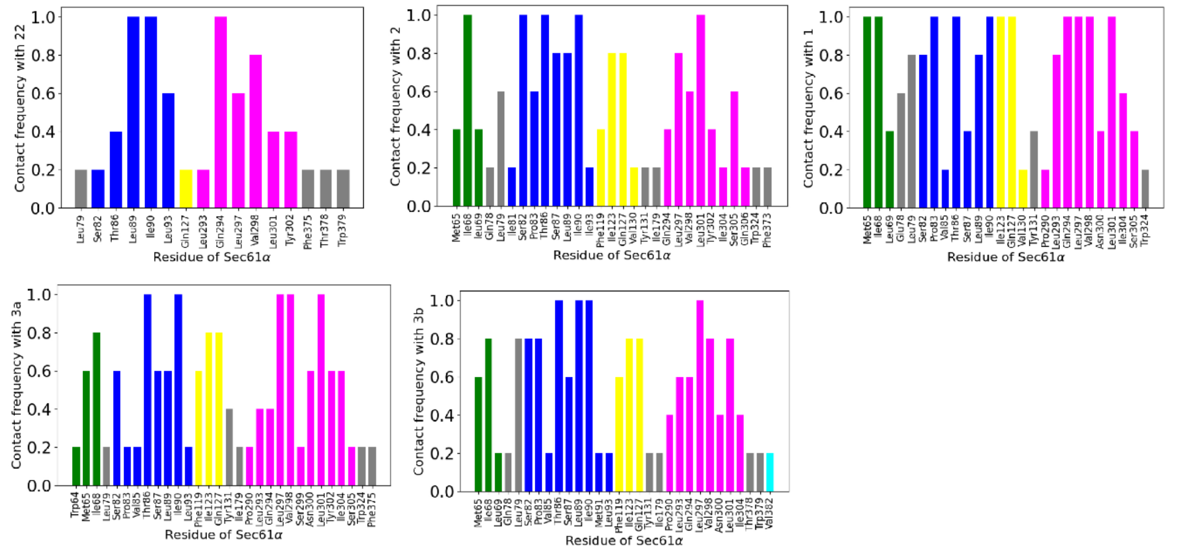

C Brief of the contact region between analogues and Sec61α

	Sec61α						
**Compound**	**TM2 (G80-G96)**	**CL4 (L169-G178)**	**% of contact residue from CL4 region (L168-S180) / likely to form hydrogen bonds with CL4 region**	**CL8 (I380-E406)**	**TM3 (A110-V130)**	**TM7 (I289-R311)**	**Plug (A59-L69)**
**Ipom-F**	**yes**	**yes**	**76.9% / yes**	**yes**	**-**	**-**	**-**
**22**	**yes**	**yes**	**30.7% /-**	**yes**	**-**	**yes**	**-**
**2**	**yes**	**yes**	**76.9% / yes**	**yes**	**yes**	**yes**	**yes**
**5**	**yes**	**yes**	**61.5% / -**	**yes**	**-**	**-**	**-**
**1**	**yes**	**-**	**- / -**	**-**	**-**	**yes**	**-**
**3a**	**yes**	**yes**	**61.5% / -**	**yes**	**yes**	**yes**	**yes**
**3b**	**yes**	**yes**	**76.9% / yes**	**yes**	**yes**	**yes**	**yes**

Fig. S4 (Related to Figs 4 and 5). Contacts between Sec61α, Ipom-F and analogues. (A) Compounds binding in the groove of the mycolactone binding site. (B) compounds binding in the lateral gate. (C) Brief characterization of the binding interface between Sec61α and modelled compounds.

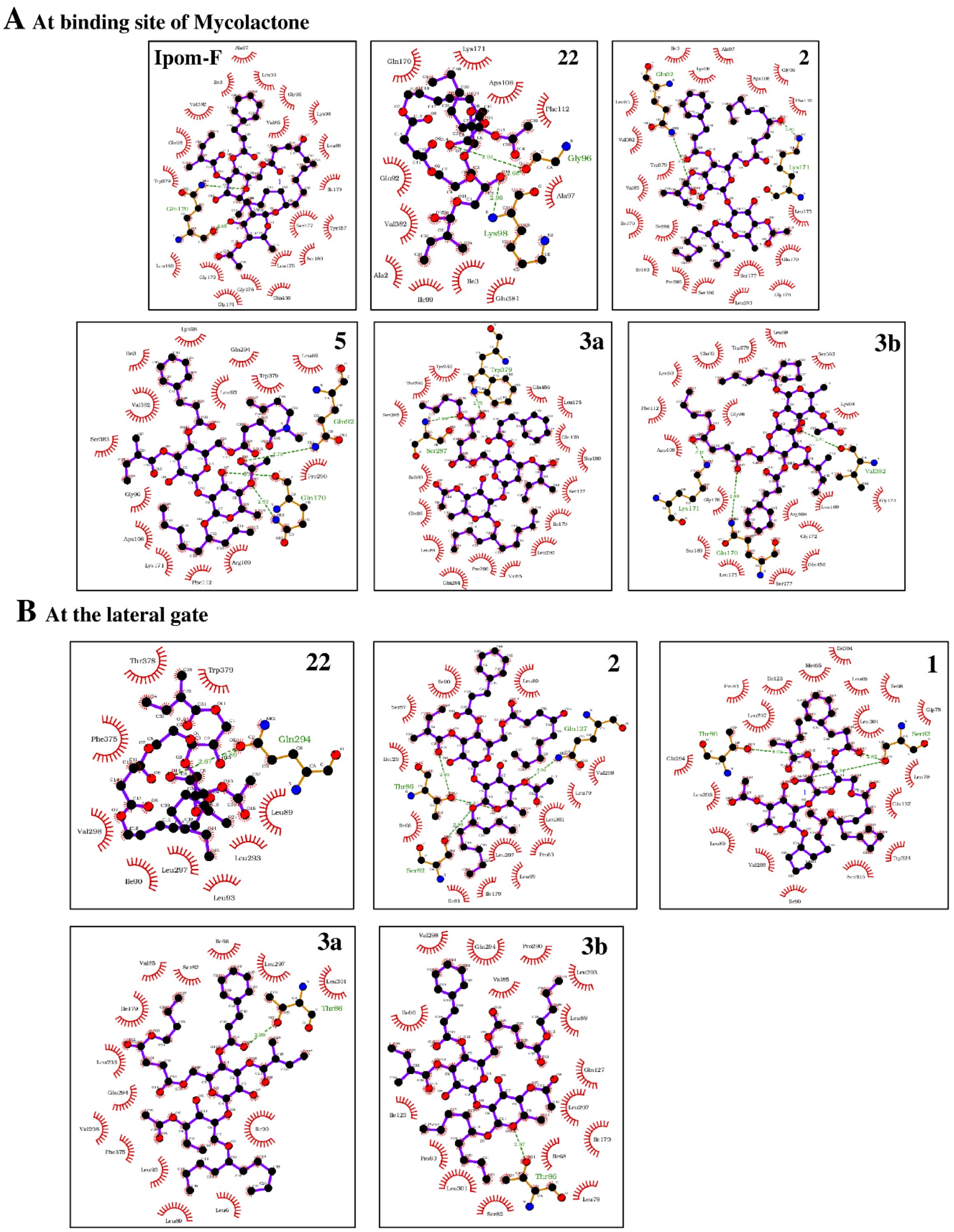

Fig. S5 (Related to Figs 4 and 5). The polar and non-polar contacts between the docked Ipom-F derivatives and Sec61α residues in the energetically most favorable binding poses.

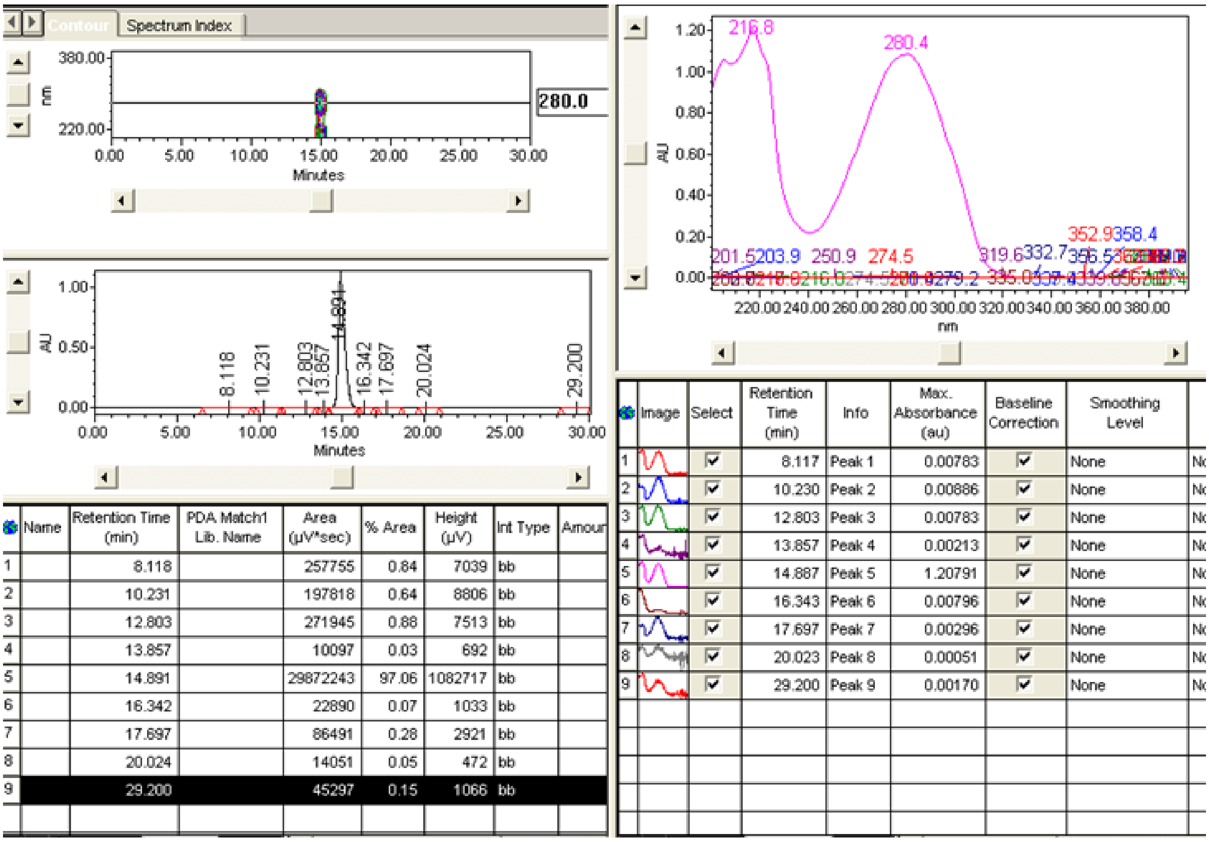

Fig. S6. HPLC analysis of analogue **3**. The purity of **3** was analysed by a Waters HPLC with a photodiode array (PDA) detector using a DIONEX Acclaim® 120 reverse phase column (C18, 5µm, 120Å, 4.6x150 mm) and an isocratic mobile phase of 83% acetonitrile in water at a flow rate of 1.5 mL/min.

References

1. G. Zong, E. Barber, H. Aljewari, J. Zhou, Z. Hu, Y. Du, W.Q. Shi, Total synthesis and biological evaluation of Ipomoeassin F and its unnatural 11R-epimer. J. Org. Chem. 80 (2015) 9279-9291. DOI: 10.1021/acs.joc.5b01765.
2. G. Zong, X. Sun, R. Bhakta, L. Whisenhunt, Z. Hu, F. Wang, W.Q. Shi, New insights into structure-activity relationship of Ipomoeassin F from its bioisosteric 5-oxa/aza analogues. Eur. J. Med. Chem. 144 (2018) 751-757. DOI: 10.1016/j.ejmech.2017.11.022.
3. G. Zong, H. Aljewari, Z. Hu, W.Q. Shi, Revealing the pharmacophore of Ipomoeassin F through molecular editing. Org. Lett. 18 (2016) 1674-1677. DOI: 10.1021/acs.orglett.6b00555.
4. G. Zong, L. Whisenhunt, Z. Hu, W.Q. Shi, Synergistic contribution of tiglate and cinnamate to cytotoxicity of ipomoeassin F. J. Org. Chem. 82 (2017) 4977−4985. DOI: 10.1021/acs.joc.7b00409.
