# Supplementary material for: Synthesis, Biological Evaluation and Docking Studies of Ring-Opened Analogues of Ipomoeassin F
Source: Molecules. 2022 Jul 10;27(14):4419. doi: 10.3390/molecules27144419 (PMC9320607; doi:10.3390/molecules27144419)
Supplement: Supplementary file 1 [file molecules-27-04419-s001.zip › Supplementary material S3.pdf]

Supplementary Material S3:

Synthesis, Biological Evaluation and Docking Studies of Ring-Opened Analogues of Ipomoeassin F

Sarah O'Keefe, Pratiti Bhadra, Kwabena B. Duah, Guanghui Zong, Levise Tenay, Lauren Andrews, Hayden Schneider, Ashley Anderson, Zhijian Hu, Hazim S. Aljewari, Belinda S. Hall, *Rachel E. Simmonds,* Volkhard Helms, Stephen High and Wei Q. Shi

Table of Contents

1	^{1}H NMR spectrum of **S2**	S4
2	^{13}C NMR spectrum of **S2**	S4
3	COSY NMR spectrum of **S2**	S5
4	HSQC NMR spectrum of **S2**	S5
5	^{1}H NMR spectrum of **S3**	S6
6	^{13}C NMR spectrum of **S3**	S6
7	COSY NMR spectrum of **S3**	S7
8	HSQC NMR spectrum of **S3**	S7
9	HMBC NMR spectrum of **S3**	S8
10	^{1}H NMR spectrum of **4**	S8
11	^{13}C NMR spectrum of **4**	S9
12	COSY NMR spectrum of **4**	S9
13	HSQC NMR spectrum of **4**	S10
14	HMBC NMR spectrum of **4**	S10
15	^{1}H NMR spectrum of **5**	S11
16	^{13}C NMR spectrum of **5**	S11
17	COSY NMR spectrum of **5**	S12
18	HSQC NMR spectrum of **5**	S12
19	^{1}H NMR spectrum of **6**	S13
20	^{13}C NMR spectrum of **6**	S13
21	COSY NMR spectrum of **6**	S14
22	HSQC NMR spectrum of **6**	S14
23	HMBC NMR spectrum of **6**	S15
24	^{1}H NMR spectrum of **7**	S15
25	^{13}C NMR spectrum of **7**	S16
26	COSY NMR spectrum of **7**	S16
27	HSQC NMR spectrum of **7**	S17
28	^{1}H NMR spectrum of **8**	S17
29	^{13}C NMR spectrum of **8**	S18
30	COSY NMR spectrum of **8**	S18
31	HSQC NMR spectrum of **8**	S19
32	HMBC NMR spectrum of **8**	S19
33	^{1}H NMR spectrum of **9**	S20
34	^{13}C NMR spectrum of **9**	S20
35	COSY NMR spectrum of **9**	S21
36	HSQC NMR spectrum of **9**	S21
37	HMBC NMR spectrum of **9**	S22
38	^{1}H NMR spectrum of **10**	S22
39	^{13}C NMR spectrum of **10**	S23
40	COSY NMR spectrum of **10**	S23
41	HSQC NMR spectrum of **10**	S24
42	HMBC NMR spectrum of **10**	S24
43	^{1}H NMR spectrum of **11**	S25
44	^{13}C NMR spectrum of **11**	S25
45	COSY NMR spectrum of **11**	S26
46	HSQC NMR spectrum of **11**	S26
47	HMBC NMR spectrum of **11**	S27
48	^{1}H NMR spectrum of **12**	S27
49	^{13}C NMR spectrum of **12**	S28
50	COSY NMR spectrum of **12**	S28
51	HSQC NMR spectrum of **12**	S29
52	^{1}H NMR spectrum of **28**	S30
53	^{13}C NMR spectrum of **28**	S30
54	^{1}H NMR spectrum of **S8**	S31

55	^{13}C NMR spectrum of **S8**	S31
56	COSY NMR spectrum of **S8**	S32
57	HMQC NMR spectrum of **S8**	S32
58	^{1}H NMR spectrum of **S9**	S33
59	^{13}C NMR spectrum of **S9**	S33
60	COSY NMR spectrum of **S9**	S34
61	HMQC NMR spectrum of **S9**	S34
62	^{1}H NMR spectrum of **24**	S35
63	^{13}C NMR spectrum of **24**	S35
64	COSY NMR spectrum of **24**	S36
65	HMQC NMR spectrum of **24**	S36
66	^{1}H NMR spectrum of **25**	S37
67	^{13}C NMR spectrum of **25**	S37
68	COSY NMR spectrum of **25**	S38
69	HMQC NMR spectrum of **25**	S38
70	^{1}H NMR spectrum of **26**	S39
71	^{13}C NMR spectrum of **26**	S39
72	COSY NMR spectrum of **26**	S40
73	HMQC NMR spectrum of **26**	S40
74	^{1}H NMR spectrum of **27**	S41
75	^{13}C NMR spectrum of **27**	S41
76	COSY NMR spectrum of **27**	S42

77	HMQC NMR spectrum of **27**	S42
78	^{1}H NMR spectrum of **29**	S43
79	^{13}C NMR spectrum of **29**	S43
80	COSY NMR spectrum of **29**	S44
81	HMQC NMR spectrum of **29**	S44
82	^{1}H NMR spectrum of **30**	S45
83	^{13}C NMR spectrum of **30**	S45
84	COSY NMR spectrum of **30**	S46
85	HMQC NMR spectrum of **30**	S46
86	^{1}H NMR spectrum of **31**	S47
87	^{13}C NMR spectrum of **31**	S47
88	COSY NMR spectrum of **31**	S48
89	HMQC NMR spectrum of **31**	S48
90	^{1}H NMR spectrum of **32**	S49
91	^{13}C NMR spectrum of **32**	S49
92	COSY NMR spectrum of **32**	S50
93	HMQC NMR spectrum of **32**	S50
94	^{1}H NMR spectrum of **3**	S51
95	^{13}C NMR spectrum of **3**	S51
96	COSY NMR spectrum of **3**	S52
97	HMQC NMR spectrum of **3**	S52
98	HMBC NMR spectrum of **3**	S53

^{1}H NMR spectrum of **S2** ($CDCl_3$, 400 MHz)

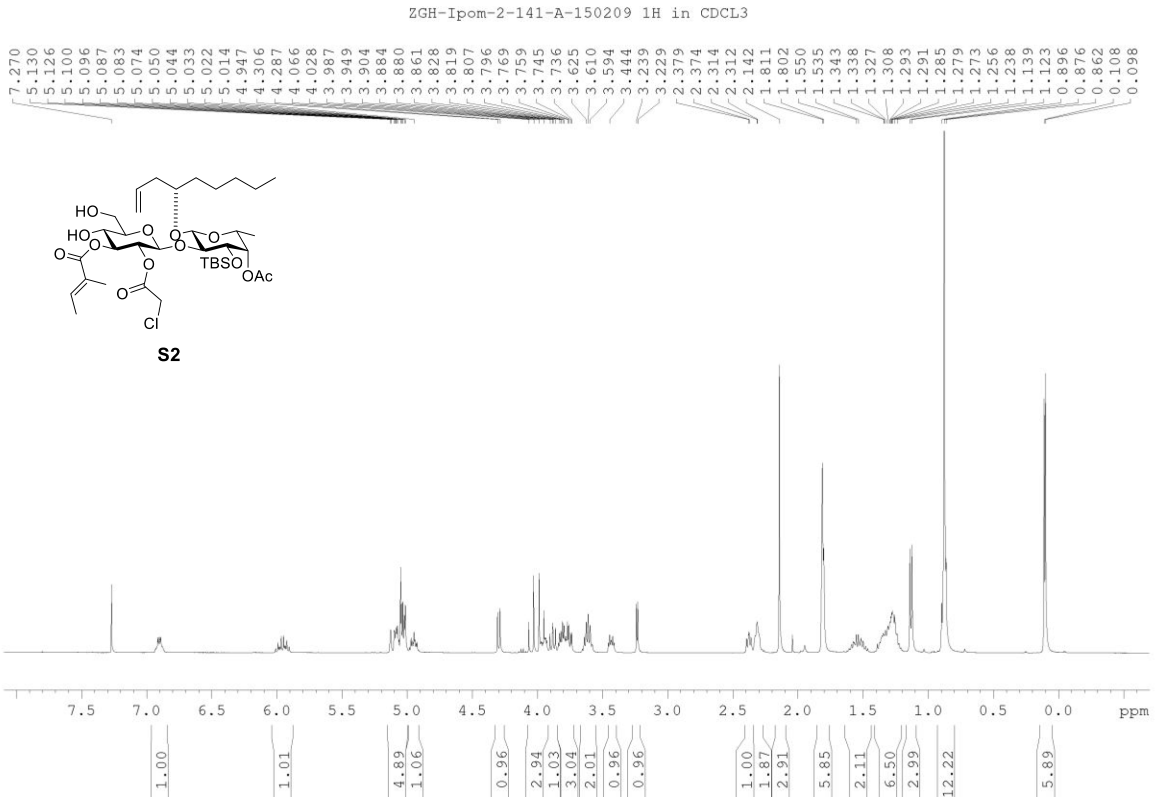

^{13}C NMR spectrum of **S2** ($CDCl_3$, 400 MHz)

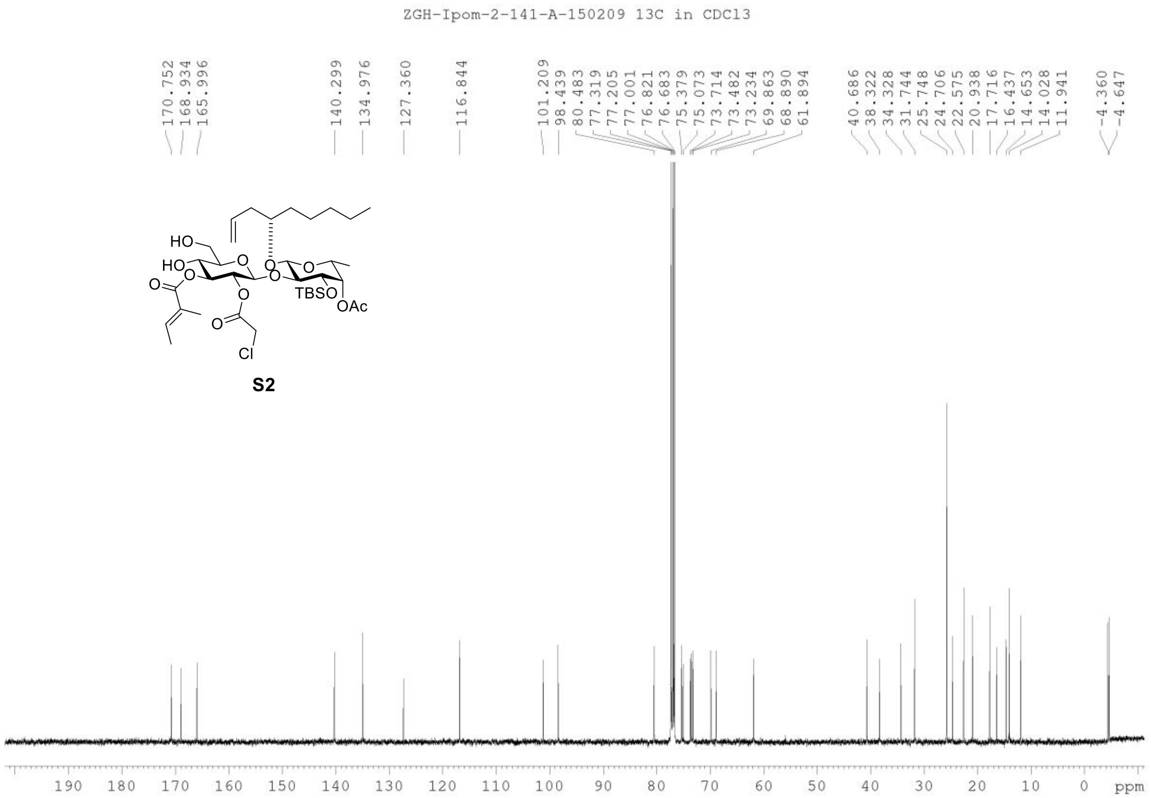

COSY NMR spectrum of **S2** ($CDCl_3$, 400 MHz)

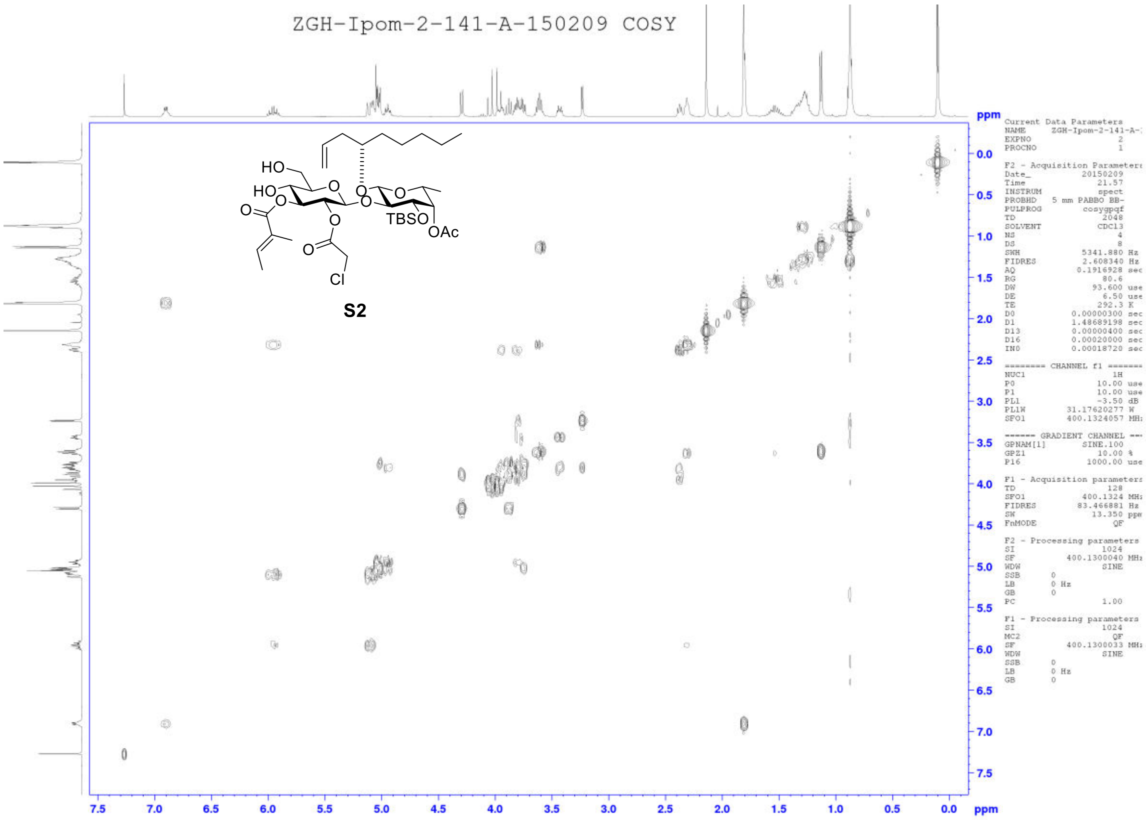

HSQC NMR spectrum of **S2** ($CDCl_3$, 400 MHz)

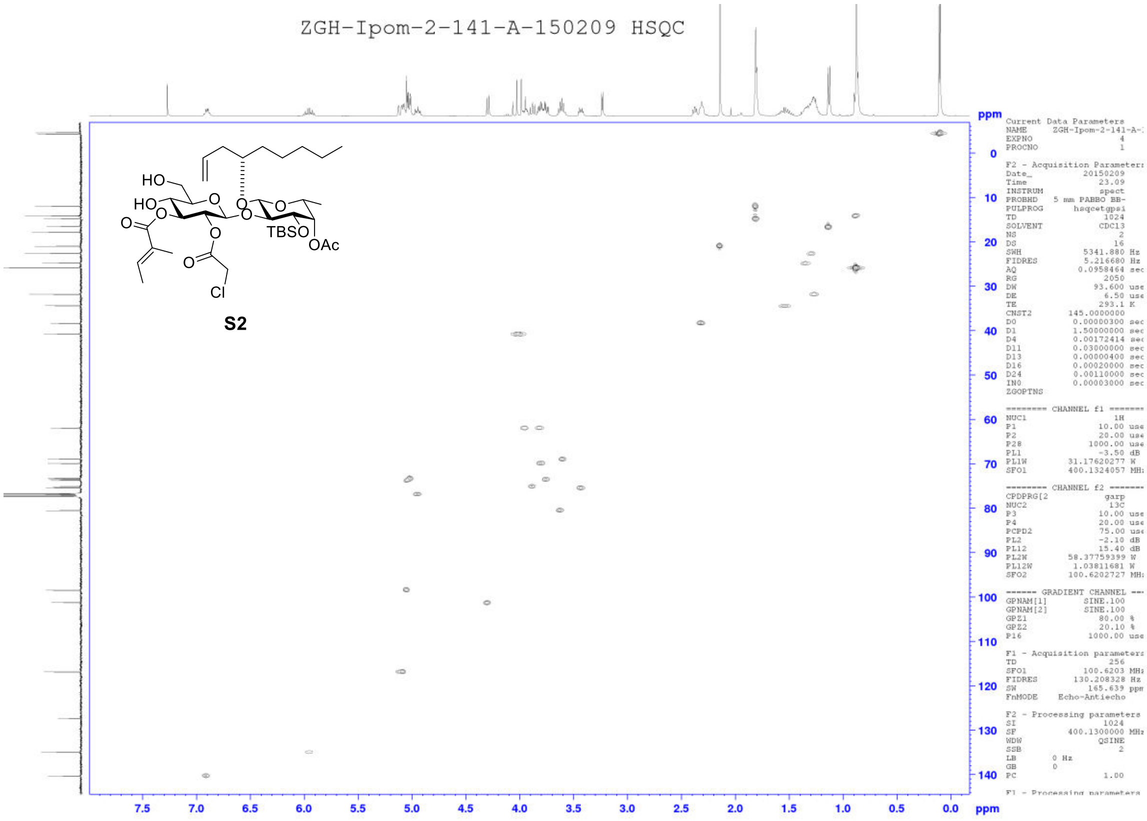

^{1}H NMR spectrum of **S3** ($CDCl_3$, 400 MHz)

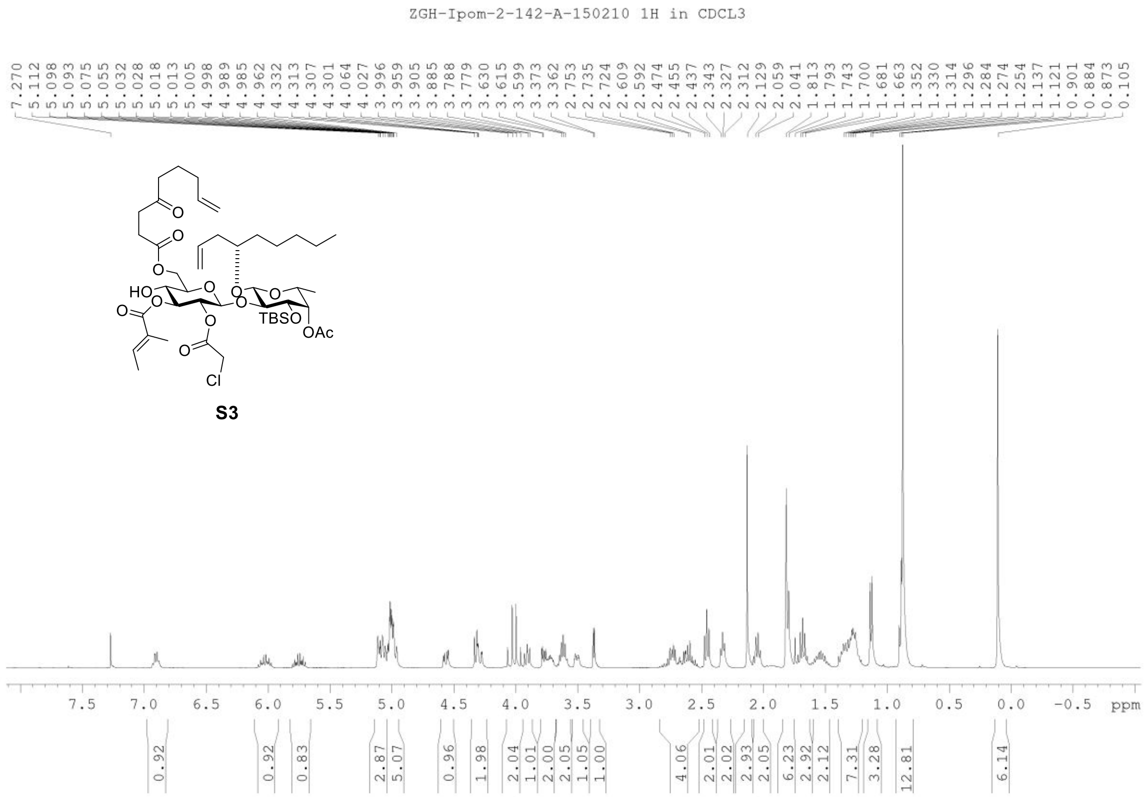

^{13}C NMR spectrum of **S3** ($CDCl_3$, 400 MHz)

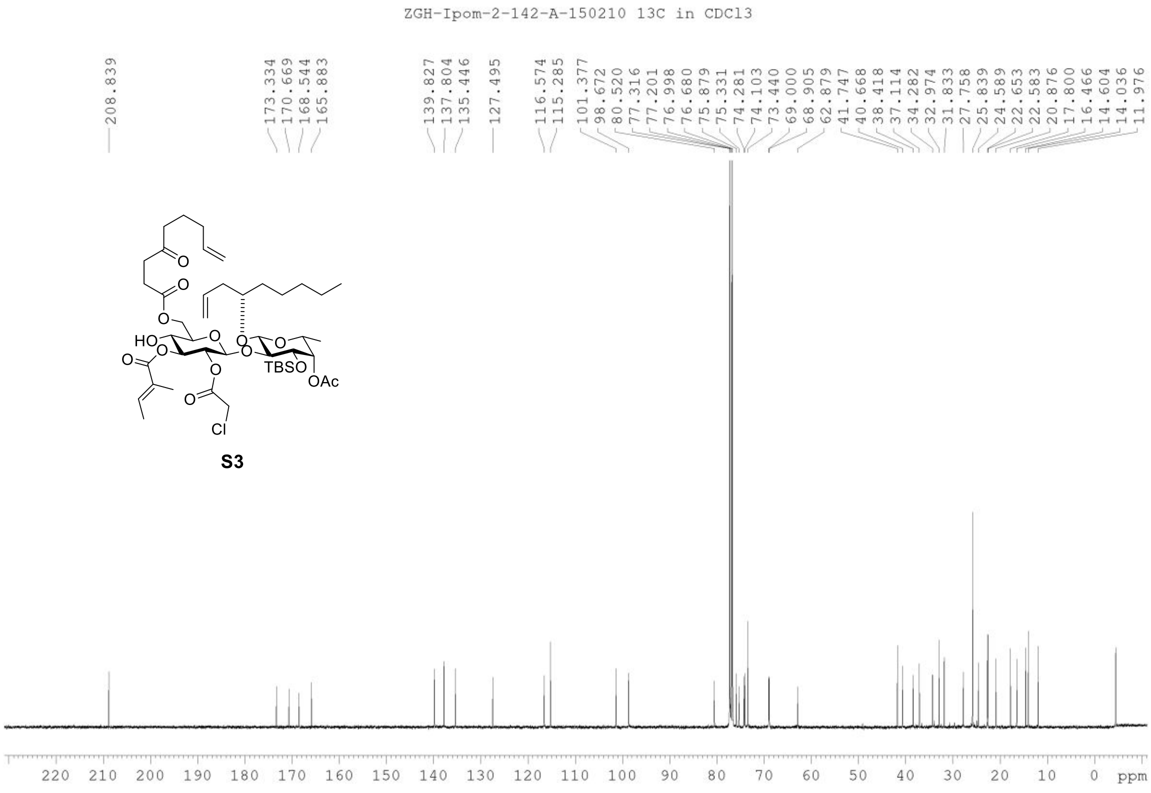

COSY NMR spectrum of **S3** ($CDCl_3$, 400 MHz)

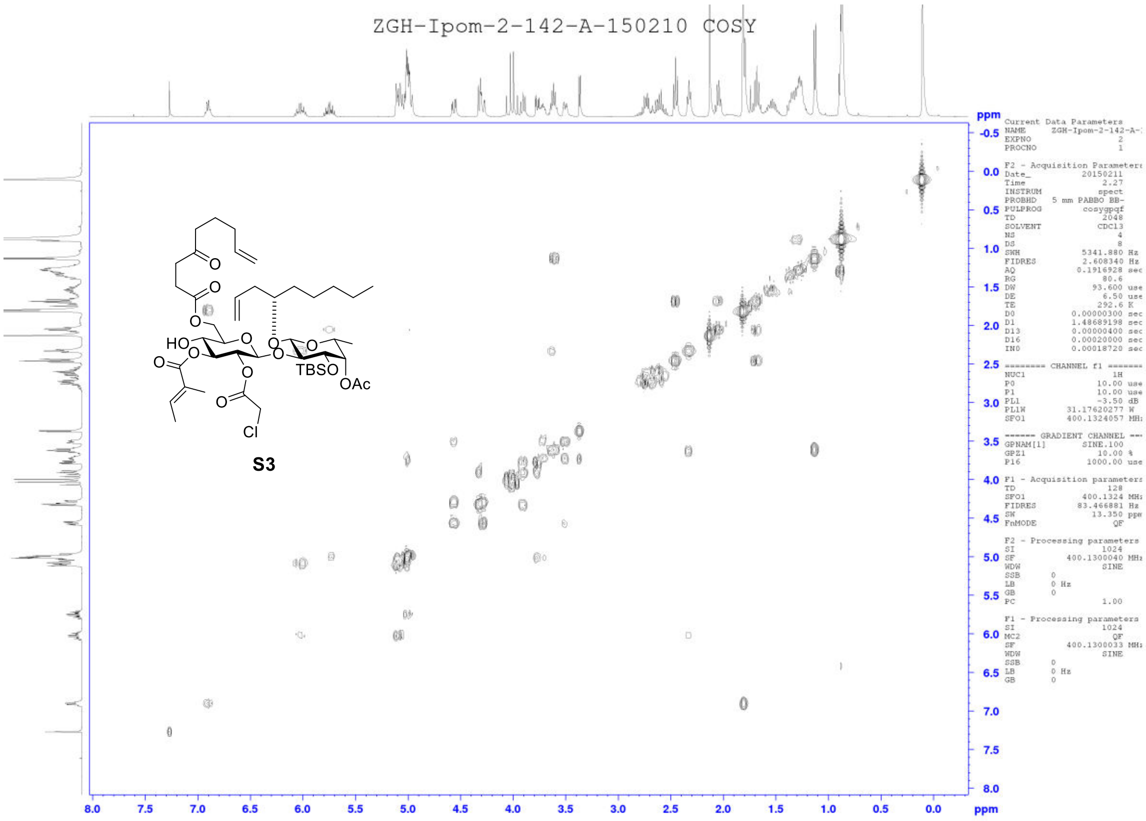

HSQC NMR spectrum of **S3** ($CDCl_3$, 400 MHz)

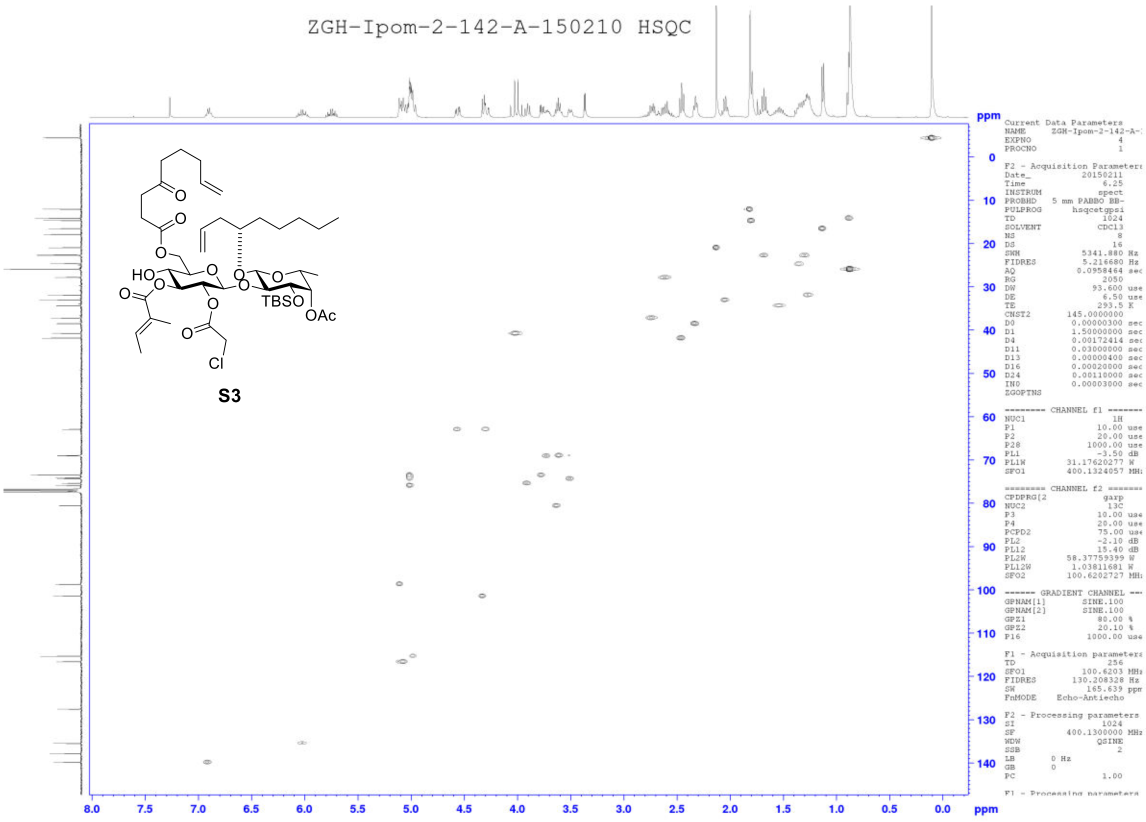

HMBC NMR spectrum of **S3** ($CDCl_3$, 400 MHz)

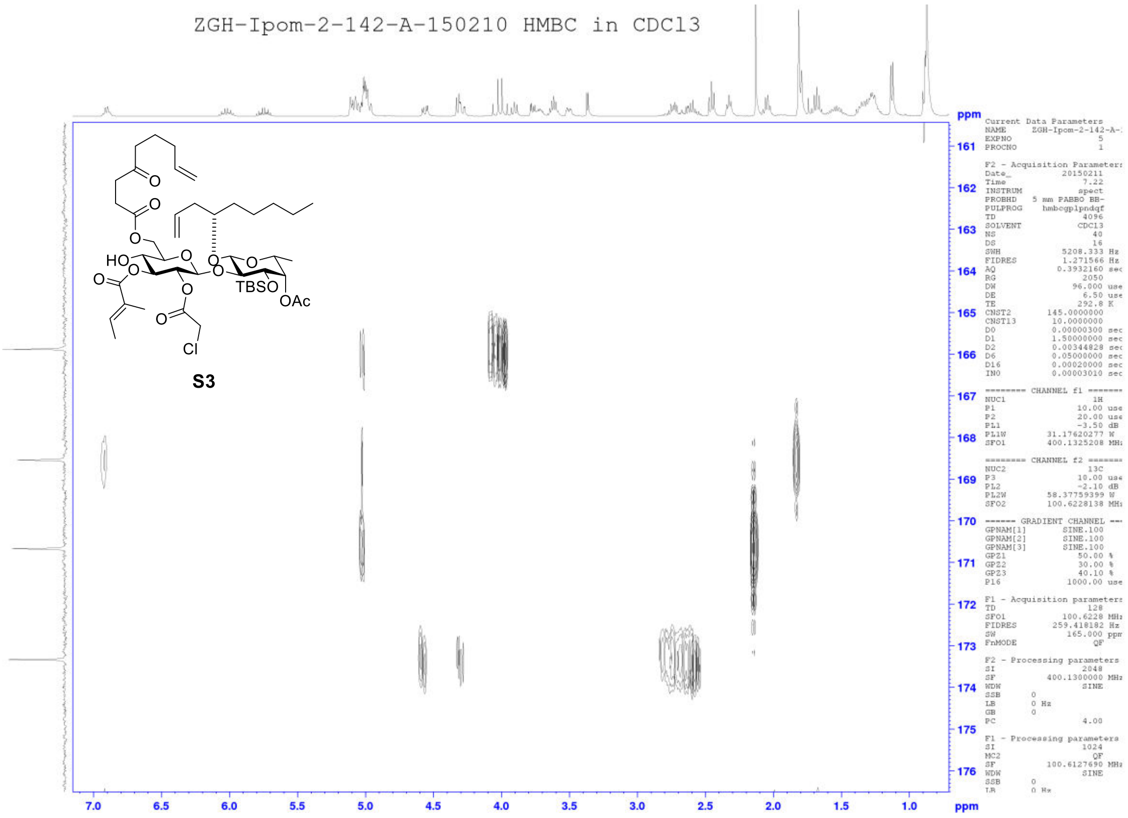

^{1}H NMR spectrum of **4** ($CDCl_3$, 400 MHz)

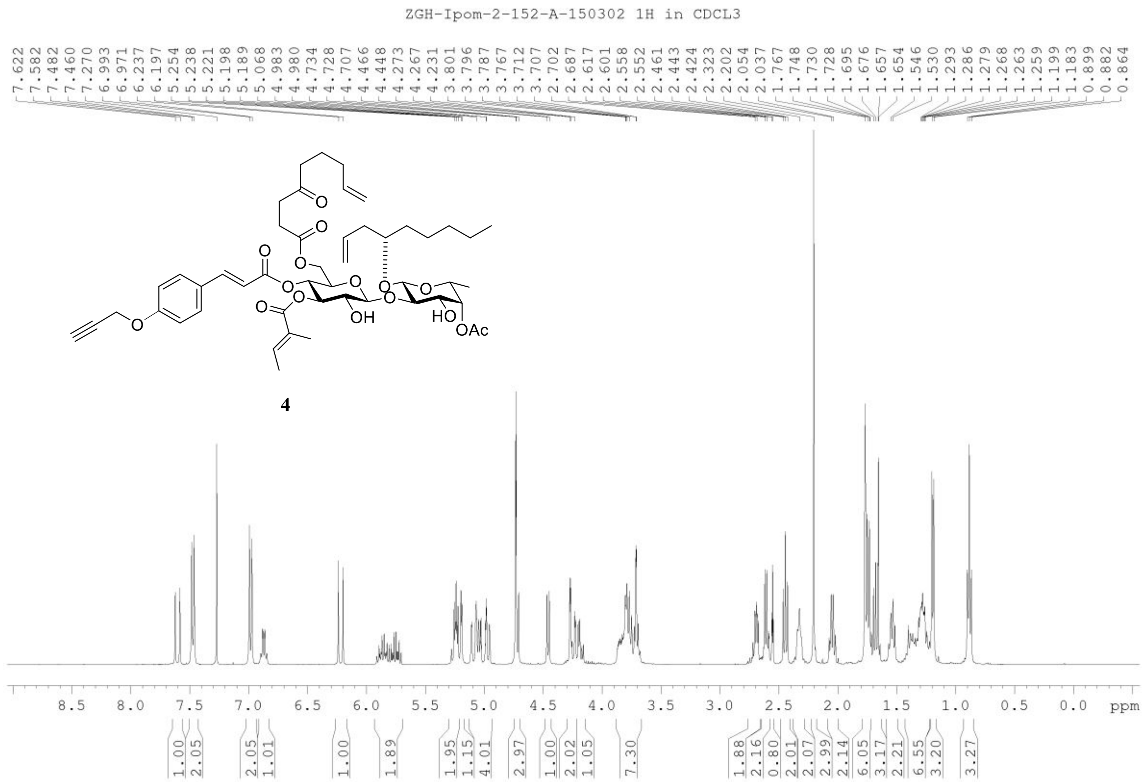

^{13}C NMR spectrum of **4** ($CDCl_3$, 400 MHz)

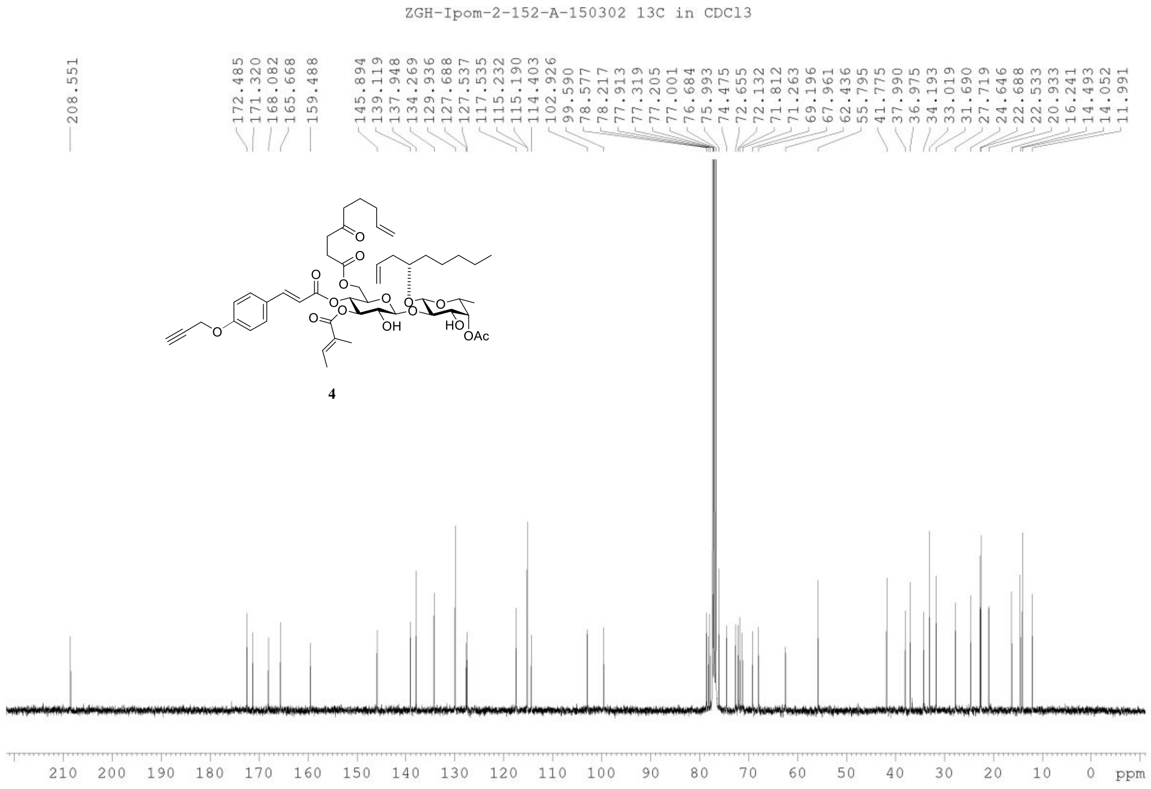

COSY NMR spectrum of **4** ($CDCl_3$, 400 MHz)

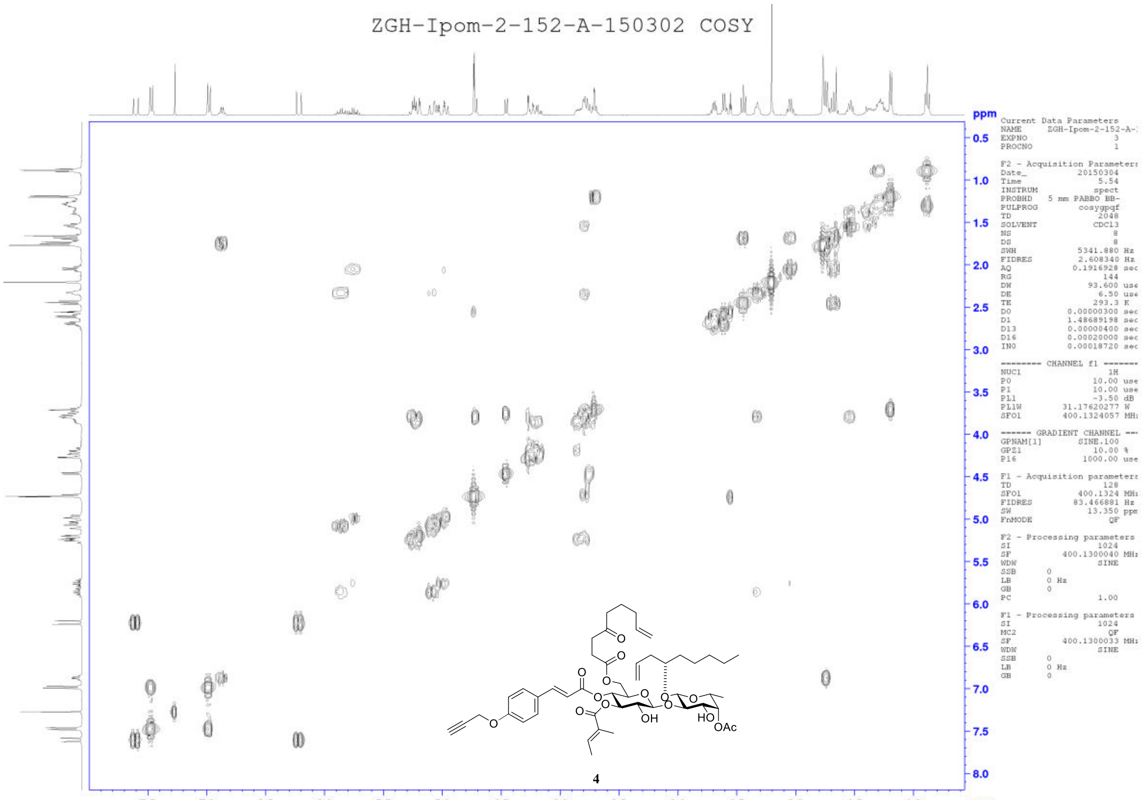

HSQC NMR spectrum of **4** ($CDCl_3$, 400 MHz)

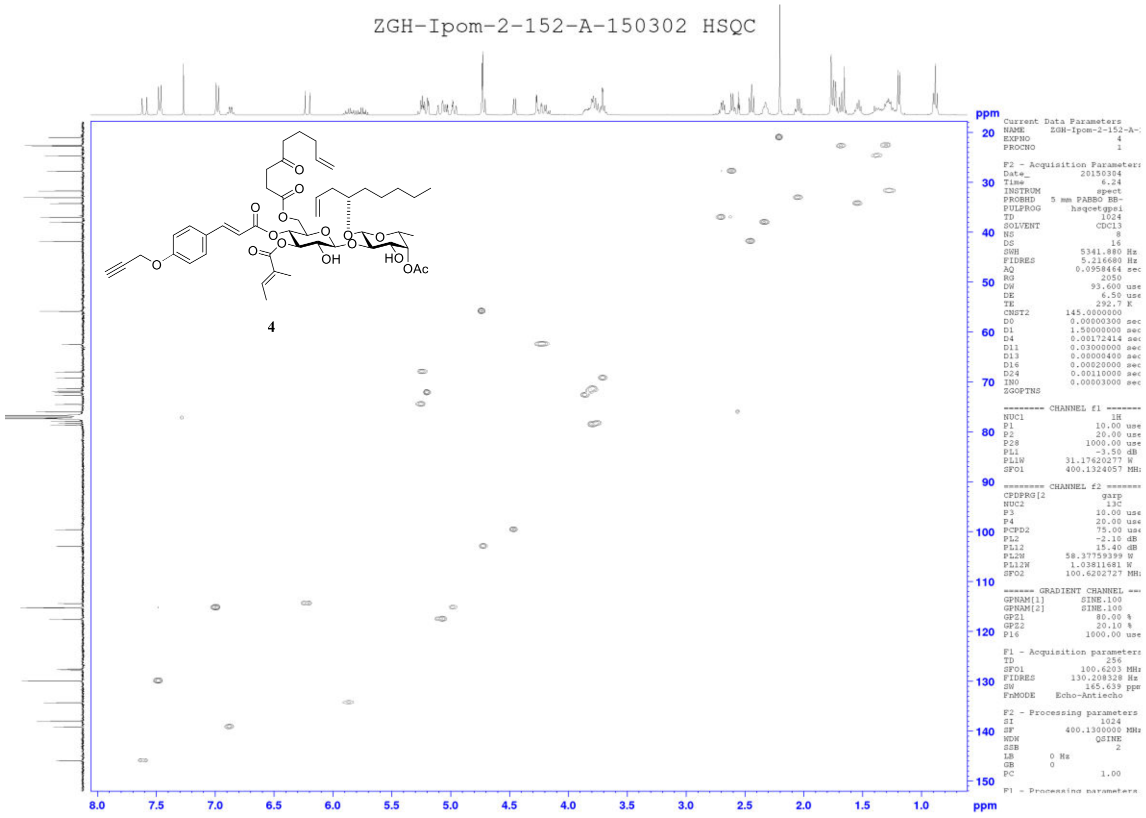

HMBC NMR spectrum of **4** ($CDCl_3$, 400 MHz)

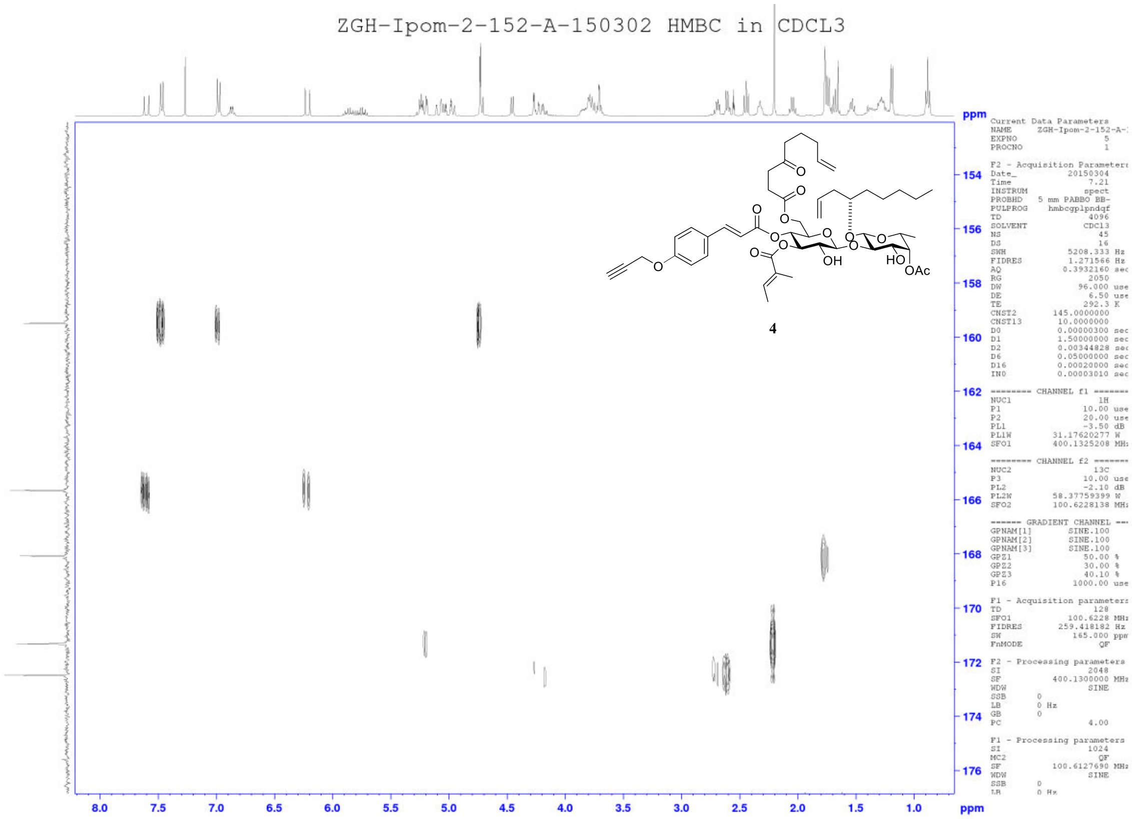

^{1}H NMR spectrum of **5** ($CDCl_3$, 400 MHz)

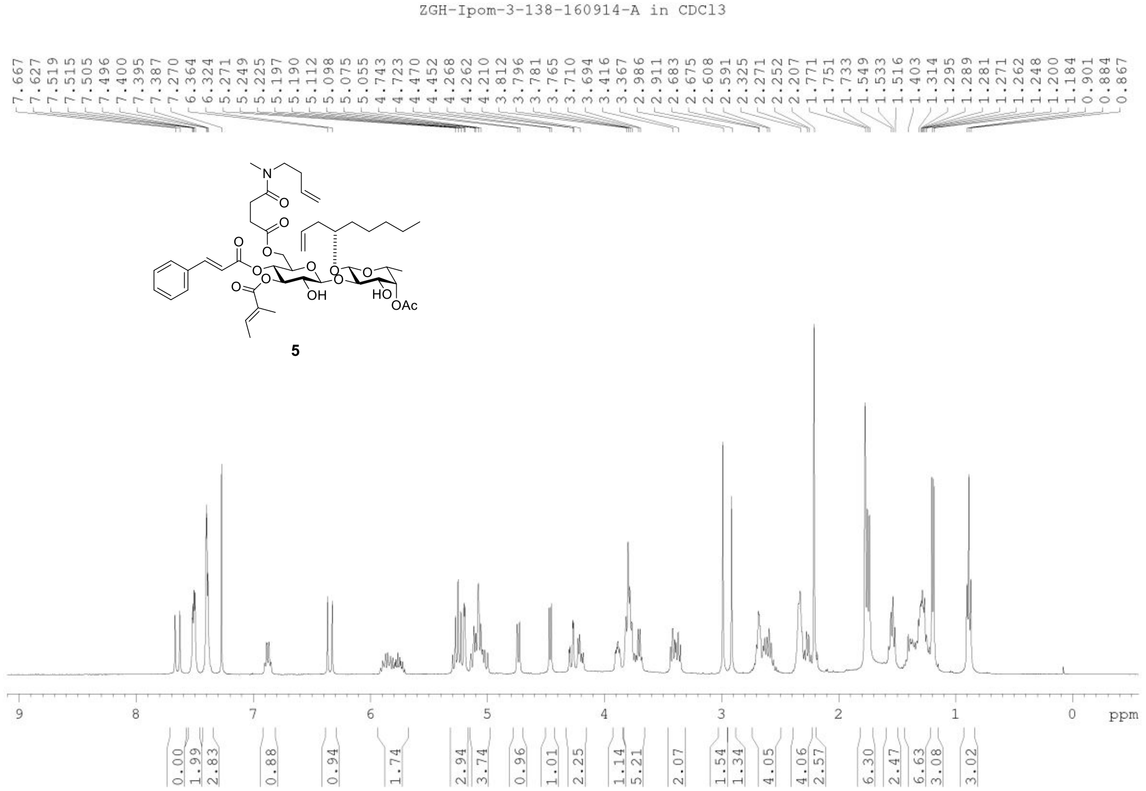

^{13}C NMR spectrum of **5** ($CDCl_3$, 400 MHz)

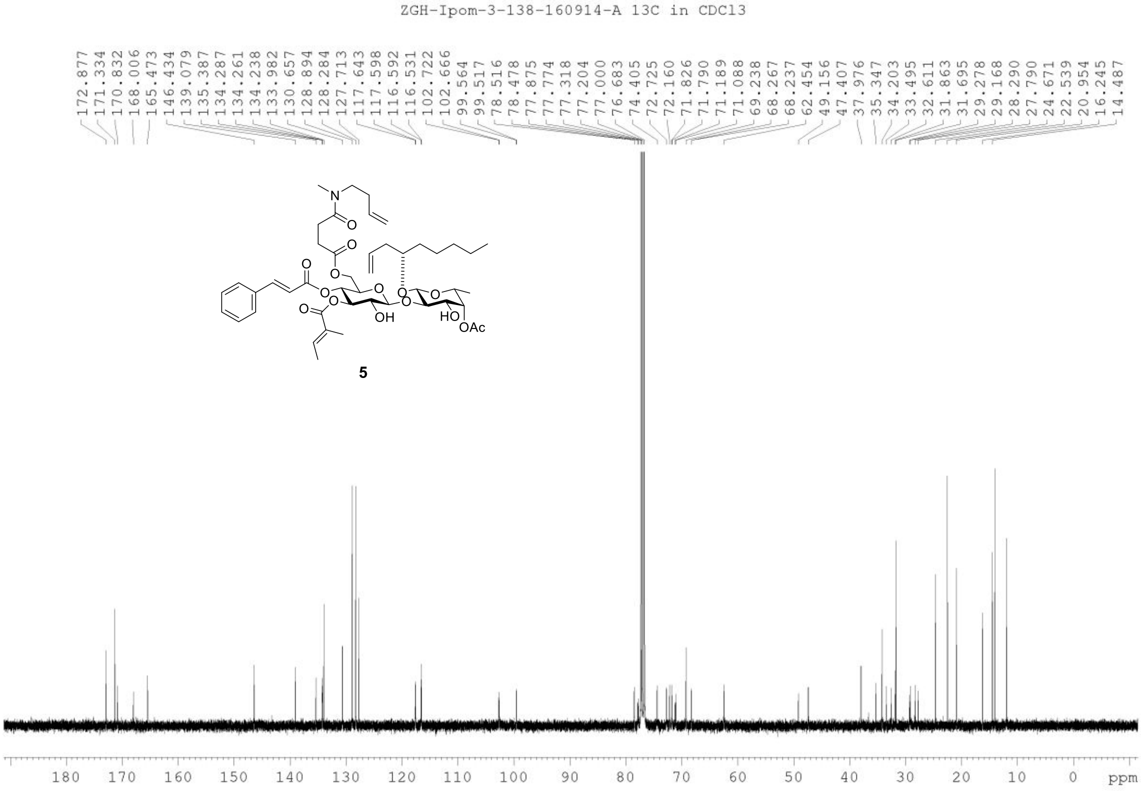

COSY NMR spectrum of **5** ($CDCl_3$, 400 MHz)

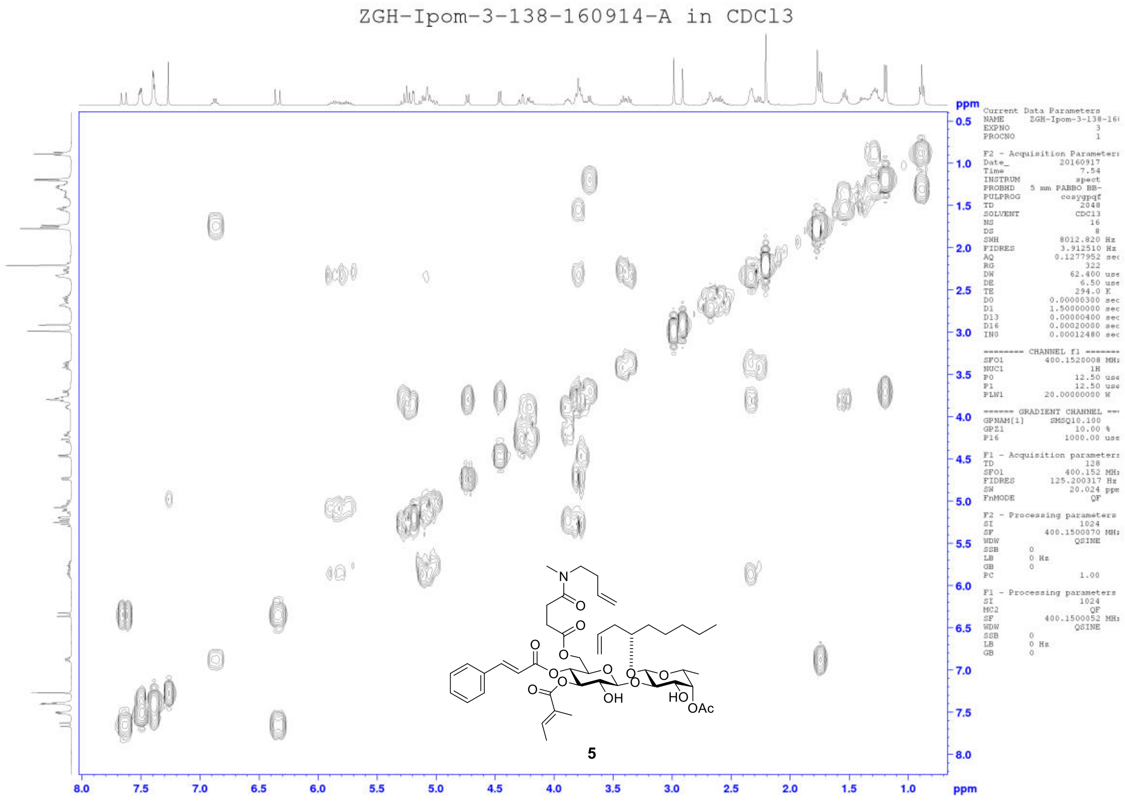

HSQC NMR spectrum of **5** ($CDCl_3$, 400 MHz)

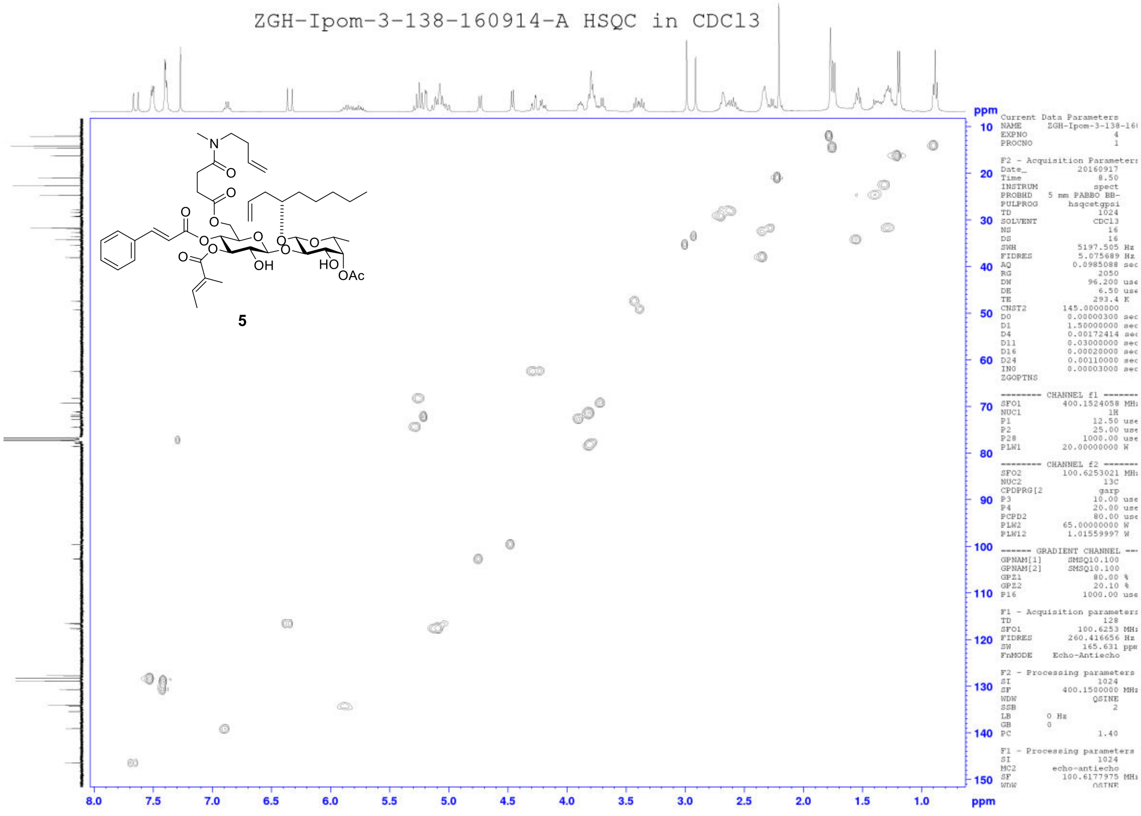

^{1}H NMR spectrum of **6** ($CDCl_3$, 400 MHz)

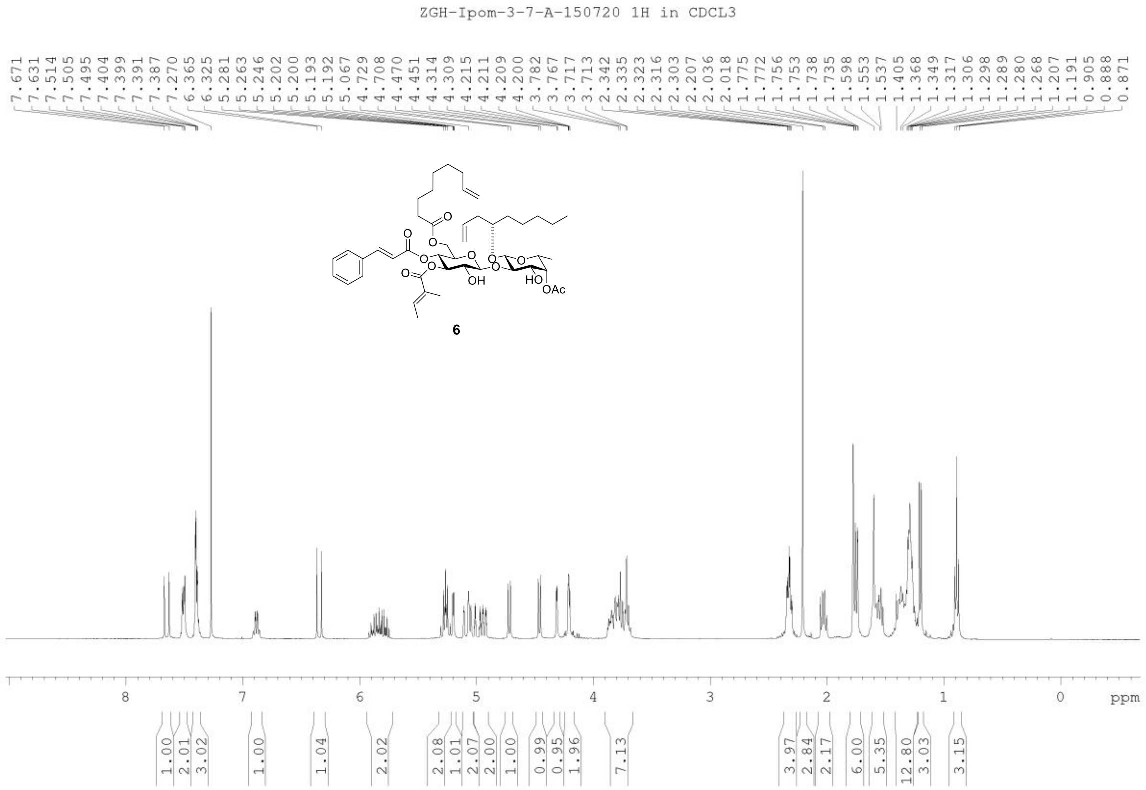

^{13}C NMR spectrum of **6** ($CDCl_3$, 400 MHz)

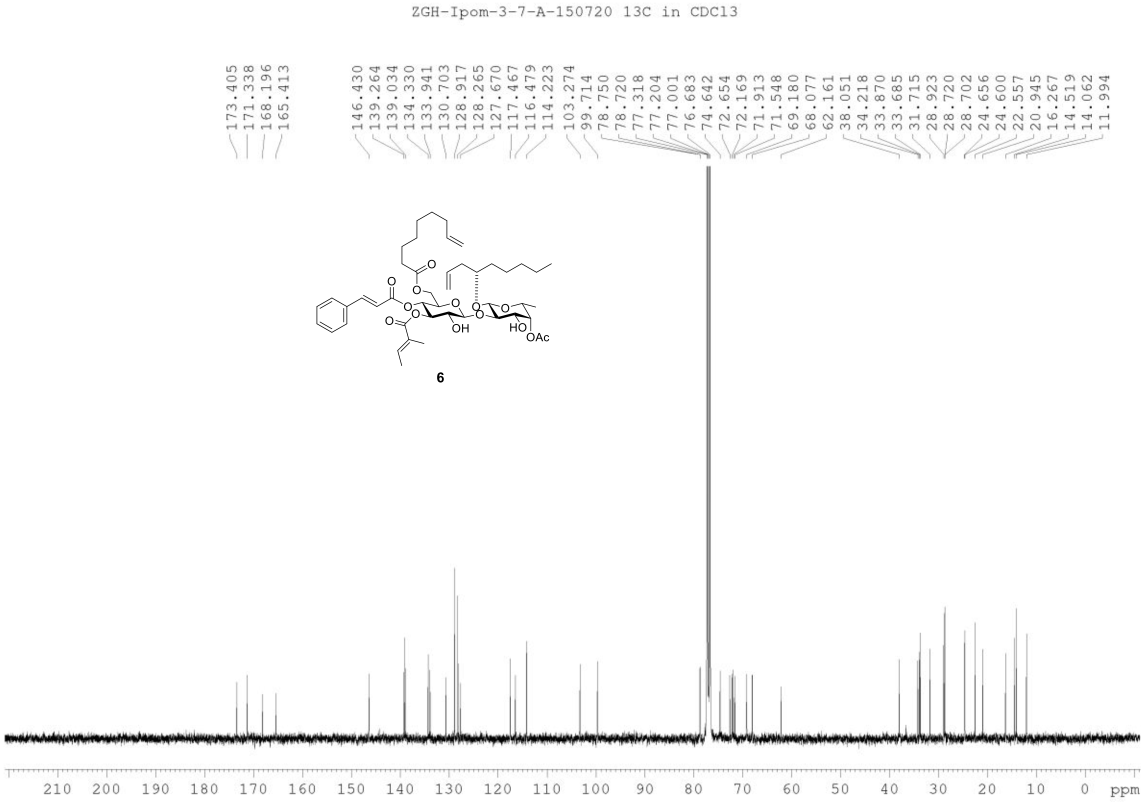

COSY NMR spectrum of **6** ($CDCl_3$, 400 MHz)

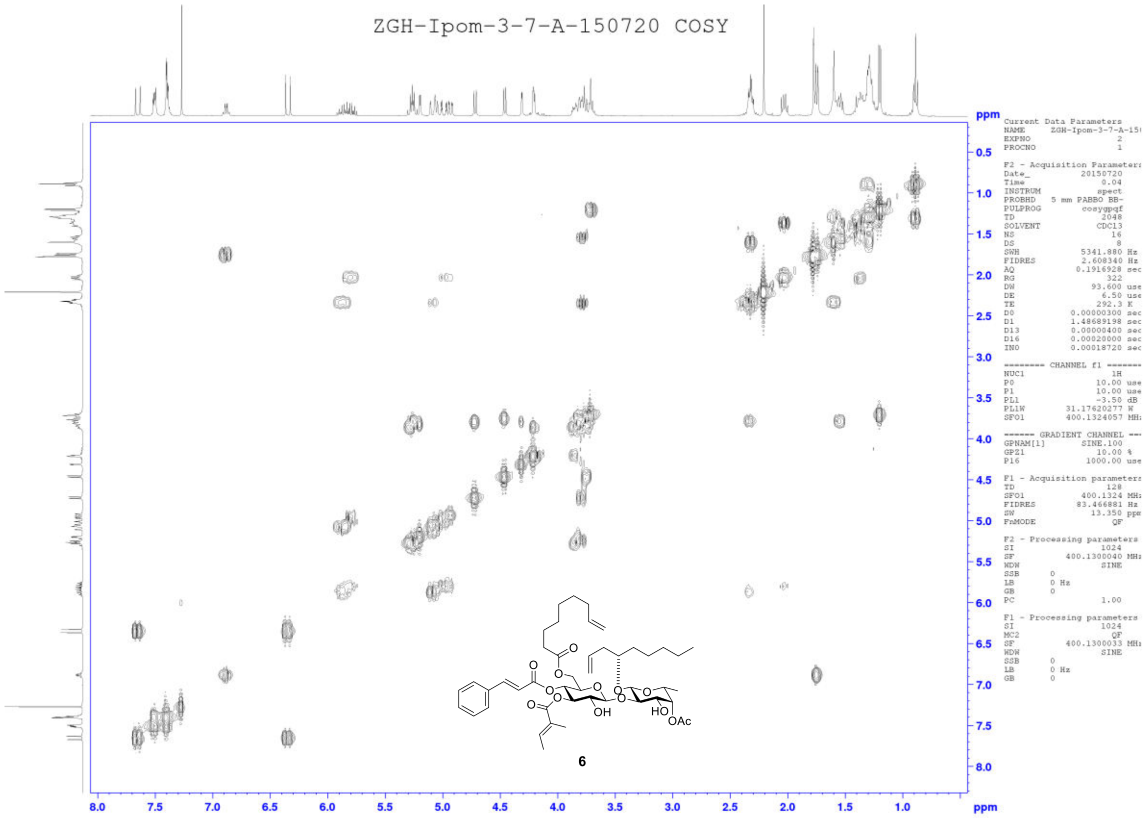

HSQC NMR spectrum of **6** ($CDCl_3$, 400 MHz)

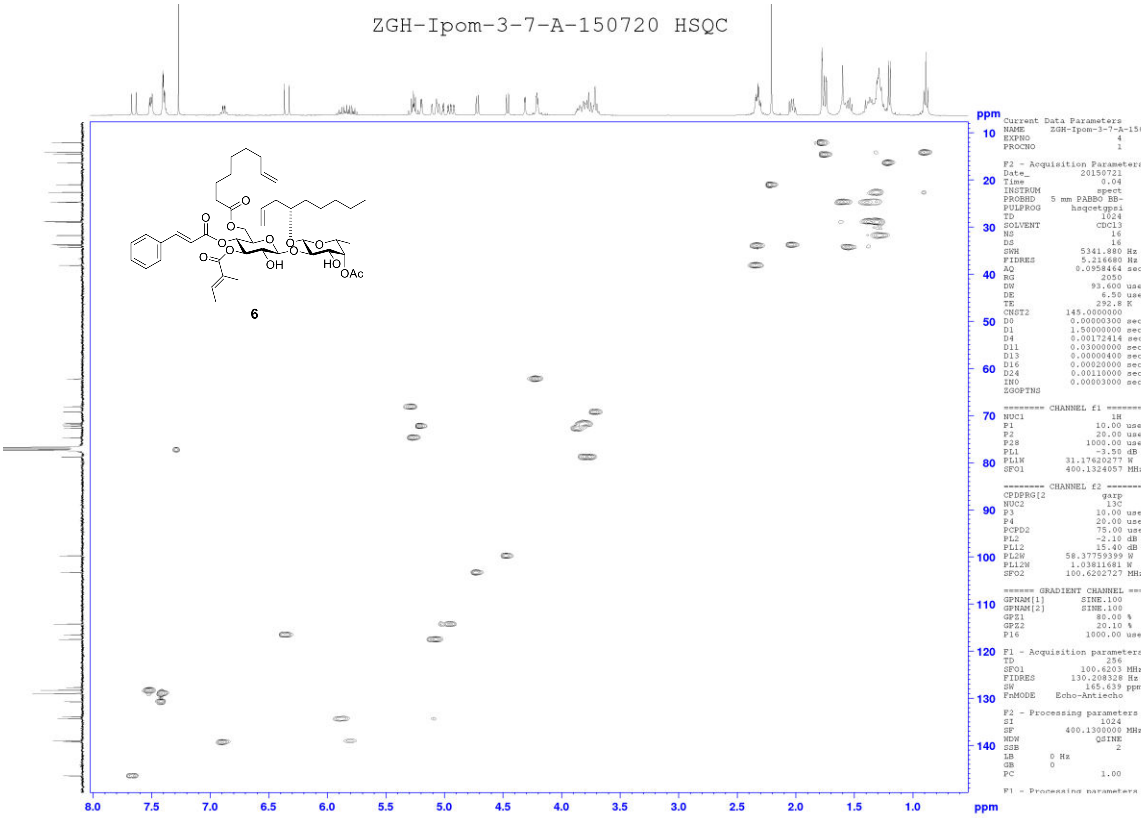

HMBC NMR spectrum of **6** ($CDCl_3$, 400 MHz)

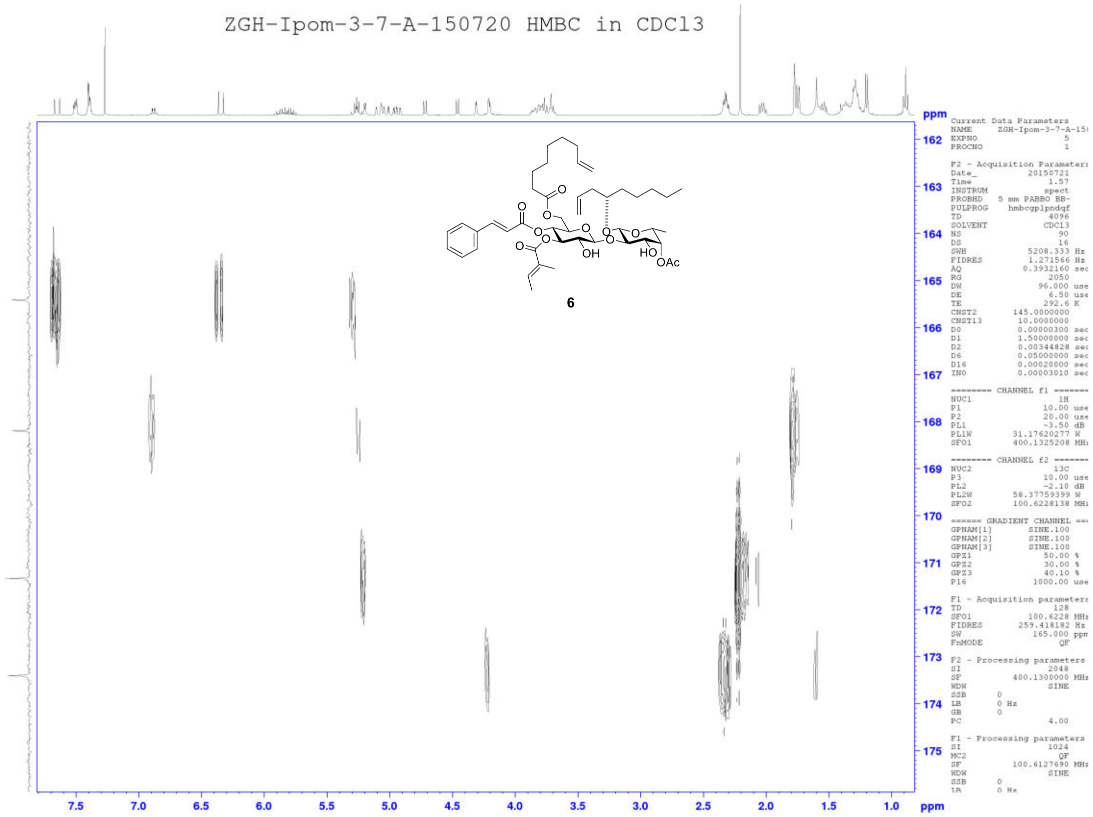

^{1}H NMR spectrum of **7** ($CDCl_3$, 400 MHz)

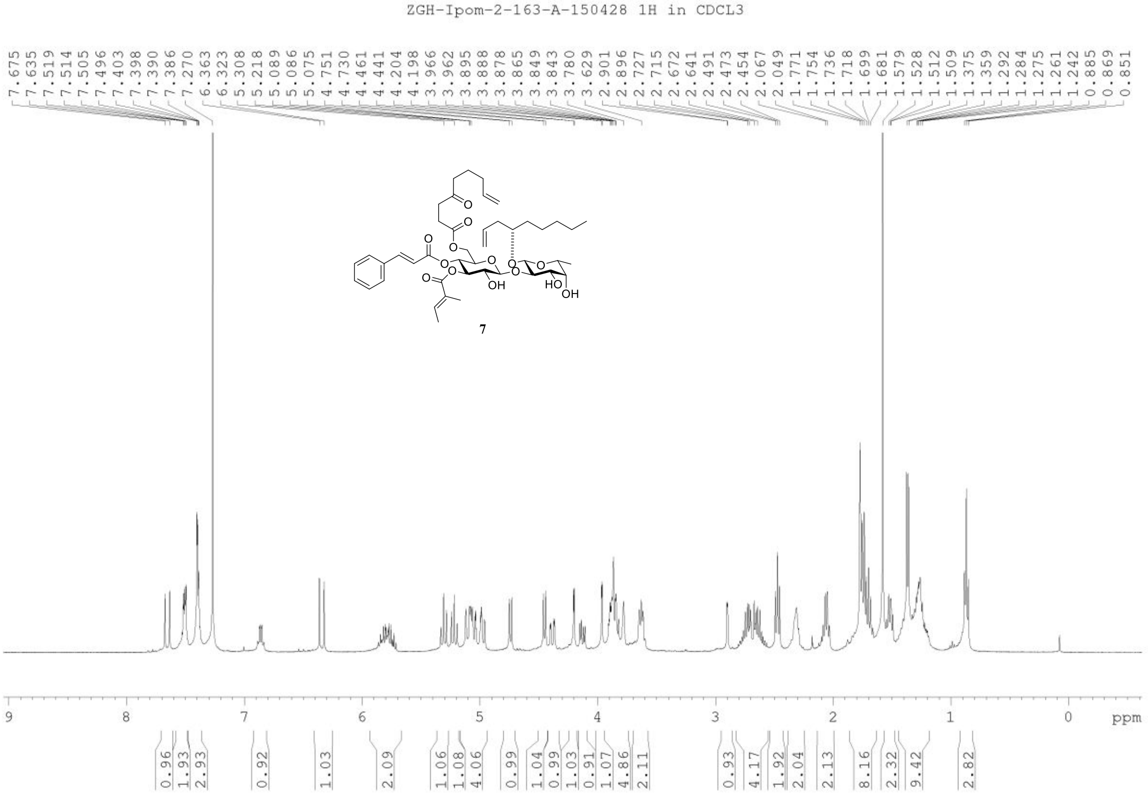

^{13}C NMR spectrum of **7** ($CDCl_3$, 400 MHz)

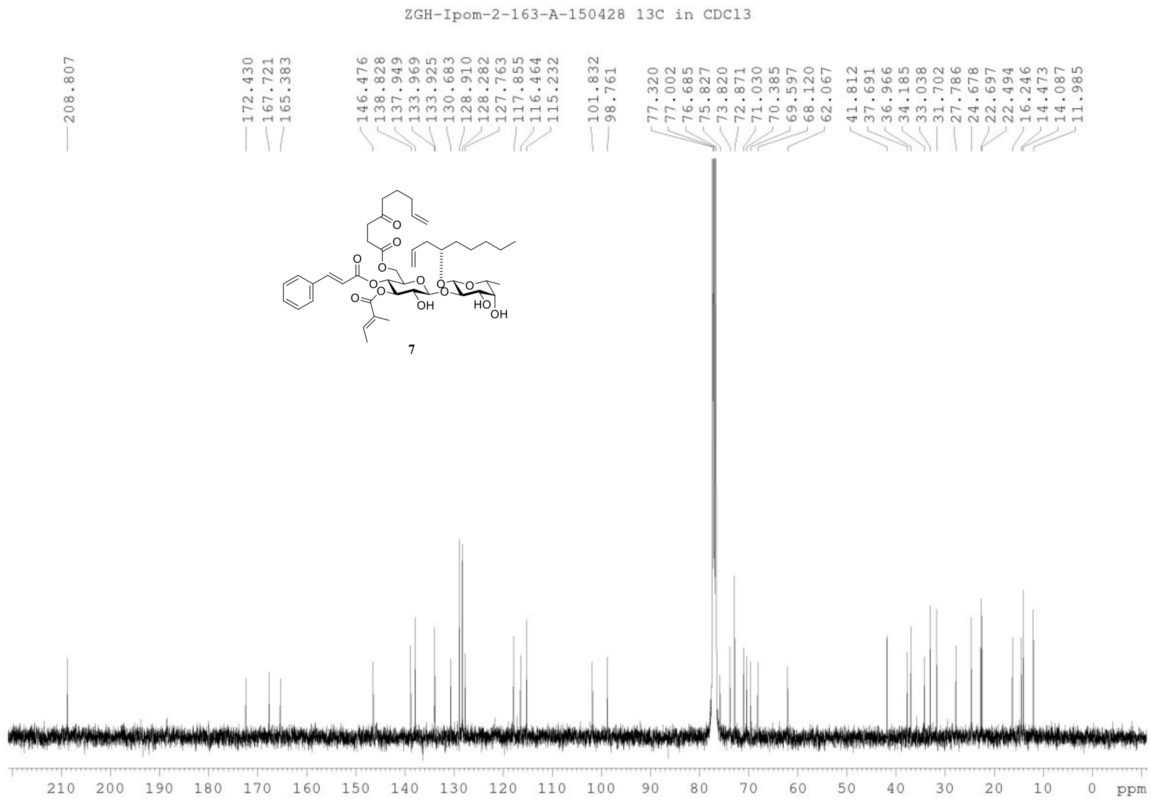

COSY NMR spectrum of **7** ($CDCl_3$, 400 MHz)

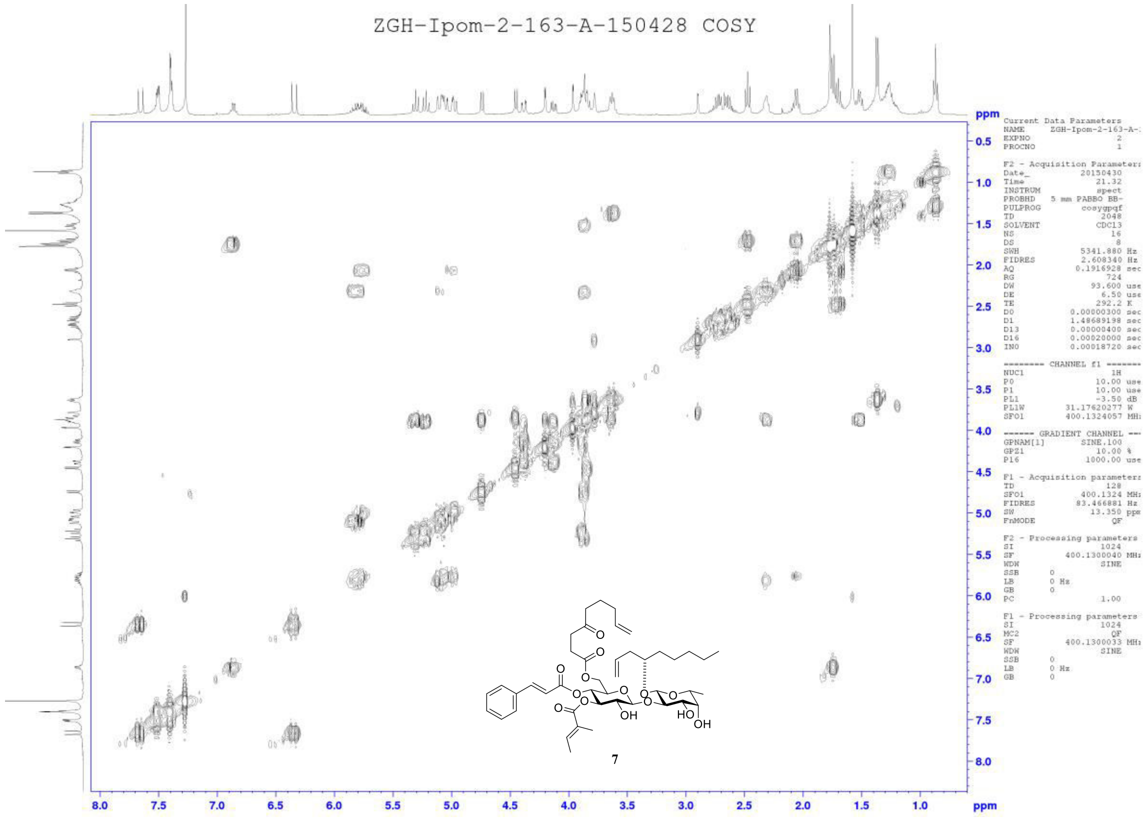

HSQC NMR spectrum of **7** ($CDCl_3$, 400 MHz)

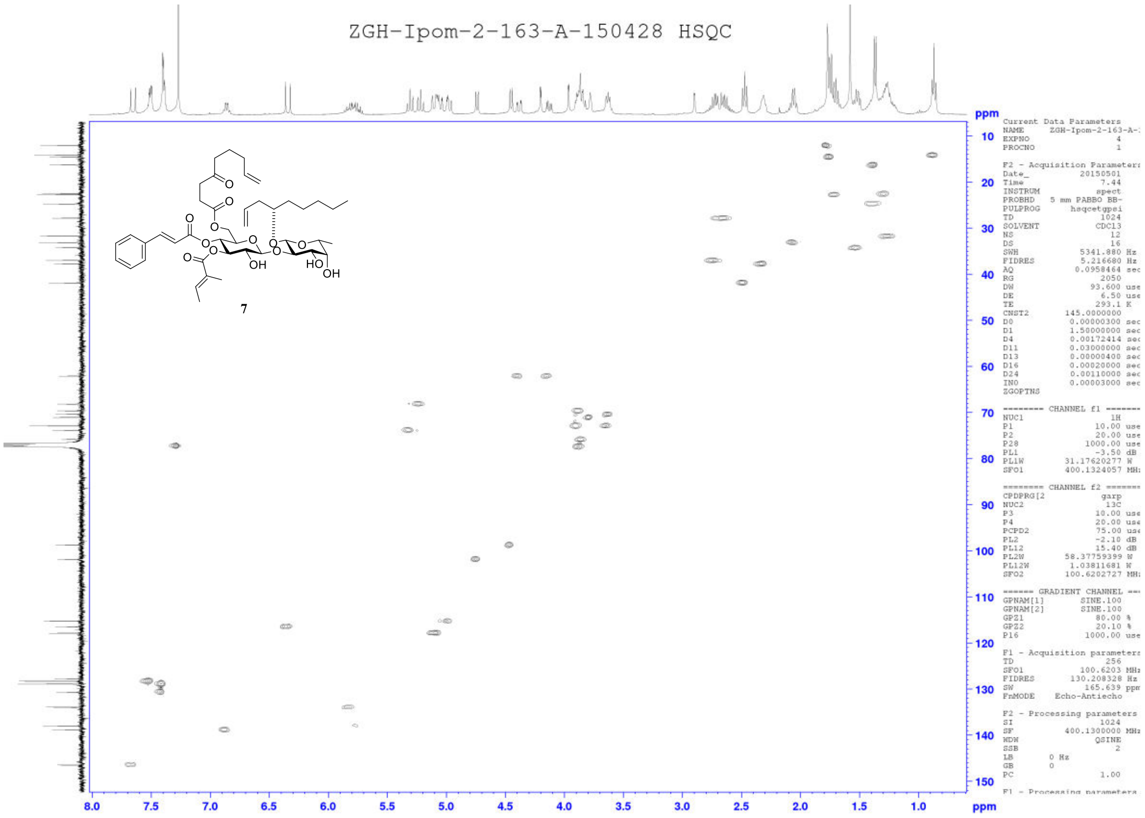

^{1}H NMR spectrum of **8** ($CDCl_3$, 400 MHz)

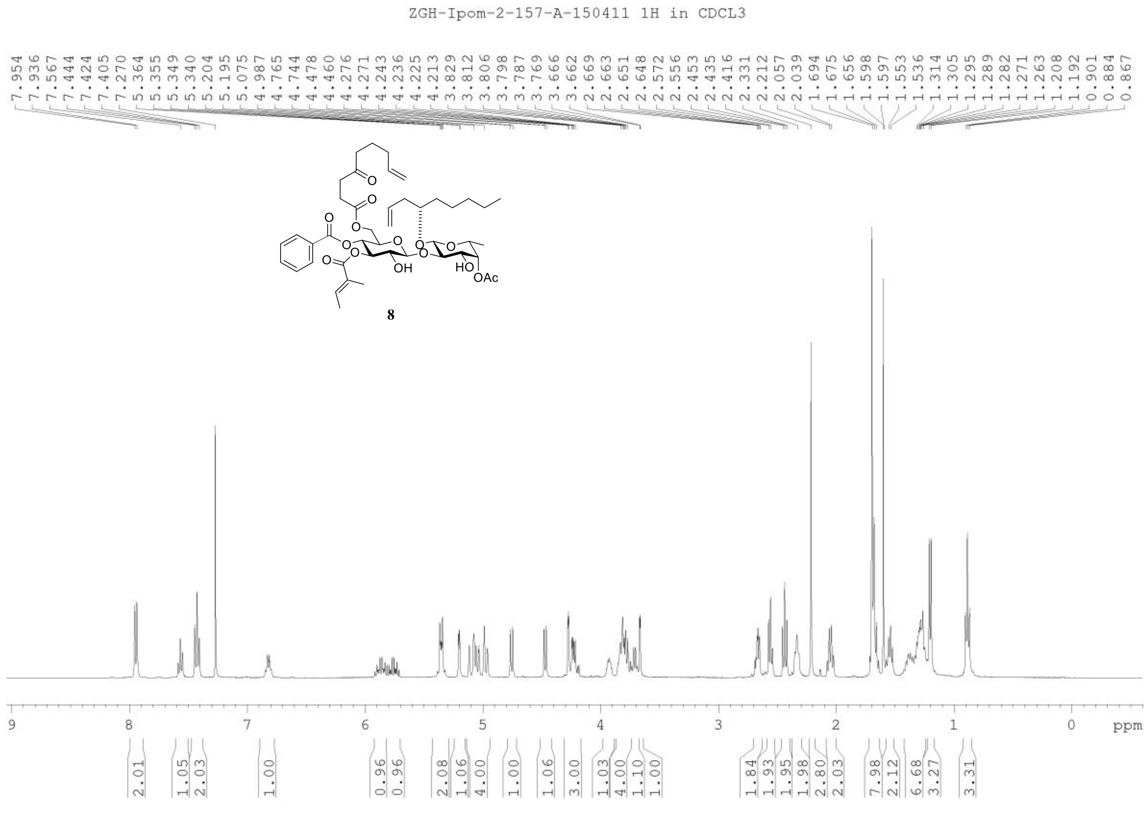

^{13}C NMR spectrum of **8** ($CDCl_3$, 400 MHz)

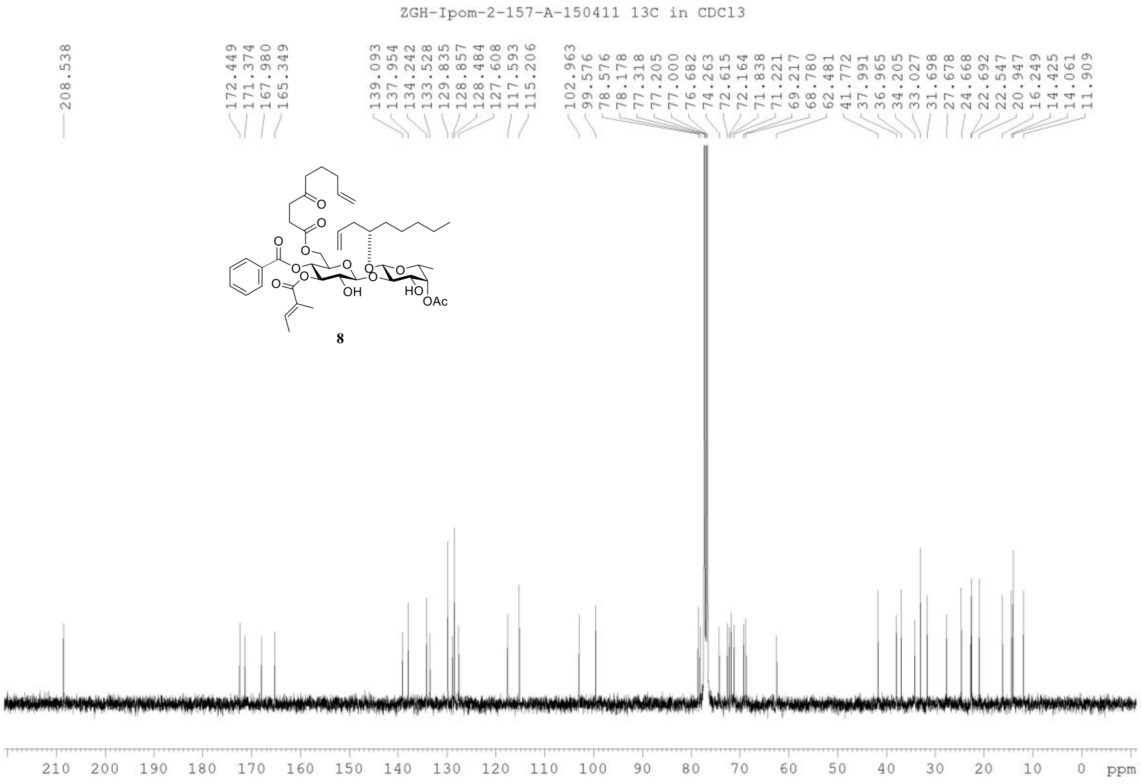

COSY NMR spectrum of **8** ($CDCl_3$, 400 MHz)

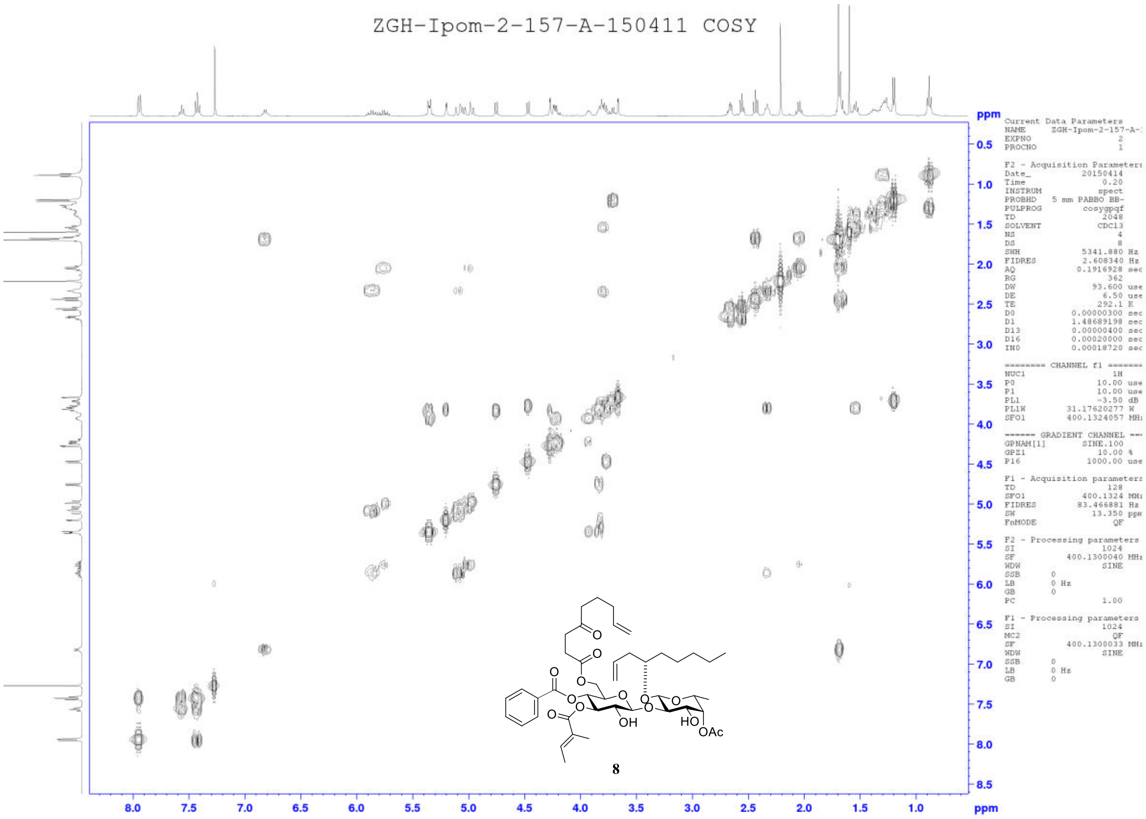

HSQC NMR spectrum of **8** ($CDCl_3$, 400 MHz)

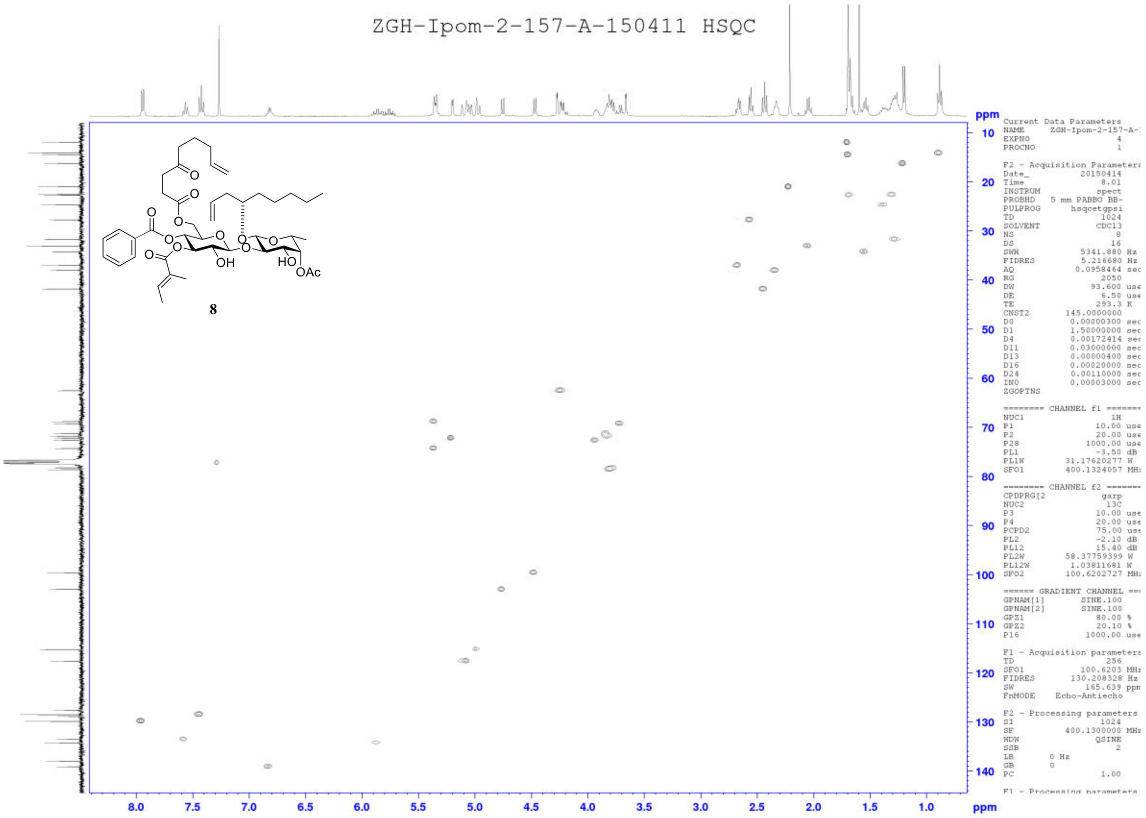

HMBC NMR spectrum of **8 (ZIF-20)** ($CDCl_3$, 400 MHz)

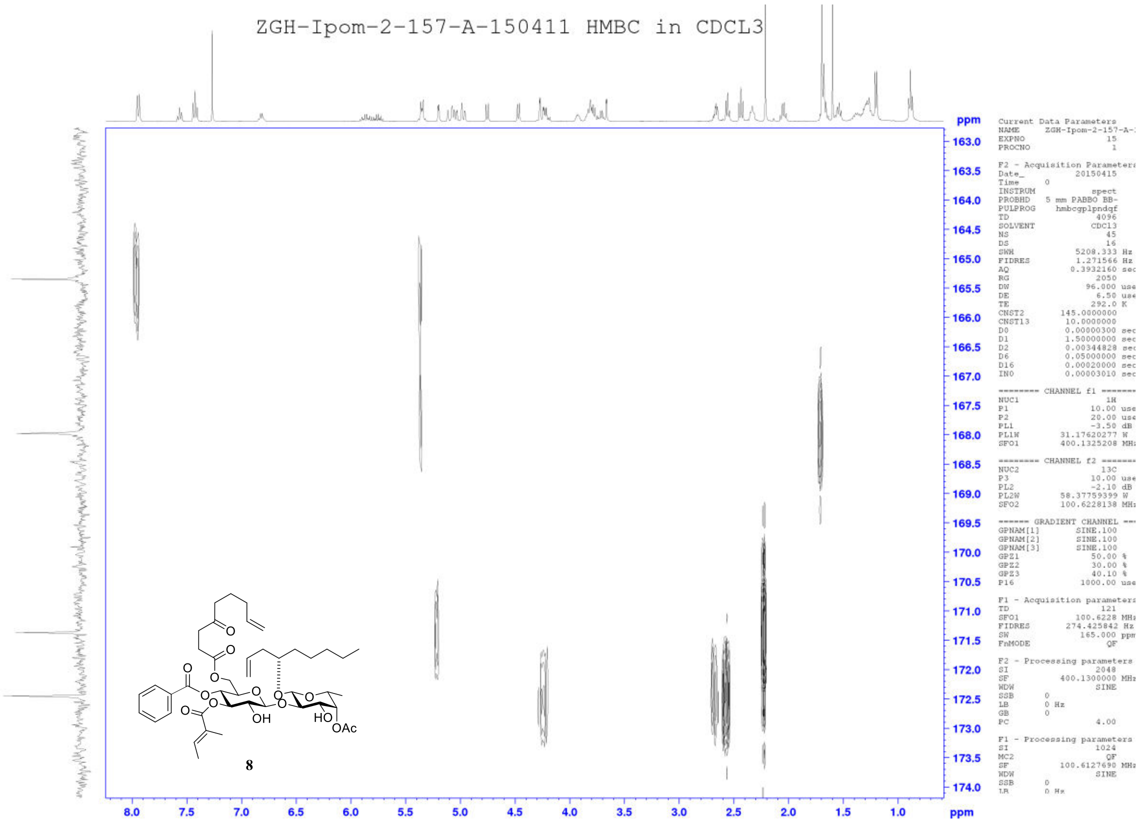

^{1}H NMR spectrum of **9** ($CDCl_3$, 400 MHz)

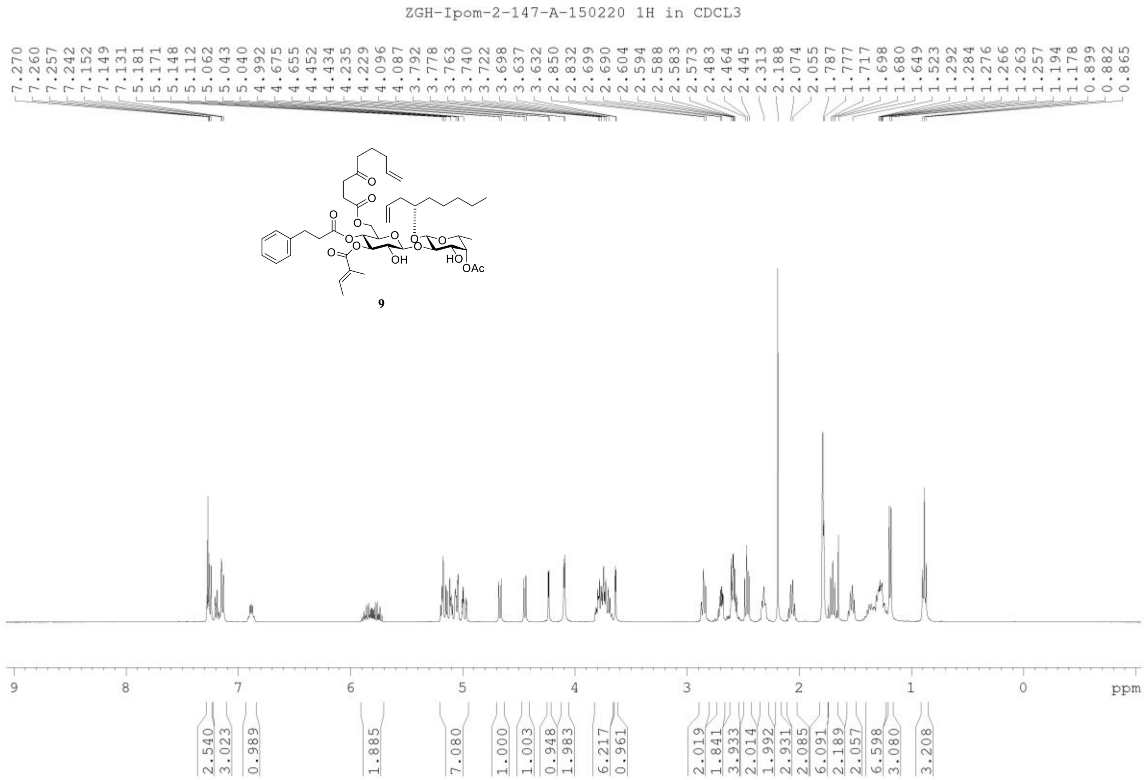

^{13}C NMR spectrum of **9** ($CDCl_3$, 400 MHz)

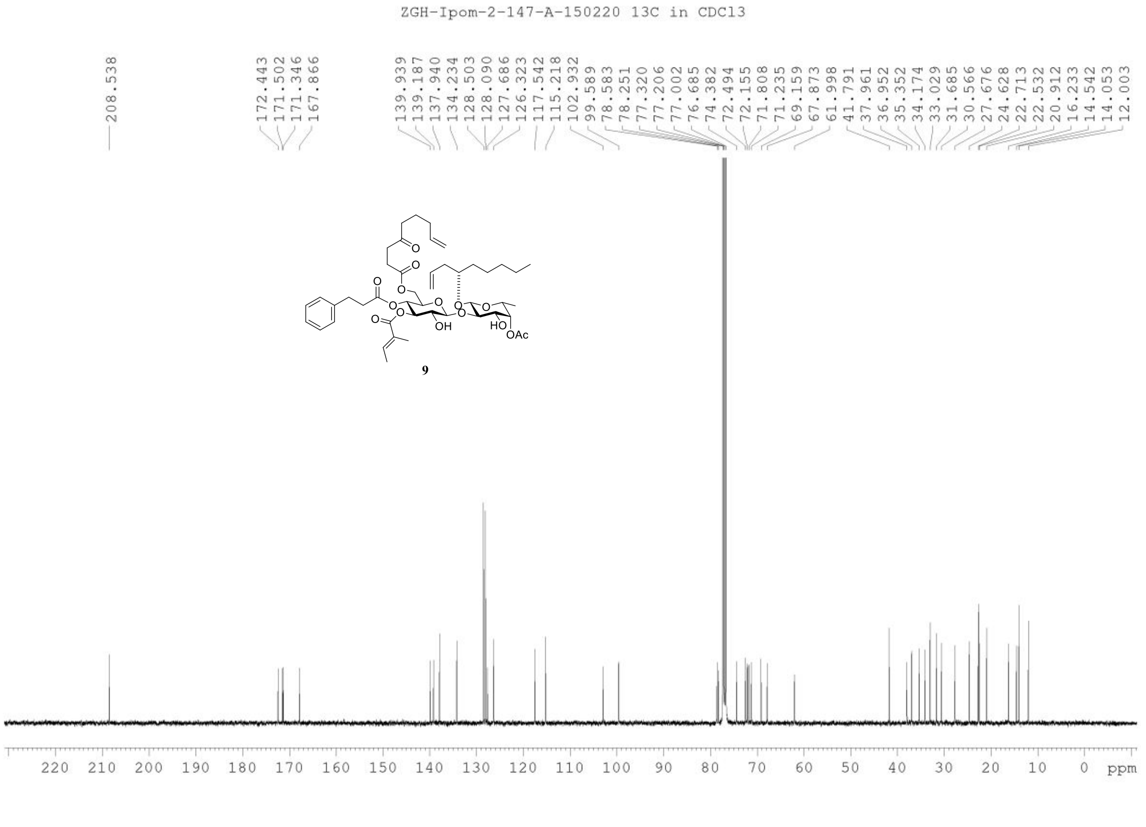

COSY NMR spectrum of **9** ($CDCl_3$, 400 MHz)

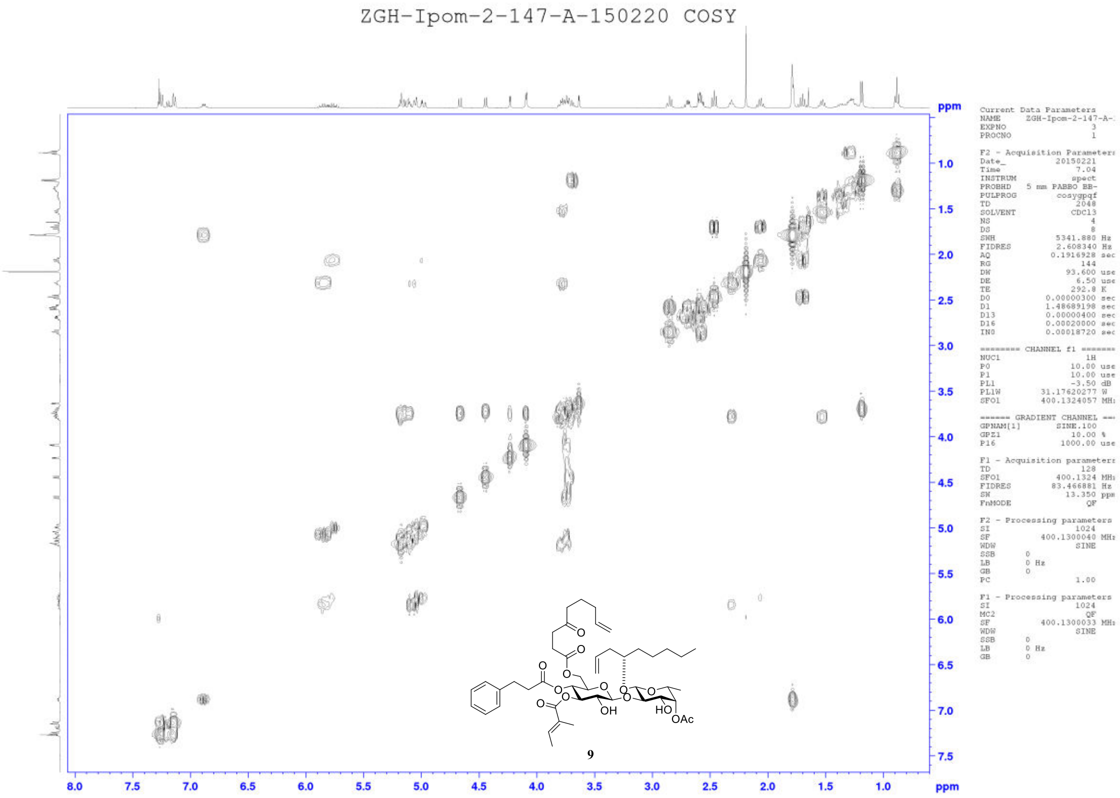

HSQC NMR spectrum of **9** ($CDCl_3$, 400 MHz)

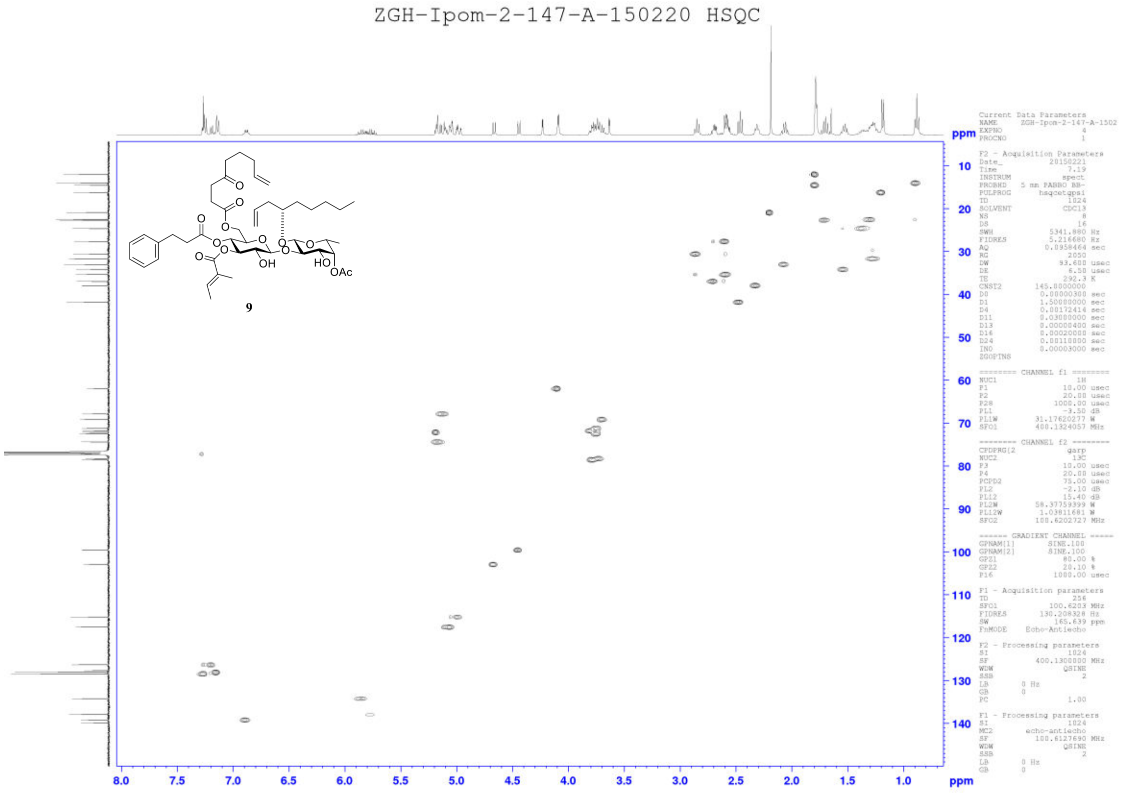

HMBC NMR spectrum of **9** ($CDCl_3$, 400 MHz)

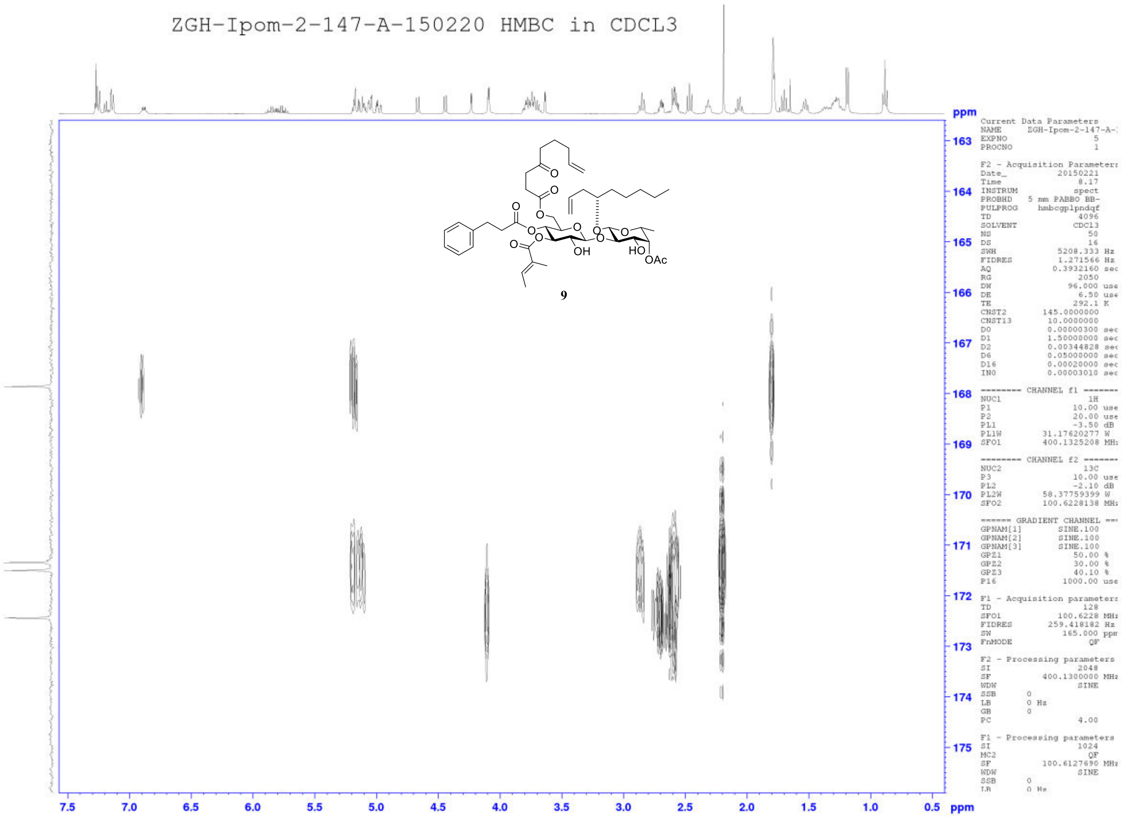

^{1}H NMR spectrum of **10** ($CDCl_3$, 400 MHz)

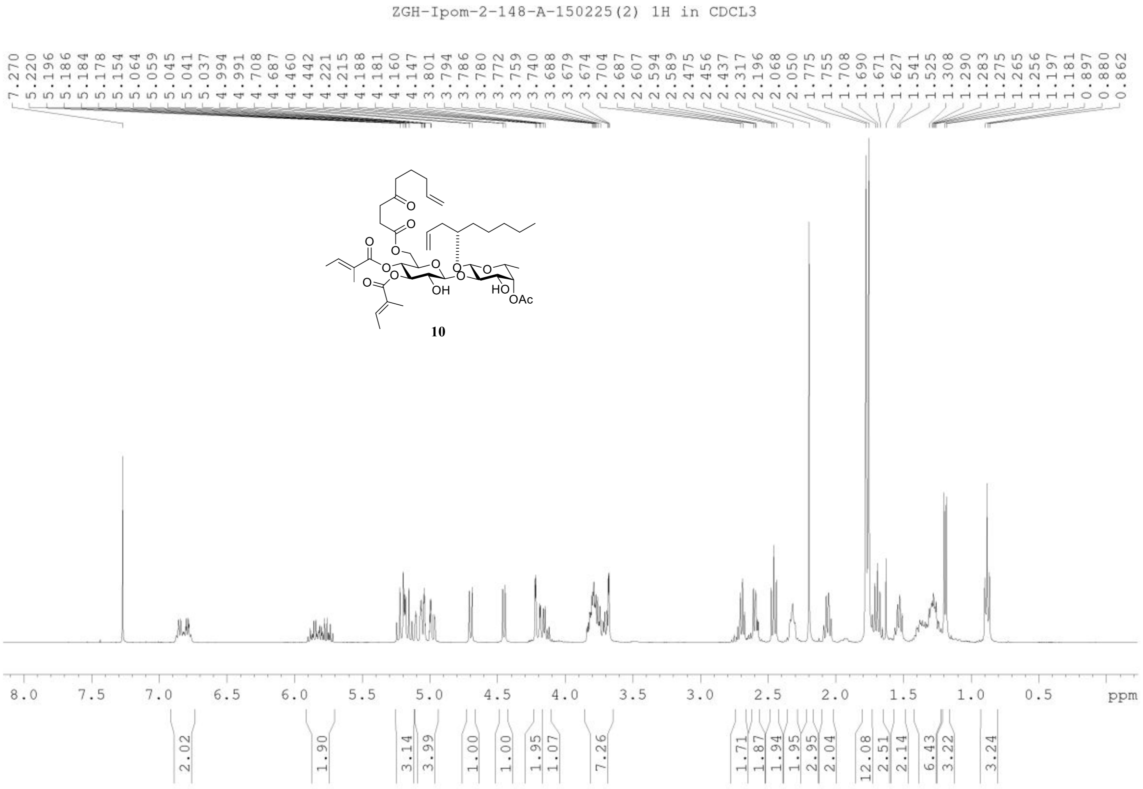

^{13}C NMR spectrum of **10** ($CDCl_3$, 400 MHz)

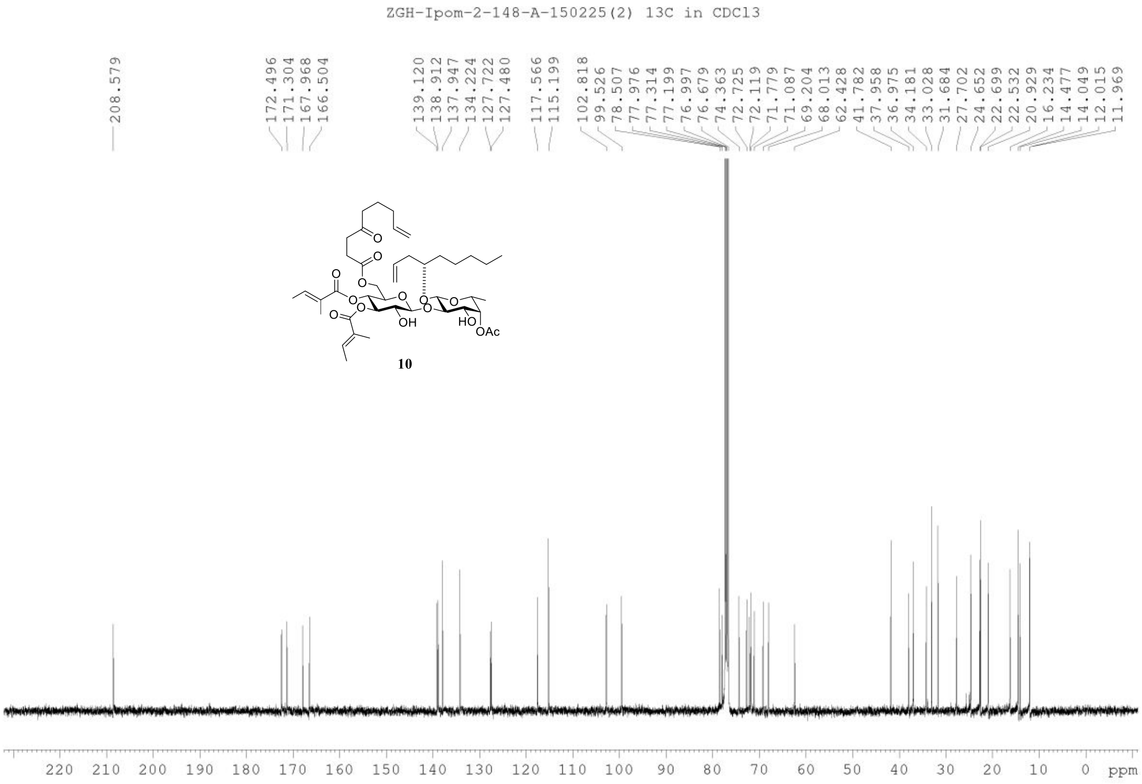

COSY NMR spectrum of **10** ($CDCl_3$, 400 MHz)

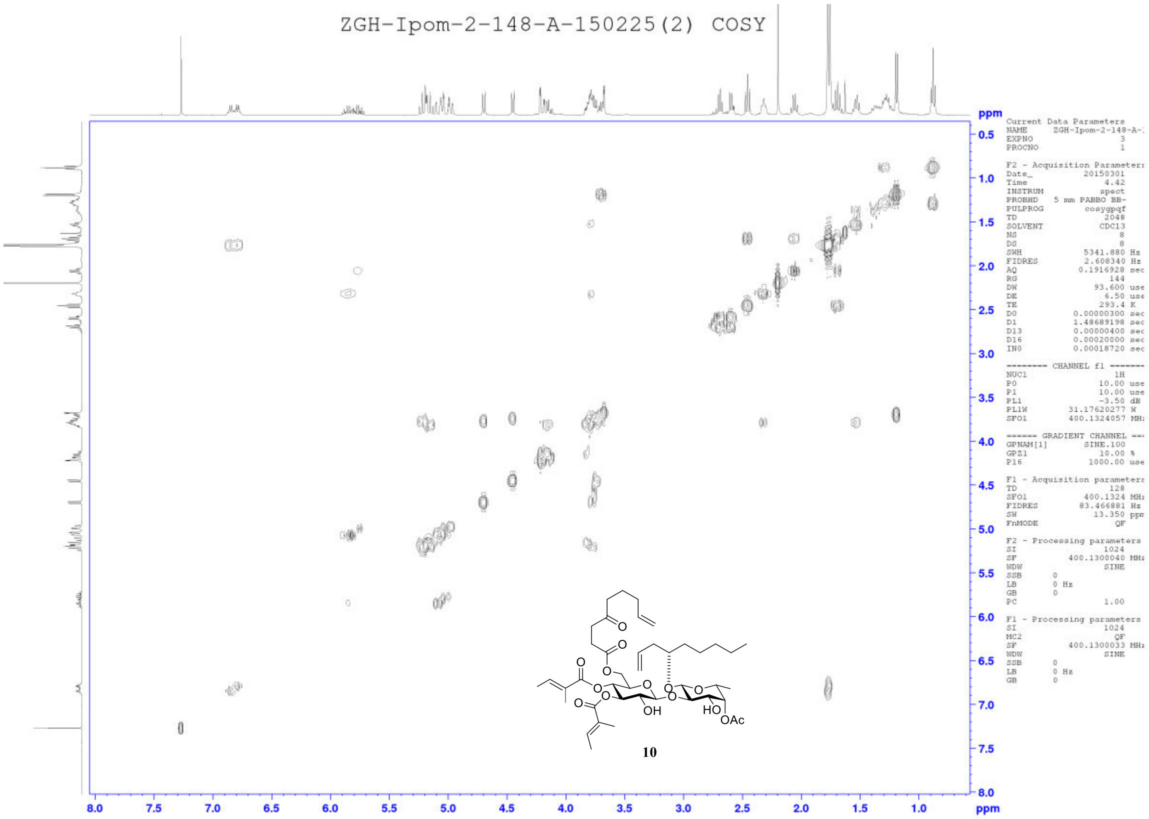

HSQC NMR spectrum of **10** ($CDCl_3$, 400 MHz)

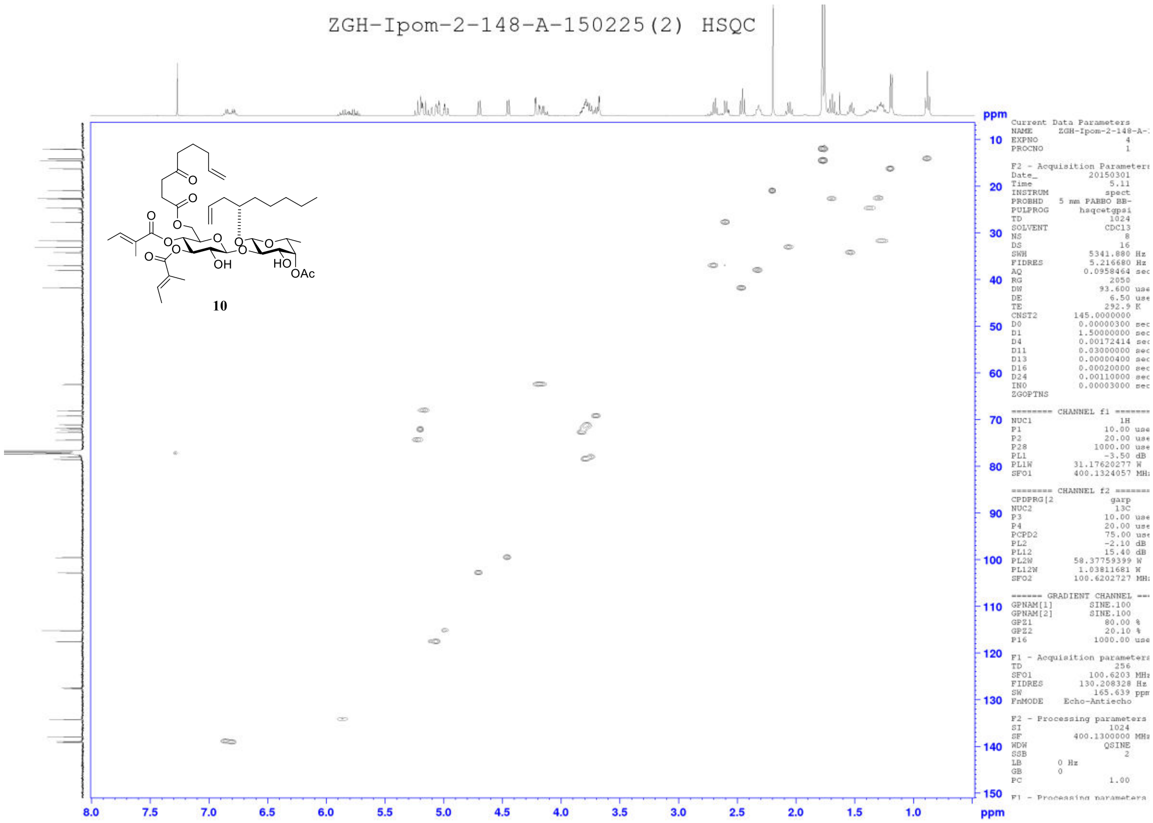

HMBC NMR spectrum of **10** ($CDCl_3$, 400 MHz)

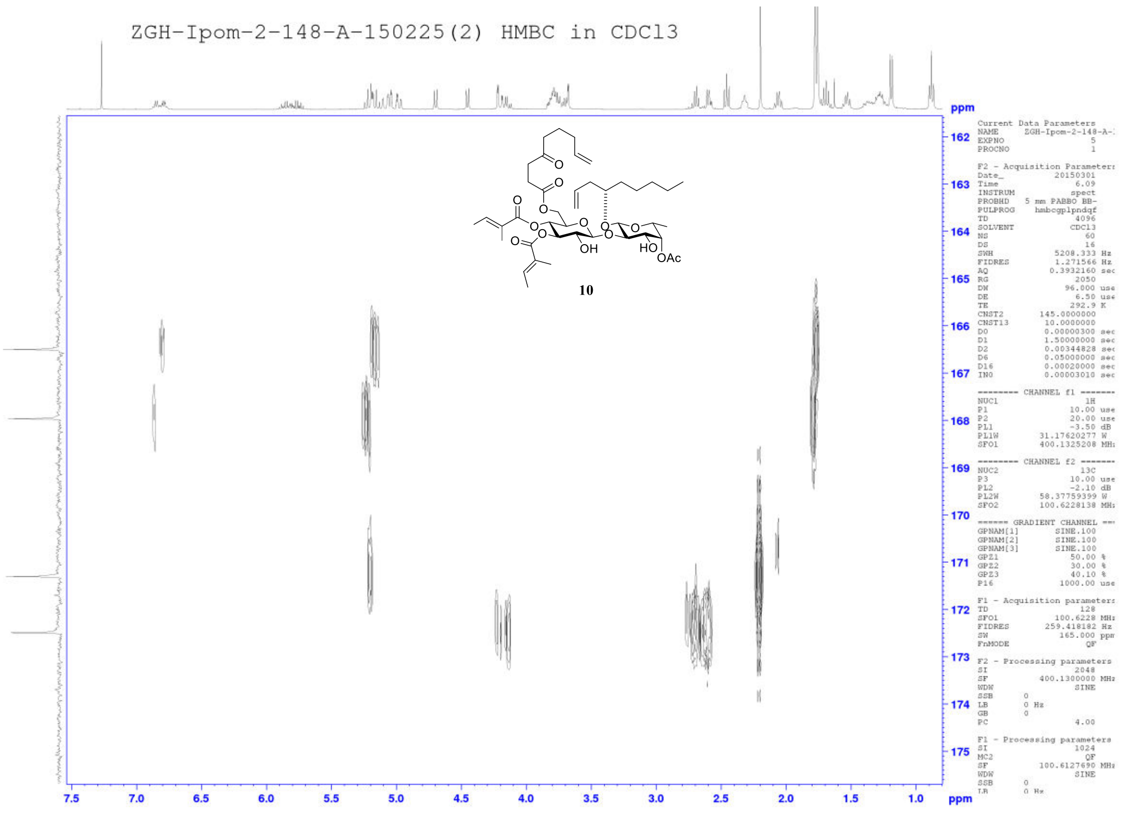

^{1}H NMR spectrum of **11** ($CDCl_3$, 400 MHz)

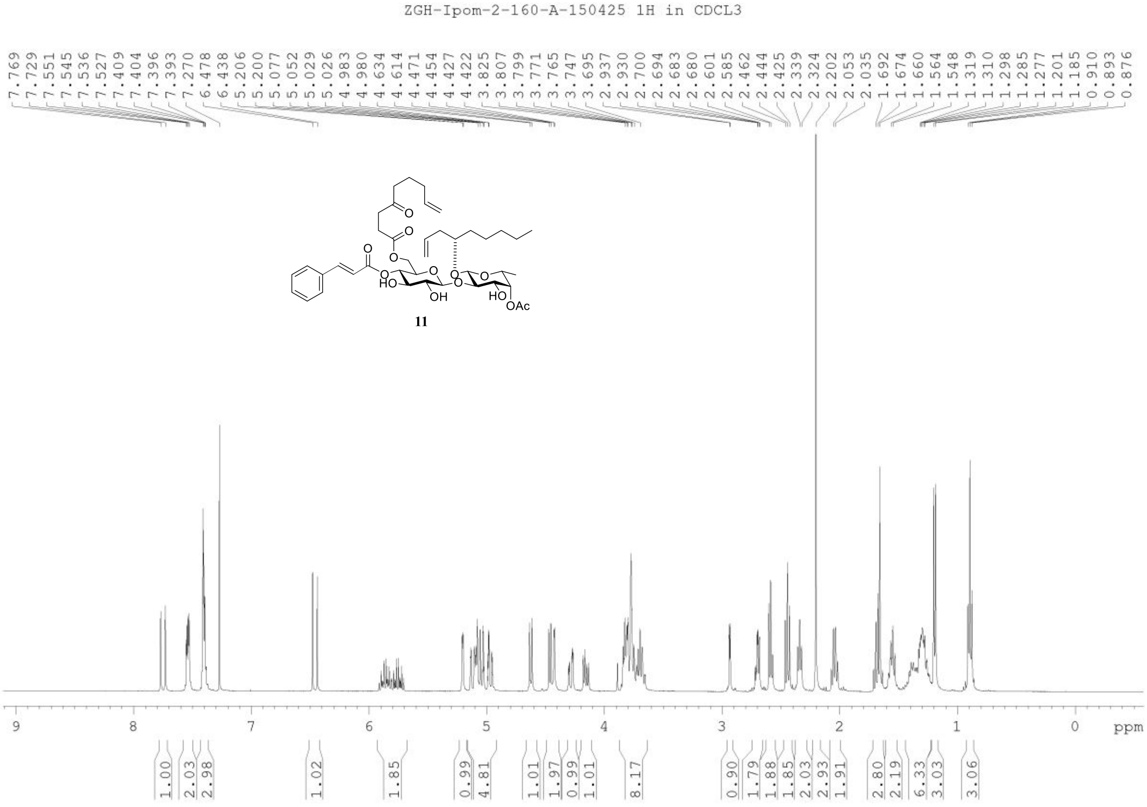

^{13}C NMR spectrum of **11** ($CDCl_3$, 400 MHz)

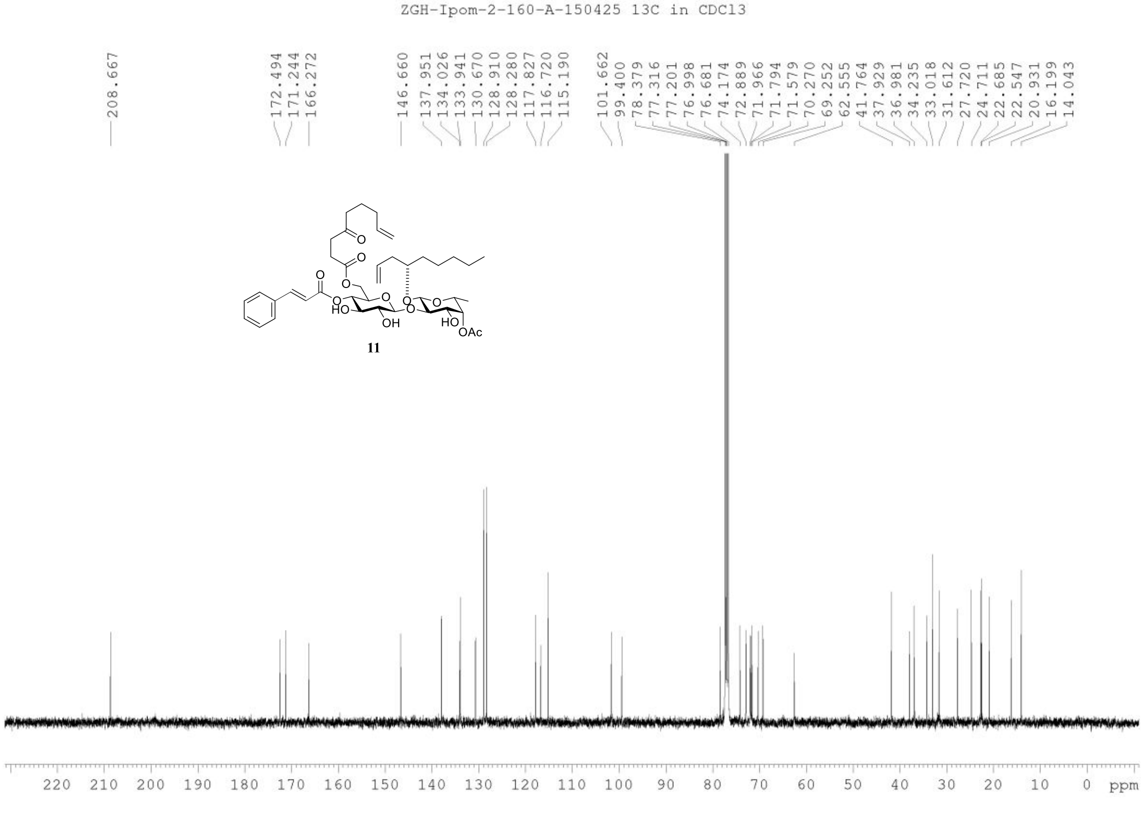

COSY NMR spectrum of **11** ($CDCl_3$, 400 MHz)

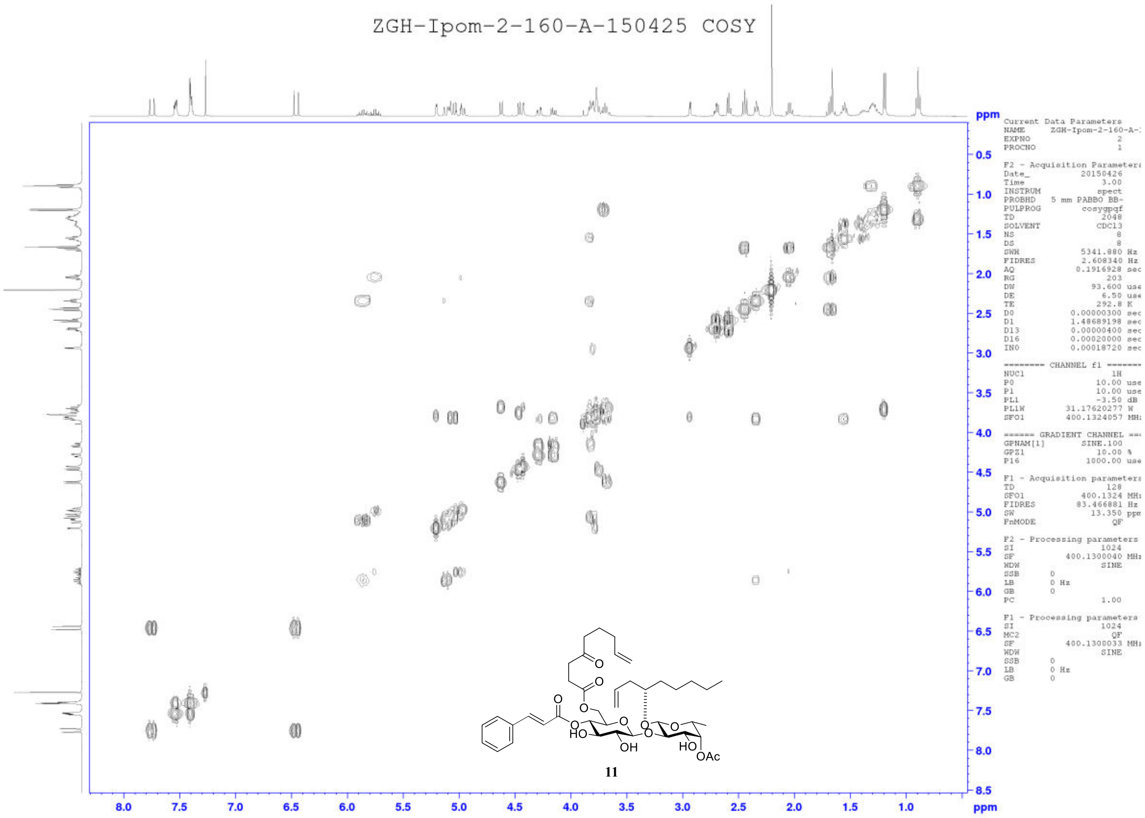

HSQC NMR spectrum of **11** ($CDCl_3$, 400 MHz)

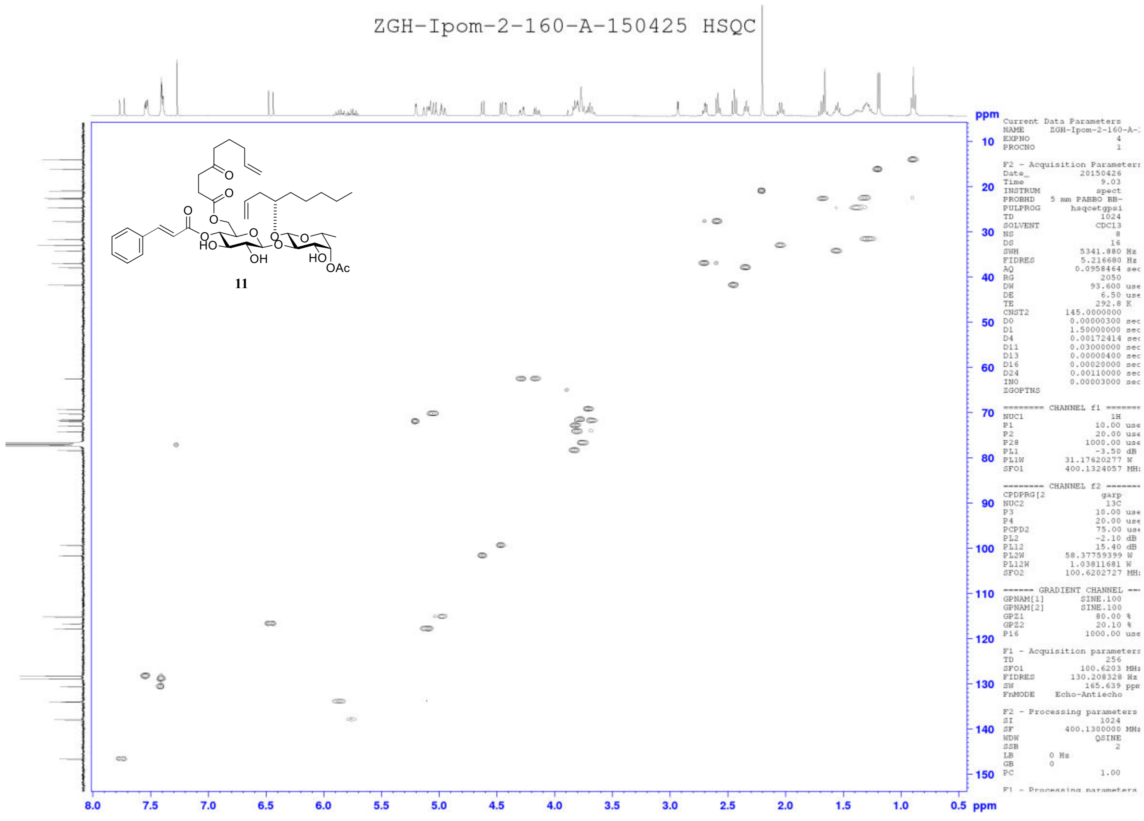

HMBC NMR spectrum of **11** ($CDCl_3$, 400 MHz)

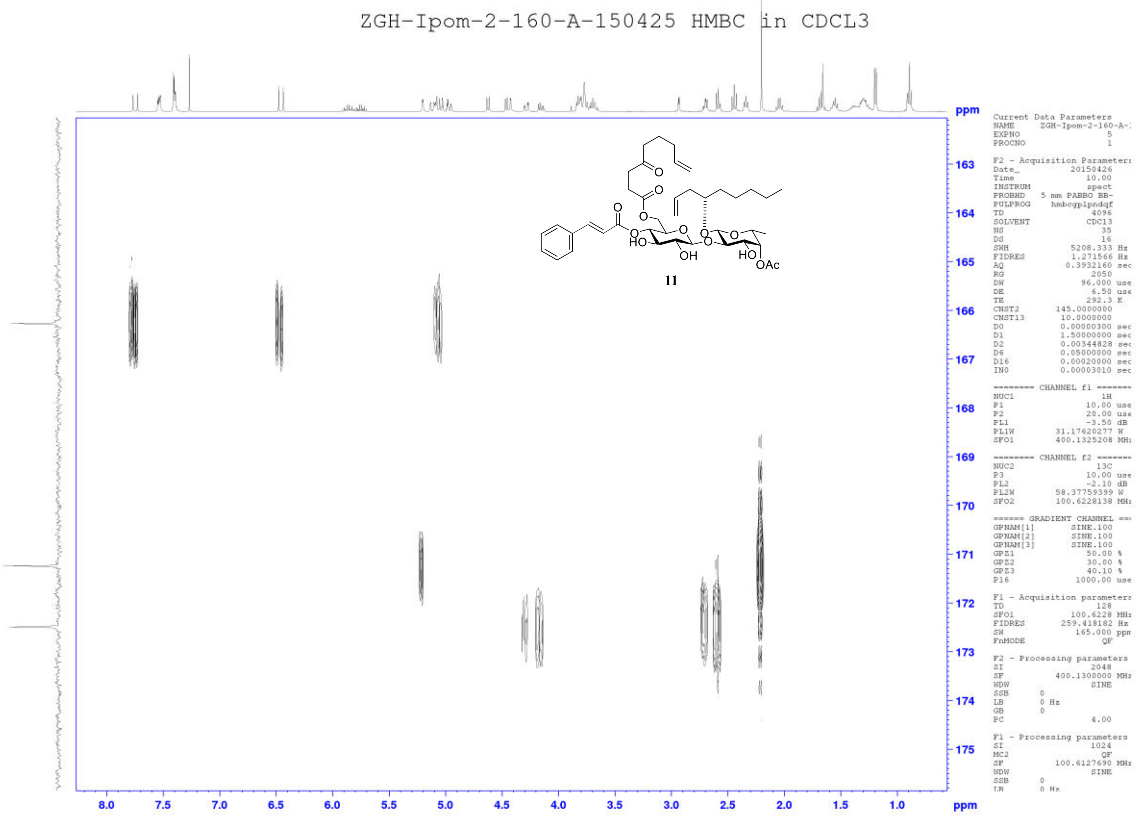

^{1}H NMR spectrum of **12** ($CDCl_3$, 400 MHz)

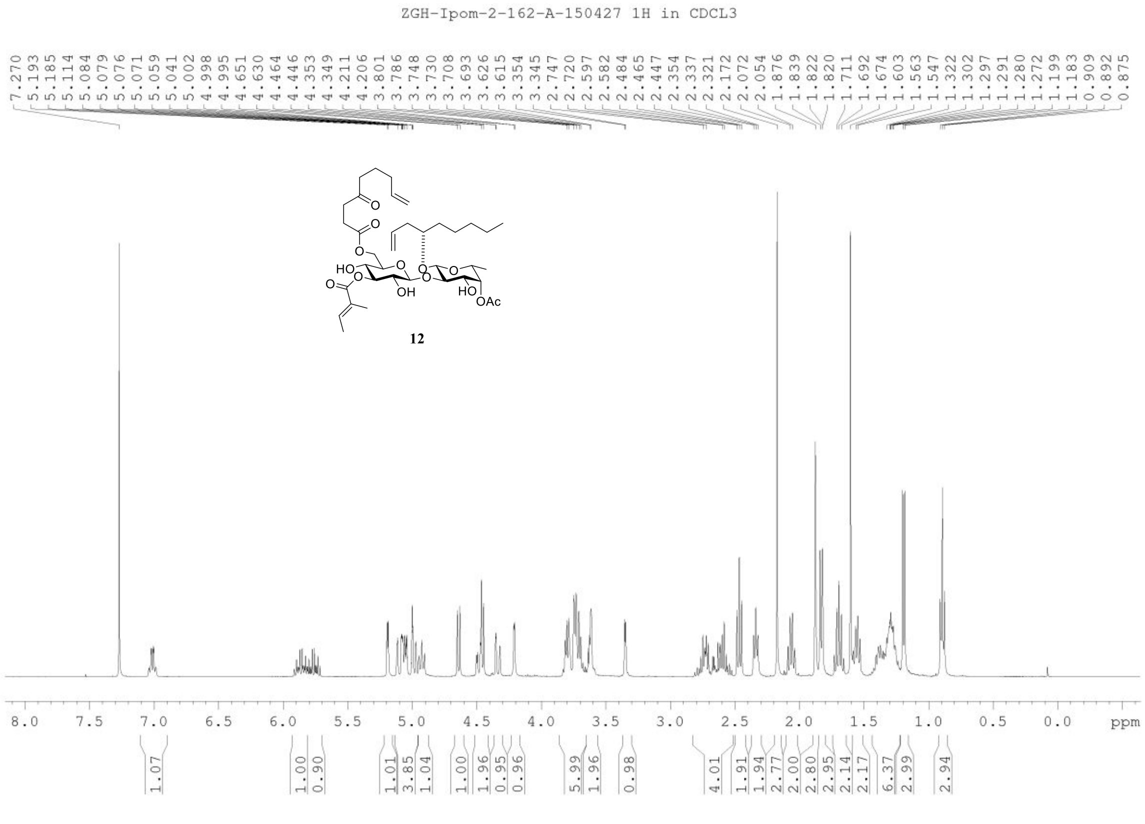

^{13}C NMR spectrum of **12** ($CDCl_3$, 400 MHz)

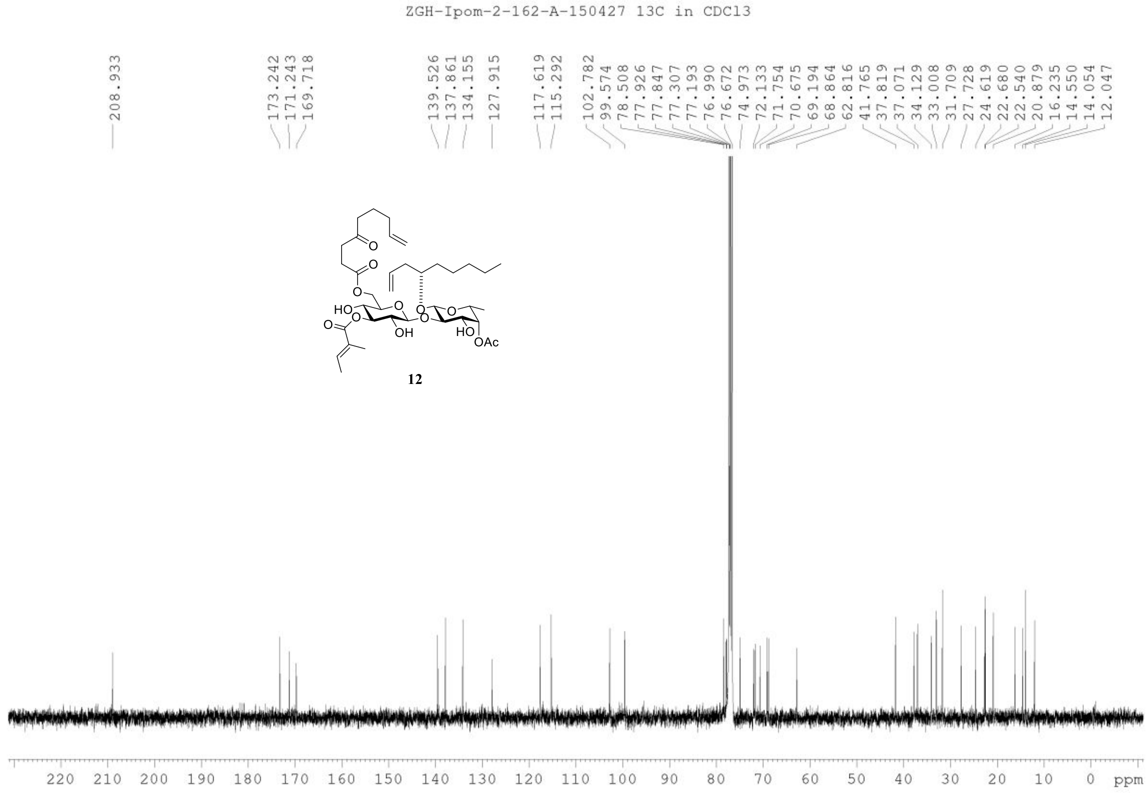

COSY NMR spectrum of **12** ($CDCl_3$, 400 MHz)

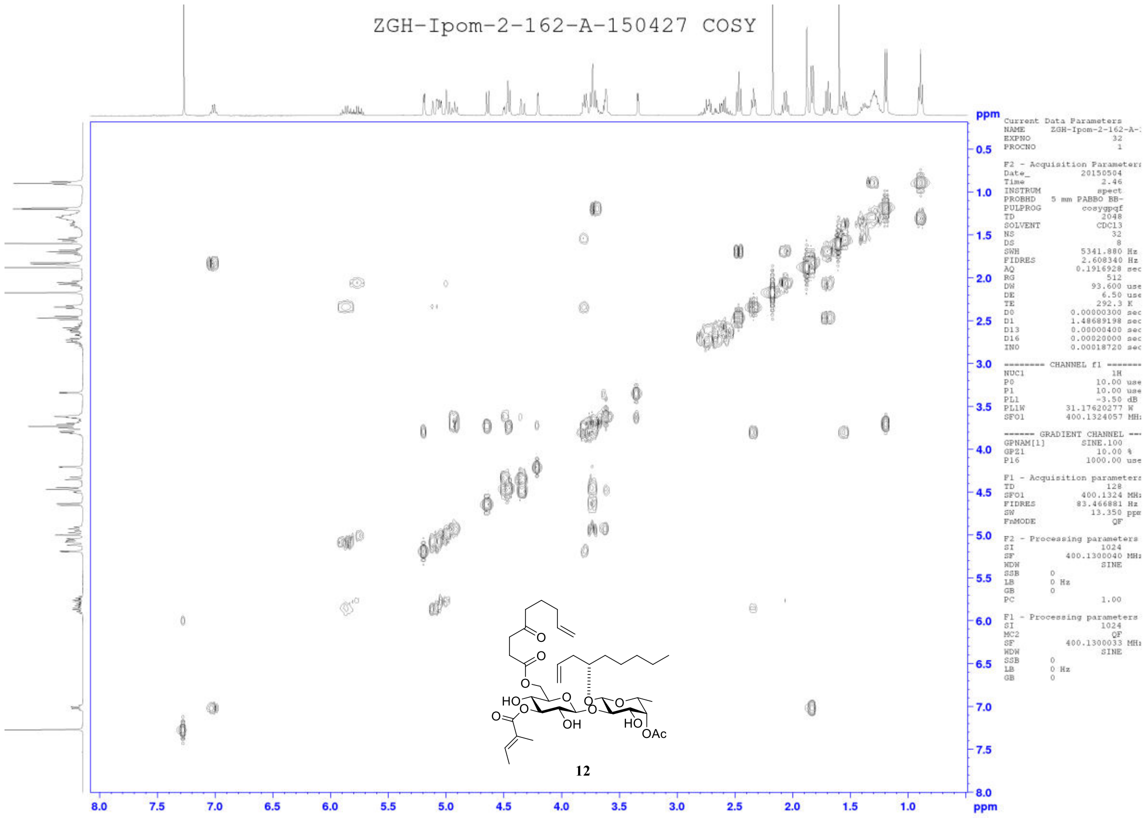

HSQC NMR spectrum of **12** ($CDCl_3$, 400 MHz)

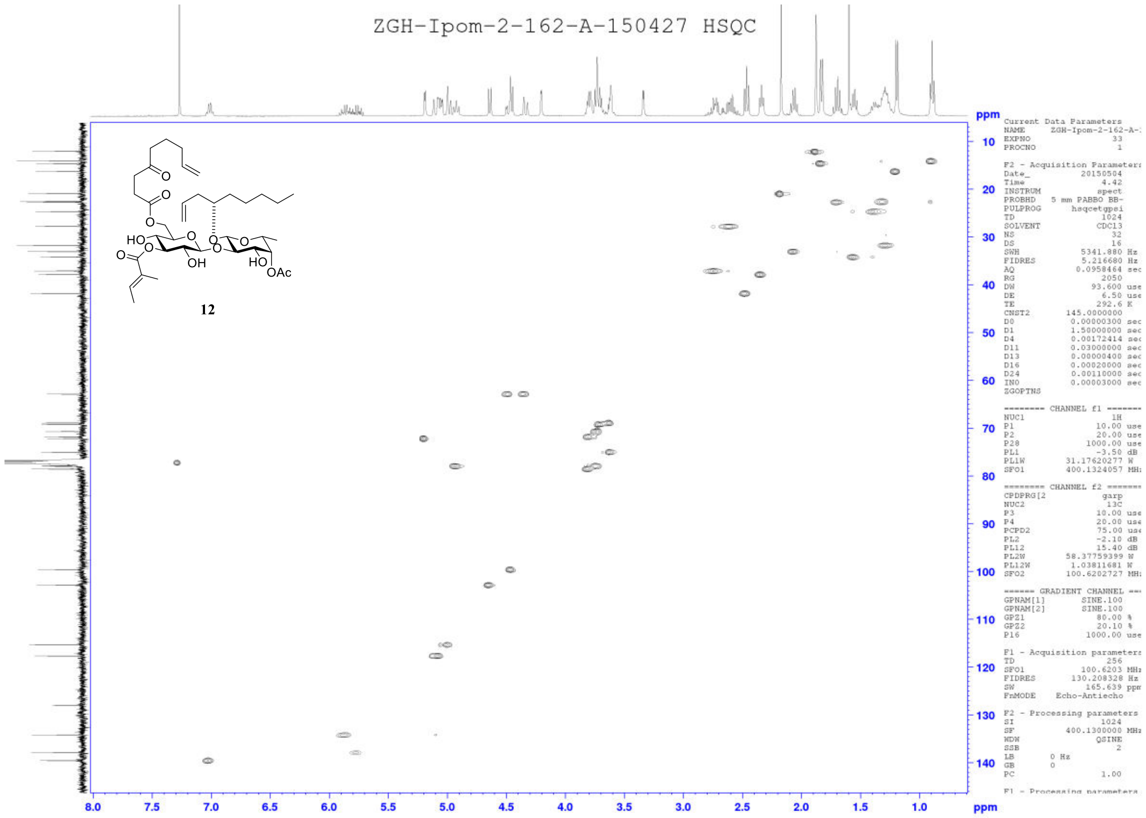

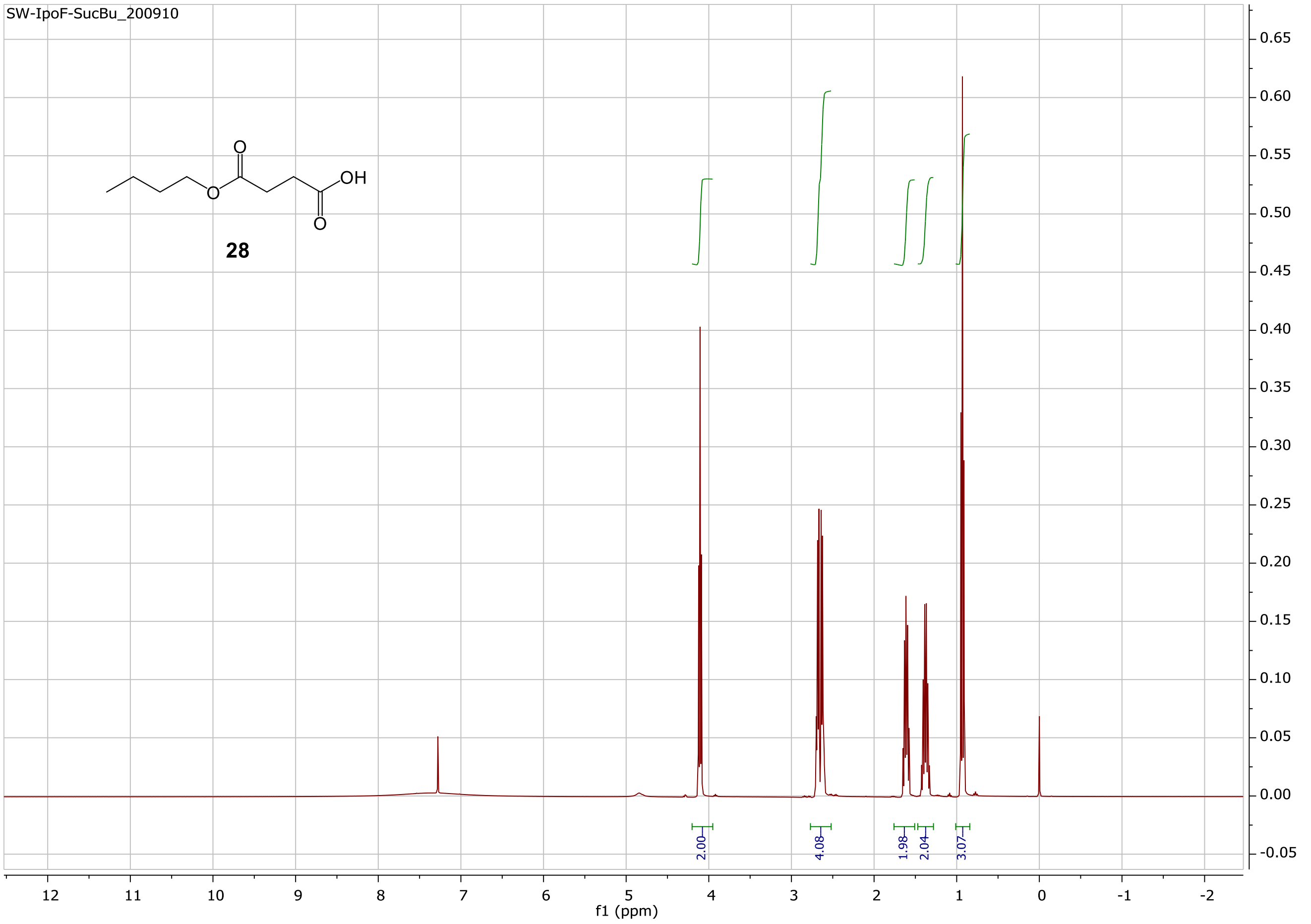
SW-IpoF-SucBu_200910
OH
O
O
O
28
2.00
4.08
1.98
2.04
3.07
f1 (ppm)

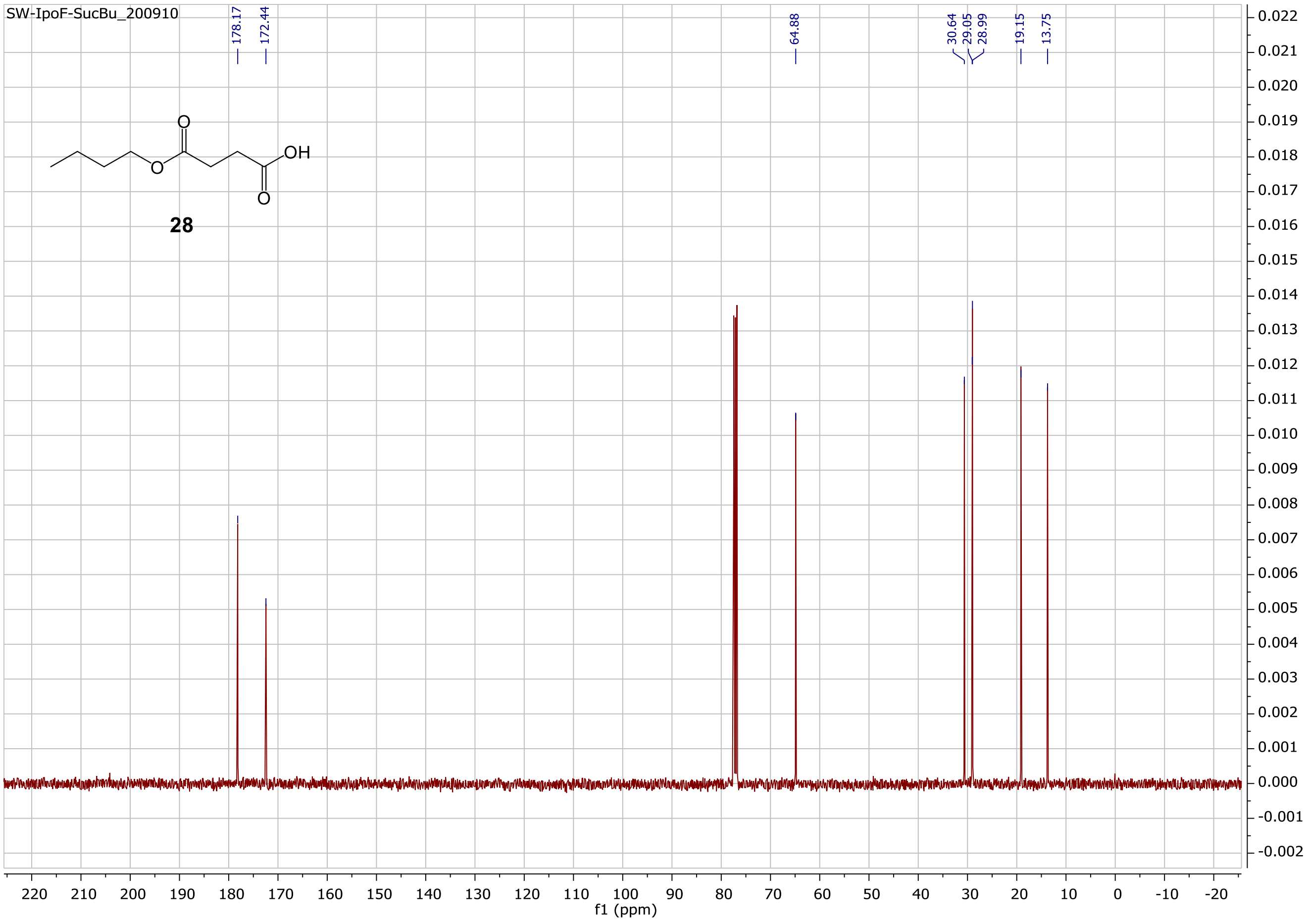
SW-IpoF-SucBu_200910
178.17
172.44
64.88
30.64
29.05
28.99
19.15
13.75
OH
O
O
O
28
f1 (ppm)

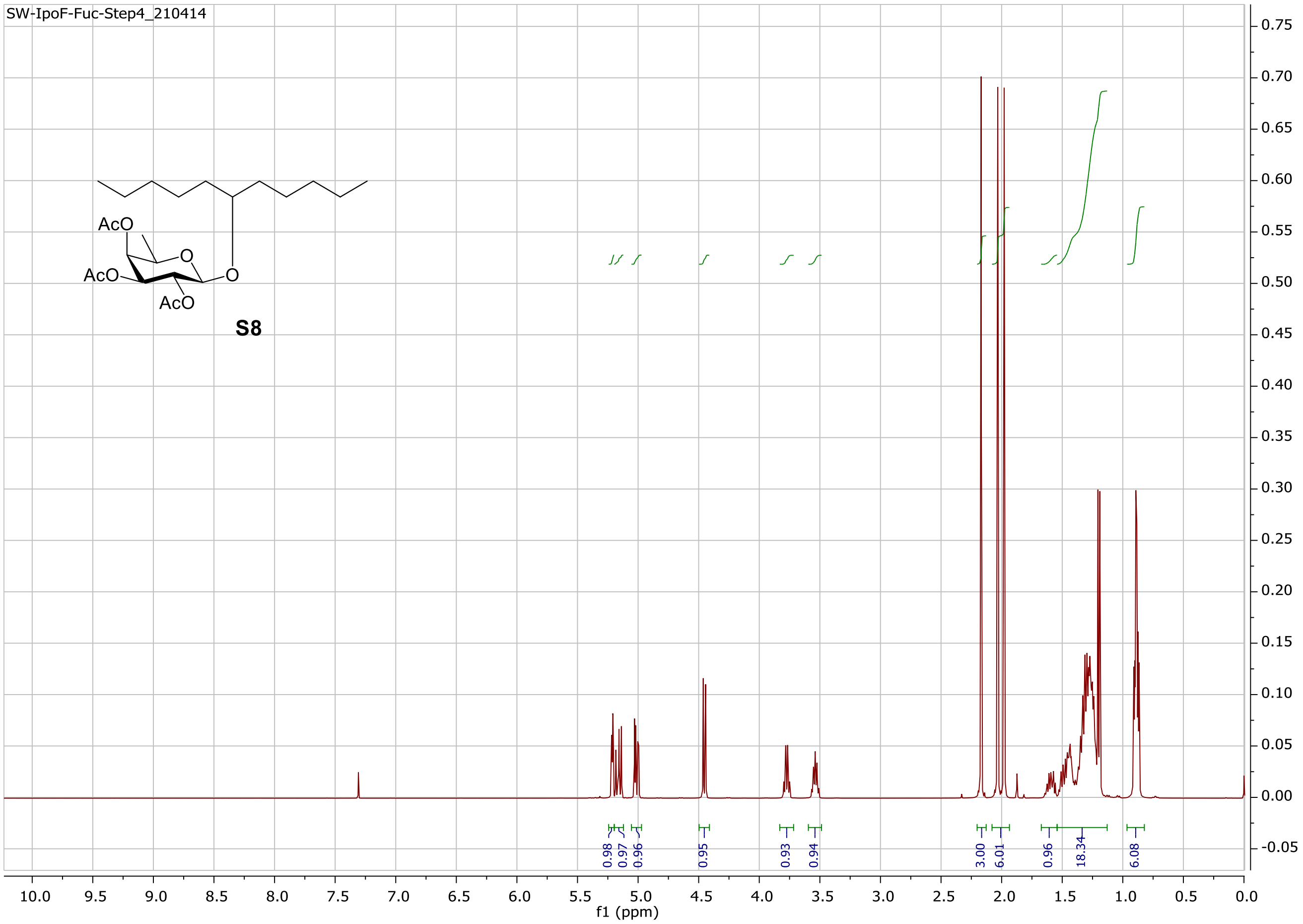
SW-IpoF-Fuc-Step4_210414
AcO
AcO
AcO
O
O
S8
0.98
0.97
0.96
0.95
0.93
0.94
3.00
6.01
0.96
18.34
6.08
f1 (ppm)

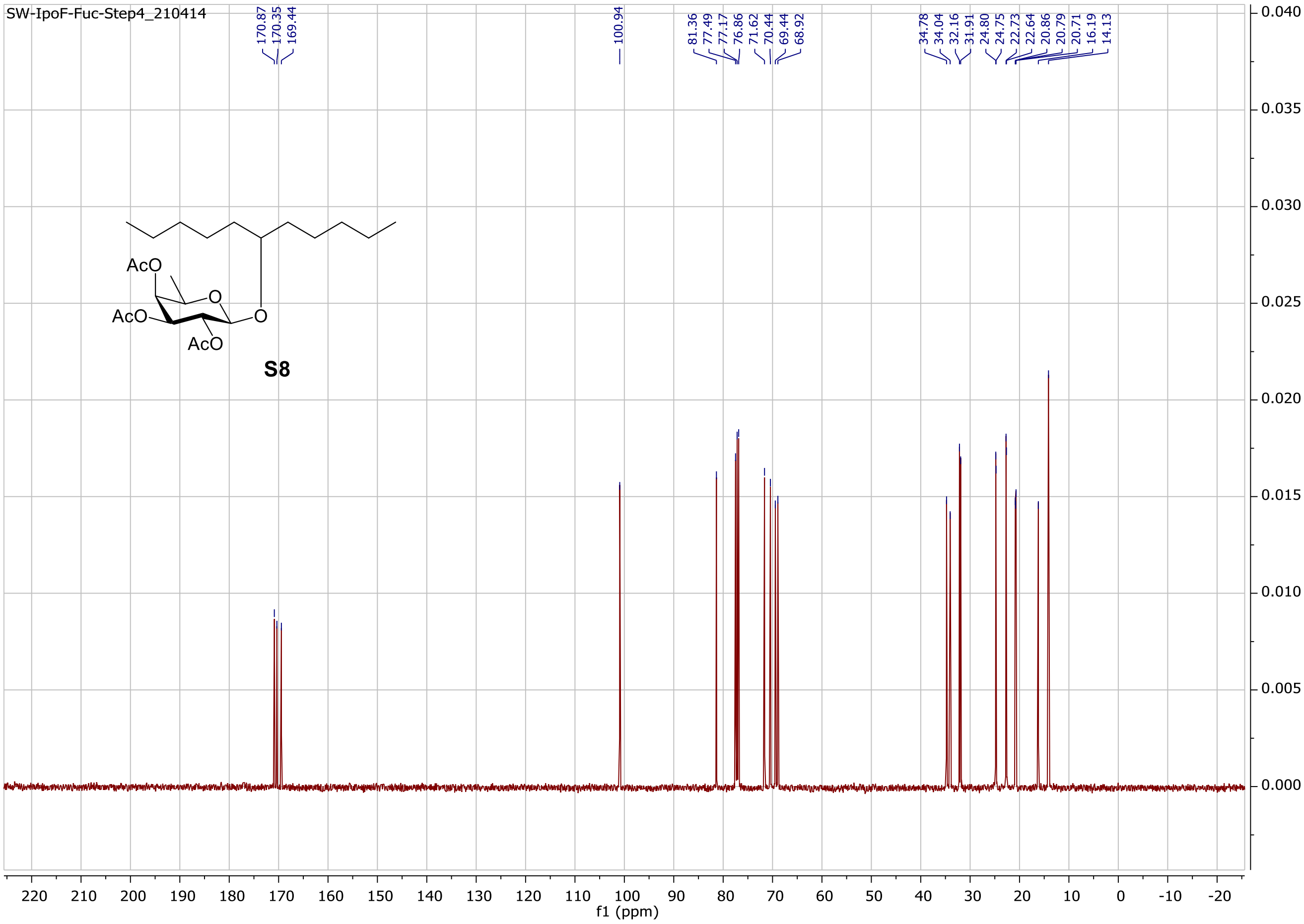
SW-IpoF-Fuc-Step4_210414
170.87
170.35
169.44
100.94
81.36
77.49
77.17
76.86
71.62
70.44
69.44
68.92
34.78
34.04
32.16
31.91
24.80
24.75
22.73
22.64
20.86
20.79
20.71
16.19
14.13
AcO
AcO
AcO
O
O
S8
f1 (ppm)

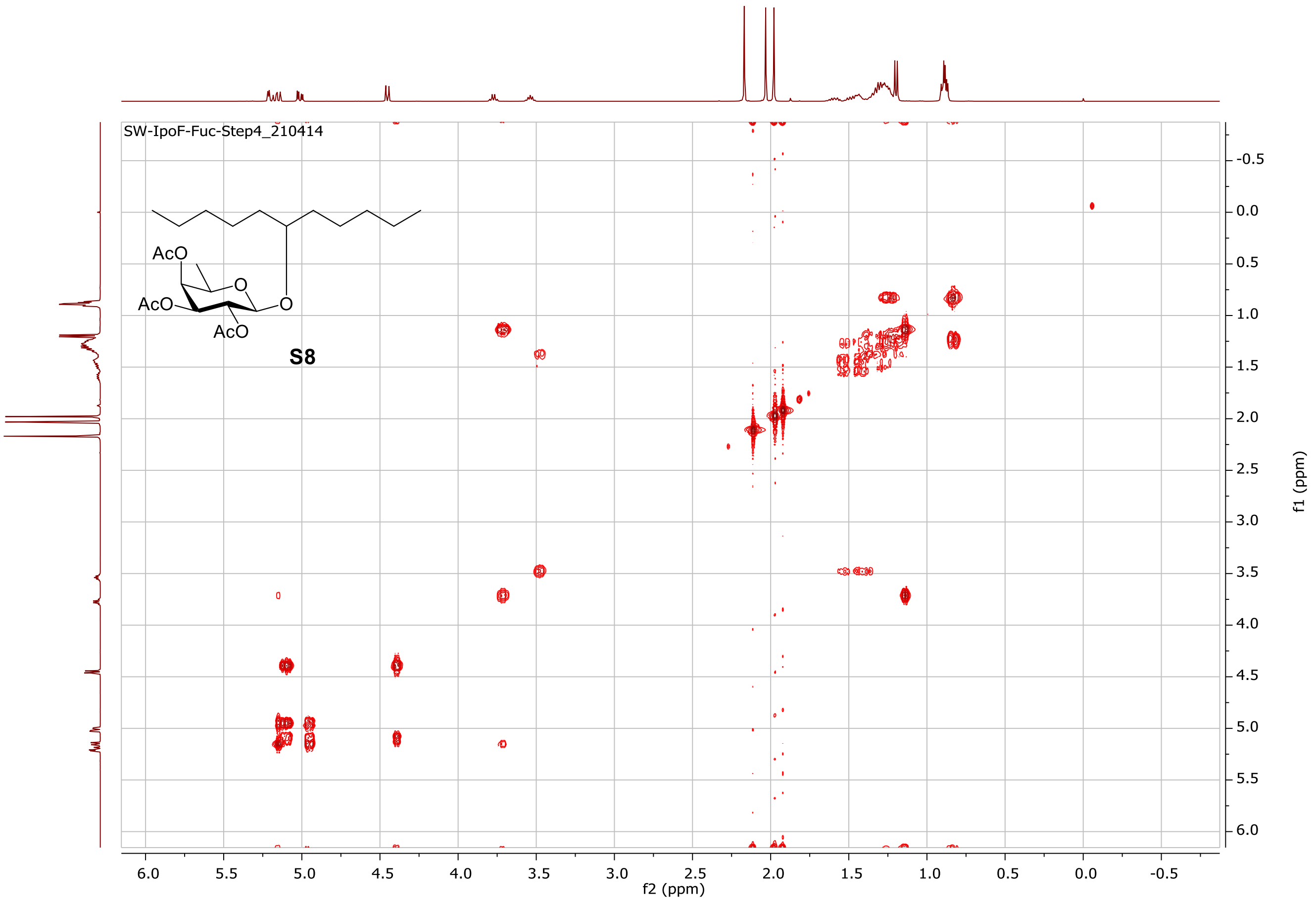
SW-IpoF-Fuc-Step4_210414
AcO
AcO
O
O
AcO
S8
f2 (ppm)
f1 (ppm)

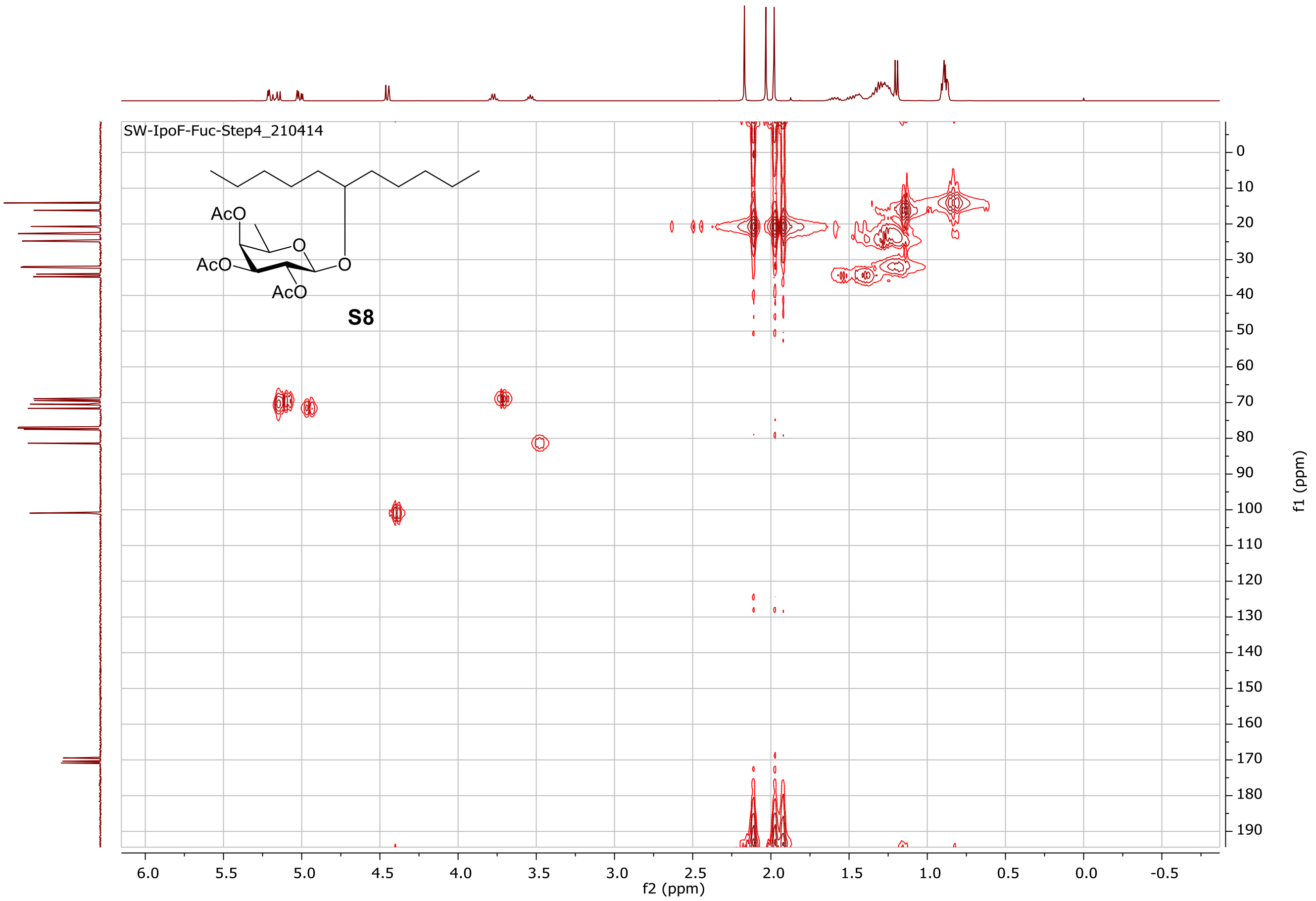
SW-IpoF-Fuc-Step4_210414
AcO
AcO
O
O
AcO
S8
f2 (ppm)
f1 (ppm)

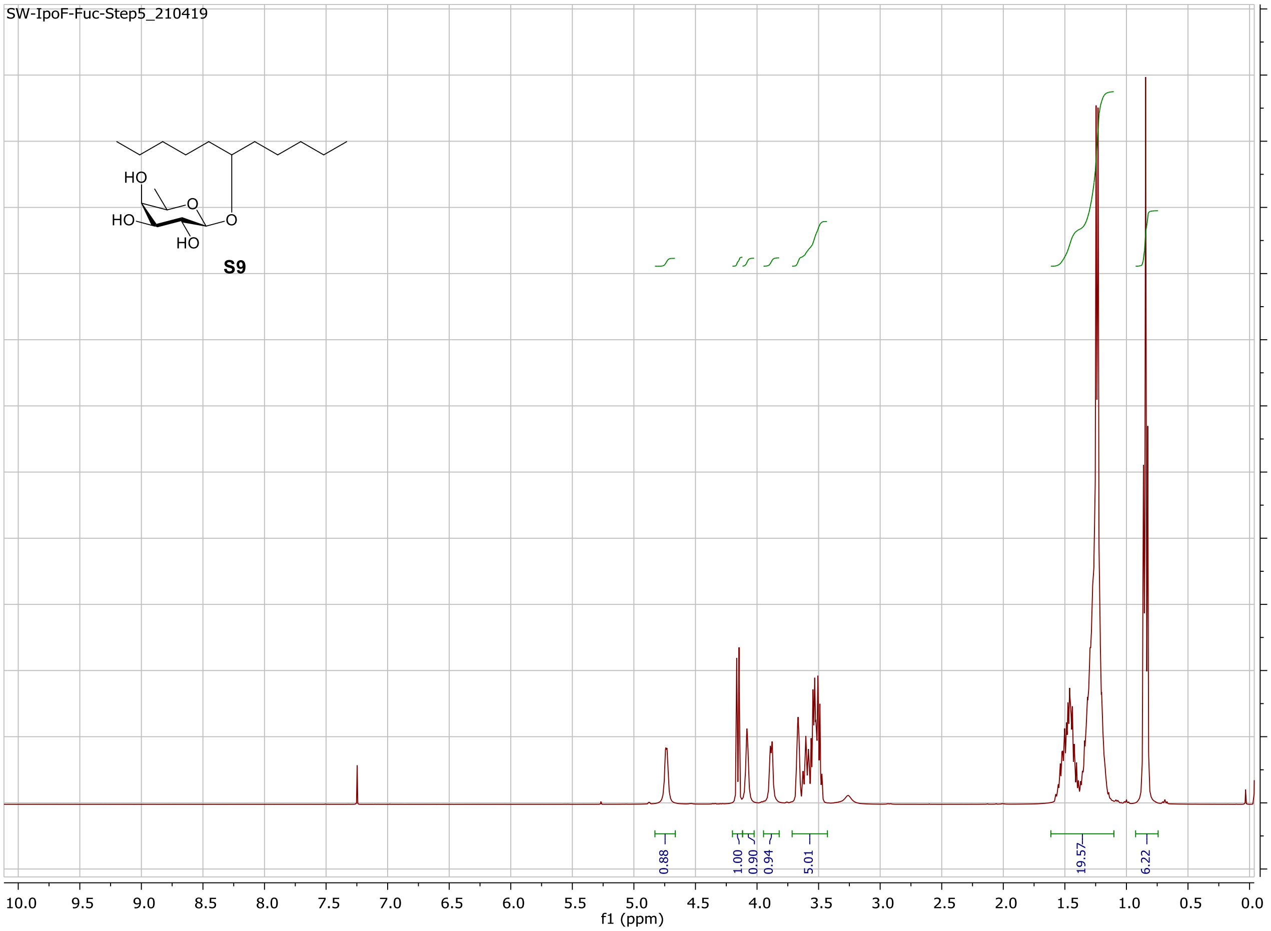
SW-IpoF-Fuc-Step5_210419
HO
HO
O
O
HO
S9
0.88
1.00
0.90
0.94
5.01
19.57
6.22
f1 (ppm)

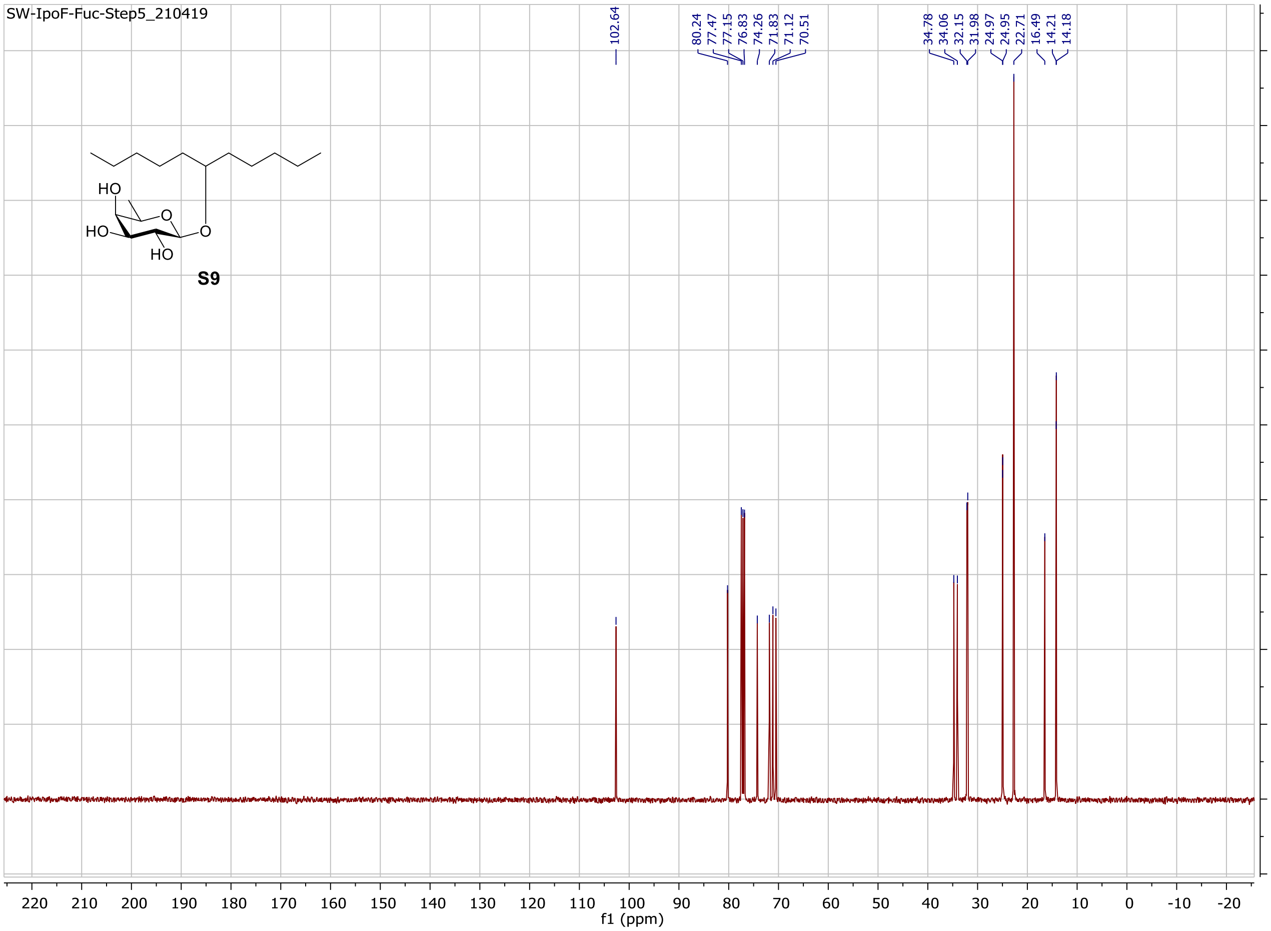
SW-IpoF-Fuc-Step5_210419
102.64
80.24
77.47
77.15
76.83
74.26
71.83
71.12
70.51
34.78
34.06
32.15
31.98
24.97
24.95
22.71
16.49
14.21
14.18
HO
HO
O
O
HO
S9
f1 (ppm)

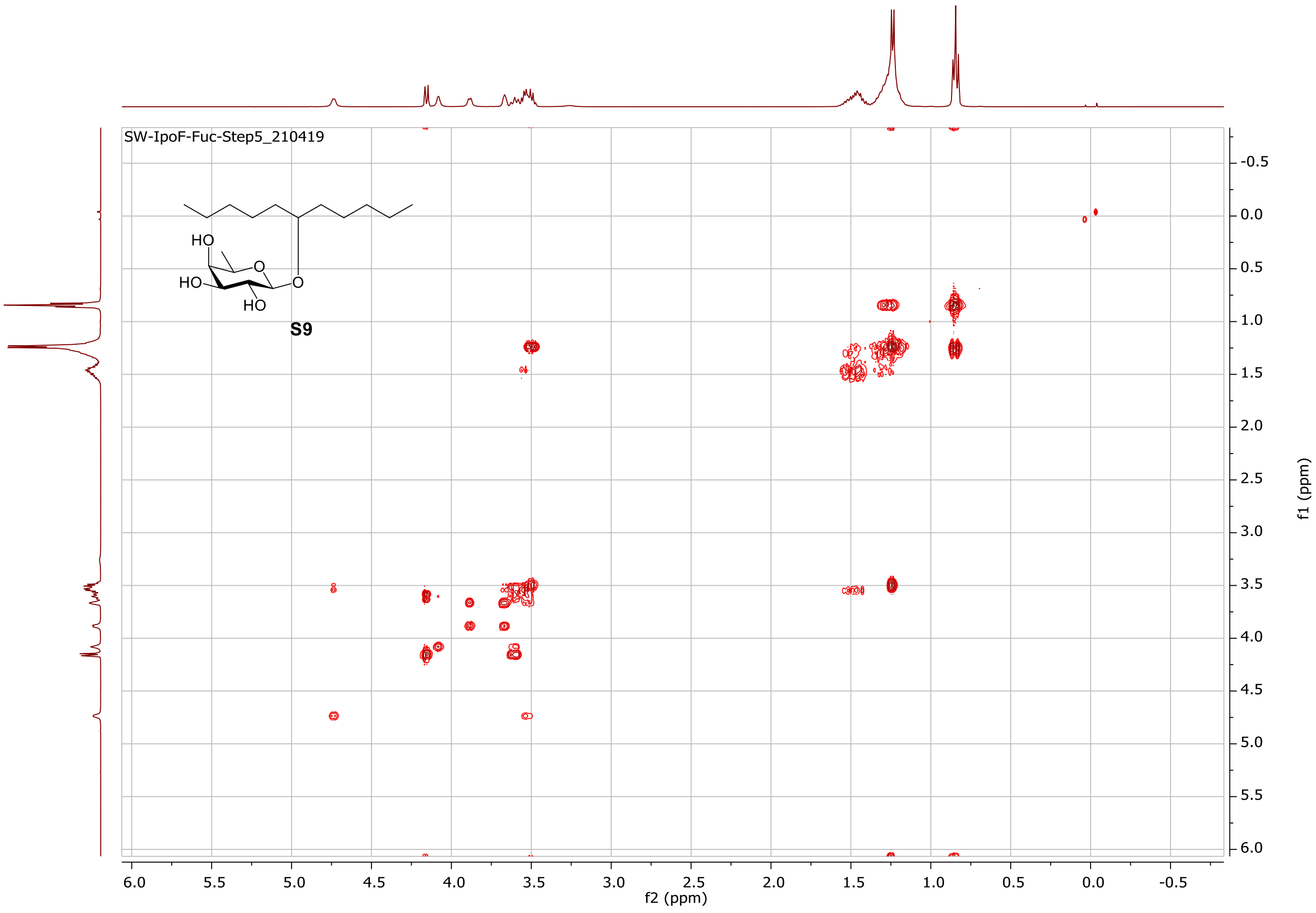
SW-IpoF-Fuc-Step5_210419
HO
HO
HO
O
O
S9
f2 (ppm)
f1 (ppm)

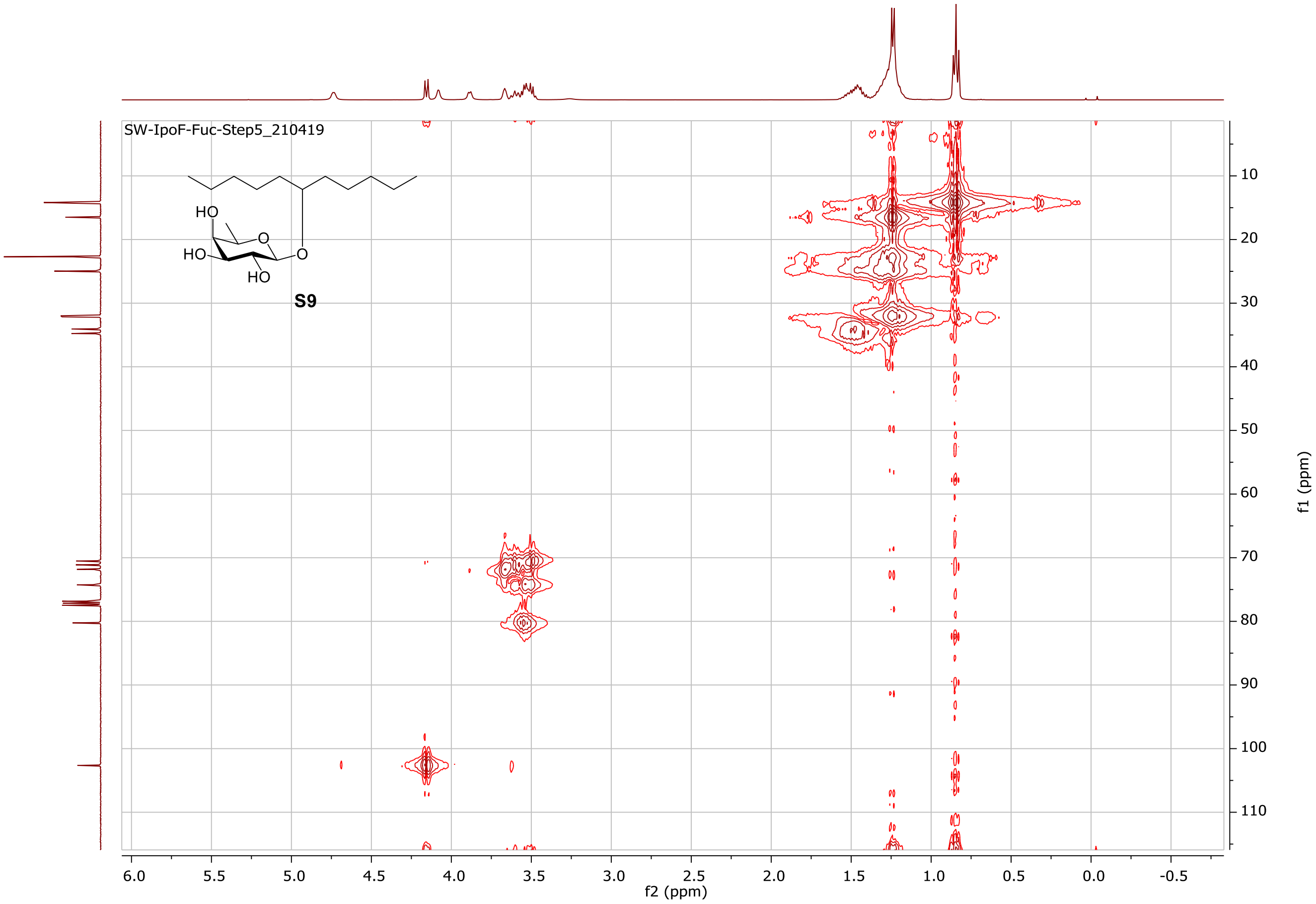
SW-IpoF-Fuc-Step5_210419
HO
HO
HO
O
O
S9
f2 (ppm)
f1 (ppm)

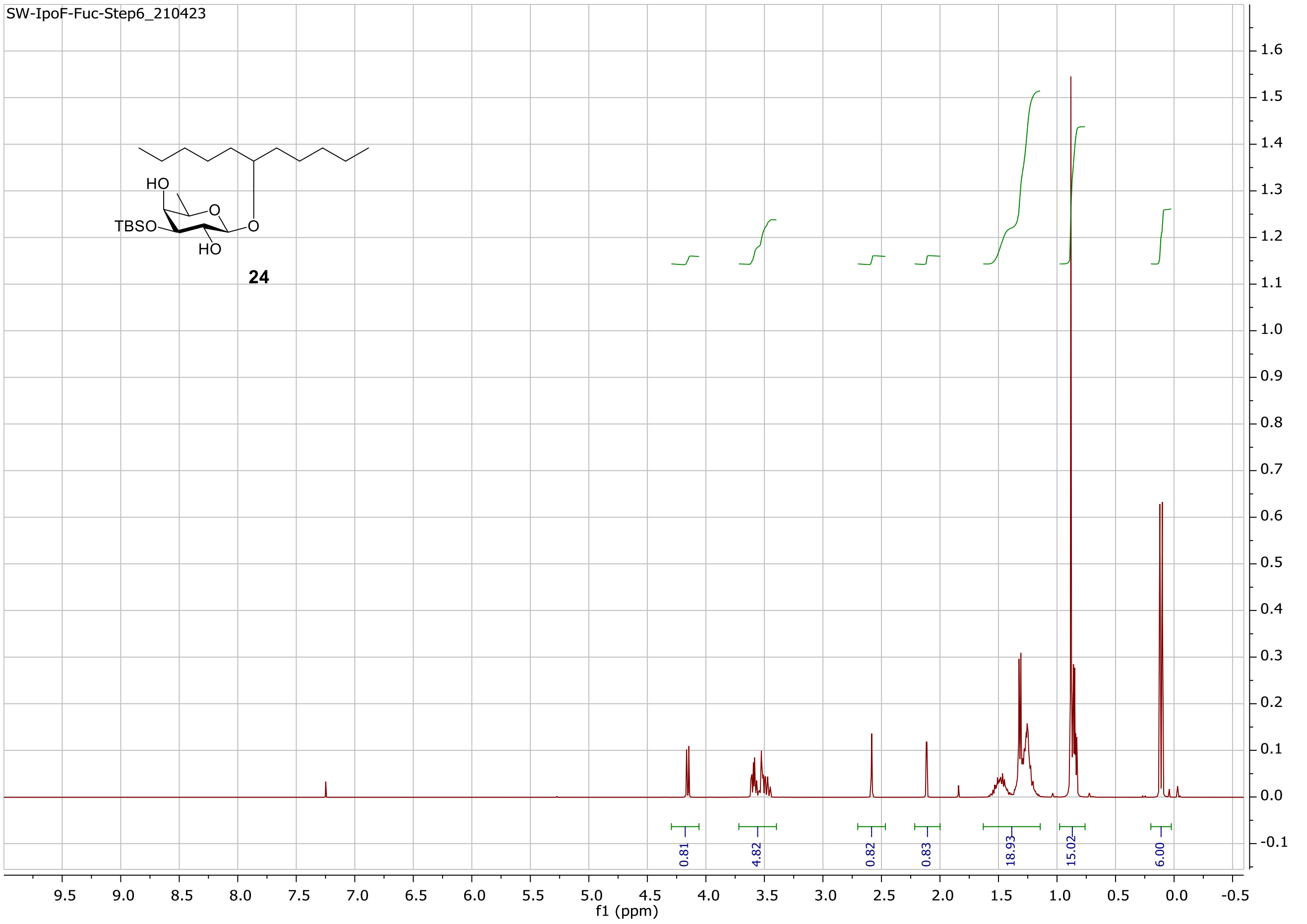
SW-IpoF-Fuc-Step6_210423
HO
TBSO
O
HO
O
24
0.81
4.82
0.82
0.83
18.93
15.02
6.00
f1 (ppm)

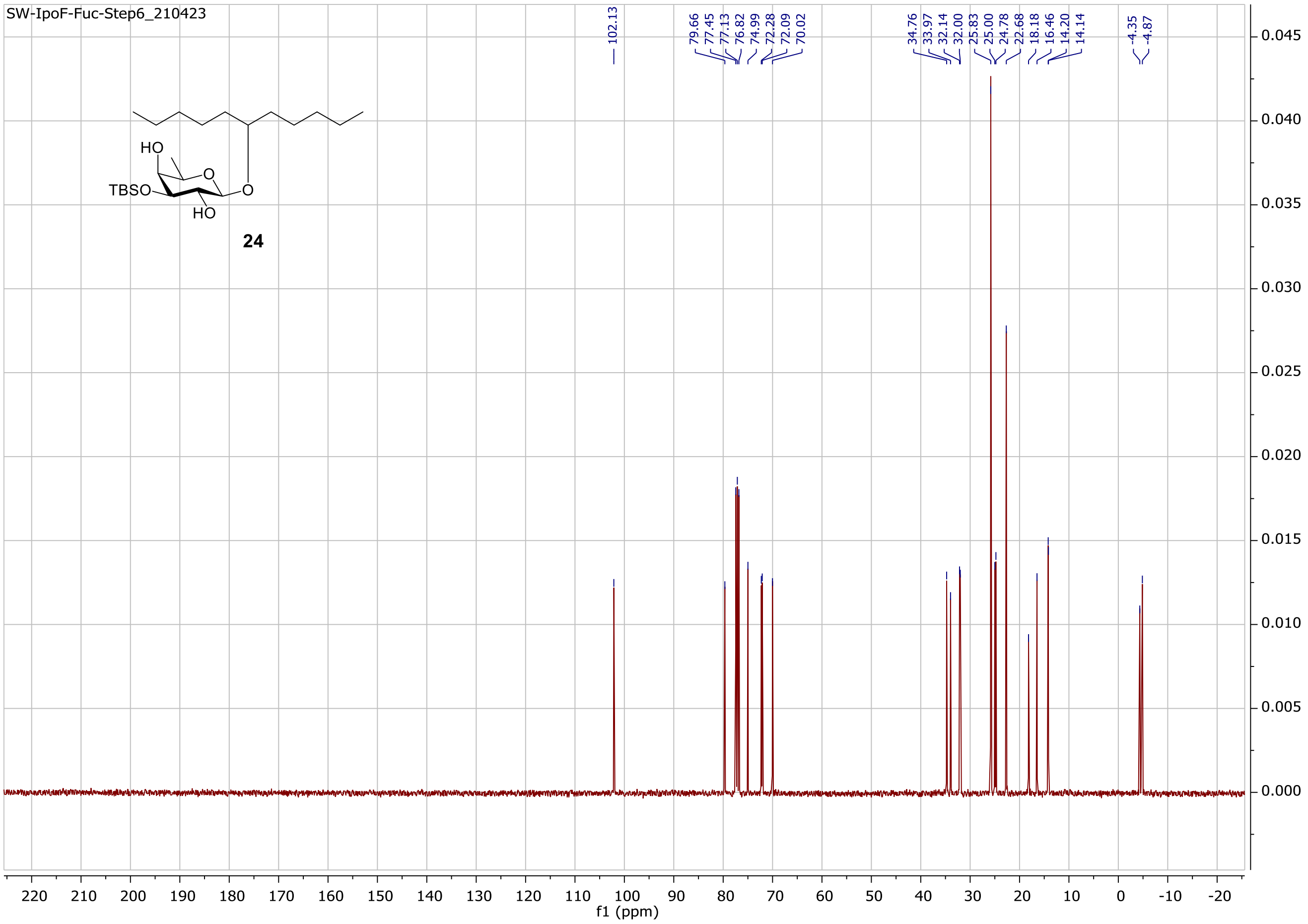
SW-IpoF-Fuc-Step6_210423
102.13
79.66
77.45
77.13
76.82
74.99
72.28
72.09
70.02
34.76
33.97
32.14
32.00
25.83
25.00
24.78
22.68
18.18
16.46
14.20
14.14
-4.35
-4.87
HO
TBSO
O
HO
O
24
f1 (ppm)

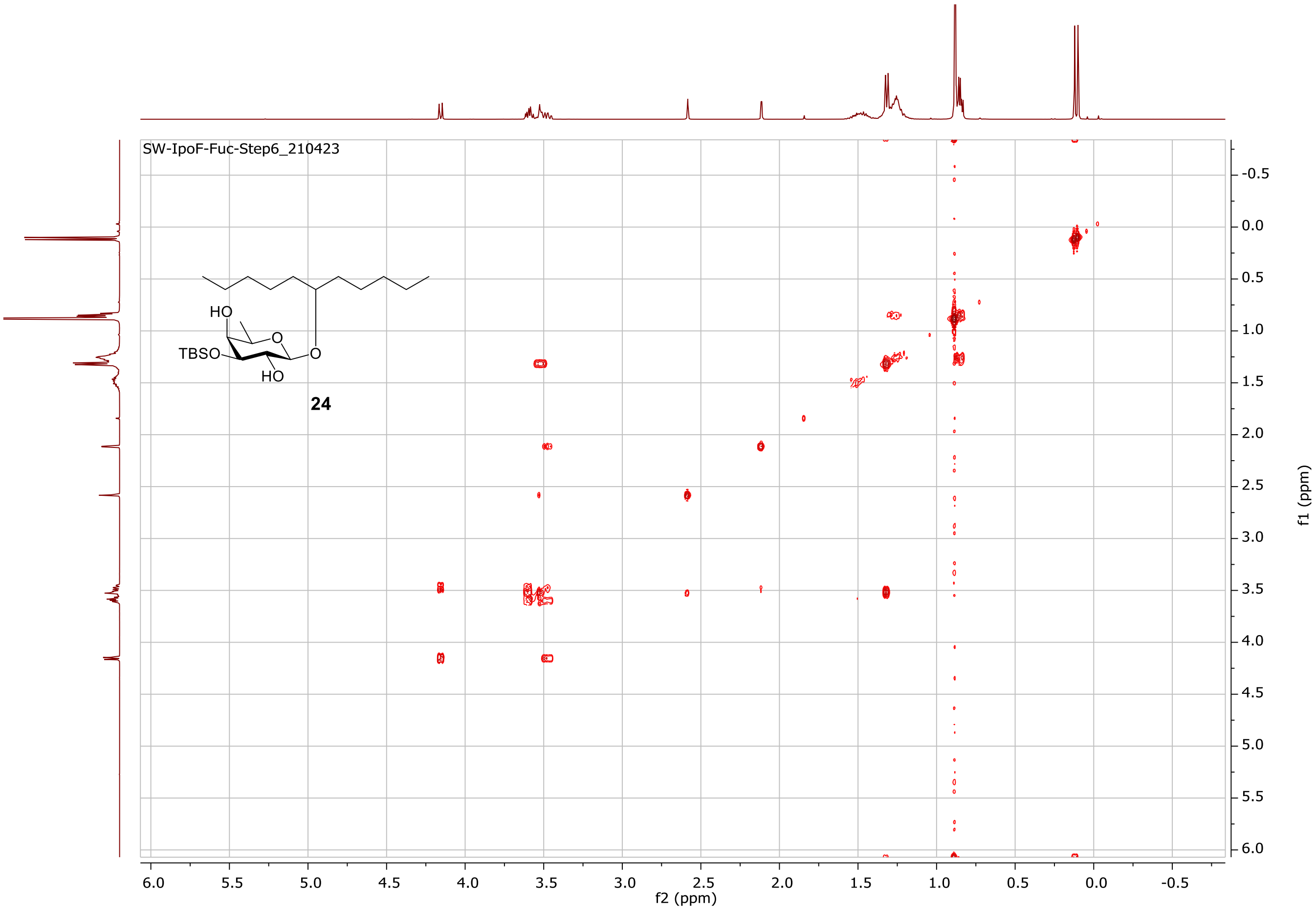
SW-IpoF-Fuc-Step6_210423
HO
O
TBSO
O
HO
24
f2 (ppm)
f1 (ppm)

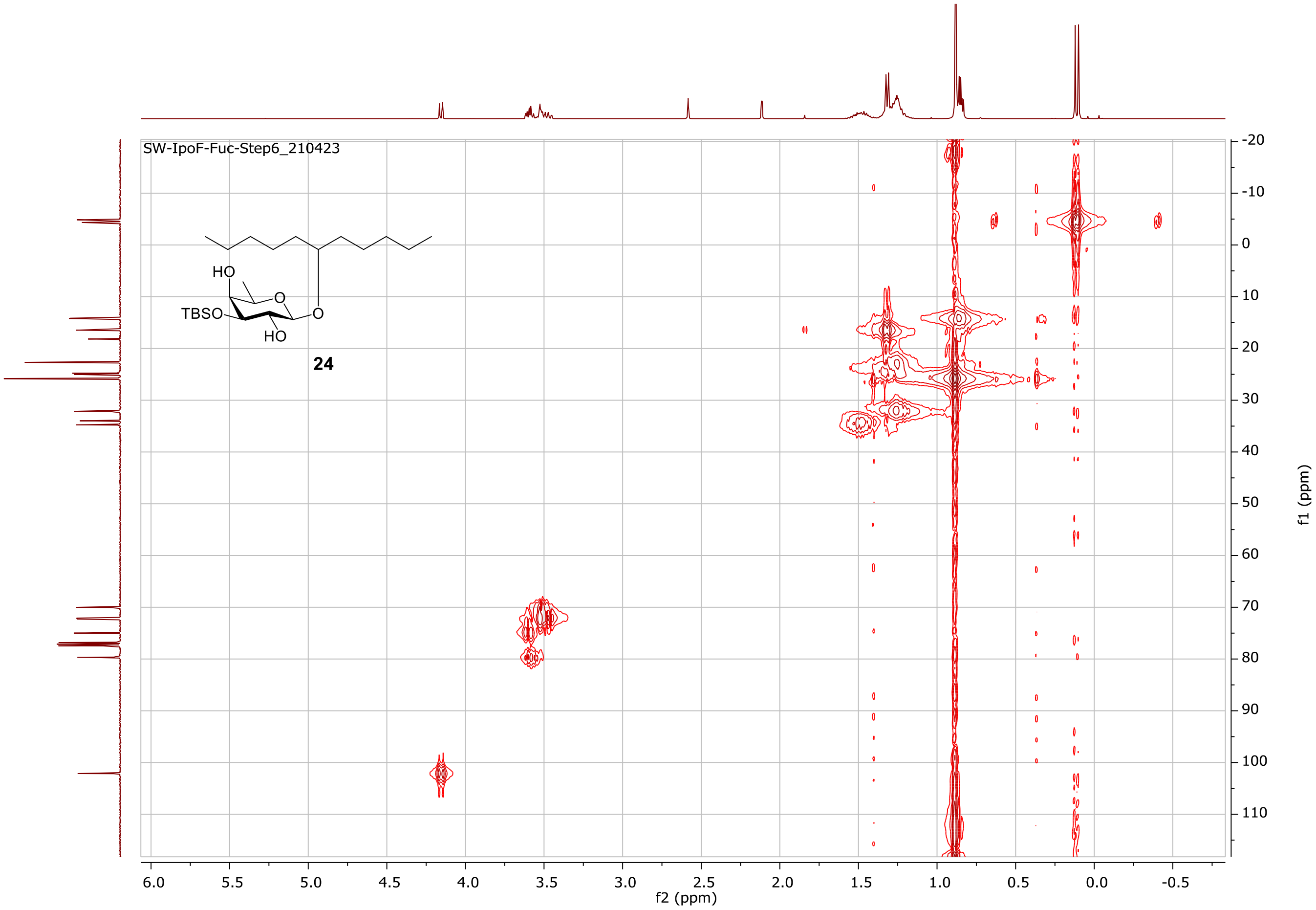
SW-IpoF-Fuc-Step6_210423
HO
O
TBSO
O
HO
24
f2 (ppm)
f1 (ppm)

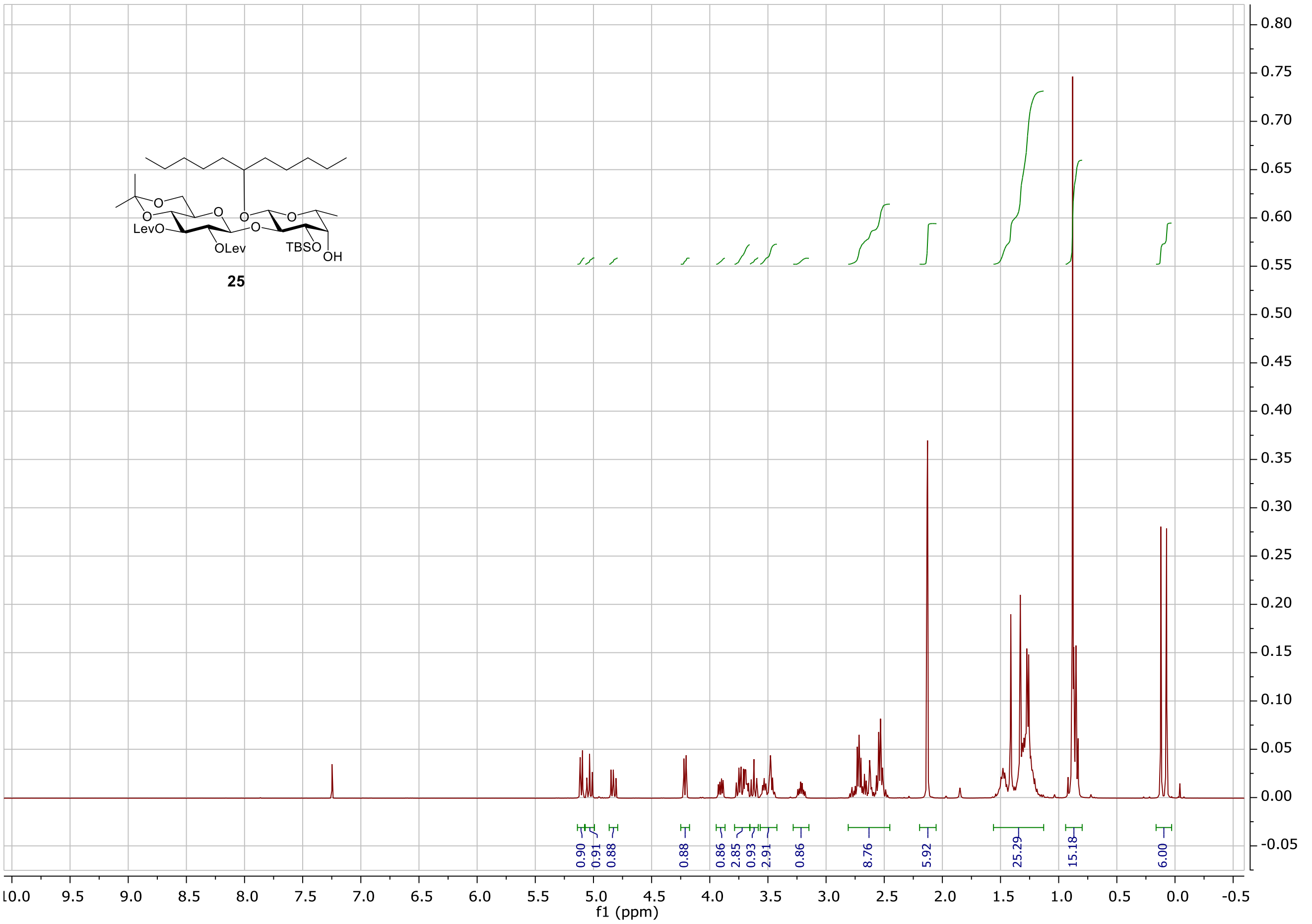

LevO
OLev
TBSO
OH
25
0.90
0.91
0.88
0.88
0.86
2.85
0.93
2.91
0.86
8.76
5.92
25.29
15.18
6.00
f1 (ppm)

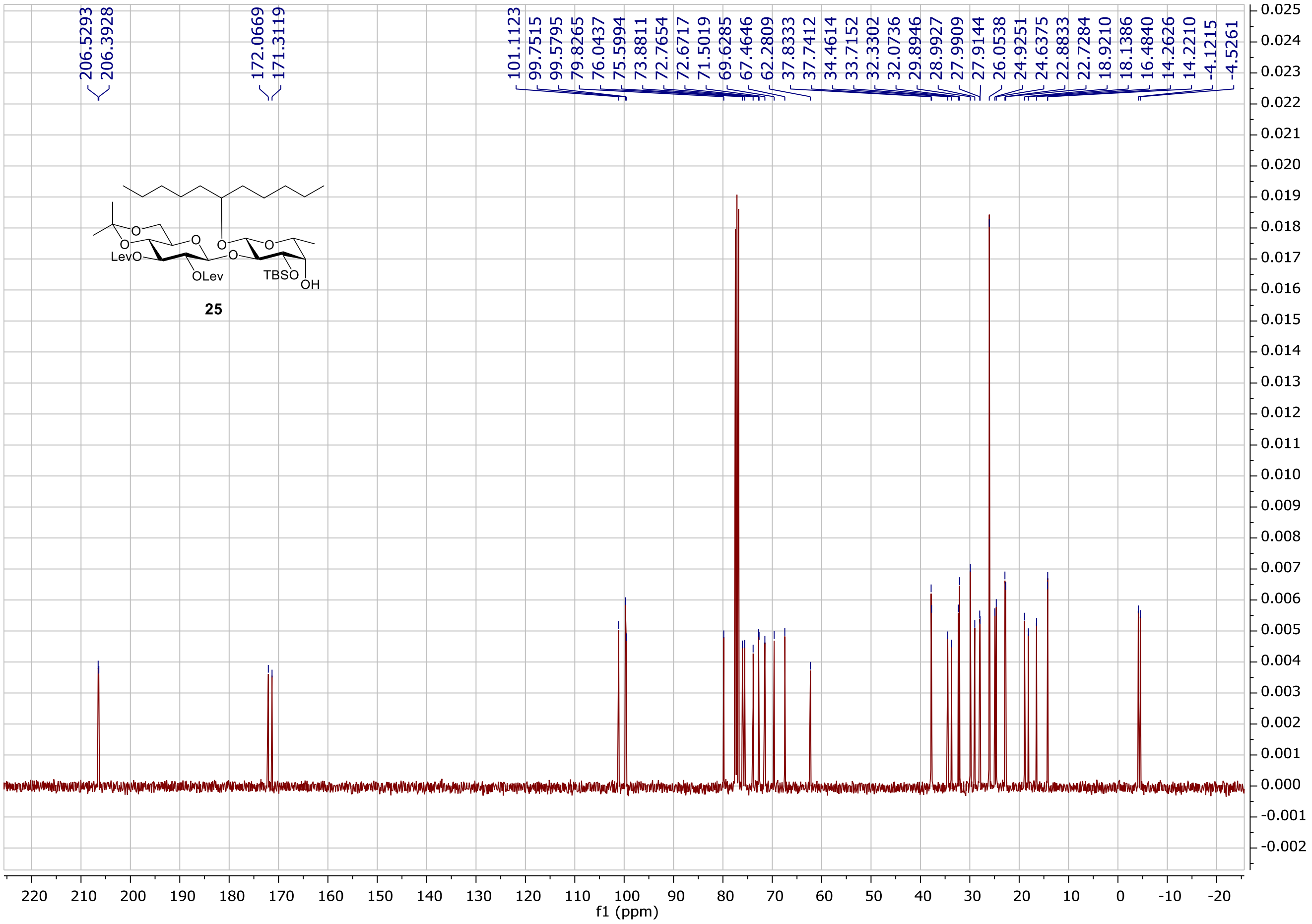

206.5293
206.3928
172.0669
171.3119
101.1123
99.7515
99.5795
79.8265
76.0437
75.5994
73.8811
72.7654
72.6717
71.5019
69.6285
67.4646
62.2809
37.8333
37.7412
34.4614
33.7152
32.3302
32.0736
29.8946
28.9927
27.9909
27.9144
26.0538
24.9251
24.6375
22.8833
22.7284
18.9210
18.1386
16.4840
14.2626
14.2210
-4.1215
-4.5261
LevO
OLev
TBSO
OH
25
f1 (ppm)

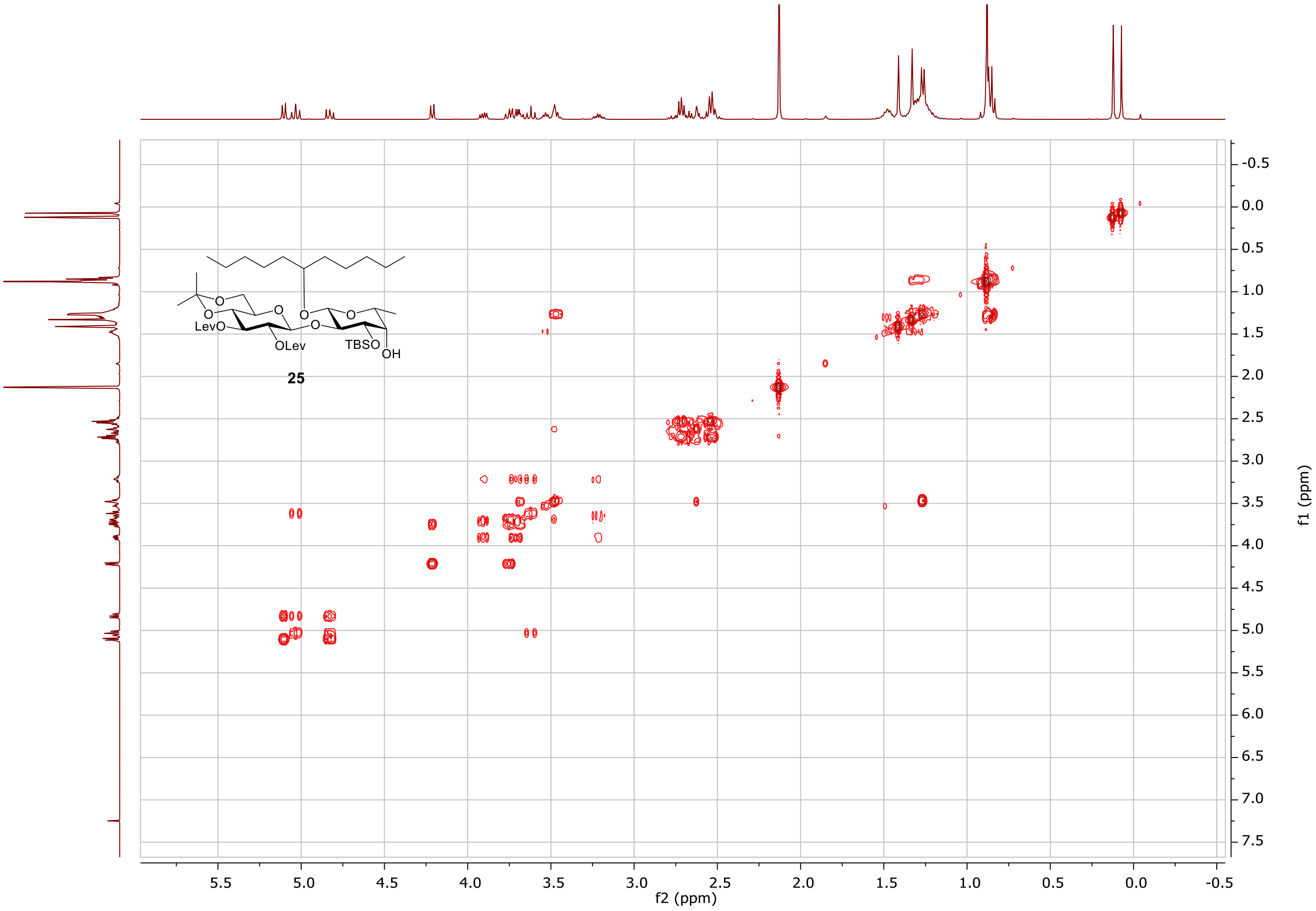

LevO
OLev
TBSO
OH
25
f2 (ppm)
f1 (ppm)

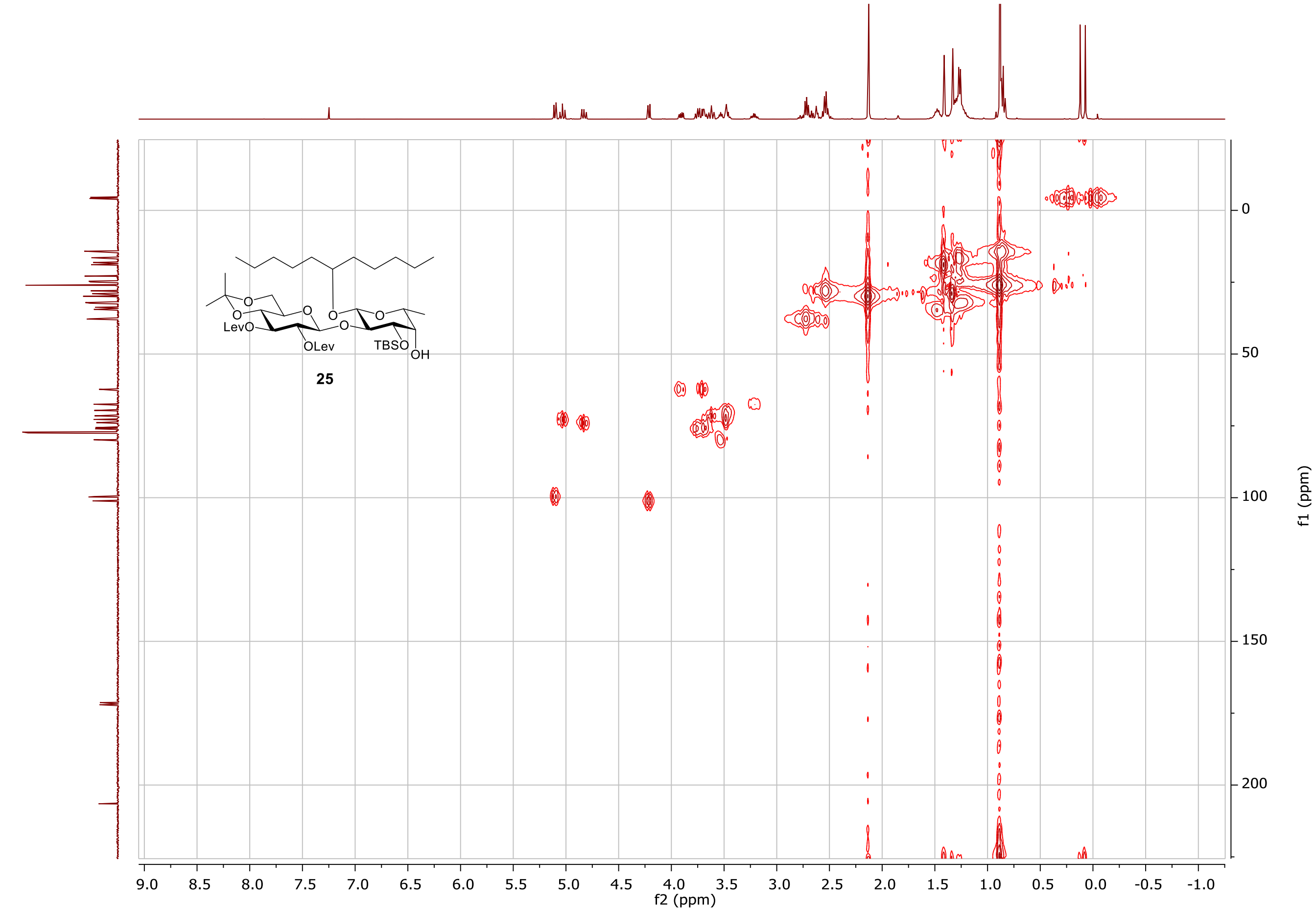

LevO
OLev
TBSO
OH
25
f2 (ppm)
f1 (ppm)

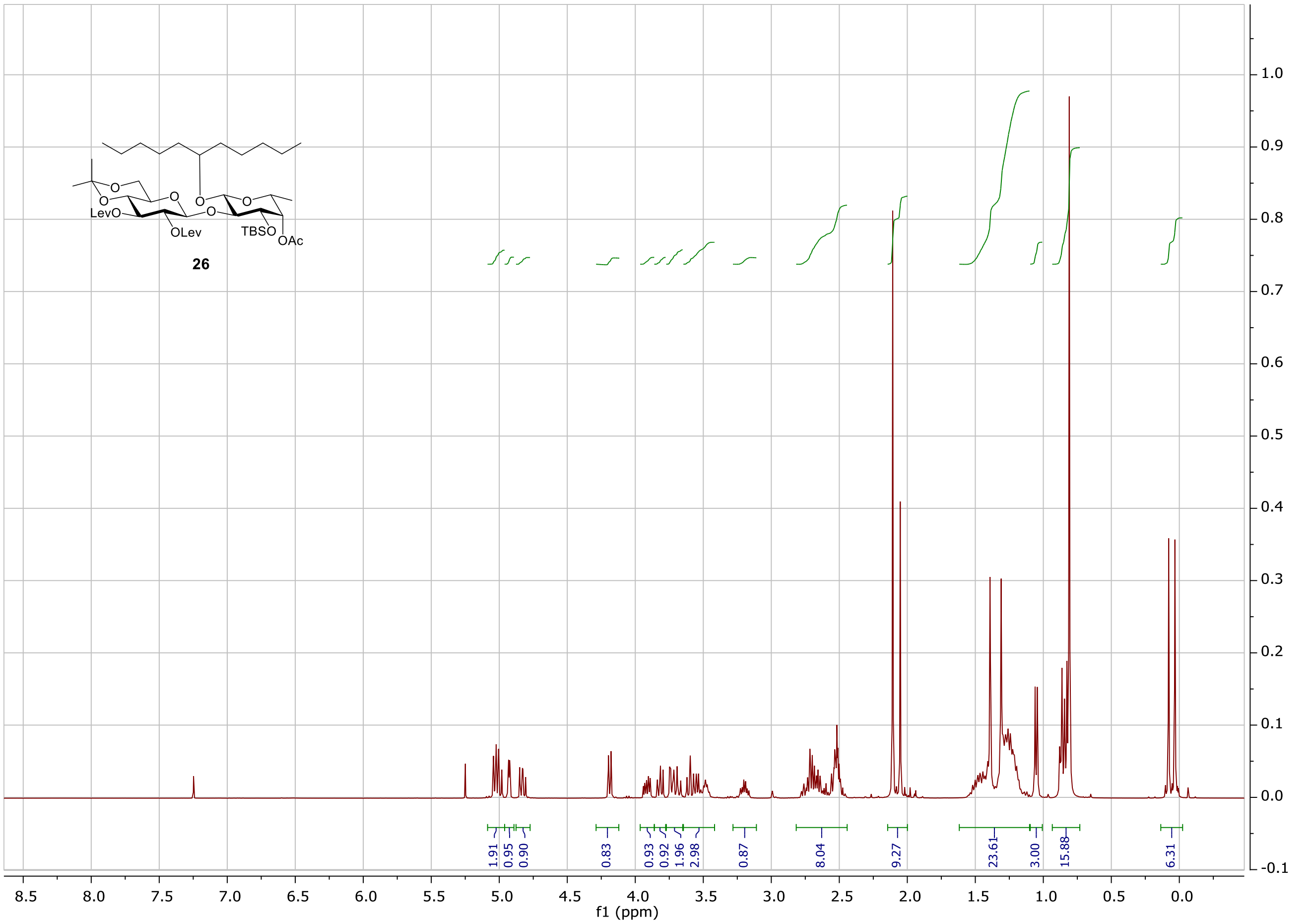
LevO
OLev
TBSO
OAc
26
1.91
0.95
0.90
0.83
0.93
0.92
1.96
2.98
0.87
8.04
9.27
23.61
3.00
15.88
6.31
f1 (ppm)

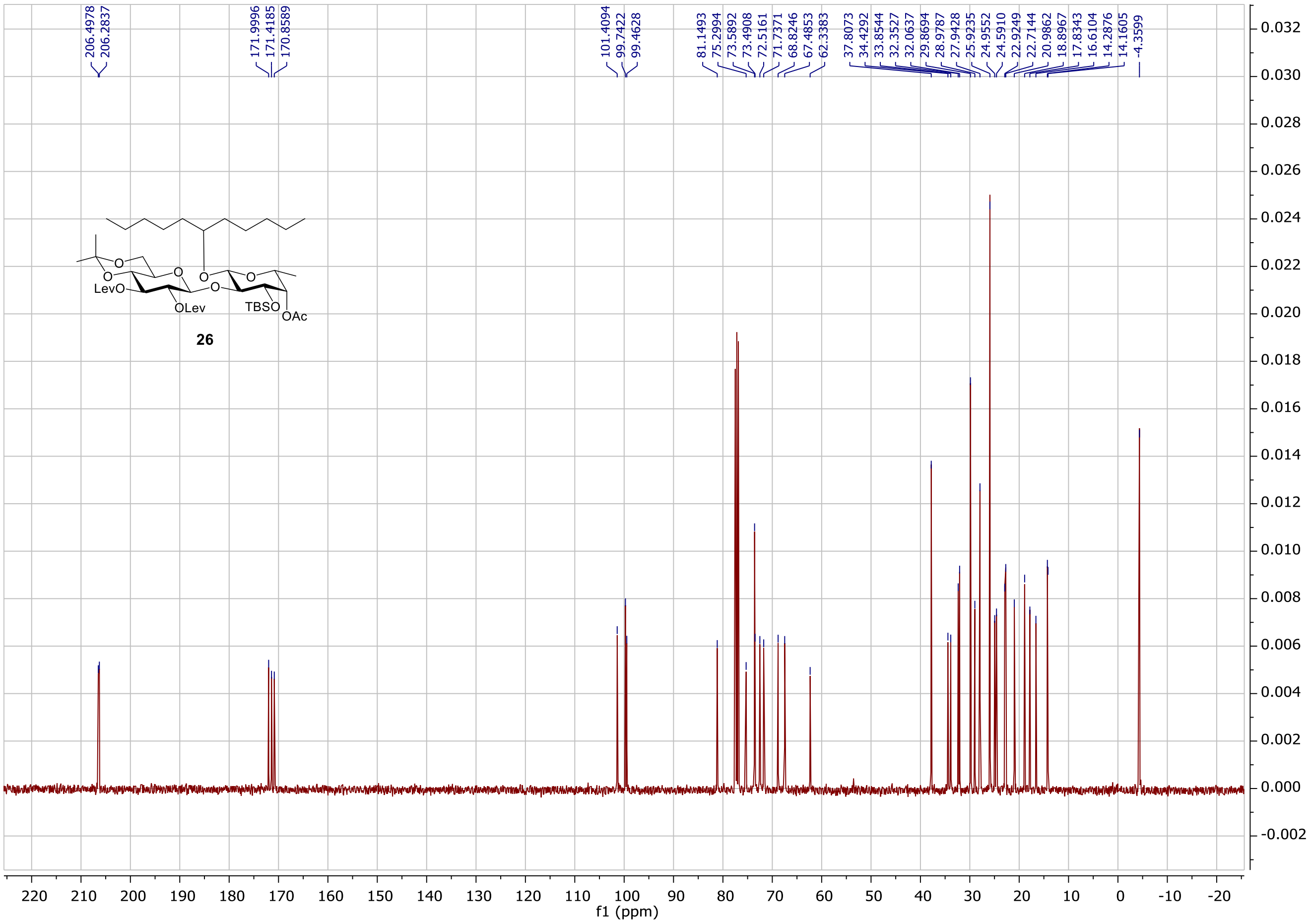
206.4978
206.2837
171.9996
171.4185
170.8589
101.4094
99.7422
99.4628
81.1493
75.2994
73.5892
73.4908
72.5161
71.7371
68.8246
67.4853
62.3383
37.8073
34.4292
33.8544
32.3527
32.0637
29.8694
28.9787
27.9428
25.9235
24.9552
24.5910
22.9249
22.7144
20.9862
18.8967
17.8343
16.6104
14.2876
14.1605
-4.3599
LevO
OLev
TBSO
OAc
26
f1 (ppm)

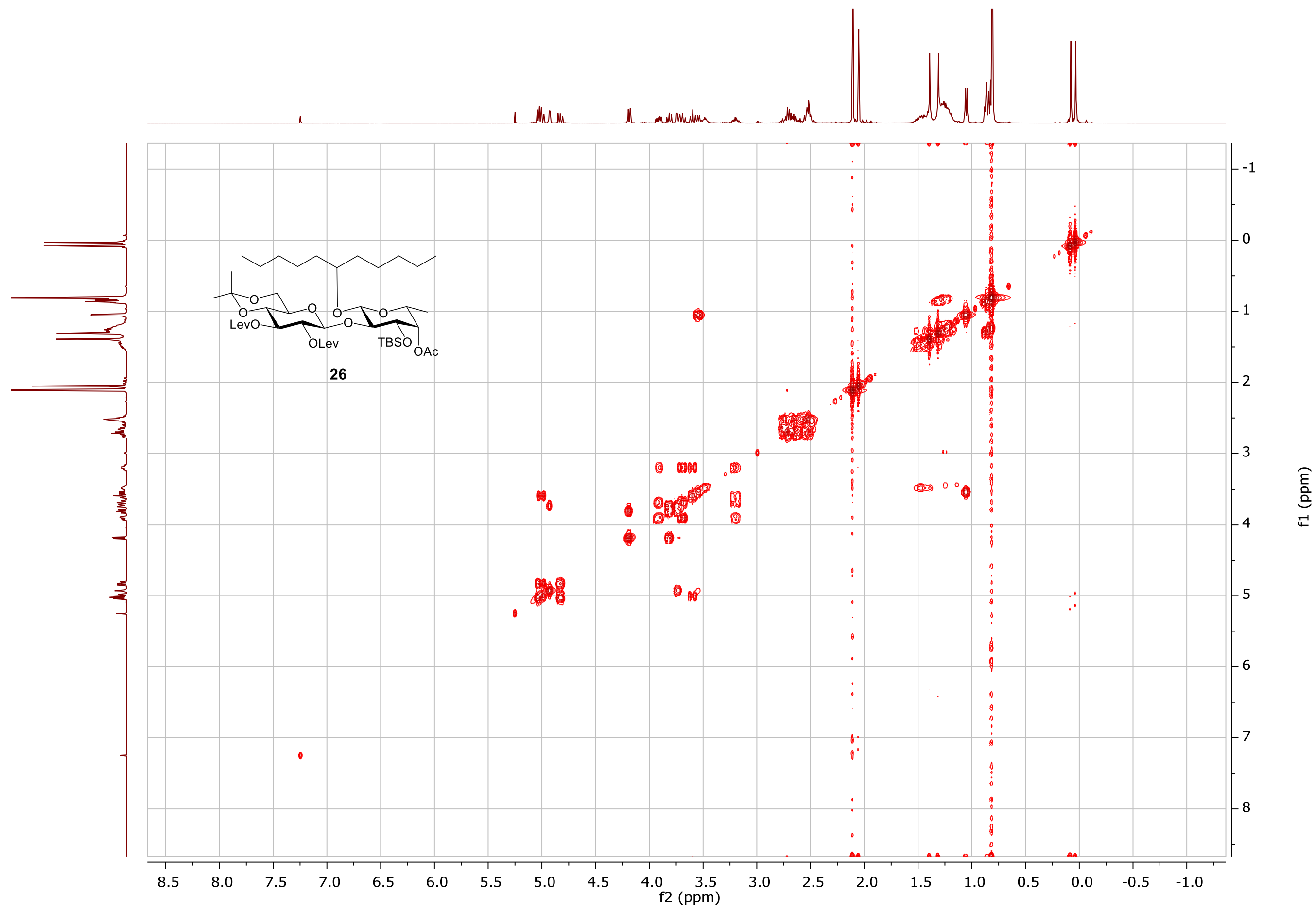

LevO
OLev
TBSO
OAc
26
f1 (ppm)
f2 (ppm)

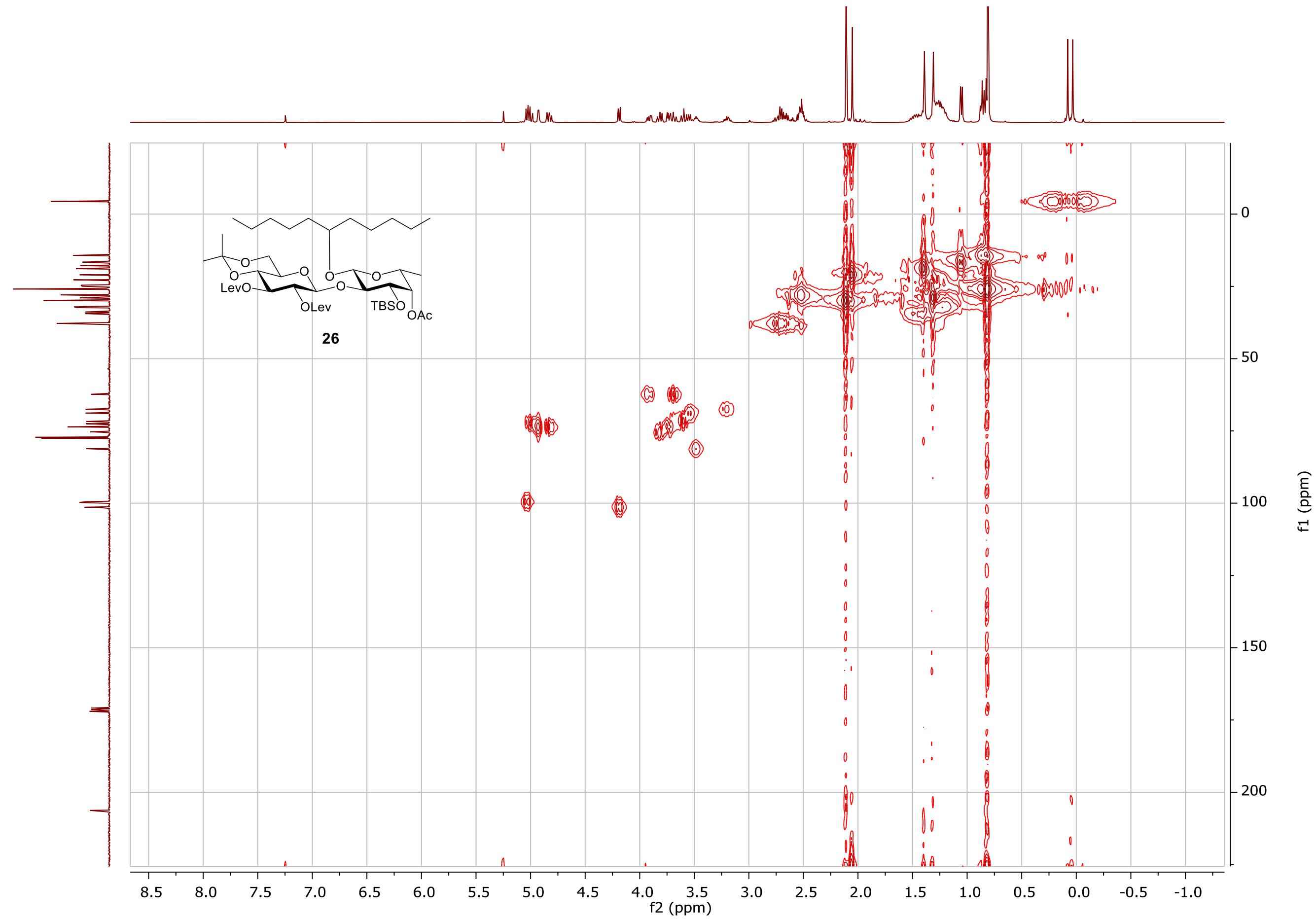

LevO
OLev
TBSO
OAc
26
f1 (ppm)
f2 (ppm)

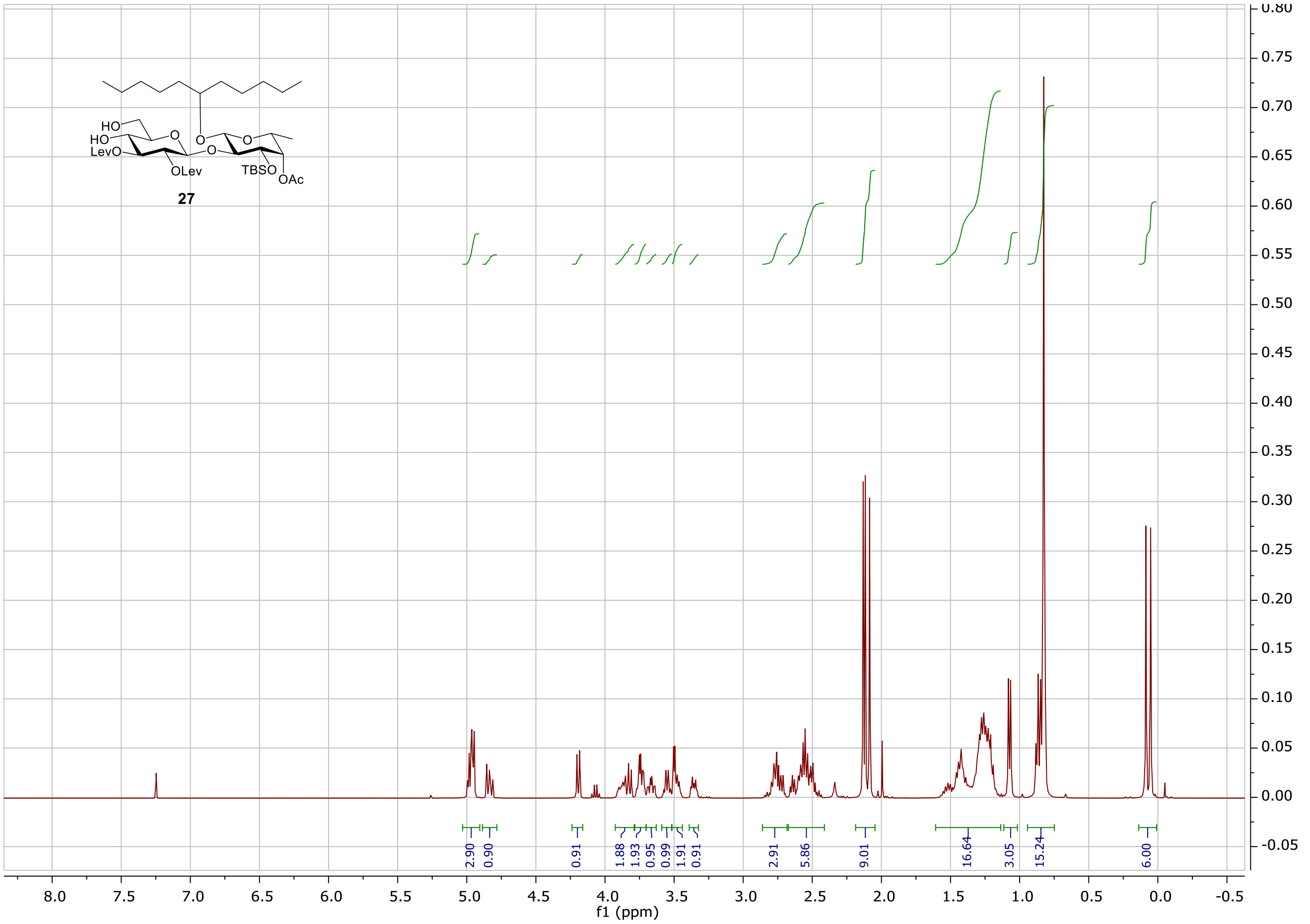
HO
HO
LevO
OLev
TBSO
OAc
27
2.90
0.90
0.91
1.88
1.93
0.95
0.99
1.91
0.91
2.91
5.86
9.01
16.64
3.05
15.24
6.00
f1 (ppm)

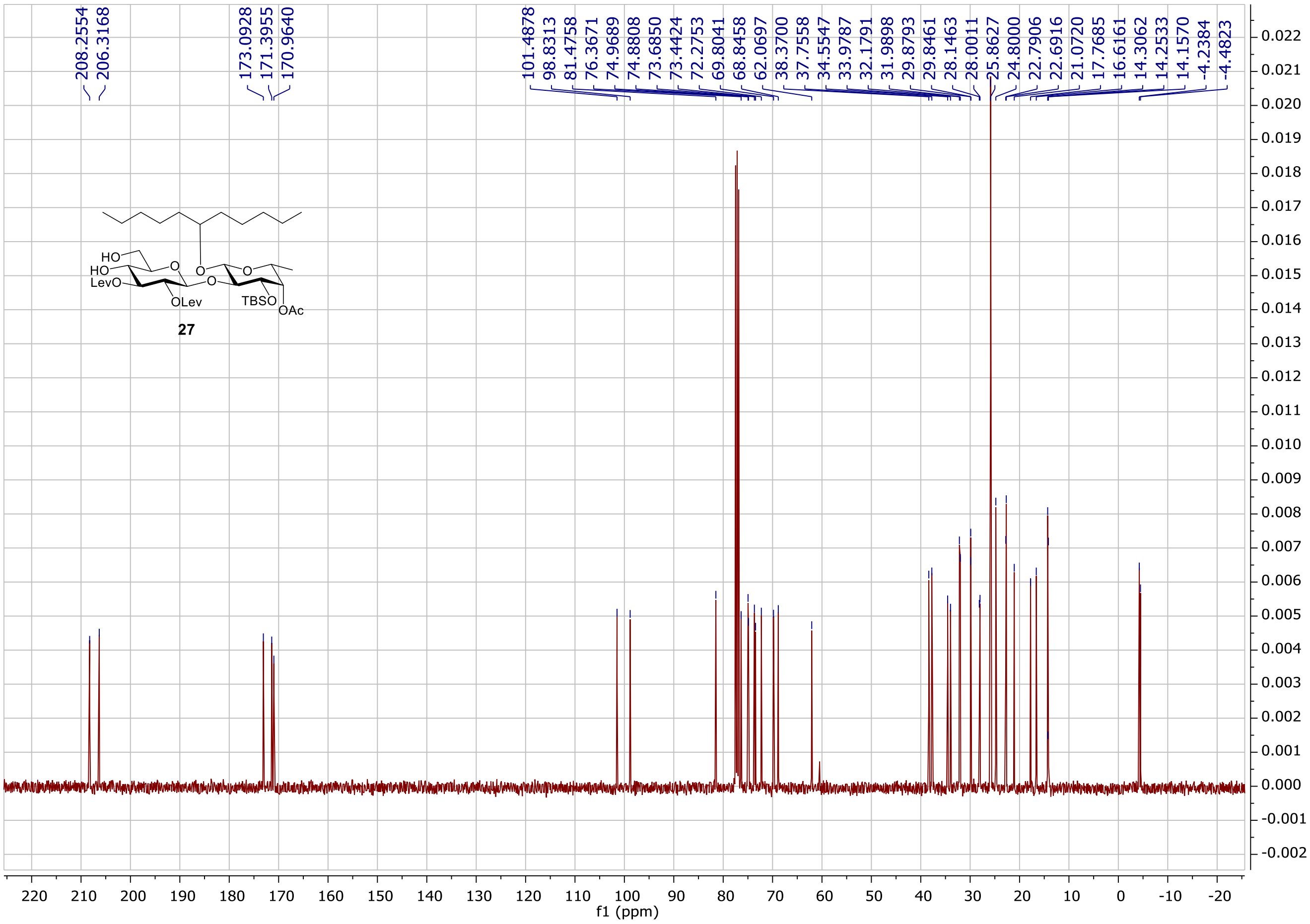
208.2554
206.3168
173.0928
171.3955
170.9640
101.4878
98.8313
81.4758
76.3671
74.9689
74.8808
73.6850
73.4424
72.2753
69.8041
68.8458
62.0697
38.3700
37.7558
34.5547
33.9787
32.1791
31.9898
29.8793
29.8461
28.1463
28.0011
25.8627
24.8000
22.7906
22.6916
21.0720
17.7685
16.6161
14.3062
14.2533
14.1570
-4.2384
-4.4823
HO
HO
LevO
OLev
TBSO
OAc
27
f1 (ppm)

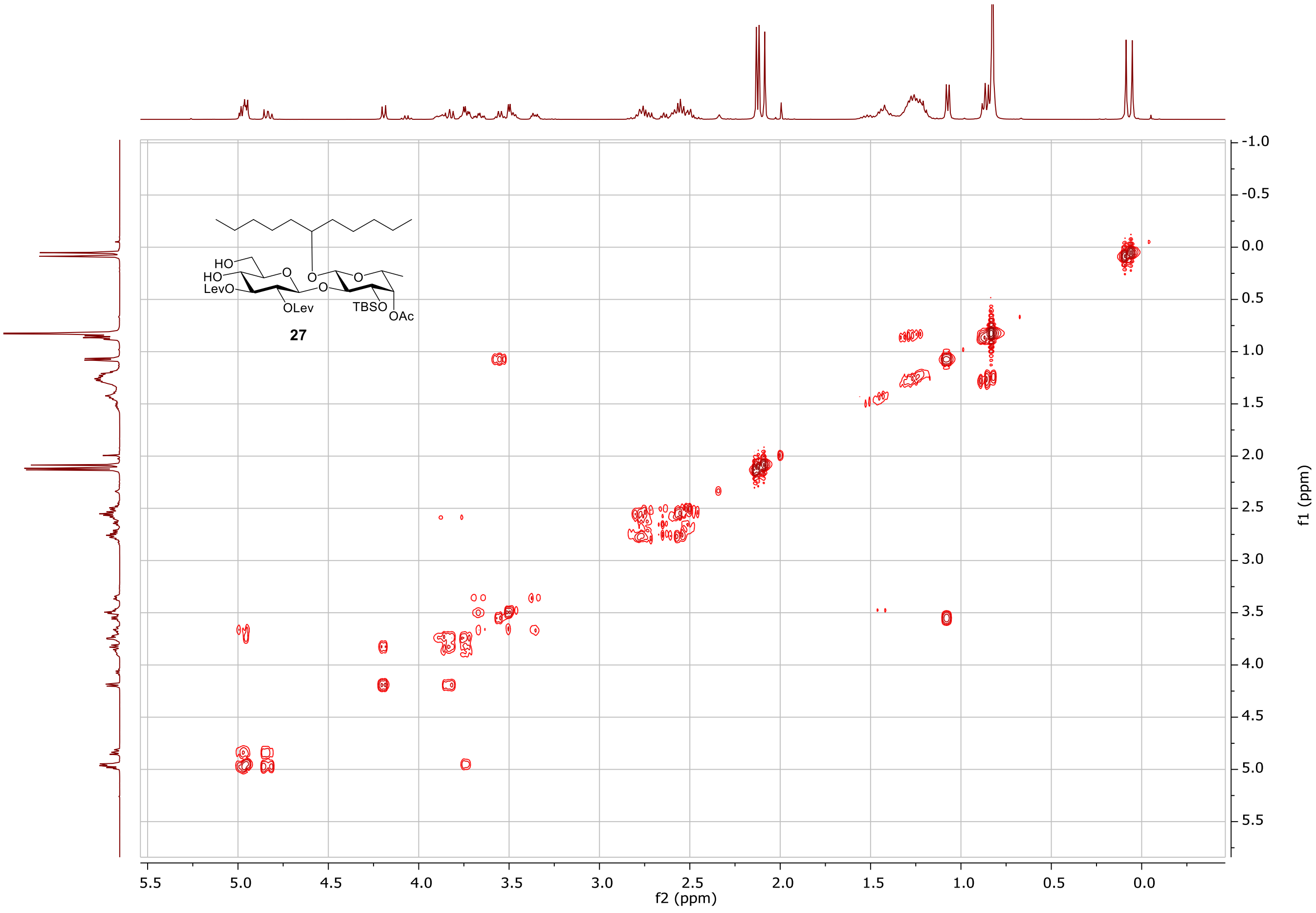
HO
HO
LevO
O
O
O
O
OLev
TBSO
OAc
27
f2 (ppm)
f1 (ppm)

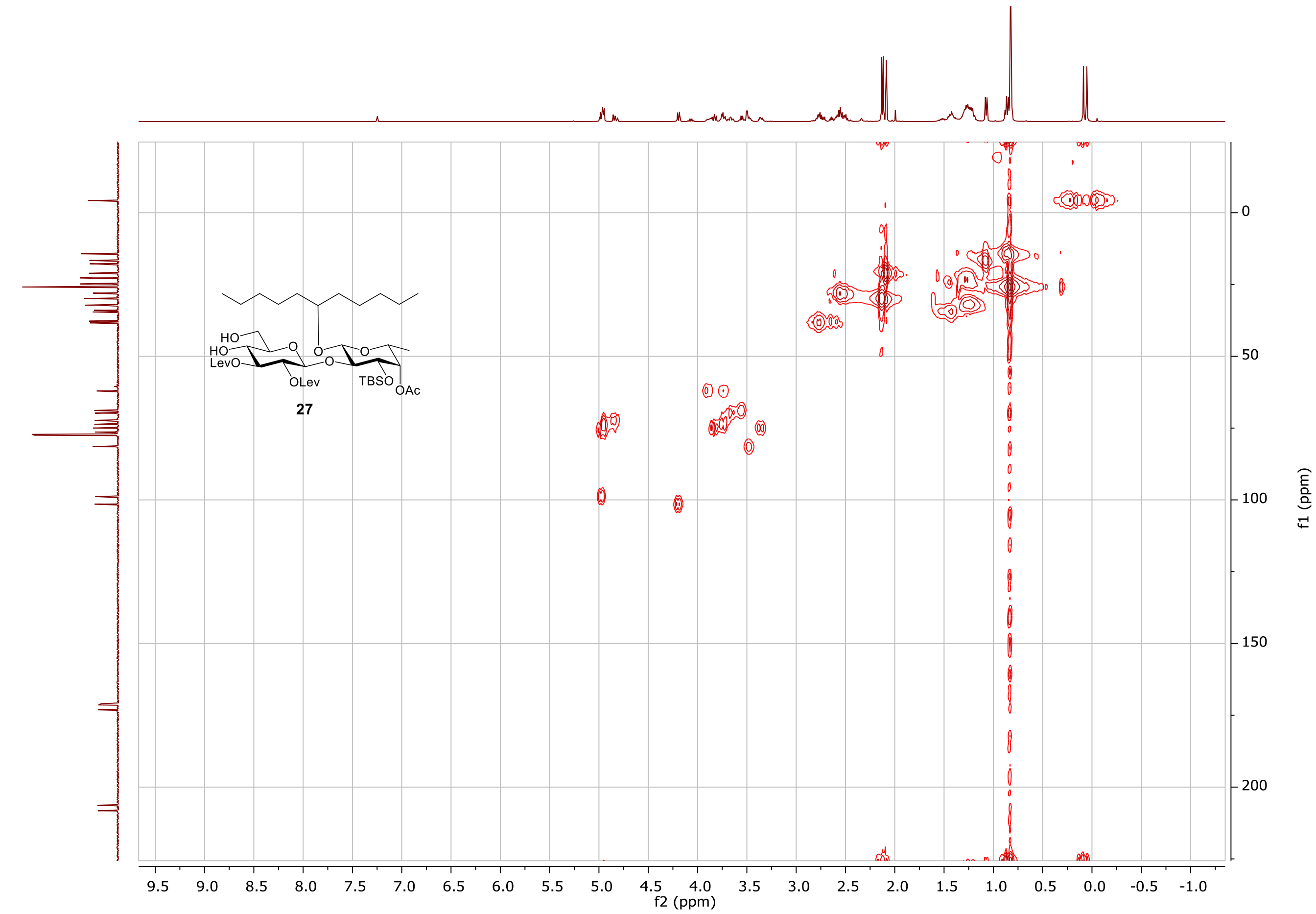
HO
HO
LevO
O
O
O
O
OLev
TBSO
OAc
27
f2 (ppm)
f1 (ppm)

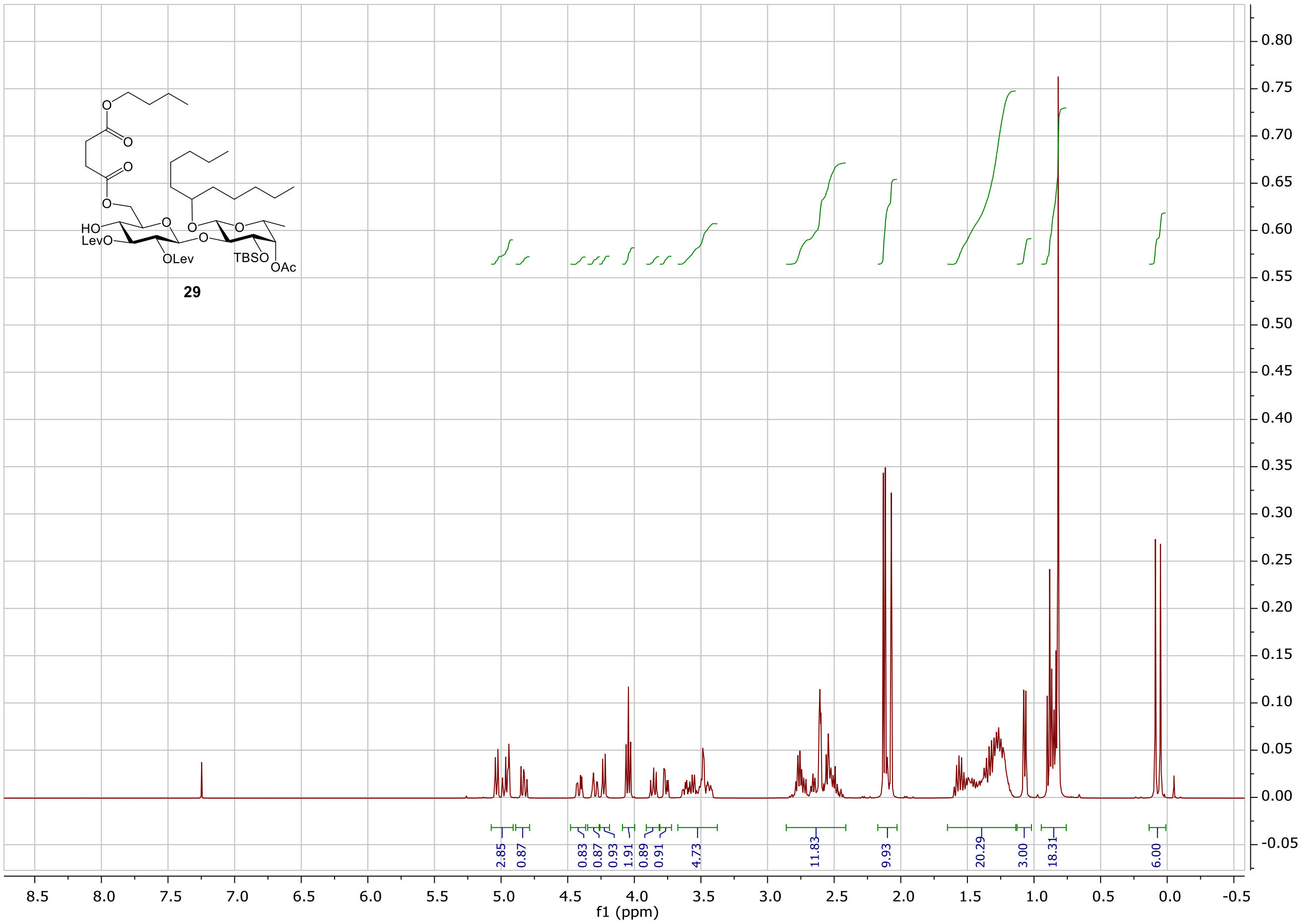
HO
LevO
OLev
TBSO
OAc
29
2.85
0.87
0.83
0.87
0.93
1.91
0.89
0.91
4.73
11.83
9.93
20.29
3.00
18.31
6.00
f1 (ppm)

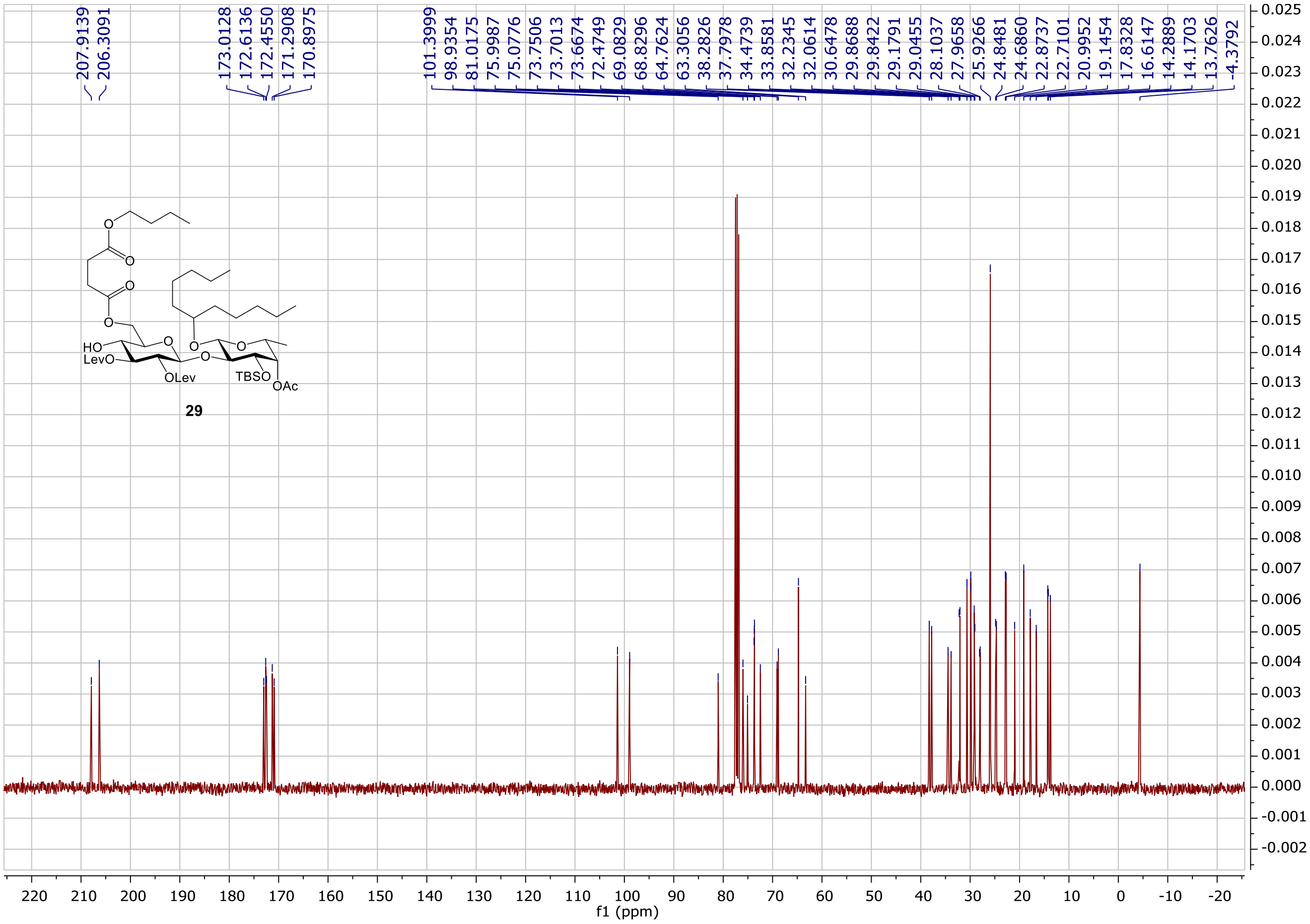
207.9139
206.3091
173.0128
172.6136
172.4550
171.2908
170.8975
101.3999
98.9354
81.0175
75.9987
75.0776
73.7506
73.7013
73.6674
72.4749
69.0829
68.8296
64.7624
63.3056
38.2826
37.7978
34.4739
33.8581
32.2345
32.0614
30.6478
29.8688
29.8422
29.1791
29.0455
28.1037
27.9658
25.9266
24.8481
24.6860
22.8737
22.7101
20.9952
19.1454
17.8328
16.6147
14.2889
14.1703
13.7626
-4.3792
HO
LevO
OLev
TBSO
OAc
29
f1 (ppm)

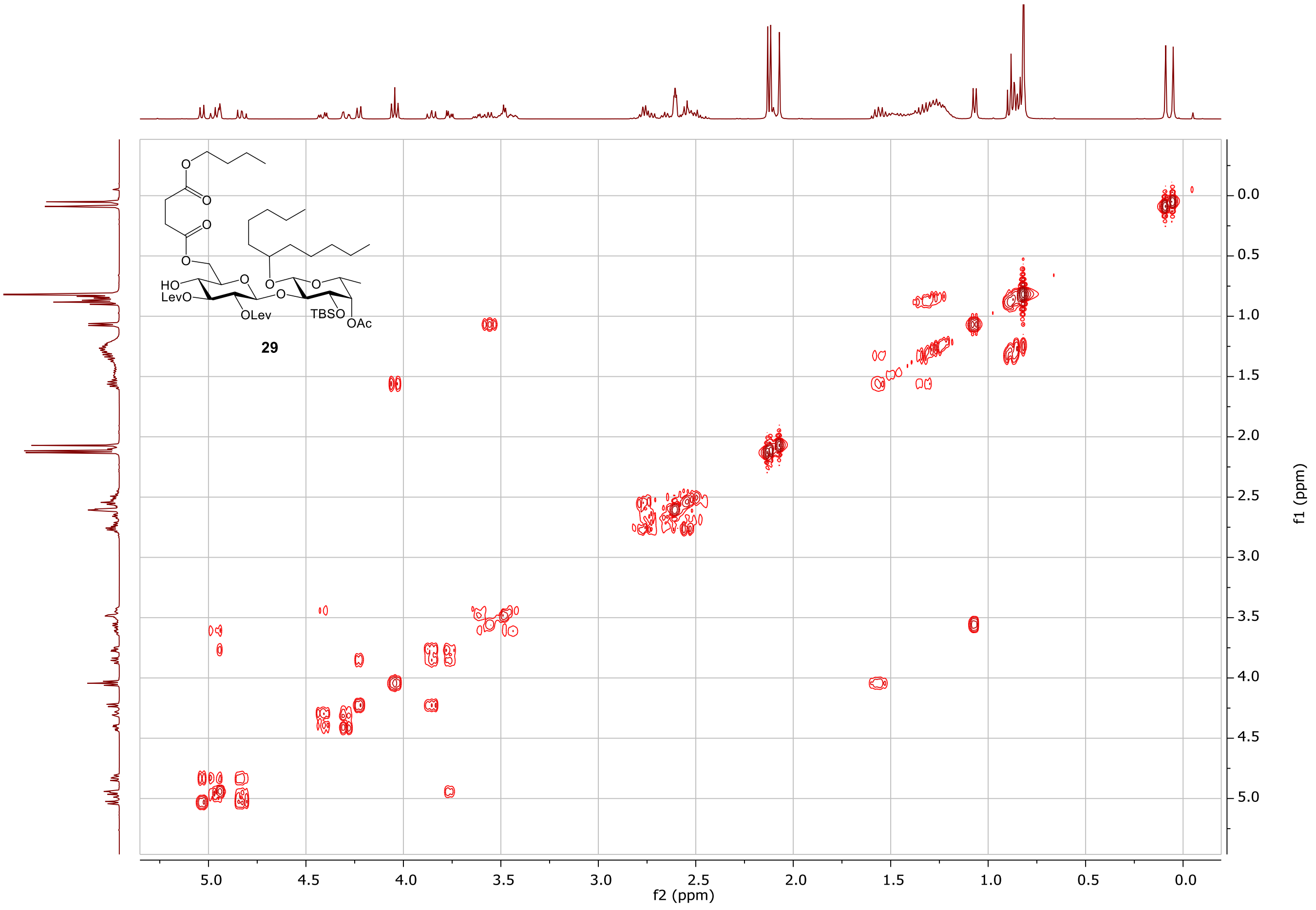
HO
LevO
O
OLev
O
O
O
TBSO
OAc
O
O
O
O
29
f1 (ppm)
f2 (ppm)

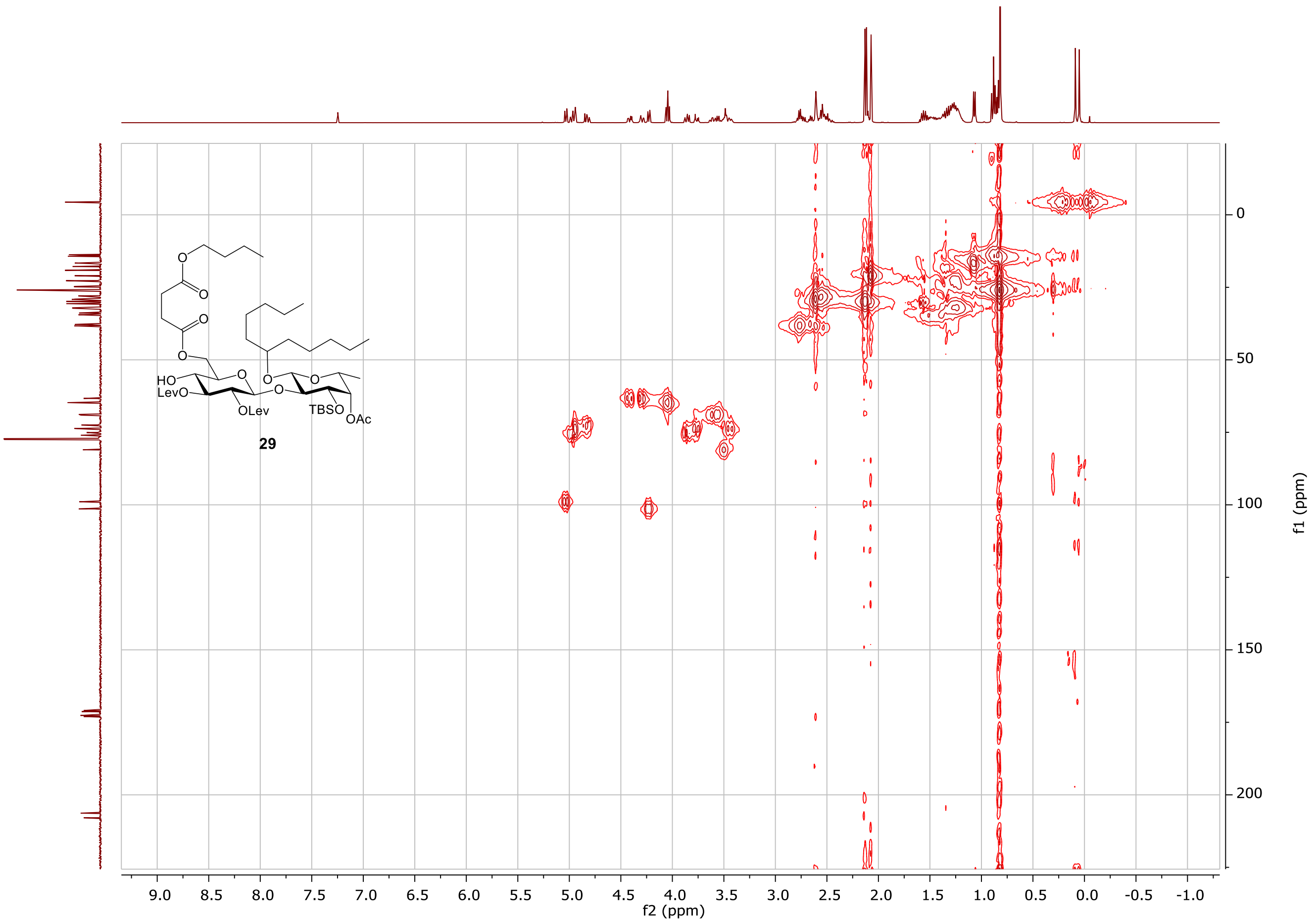
HO
LevO
O
OLev
O
O
O
TBSO
OAc
O
O
O
O
29
f1 (ppm)
f2 (ppm)

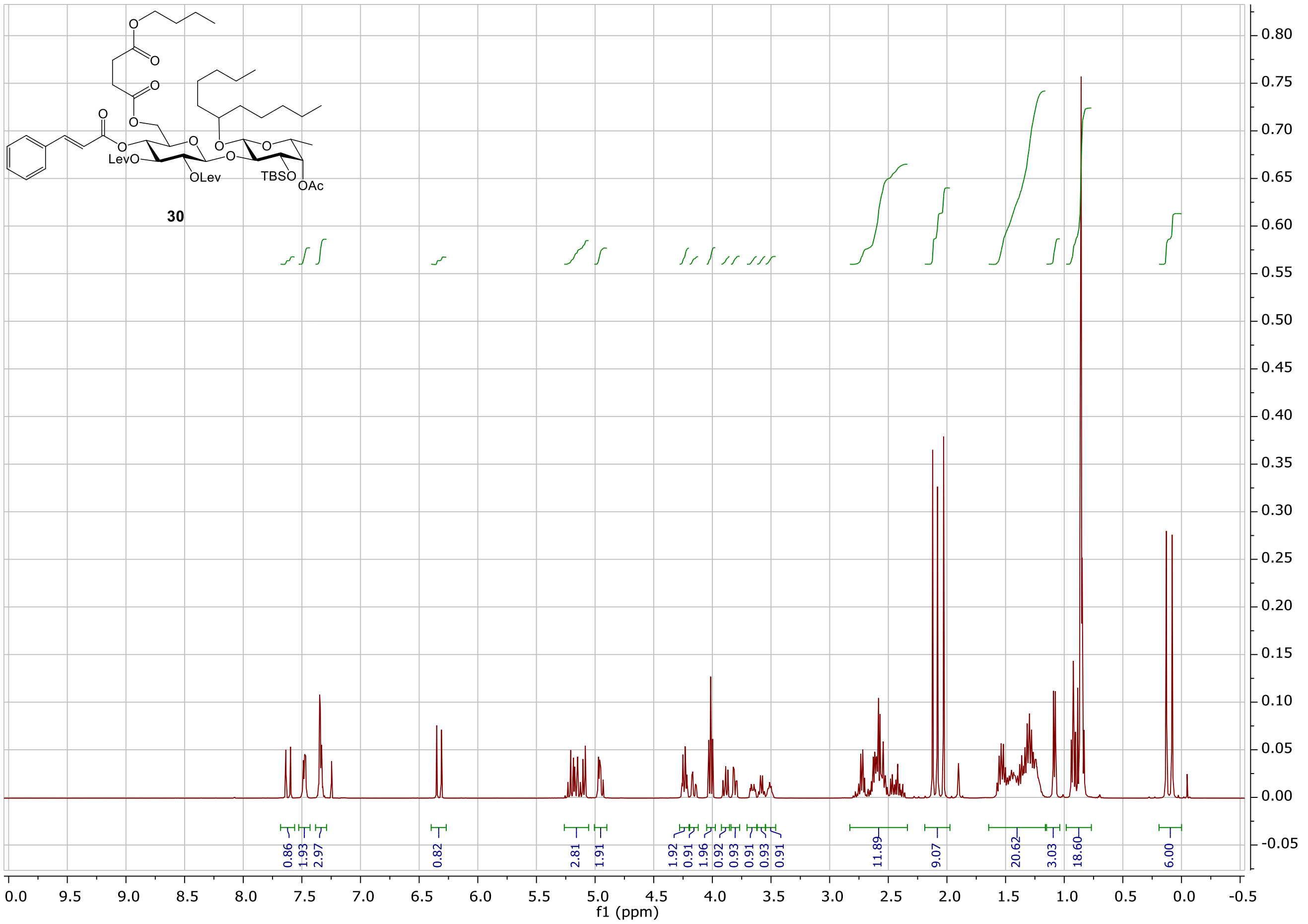

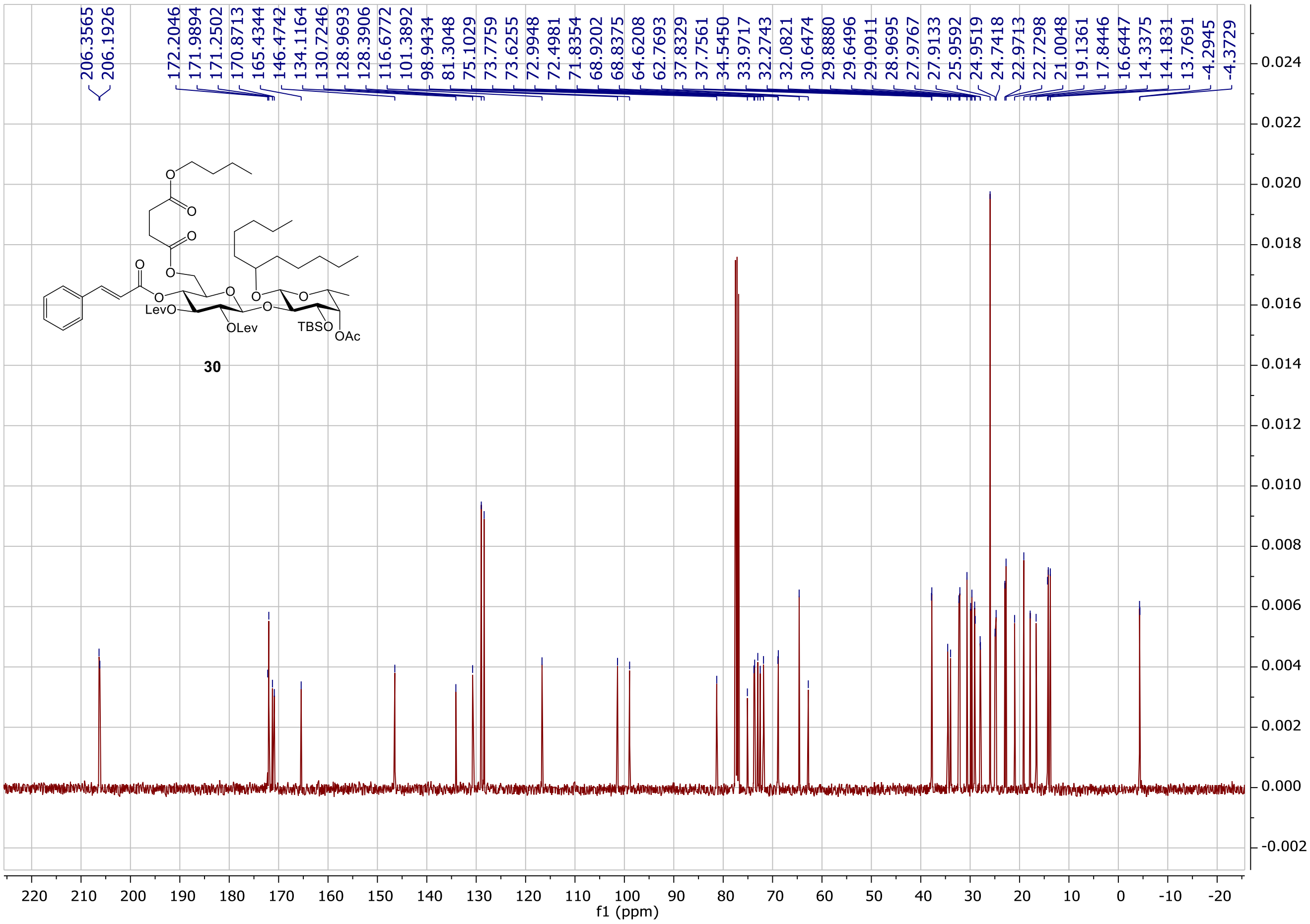

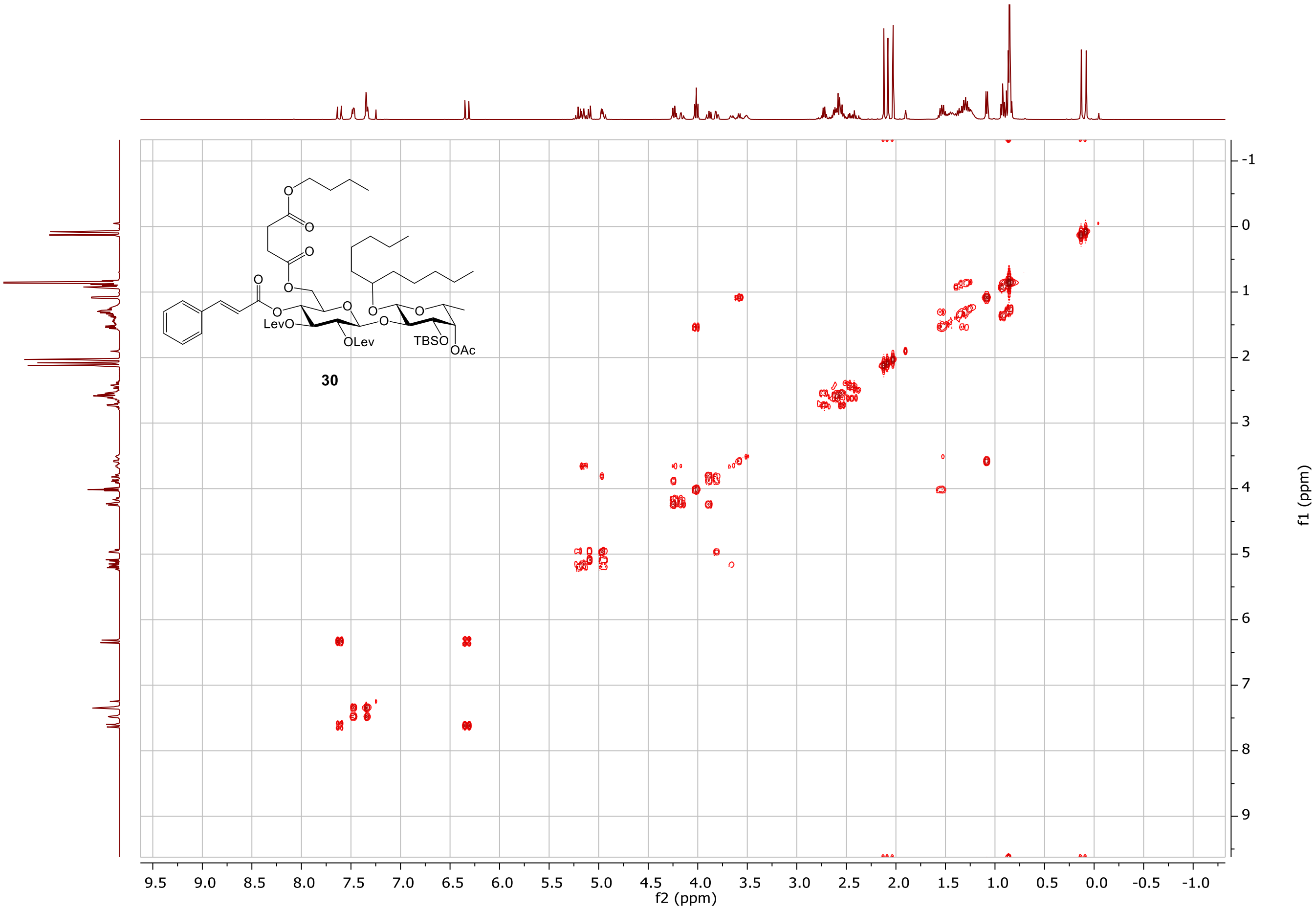
O
O
O
O
O
LevO
O
O
OLev
O
O
TBSO
OAc
30
f2 (ppm)
f1 (ppm)

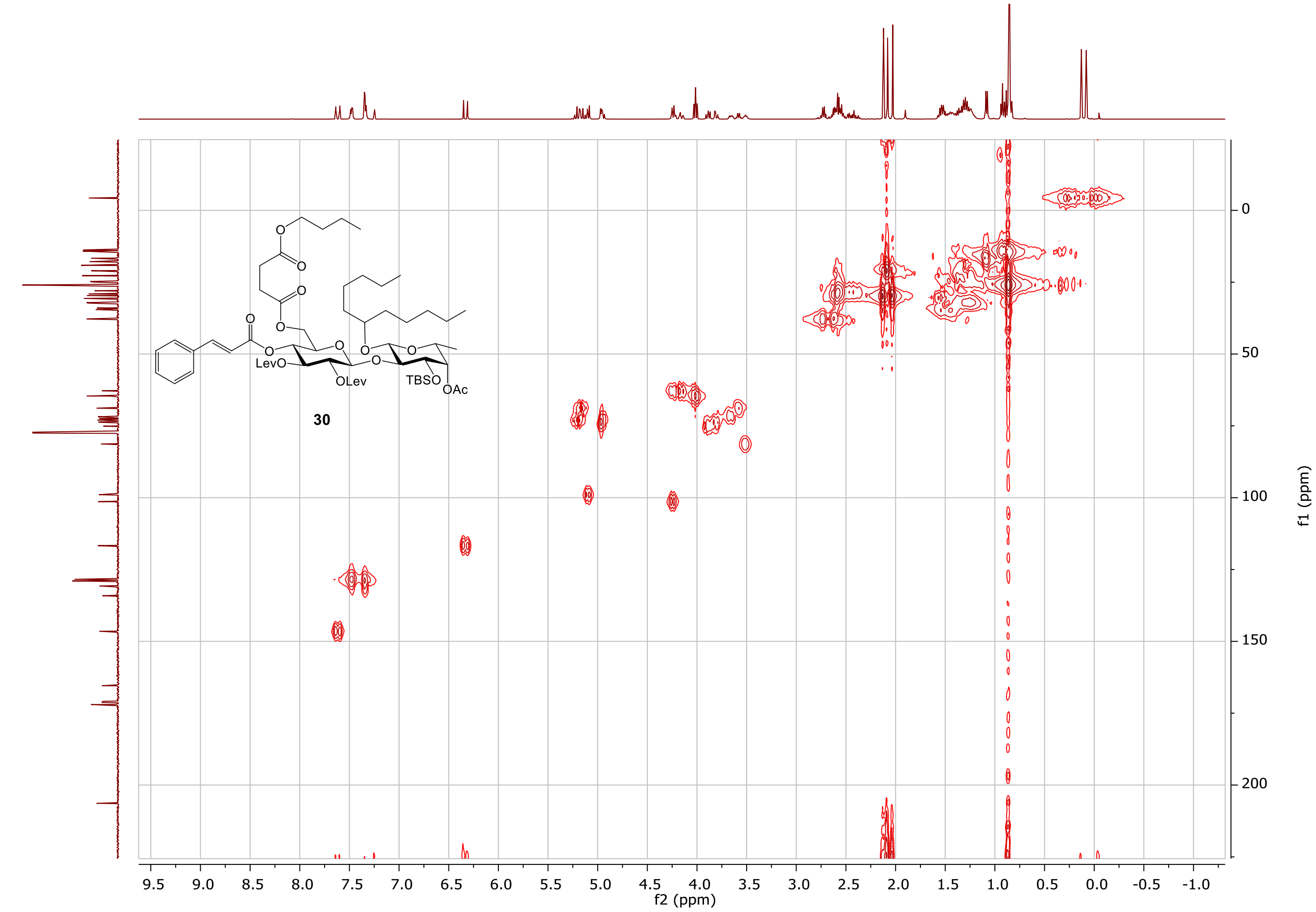
O
O
O
O
O
LevO
O
O
OLev
O
O
TBSO
OAc
30
f2 (ppm)
f1 (ppm)

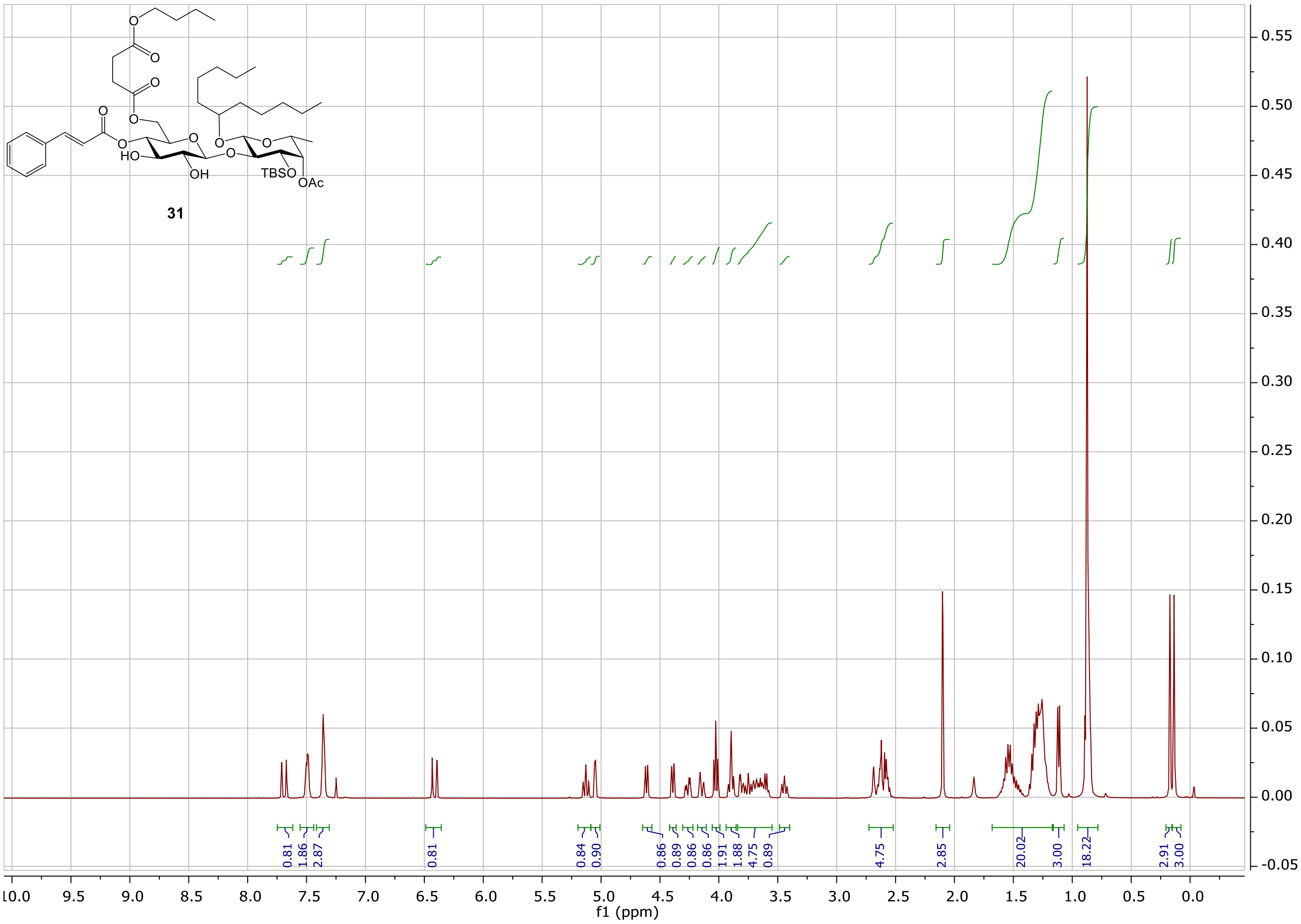
31
HO
OH
TBSO
OAc
0.81
1.86
2.87
0.81
0.84
0.90
0.86
0.89
0.86
0.86
1.91
1.88
4.75
0.89
4.75
2.85
20.02
3.00
18.22
2.91
3.00
f1 (ppm)

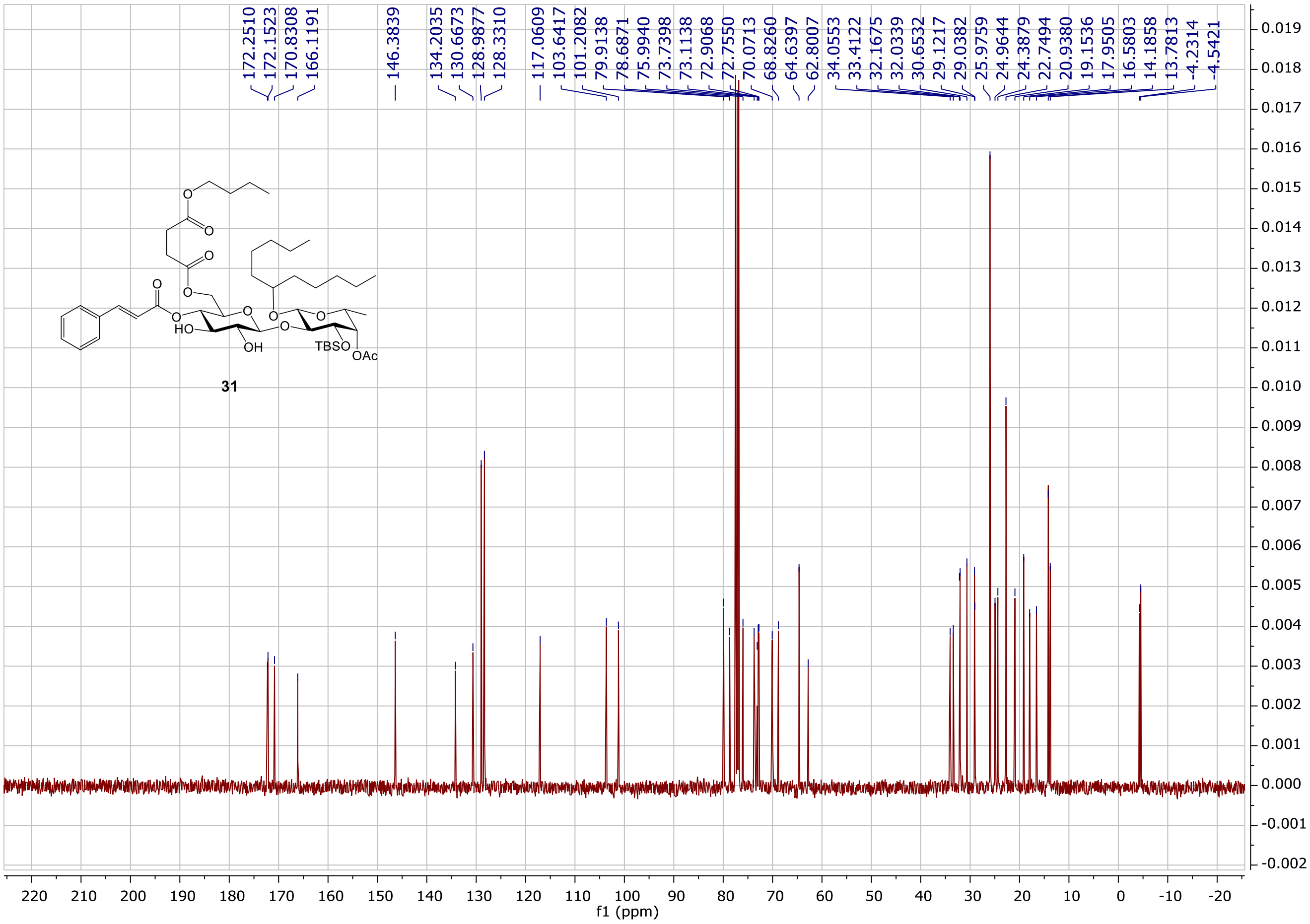
172.2510
172.1523
170.8308
166.1191
146.3839
134.2035
130.6673
128.9877
128.3310
117.0609
103.6417
101.2082
79.9138
78.6871
75.9940
73.7398
73.1138
72.9068
72.7550
70.0713
68.8260
64.6397
62.8007
34.0553
33.4122
32.1675
32.0339
30.6532
29.1217
29.0382
25.9759
24.9644
24.3879
22.7494
20.9380
19.1536
17.9505
16.5803
14.1858
13.7813
-4.2314
-4.5421
HO
OH
TBSO
OAc
31
f1 (ppm)

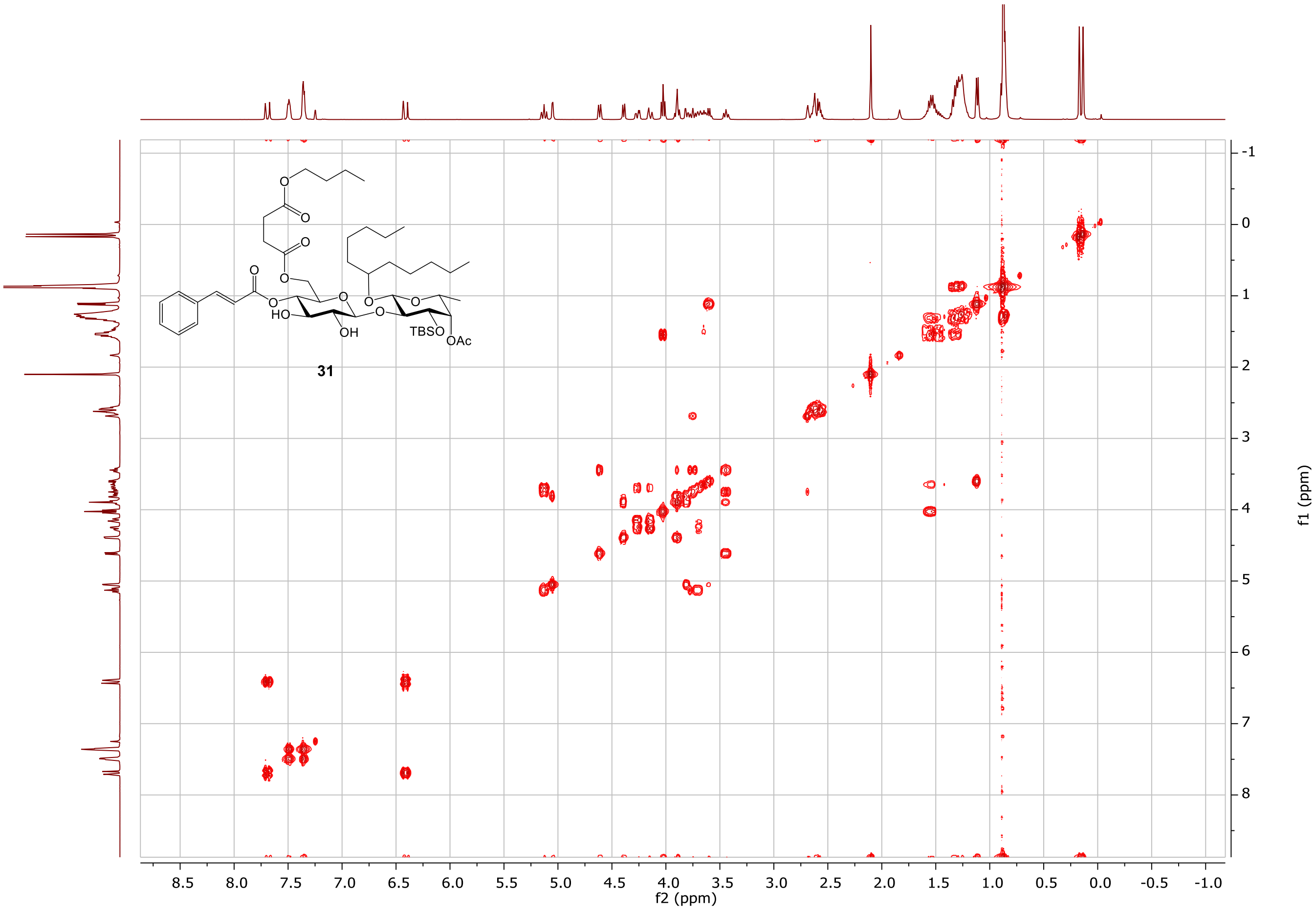

HO
OH
TBSO
OAc
31
f2 (ppm)
f1 (ppm)

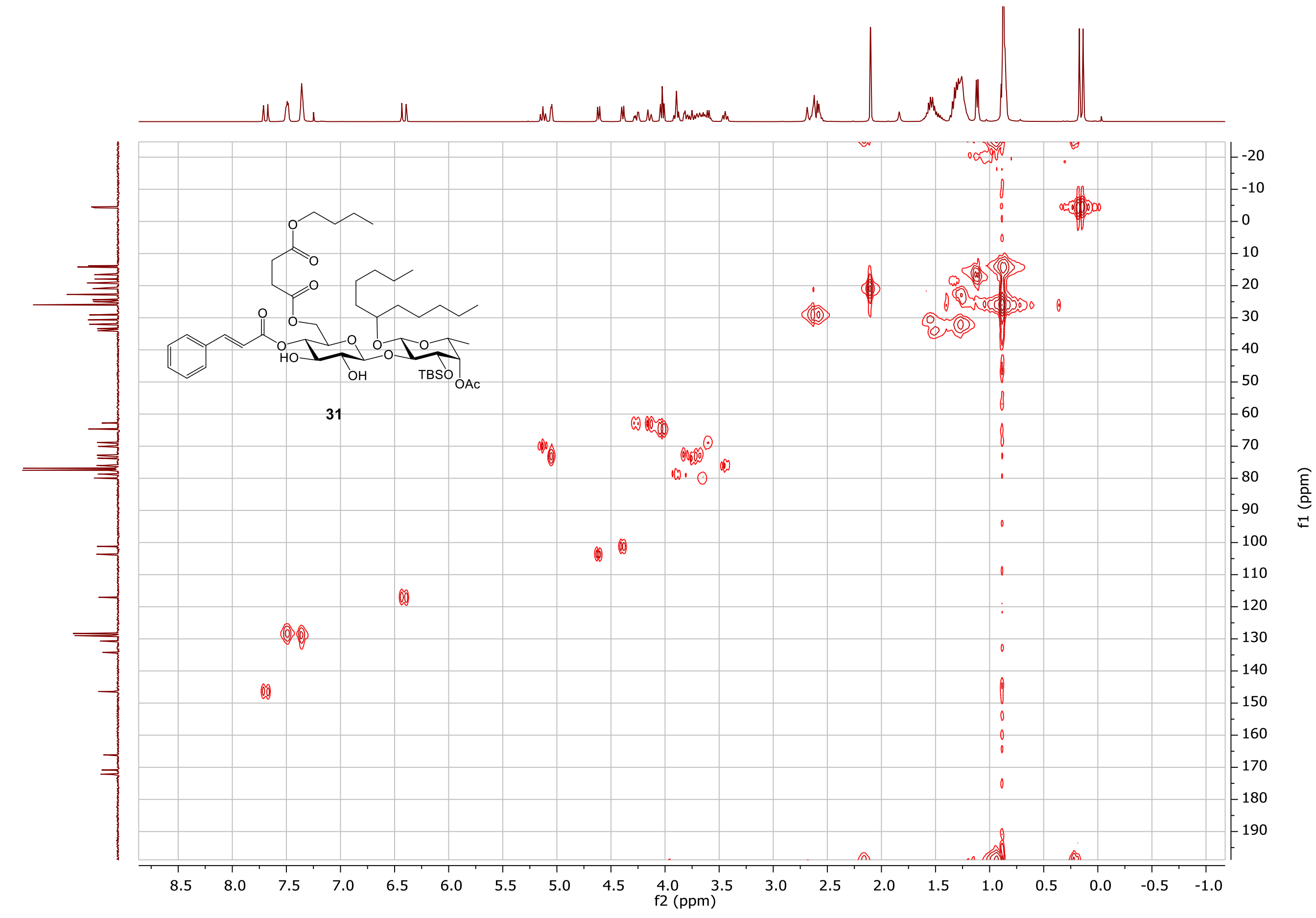

HO
OH
TBSO
OAc
31
f2 (ppm)
f1 (ppm)

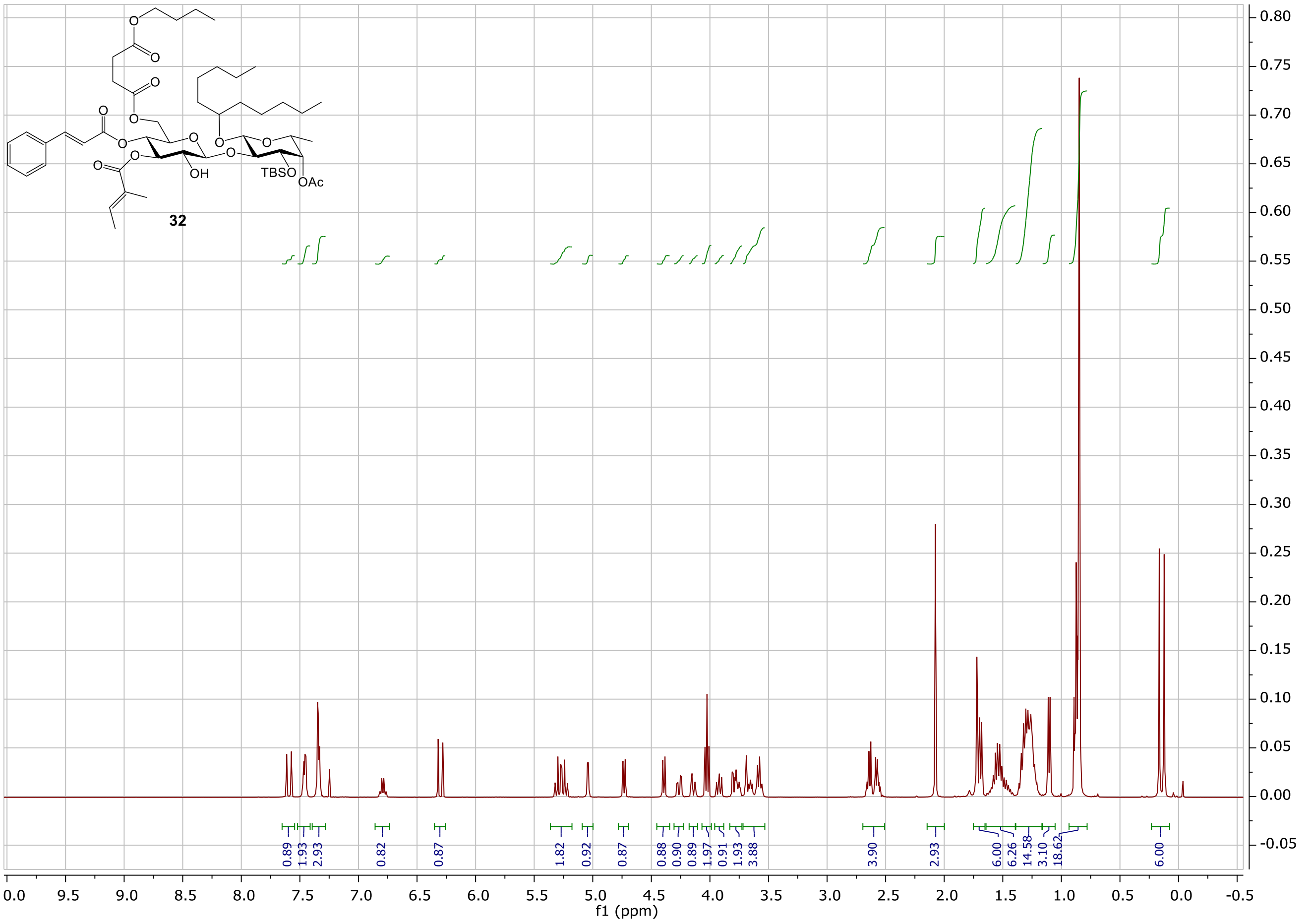

OH
TBSO
OAc
32
0.89
1.93
2.93
0.82
0.87
1.82
0.92
0.87
0.88
0.90
0.89
1.97
0.91
1.93
3.88
3.90
2.93
6.00
6.26
14.58
3.10
18.62
6.00
f1 (ppm)

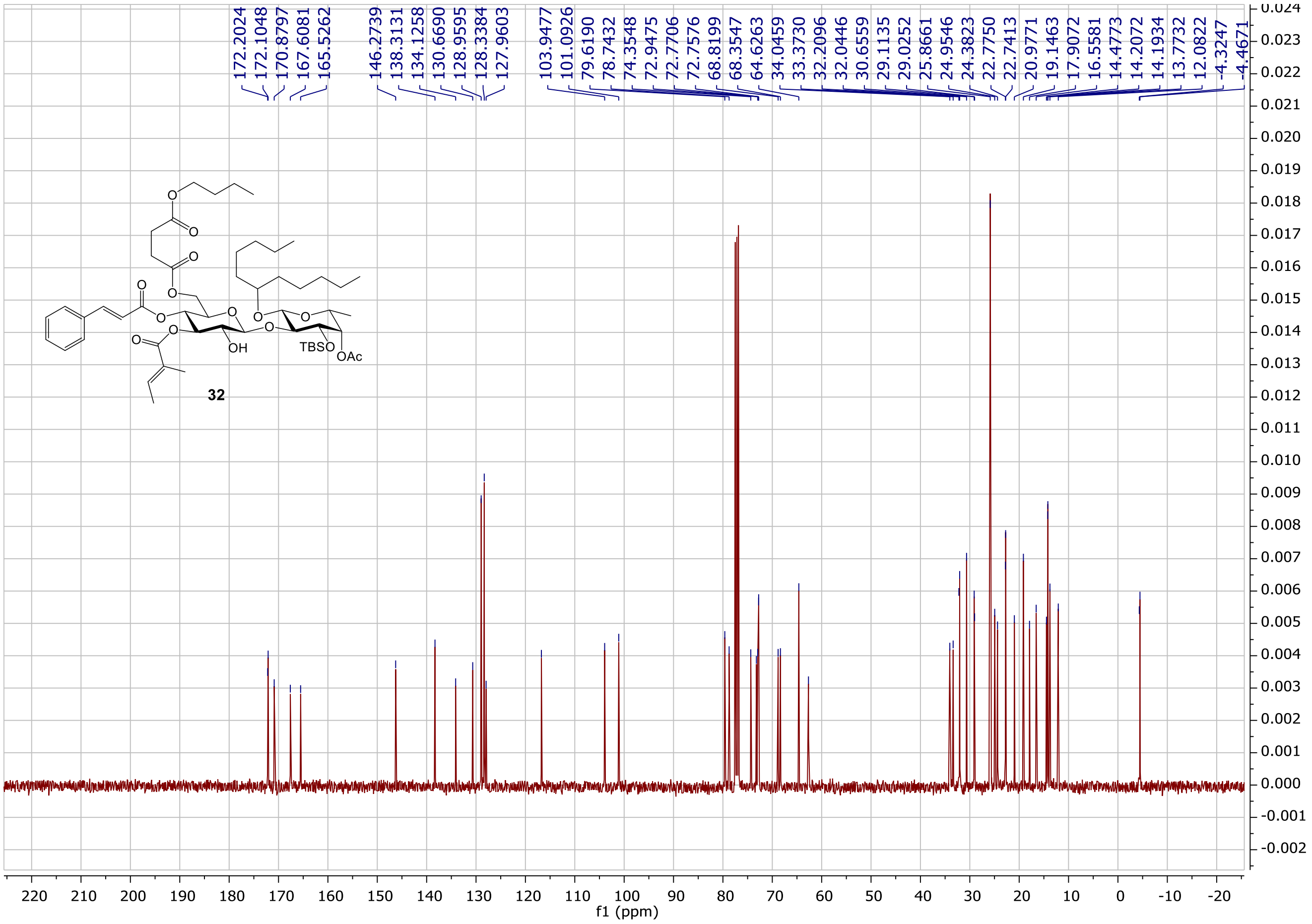

172.2024
172.1048
170.8797
167.6081
165.5262
146.2739
138.3131
134.1258
130.6690
128.9595
128.3384
127.9603
103.9477
101.0926
79.6190
78.7432
74.3548
72.9475
72.7706
72.7576
68.8199
68.3547
64.6263
34.0459
33.3730
32.2096
32.0446
30.6559
29.1135
29.0252
25.8661
24.9546
24.3823
22.7750
22.7413
20.9771
19.1463
17.9072
16.5581
14.4773
14.2072
14.1934
13.7732
12.0822
-4.3247
-4.4671
OH
TBSO
OAc
32
f1 (ppm)

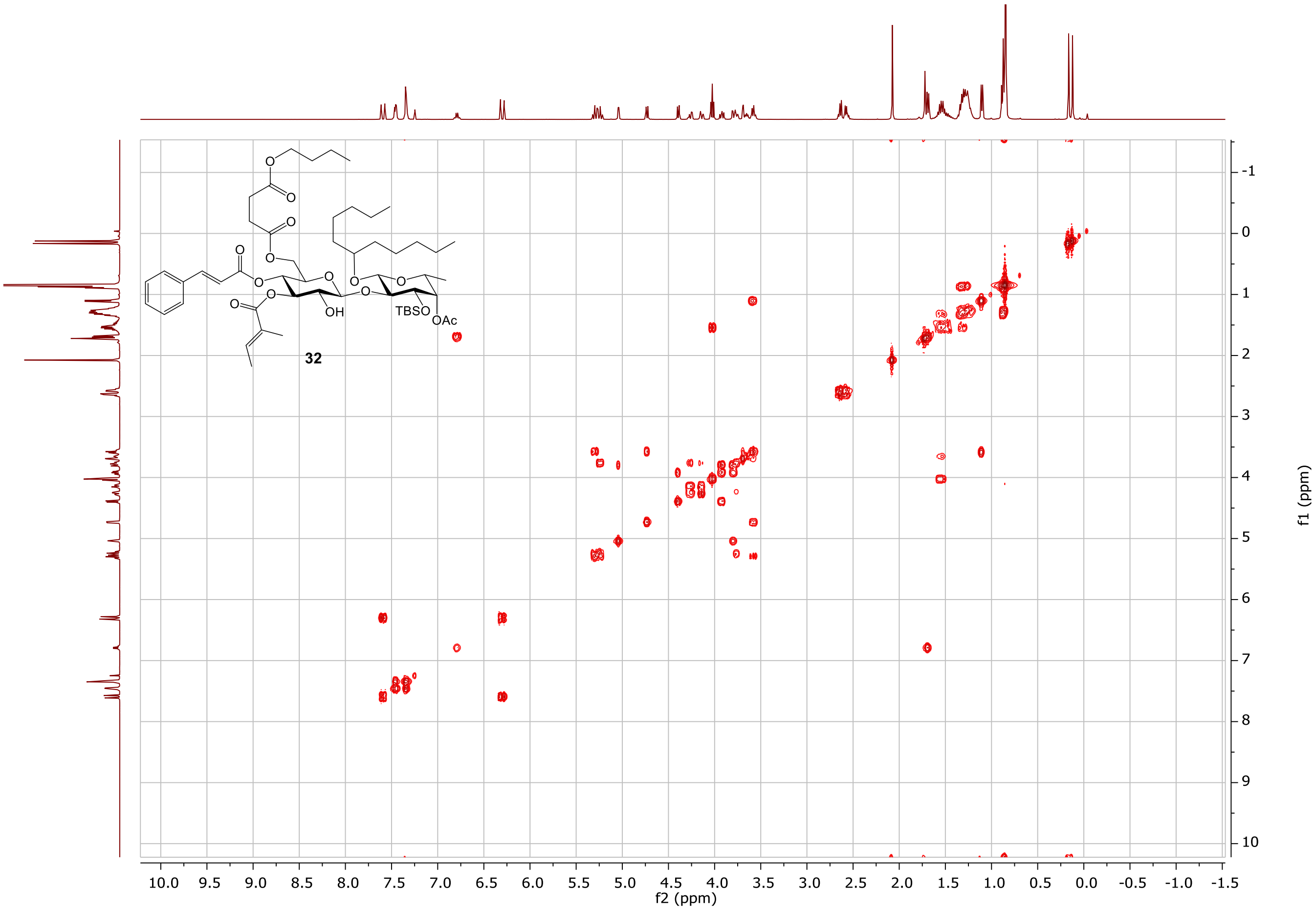

32
f2 (ppm)
f1 (ppm)

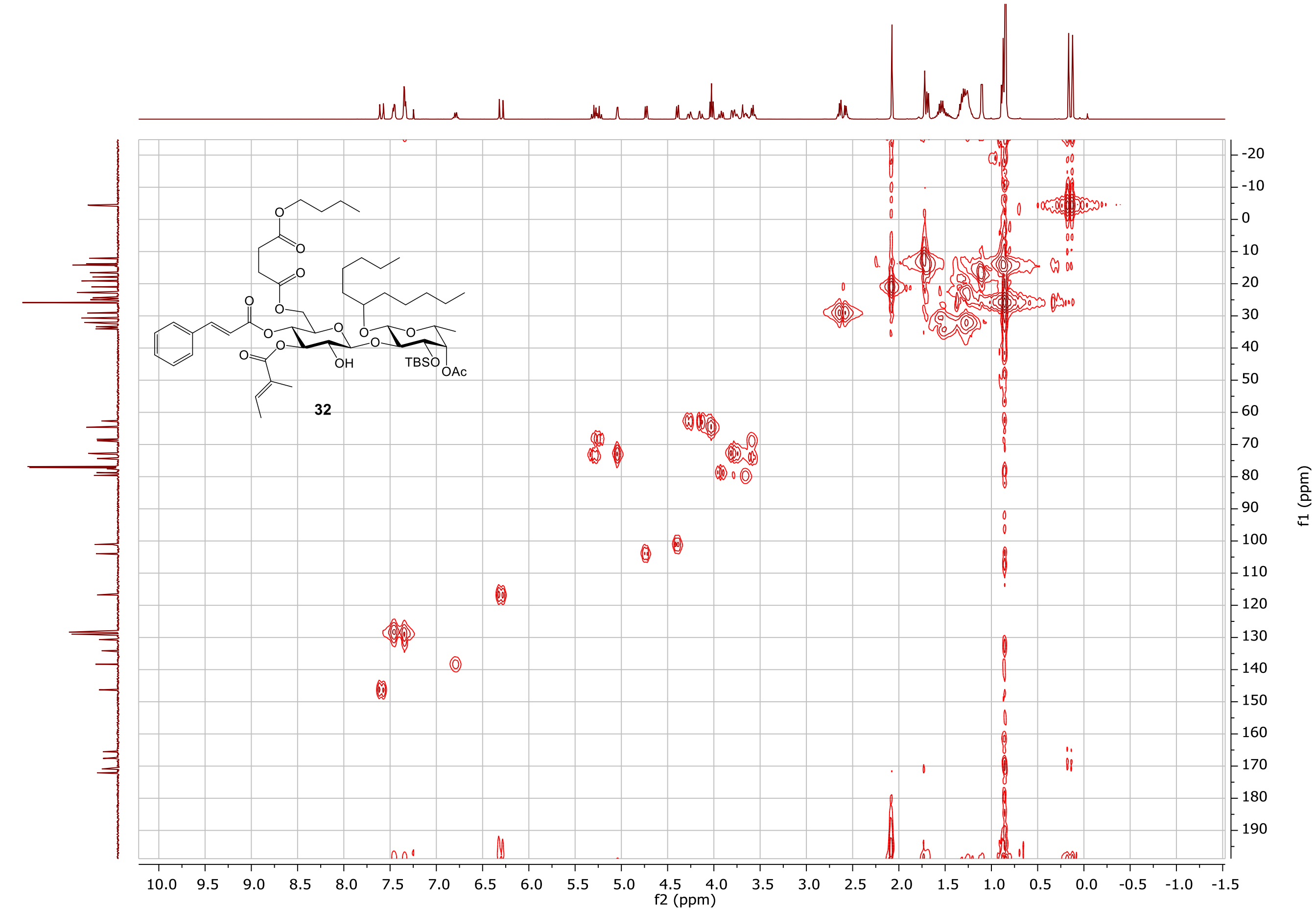

32
f2 (ppm)
f1 (ppm)

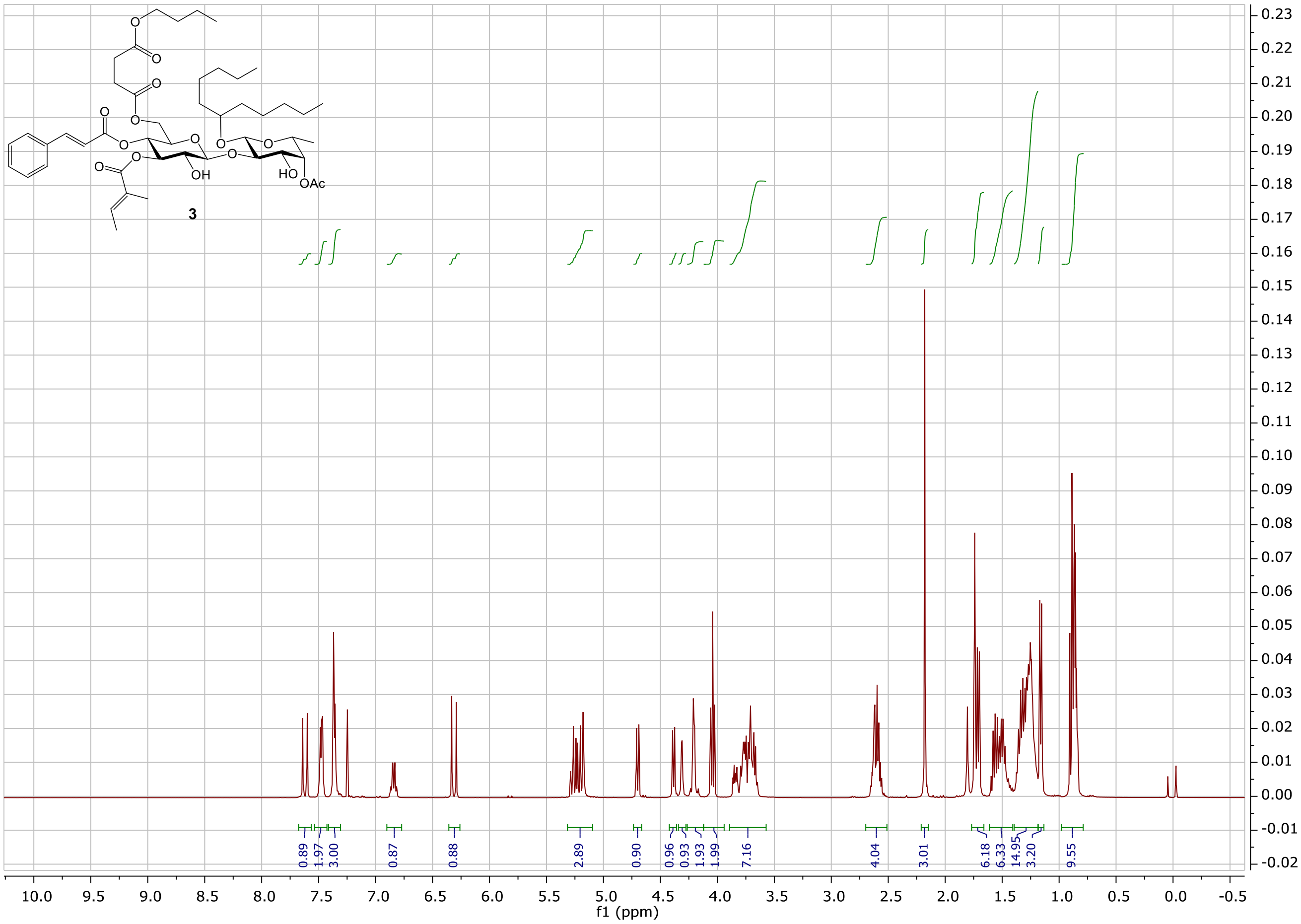

HO
OAc
OH
3
0.89
1.97
3.00
0.87
0.88
2.89
0.90
0.96
0.93
1.93
1.99
7.16
4.04
3.01
6.18
6.33
14.95
3.20
9.55
f1 (ppm)

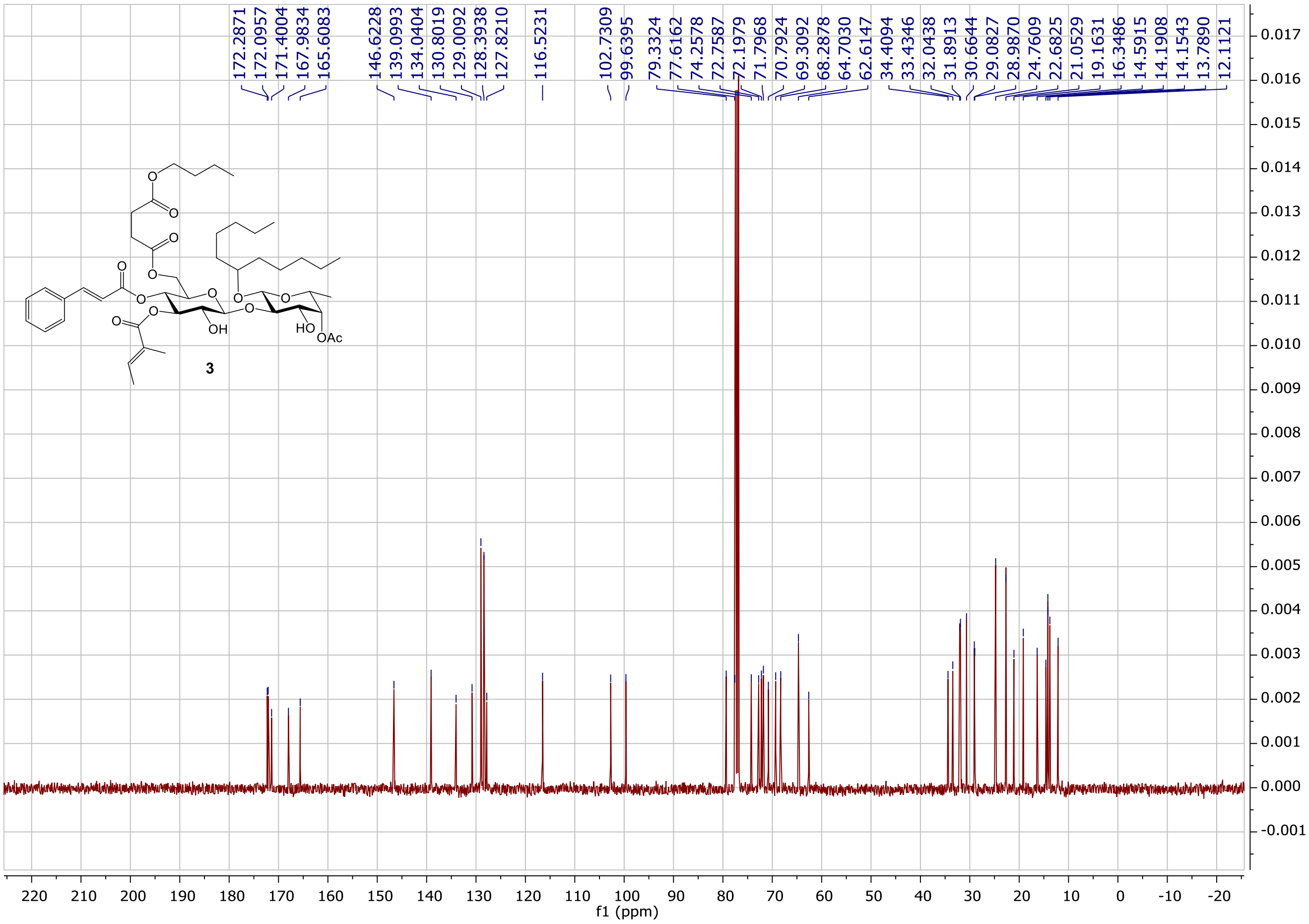

172.2871
172.0957
171.4004
167.9834
165.6083
146.6228
139.0993
134.0404
130.8019
129.0092
128.3938
127.8210
116.5231
102.7309
99.6395
79.3324
77.6162
74.2578
72.7587
72.1979
71.7968
70.7924
69.3092
68.2878
64.7030
62.6147
34.4094
33.4346
32.0438
31.8913
30.6644
29.0827
28.9870
24.7609
22.6825
21.0529
19.1631
16.3486
14.5915
14.1908
14.1543
13.7890
12.1121
HO
OAc
OH
3
f1 (ppm)

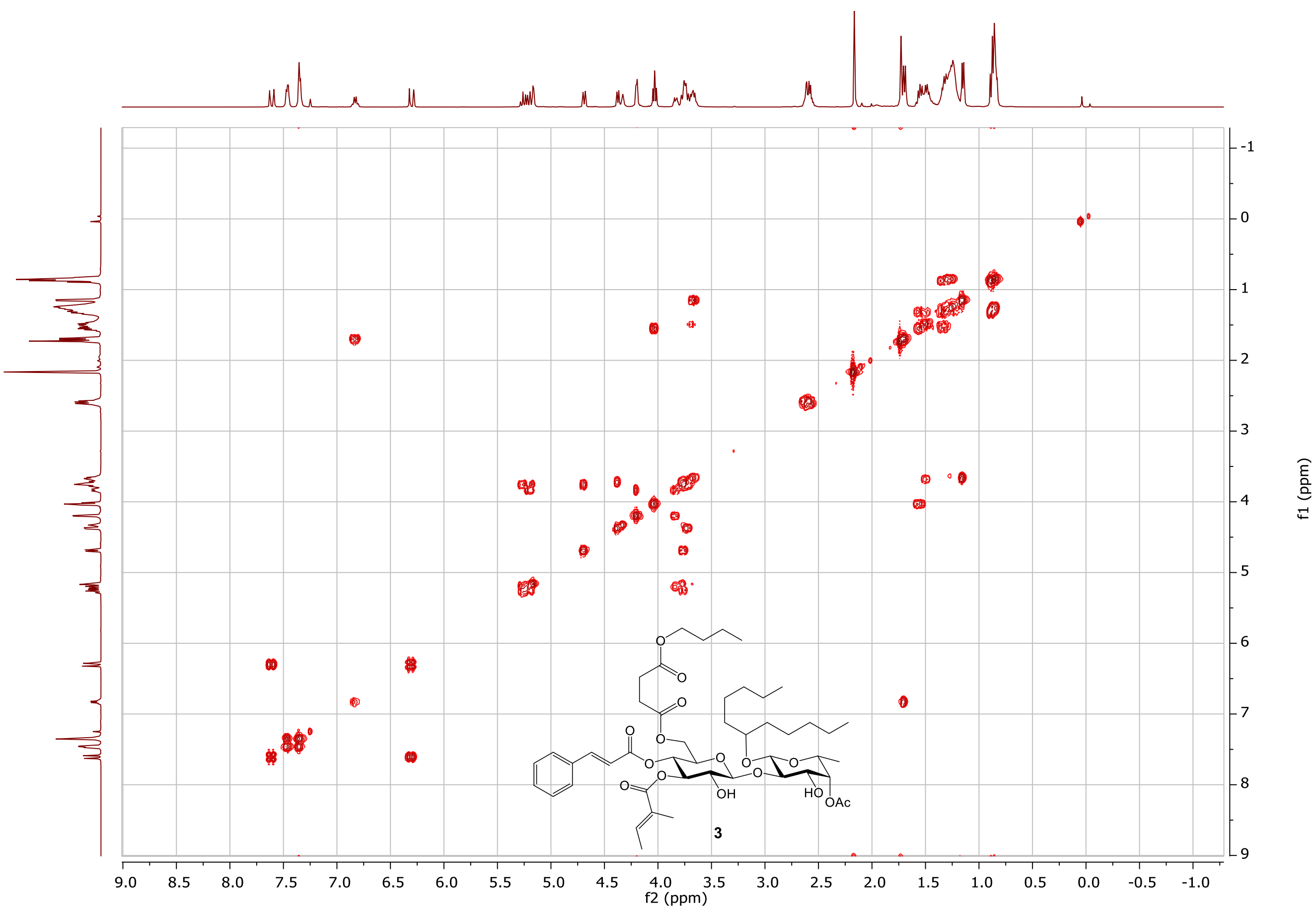
f2 (ppm)
f1 (ppm)
OH
HO
OAc
3

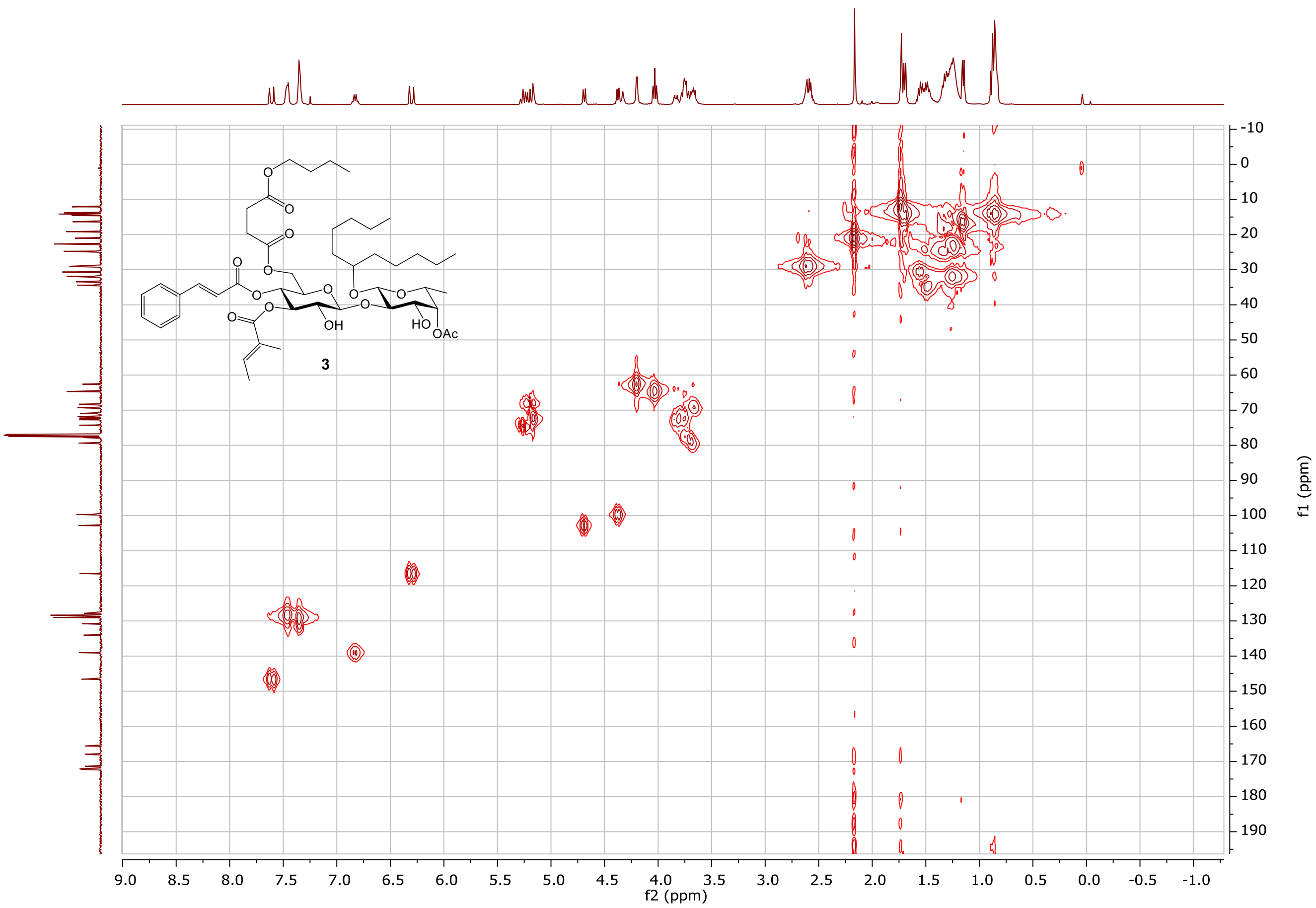
f2 (ppm)
f1 (ppm)
OH
HO
OAc
3

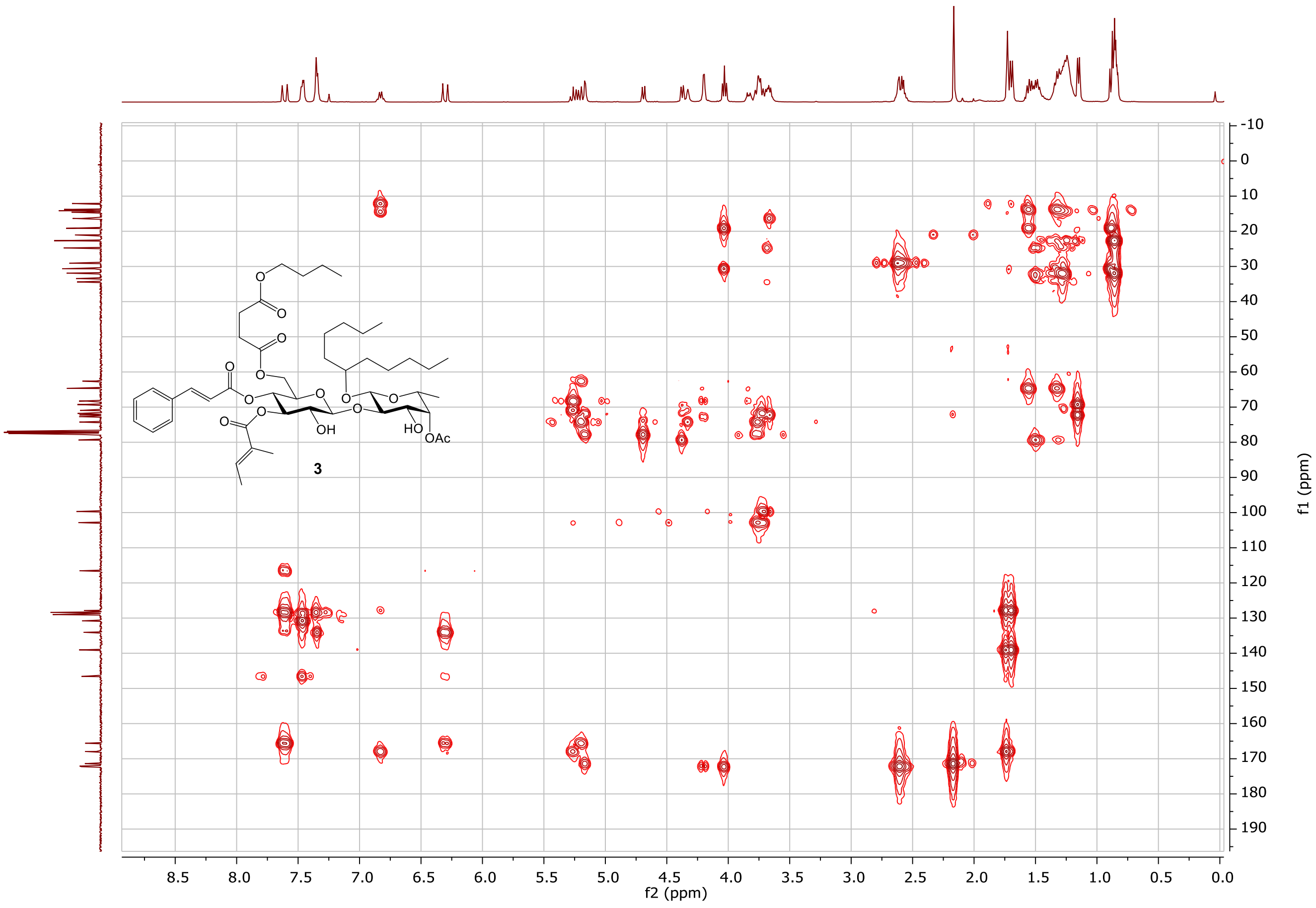

OH
HO
OAc
3
f2 (ppm)
f1 (ppm)
